# Elucidation of the relative and absolute stereochemistry of the kalimantacin/batumin antibiotics†
†Electronic supplementary information (ESI) available: Complete description of materials and methods and additional tables, figures and schemes including full ^1^H and ^13^C NMR data for all new compounds. See DOI: 10.1039/c7sc01670k



**DOI:** 10.1039/c7sc01670k

**Published:** 2017-07-11

**Authors:** Iain R. G. Thistlethwaite, Freya M. Bull, Chengsen Cui, Paul D. Walker, Shu-Shan Gao, Luoyi Wang, Zhongshu Song, Joleen Masschelein, Rob Lavigne, Matthew P. Crump, Paul R. Race, Thomas J. Simpson, Christine L. Willis

**Affiliations:** a School of Chemistry , University of Bristol , Cantock's Close , Bristol BS8 1TS , UK . Email: chris.willis@bristol.ac.uk; b Laboratory of Gene Technology , KULeuven , Leuven B-3001 , Belgium; c School of Biochemistry , University of Bristol , Bristol BS8 1TD , UK

## Abstract

Kalimantacin A and batumin exhibit potent and selective antibiotic activity against *Staphylococcus* species including MRSA. Both compounds are formed *via* a hybrid polyketide synthase/non-ribosomal peptide synthetase (PKS-NRPS) biosynthetic pathway and from comparison of the gene clusters it is apparent that batumin from *Pseudomonas batumici* and kalimantacin from *P. fluorescens* are the same compound. The linear structure of this unsaturated acid was assigned by spectroscopic methods, but the relative and absolute stereochemistry of the five stereocentres remained unknown. Herein we describe isolation of kalimantacin A and two further metabolites 17,19-diol **2** and 27-descarbomyl hydroxyketone **3** from cultures of *P. fluorescens*. Their absolute and relative stereochemistries are rigorously determined using a multidisciplinary approach combining natural product degradation and fragment synthesis with bioinformatics and NMR spectroscopy. Diol **2** has the 5*R*, 15*S*, 17*S*, 19*R*, 26*R*, 27*R* configuration and is the immediate biosynthetic precursor of the bioactive kalimantacin A formed by oxidation of the 17-alcohol to the ketone.

## Abstract 2

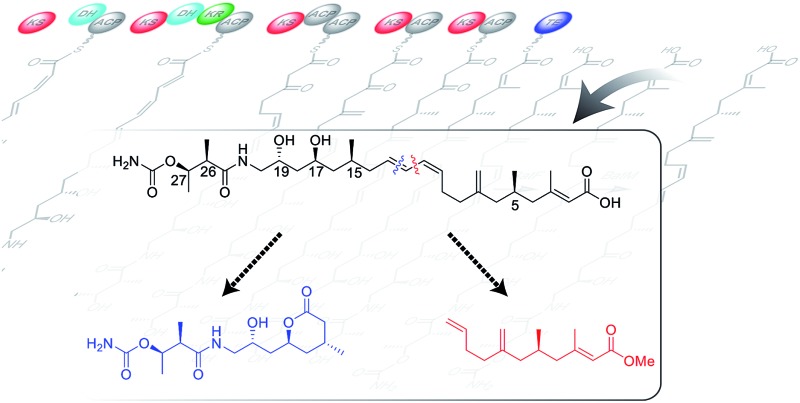
A multidisciplinary approach combining natural product degradation, fragment synthesis, bioinformatics and NMR spectroscopy was used.

## Introduction

Polyketides isolated from bacteria exhibit a range of important bioactivities making them attractive leads for the development of therapeutics,^1^ for example mupirocin is used worldwide as a topical antibiotic.^2^ These compounds are efficiently assembled in the host microorganism *via* sophisticated multiple enzyme architectures known as modular polyketide synthases. The rational alteration of these biosynthetic pathways offers exciting opportunities to access novel bioactive agents.^3^

**Scheme 1:**
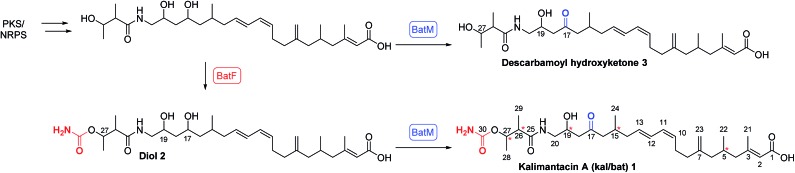
Proposed final stages of the biosynthesis of kalimantacin A.

In the course of a screening program for novel antibiotics, the kalimantacins were isolated from cultures of *Alcaligenes* sp. YL-02632S and the major metabolite, kalimantacin A **1** was shown to exhibit activity against Gram-positive bacteria including MRSA (Scheme 1).^4,5^

A short time later the antibiotic batumin was isolated from *Pseudomonas batumici* and found to have the same molecular weight and spectroscopic properties as kalimantacin A.^6,7^ Batumin has been patented in the Ukraine and formulated as Diastaf and used to detect staphylococci by taking advantage of its selectivity for these strains.^8^ Decreased biofilm formation has also been reported for the majority of *S. aureus* strains investigated^9^ and it has been found to reduce nasal *S. aureus* carriage.^10^ Peschel and co-workers have recently highlighted the potential scope of narrow spectrum antibiotics in the control of bacterial populations.^11^

In 2010 Lavigne and co-workers isolated a metabolite from cultures of *Pseudomonas fluorescens* BCCM_ID9359 which again had spectroscopic properties in accord with the structure of kalimantacin and batumin hence it was named kal/bat (Scheme 1). The kal/bat gene cluster was identified and characterized and shown to consist of 16 open reading frames encoding a hybrid modular polyketide synthase/non-ribosomal peptide synthetase (PKS-NRPS) system.^12,13^ It has recently been established that the biosynthetic gene clusters of kalimantacin from *P. fluorescens* and batumin from *P. batumici* ^14,15^ are identical, confirming that they both produce the same natural product based on a structural framework assembled on a linear unsaturated acid featuring five stereocenters. The PKS-NRPS is a member of the *trans*-acyltransferase (AT) class of modular polyketide synthases that lack integral AT-domains whereby they gain AT and other tailoring activities in *trans*.^16,17^ This can dramatically increase the diversity and complexity of the chemical transformations available to the pathway and offers significant potential for pathway engineering of new compounds. New metabolites diol **2**, with a 17-hydroxyl group rather than carbonyl, and descarbamoyl compound **3** were isolated by inactivation of the last two tailoring genes *batF* (carbamoyl transferase) and *batM* (oxidoreductase) of the *P. fluorescens* gene cluster (Scheme 1).

The final conversion by BatM was particularly interesting as it transformed the almost inactive precursor, diol **2**, to the bioactive 17-ketone **1**. This key step was hypothesized to be essential for bacterial physiology whereby production of the inactive precursor functions as a pro-drug to avoid self-toxicity.^12,13^ The mode of action of kal/bat was suggested to be inhibition of bacterial fatty acid biosynthesis, due to the presence of a FabI isoform, namely BatG and the antibiotic has high specific activity against staphylococci.^13,18^ Kalimantacin/batumin has potential value as a lead antibiotic, exhibiting selective and high specific activity against staphylococci (MIC value of 0.064 μg mL^–1^) and is significantly more active than other antibiotics in commercial use (*e.g.* mupirocin MIC *ca.* 0.5 μg mL^–1^).

The relative and absolute structures of kal/bat **1** and putative biosynthetic intermediates **2** and **3** have remained elusive using spectroscopic methods or *via* attempts to generate a crystalline derivative for X-ray studies. With 32 possible isomers it is important to determine the full structure of this antibiotic. Herein we report a multidisciplinary approach combining natural product isolation, chemical degradation and fragment synthesis with bioinformatics and extensive NMR studies to determine the relative and absolute configuration of **1** and **2** isolated from *P. fluorescens*. For simplicity we use the name kalimantacin.

## Results and discussion

Initially, preparative amounts of pure kalimantacin A **1** were isolated from *P. fluorescens* for degradation and derivatization studies. We found that higher titres were obtained by growing cultures in a modified L-medium rather than a tryptose broth supplemented with sucrose and glycine as reported previously.^12^ The natural product was readily purified, using either normal phase followed by reversed phase silica chromatography or HPLC to yield typically 50 mg L^–1^ of kalimantacin A. A similar protocol was used to grow the Δ*batF* and Δ*batM* mutants of *P. fluore*s*cens* giving biosynthetic intermediates **2** and **3**. The spectral data of **1**, **2** and **3** were in accord with those reported previously^12^ and these compounds were found to be stable for several weeks at room temperature.

**Scheme 2:**
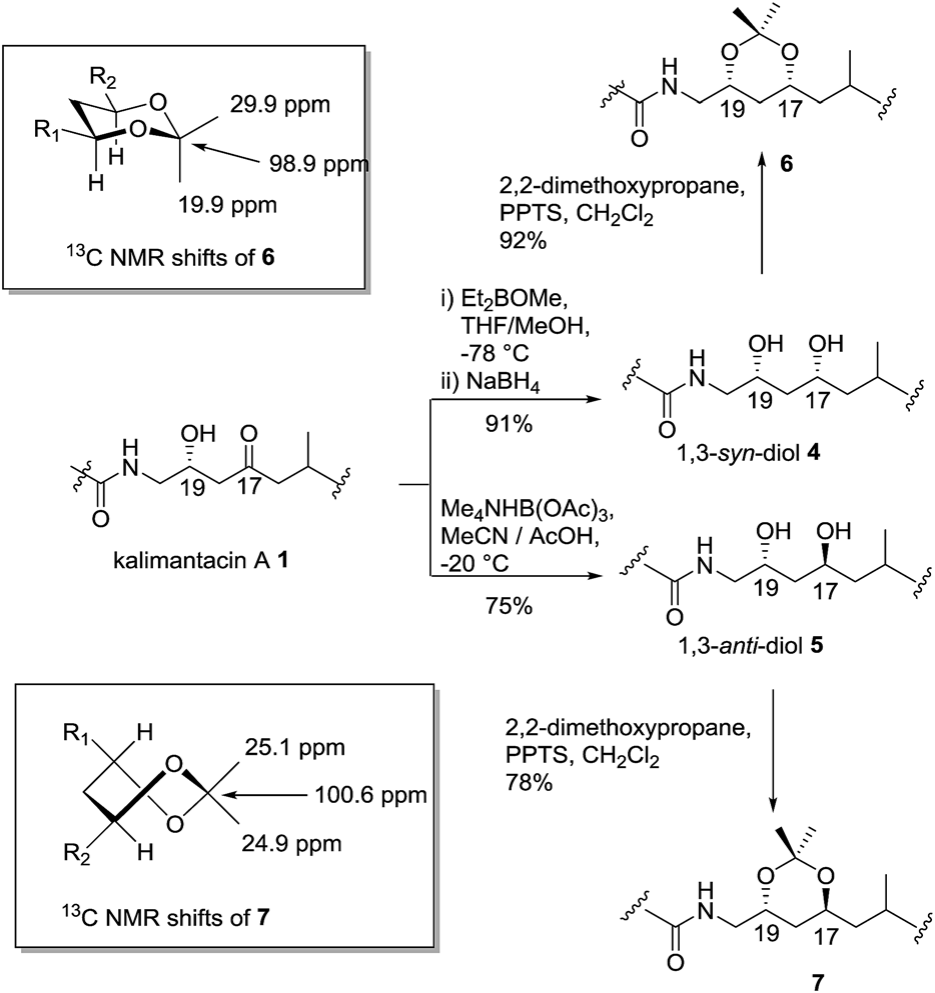
Stereoselective reduction of kalimantacin A **1** and acetonide formation.

The first structural investigation was the relative stereochemistry of the 17,19-diol in **2** isolated from the Δ*batM* mutant of *P. fluorescens*. Directed reductions^19,20^ of the β-hydroxyketone moiety of kalimantacin A **1** gave 1,3-*syn* and 1,3-*anti* diols **4** and **5** for comparison with diol **2** (Scheme 2). The optical rotation of synthetic *anti* diol **5**, [*α*]_D_ –10.0 and natural product **2**, [*α*]_D_ –10.4 correlated well and their ^1^H and ^13^C NMR spectra were identical. In contrast 1,3-*syn* diol **4** with [*α*]_D_ –5.9 showed significant differences in the chemical shifts of C-17 (*δ*_c_ 68.4 for **2**, *δ*_c_ 70.3 for **4**) and C-19 (*δ*_c_ 66.3 for **2**, *δ*_c_ 72.0 for **4**) (Tables 1 and 2, ESI†).

The assignment of the relative stereochemistry of the 1,3-*syn* and 1,3-*anti* diols **4** and **5** was confirmed using the method of Rychnovsky and Evans^21^ from the characteristic quaternary and methyl group ^13^C chemical shifts of the corresponding acetonides **6** and **7** (Scheme 2). Similarly the natural diol **2** was converted to its acetonide and was the same by NMR to **7** confirming the *anti* relationship in the 17,19 diol. Bioinformatic analysis of active site motifs of the appropriate modular ketoreductase (KR) domains predicts that the 17-alcohol should be introduced with the “*R*” configuration on reduction of the β-keto–thiol ester assembly intermediate.^12^ Hence taking into account the Cahn–Prelog–Ingold priority change upon further chain elongation, we made a preliminary assignment of the absolute configuration for diol **2** as 17*S*, 19*R*.

**Fig. 1:**
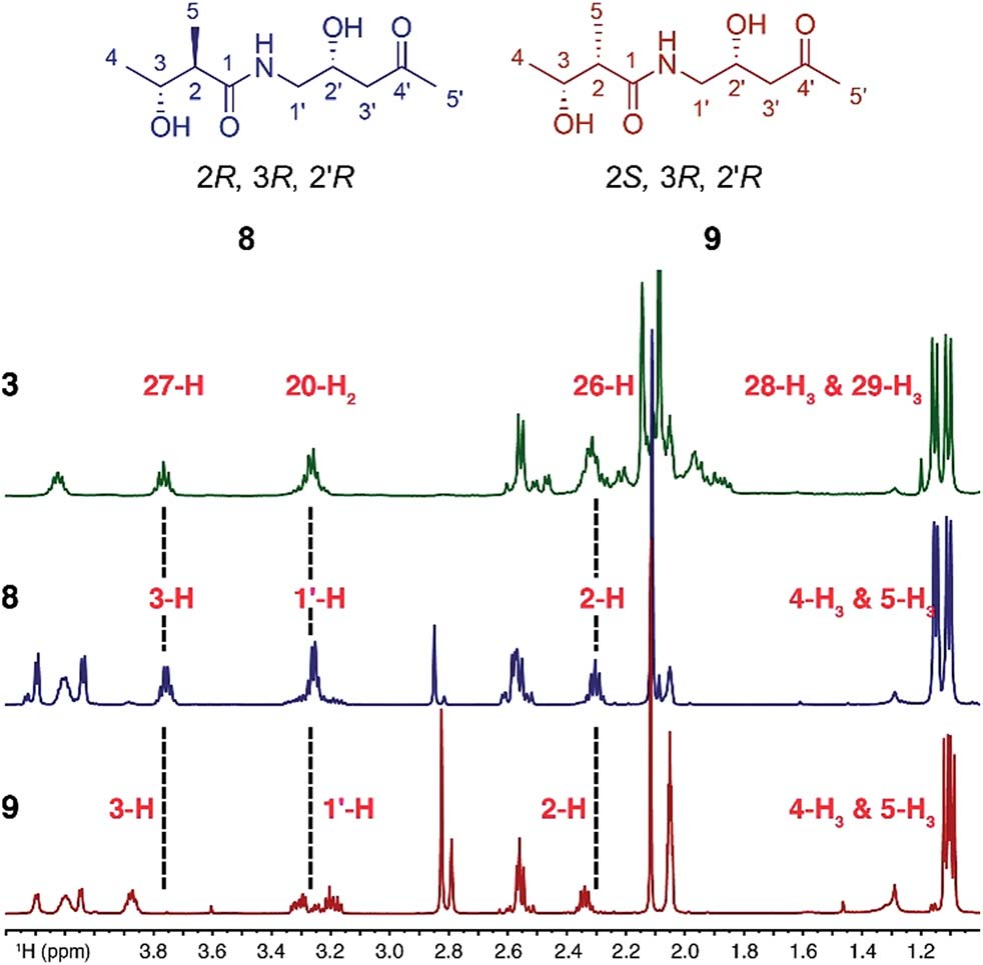
^1^H NMR data comparison for synthetic fragments **8** and **9** with 27-descarbamoyl metabolite **3**.

Sequence analysis of the relevant KR domain suggested the *R* configuration at C27 but this, as well as the relative stereochemistry of C26 and C27, remained to be proven.^12^ Thus, two fragment mimics, (2*R*,3*R*,2′*R*)-diol **8** and (2*S*,3*R*,2′*R*)-diol **9**, of kalimantacin A were prepared and varied in the configuration of the 2-methyl groups (see ESI for synthetic details†). Their NMR spectra were compared with those of the descarbamoyl metabolite **3** isolated from the Δ*batF* mutant of *P. fluorescens* and the best fit was with (2*R*,3*R*,2′*R*)-diol **8** (Fig. 1). To support the proposed relative stereochemistry of the 19-alcohol and 26,27-stereocentres, the analogous (2*R*,3*R*,2′*S*)-diol was also synthesized and, as expected, the NMR data of this compound gave a poor fit with the natural product **3** (ESI, Tables 3 and 4, Fig. S2 and S3†).

**Scheme 3:**
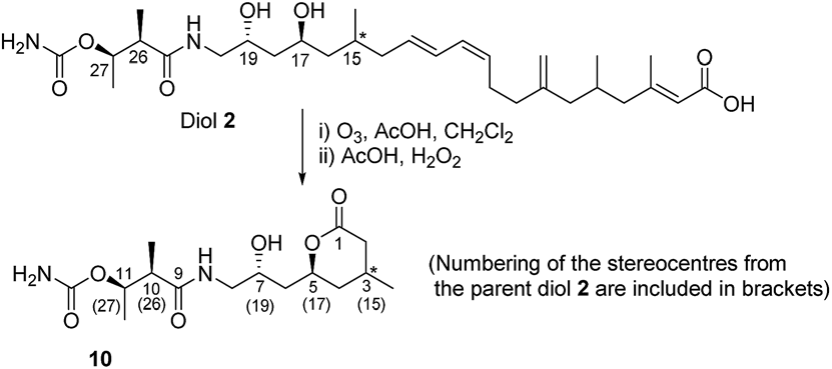
Oxidative cleavage of diol **2**.

With the above information in hand, it was necessary to determine the configuration of the C-15 stereocenter as well as confirm the tentative assignment of absolute stereochemistry (17*S*, 19*R*, 26*R*, 27*R*) for diol **2**. We turned to natural product degradation and comparison with a synthetic standard (Scheme 3). Ozonolysis of diol **2** followed by treatment with AcOH/H_2_O_2_ led to cleavage of the 12,13-double bond to give a dihydroxy acid which cyclized, after purification by HPLC, to give lactone **10** as a single diastereomer with [*α*]_D_ +25.0.

**Fig. 2:**
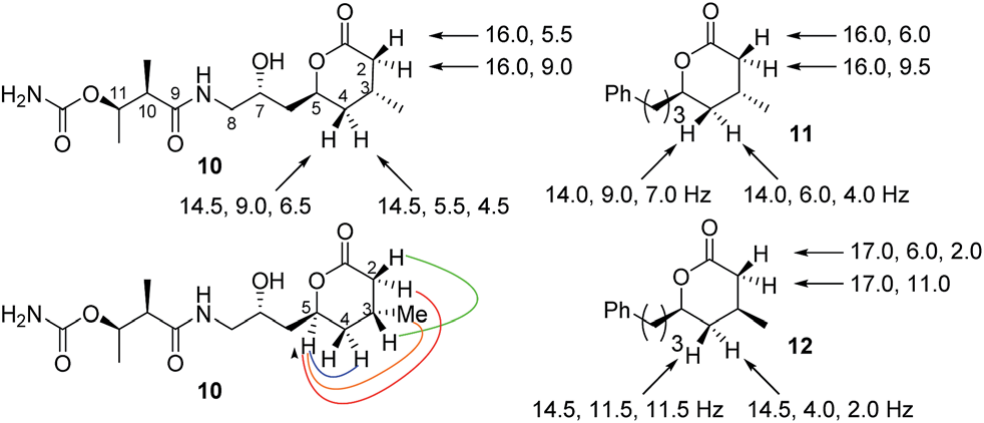
Selected NMR coupling constants for lactones **10**, **11** and **12** and nOe correlations for **10**.

From a combination of TOCSY and NOESY it was apparent that the ring adopted a boat conformation with NOESY correlations from 5-H to 2α-H, 4α-H and 3-CH_3_ consistent with these being on the same face of the molecule (Fig. 2). A NOESY correlation between 2β-H and 3-H, which was in accord with the methyl group at C-3 having a relative *anti* relationship with the C-5 side-chain so that in the ‘open chain structure’ it would have a *syn* relationship with the C-5 alcohol.

Further support came from comparing the coupling constants for the ring protons in lactone **10** with those reported for *anti* and *syn* lactones **11** and **12** ^22^ (Fig. 2). The signals assigned to 4-H_2_ in **11** and **12** have significantly different couplings constants and the NMR data for *anti* lactone **11** correlated well with those for degradation product **10**.

**Scheme 4:**
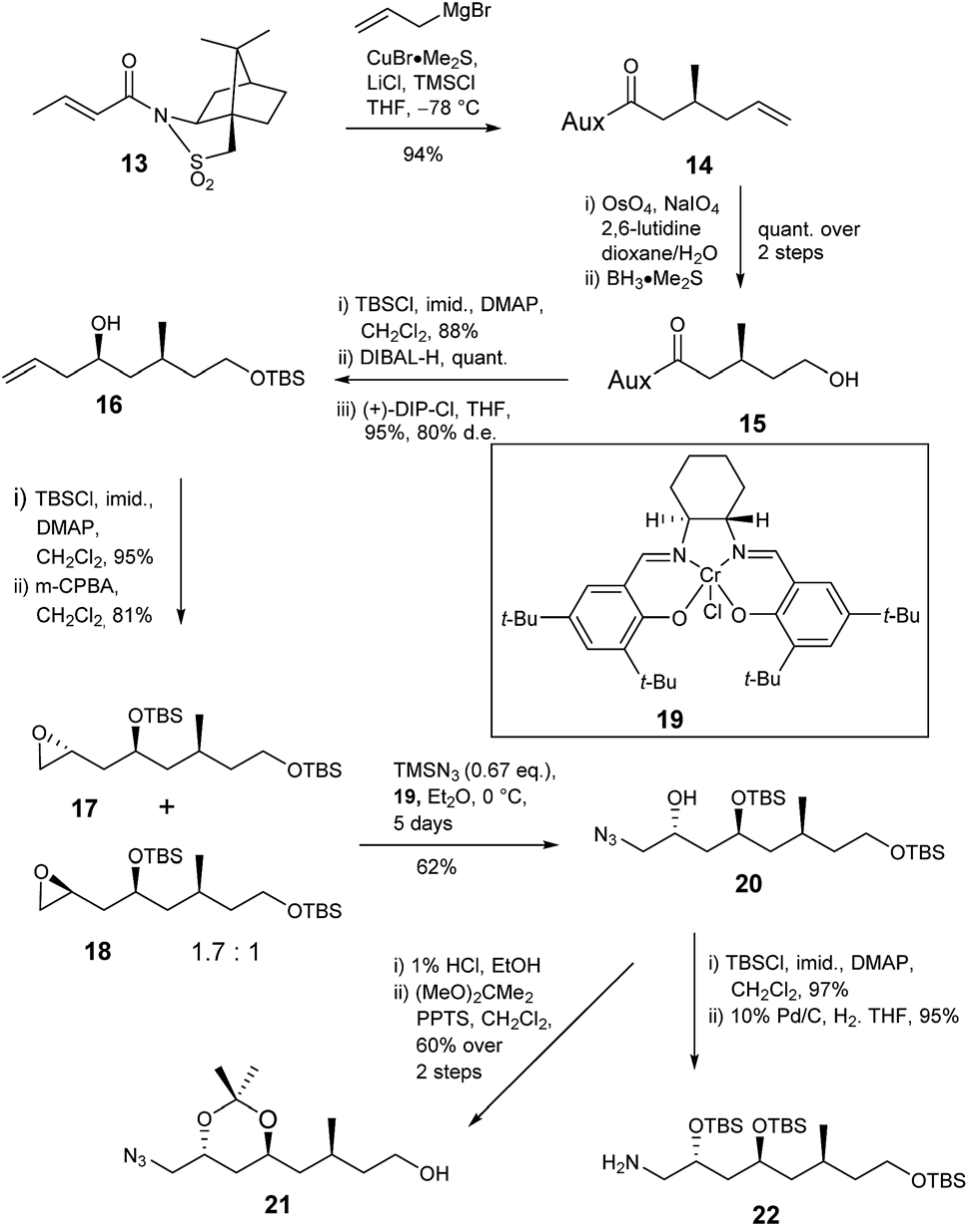
Synthesis of amine **22**.

**Scheme 5:**
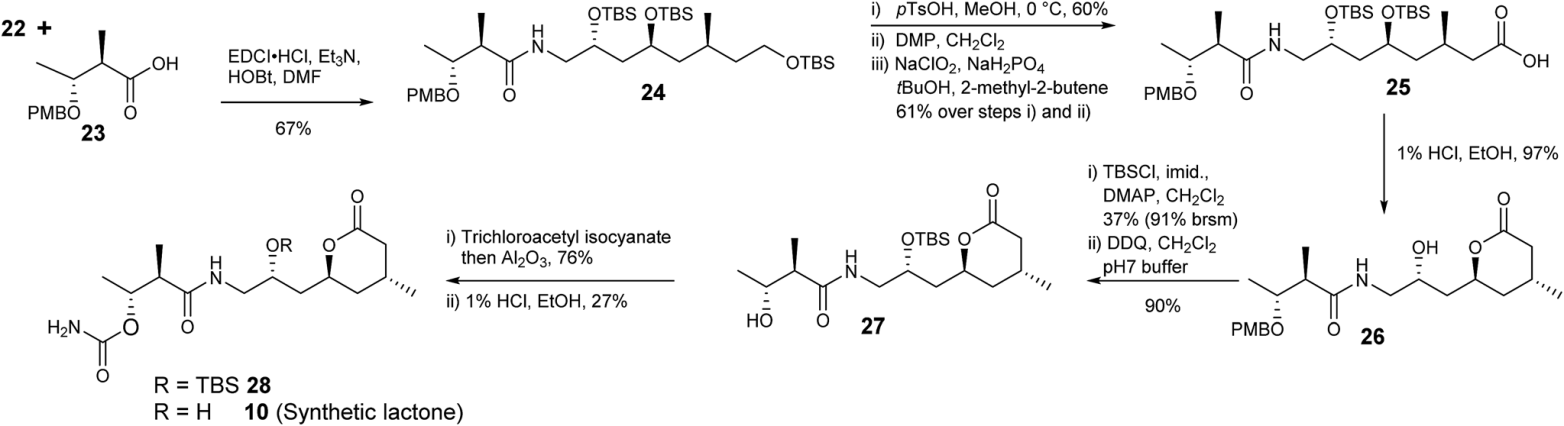
Preparation of lactone **10**.

The deduced structure and absolute configuration of degradation product **10** were confirmed by total synthesis (Schemes 4 and 5). Addition of a copper mediated Grignard reagent to chiral crotonate **13** using the conditions reported by Lipshutz^23^ gave the known allylated sultam **14** generating the 3-methyl stereocenter in the target lactone. Oxidative cleavage of the alkene using OsO_4_/NaIO_4_ in the presence of 2,6-lutidine^24^ followed by reduction of the resultant aldehyde gave alcohol **15** in quantitative yield. Addition of lutidine was important as without it only a 15% yield of aldehyde was obtained. Protection of alcohol **15** as the TBS ether and reductive cleavage of the auxiliary gave an aldehyde which was immediately subjected to Brown allylation conditions with (+)-DIP-Cl^25,26^ giving alkene **16** in 83% yield over the 3 steps. The stereochemical outcome of the reaction was confirmed by analysis of the (*R*)- and (*S*)-Mosher's ester^27,28^ derivatives of alcohol **16**.

Next the amine and the further required protected alcohol were introduced *via* manipulation of the terminal alkene of **16** (Scheme 4). 1-Azido-2-trimethylsiloxanes have been prepared *via* kinetic resolution of terminal epoxides using TMSN_3_ and a chiral bidentate salen ligand.^29^ Thus alcohol **16** was protected as the TBS ether then treatment with MCPBA gave a 1.7 : 1 mixture of epoxides **17** and **18** in 81% yield. The mixture was treated with chiral salen complex **19** and TMSN_3_ giving azide **20** (62%) and unreacted epoxide **18** (31%) which were readily separated by column chromatography. The *anti* relationship of the protected 1,3-diol was confirmed by deprotection of silyl ether **20** and conversion of the resultant *anti* 1,3-diol to acetonide **21** which gave characteristic ^13^C NMR signals at *δ*_c_ 100 for the quaternary carbon and *δ*_c_ 24.7, 24.8 for the methyl groups. TBS protection of secondary alcohol **20** and reduction of the azide gave amine **22** required for coupling to the butanoic acid derivative.

Acid **23** was readily prepared from ethyl (2*R*,3*R*)-3-hydroxy-2-methylbutanoate^30,31^ *via* formation of the *p*-methoxybenzyl ether followed by hydrolysis of the ester with barium hydroxide (Scheme 5). Amine **22** and acid **23** were coupled giving **24** with the required carbon framework of the target lactone. Further functional group manipulations included selective deprotection of the primary silyl ether **24** then a two-step oxidation protocol to give carboxylic acid **25** using DMP followed by a Pinnick oxidation.^32^

Treatment of **25** with 1% HCl in EtOH generated lactone **26**. To complete the synthesis of **10** the secondary alcohol in **26** was protected as the silyl ether and the benzyl ether removed with 2,3-dichloro-5,6-dicyanobenzoquinone (DDQ) giving **27**. The carbamoyl group was introduced using trichloroacetylisocyanate in CH_2_Cl_2_ followed by stirring with alumina to give **28** in 76% yield. Finally removal of the silyl ether with 1% HCl in EtOH gave lactone **10**.

The ^1^H- and ^13^C NMR data of synthetic lactone **10** and the product isolated from ozonolysis of metabolite **2** were the same (Table 5, ESI†). Furthermore the optical rotation of the two samples were entirely consistent. Thus, we can confirm the absolute and relative configuration of the C14–C28 fragment of diol **2** is as shown in Scheme 5.

**Scheme 6:**
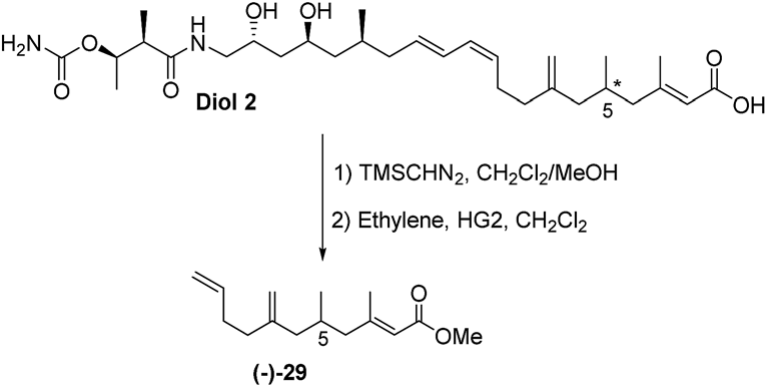
Methylation of diol **2** followed by degradation to ester **(–)-29**.

The final challenge was to determine the stereochemistry of the remote C-5 stereocentre. We again applied a combination of degradation studies and chemical synthesis. Diol **2** was methylated with TMS diazomethane then treated with 2^nd^ generation Hoveyda–Grubbs catalyst^33^ under an ethylene atmosphere to give the degradation product **(–)-29** with [*α*]_D_ –6.8 (Scheme 6).

**Scheme 7:**
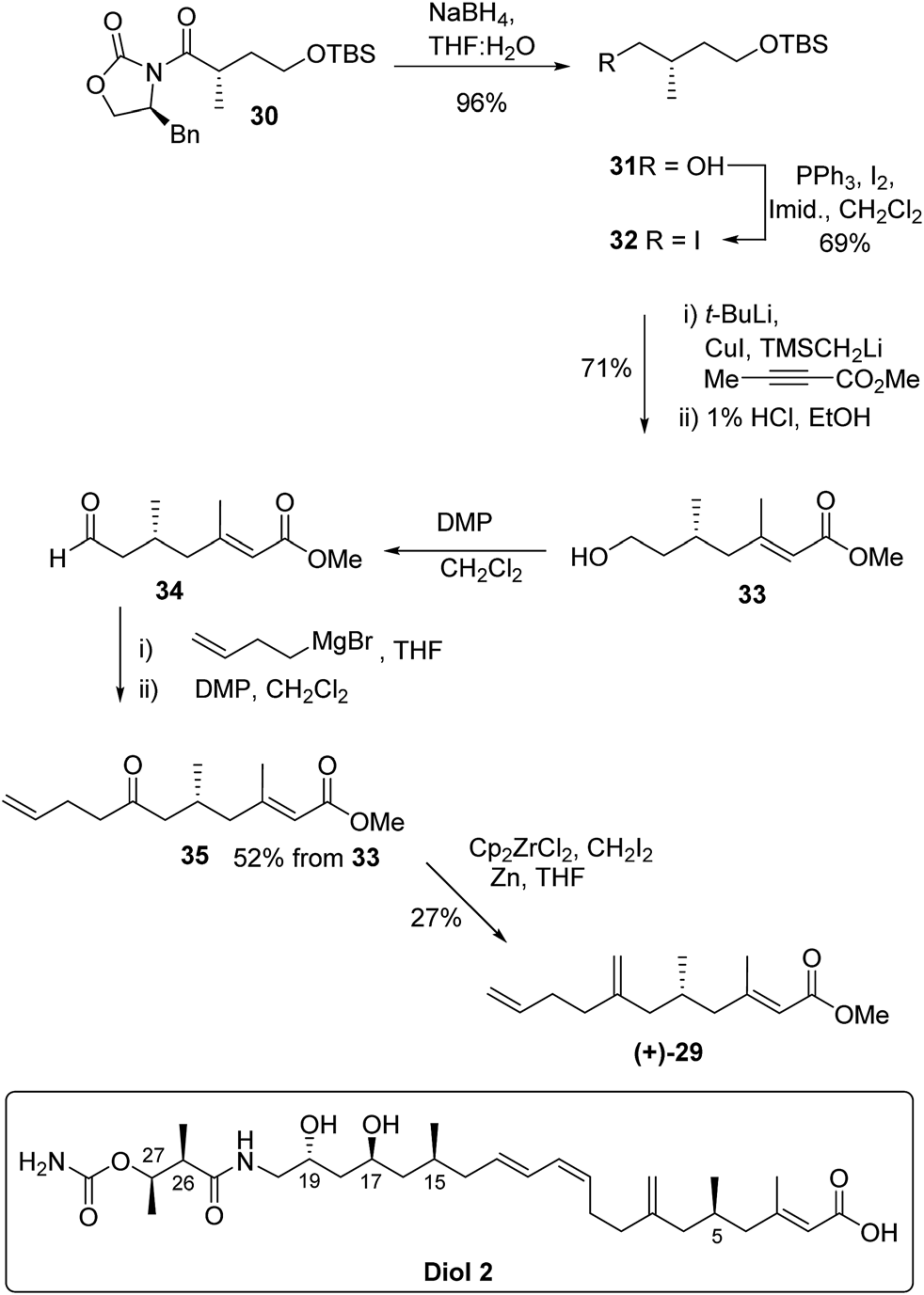
Enantioselective synthesis of ester **(+)-29** and final assignment of diol **2**.

The absolute configuration of **29** was confirmed *via* the enantioselective synthesis shown in Scheme 7. The stereocentre which would become C-5 in the target was readily generated *via* an alkylation reaction with NaHMDS/CH_3_I giving oxazolidinone **30** as previously described.^34^ Following reductive cleavage of the auxiliary, primary alcohol **31** was converted to iodide **32**.

The key synthetic step was formation of the tri-substituted unsaturated ester **33**. Preliminary studies using Horner–Wadsworth–Emmons or Julia chain extensions of a methyl ketone proved unsatisfactory giving a mixture of *E* and *Z*-alkenes. However, excellent control of alkene geometry was achieved *via* addition of the cuprate derived from iodide **32** to commercially available methyl but-2-ynoate. Following work up and deprotection, primary alcohol **33** was isolated in 71% yield from iodide **32** and the *E*-alkene geometry was confirmed by nOe.

Aldehyde **34**, prepared by Dess–Martin periodinane (DMP) oxidation^35^ of alcohol **33**, was coupled with but-3-enylmagnesium bromide and further oxidation gave ketone **35** in 52% yield over the 3 steps. In the final methylenation step it was important to avoid bond migration of the *exo* alkene to an *endo* position. This was achieved *via* the zirconium-mediated methylenation^36,37^ of the ketone. Unsaturated methyl ester **(+)-29** had identical NMR spectra to the degradation product **(–)-29** but with an optical rotation [*α*]_D_ +5.0 in accord with the synthetic material being the enantiomer of the degradation product. Thus kalimantacin A **1** has the 5*R* configuration.

## Conclusions

In conclusion, elucidating the absolute configuration of complex polyketides with multiple stereogenic centers is often an ambitious undertaking.^38,39^ Herein we used a multidisciplinary approach involving natural product isolation, chemical degradation studies, bioinformatics, NMR correlations and fragment synthesis to rigorously determine the absolute and relative stereochemistry of the kalimantacins isolated from *P. fluorescens*. Diol **2** from the Δ*batM* mutant has the 5*R*, 15*S*, 17*S*, 19*R*, 26*R*, 27*R* stereochemistry and is the immediate biosynthetic precursor of the bioactive kalimantacin A **1** formed by oxidation of the secondary alcohol to the 17-ketone. The absolute configurations of the 17, 19 and 27-hydroxyl groups in diol **2** are in good agreement with bioinformatic predictions.^12,13^

The ^13^C and ^1^H NMR data for natural product **1** isolated from *P. fluorescens* are entirely consistent with those reported^4,5^ for kalimantacin from *Alcaligenes* sp. YL-02632S in accord with both structures having the same relative stereochemistry. Furthermore it is evident from comparison of PKS/NRPS gene clusters for kalimantacin A from cultures of *P. fluorescens* ^12^ and batumin from *P. batumici* ^14^ that the natural products are indeed the same compound. Synergising chemical and biochemical methods have led to the elucidation of the structures of kalimantacin A **1** (and hence batumin) and diol **2**.

The inexorable rise of antibiotic resistant bacteria requires a multi-facetted response to meet this global threat to human health. In contrast to broad spectrum antibiotics, narrow spectrum agents can selectively decolonise specific pathogens (so called decolonisation drugs) in the human microbiota and are becoming increasingly important for detecting as well as fighting specific infections. They may also reduce the chance of emergence of antibiotic resistant strains.^11^ Kalimantacin has been identified as a highly specific agent against staphylococci that will potentially allow its deployment for the detection of resistant strains as well as a potent decolonisation agent. The determination of the absolute stereochemistry of kalimantacin as described in this paper will underpin further functional investigation of this important molecule.
