# Supplementary material for: Elucidation of the relative and absolute stereochemistry of the kalimantacin/batumin antibiotics
Source: Chem Sci. 2017 Jul 11;8(9):6196–201. doi: 10.1039/c7sc01670k (PMC5628338; doi:10.1039/c7sc01670k)

Electronic Supplementary Material (ESI) for Chemical Science.
This journal is © The Royal Society of Chemistry 2017

Elucidation of the Relative and Absolute Stereochemistry of the Kalimantacin/Batumin Antibiotics

Iain R. G. Thistlethwaite, Freya M. Bull, Chengsen Cui, Paul D. Walker, Shu-Shan Gao, Luoyi Wang, Zongshu Song, Joleen Masschelein, Rob Lavigne, Matthew P. Crump, Paul R. Race, Thomas J. Simpson and Christine L. Willis*

Chris.willis@bristol.ac.uk

Supporting Information

Contents

1. Comparison of NMR data for reduction products **4** and **5** with natural product diol **2** (Tables 1 and 2 and Figure S1).

2. Synthesis of fragment mimics **8**, **9** and **S5** (Schemes S1 and S2) and comparison of their ^{13}C- NMR data with natural product **3** (Tables 3 and 4, Figures S2 and S3).

3. Comparison of the NMR data of degradation product lactone **10** and the synthetic standard (Table 5).

4. Comparison of the NMR data of kalimantacin A from *Alcaligenes* sp. YL-02632S and *Pseudomonas fluorescens* BCCM_ID5359 (Tables 6 and 7).

5. Experimental procedures and data
   a) General experimental details.
   b) Culture conditions for growing *P. fluorescens* and isolation of **1**, **2** and **3**.
   c) Preparation of degradation product, unsaturated ester **(+)-29.**
   d) Selective reductions of 19-hydroxy-17-ketones and acetonide formation.
   e) Preparation of synthetic fragments **8**, **9** and **S5.**
   f) Oxidative cleavage of natural diol **2** and enantioselective synthesis of lactone **10.**
   g) NMR spectra of new products.

Position	δ$_H$ (ppm), multiplicity, *J* (Hz)		
	Natural Product **2**	1,3-*anti*-Diol **5**	1,3-*syn*-Diol **4**
2	5.68, d, 1.5	5.70, br s	5.69, br s
4	2.05, m	2.06, m	a: 2.08, m<br>b: 2.04, m
5	1.88, m	1.88, m	1.87, m
6	a: 2.10, m<br>b: 1.70, dd, 14.0, 9.5	a: 2.10, m<br>b: 1.70, m	a: 2.10, m<br>b: 1.73, m
8	2.06, m	2.07, m	2.07, m
9	a: 2.30, m<br>b: 2.20, m	a: 2.31, m<br>b: 2.21, m	a: 2.32, m<br>b: 2.21, m
10	5.27, dt, 11.0, 7.5	5.29, dt, 11.0, 7.5	5.30, dt, 11.0, 7.5
11	5.94, d, 11.0	5.96, t, 11.0	5.95, t, 11.0
12	6.23, dd, 15.0, 11.0	6.24, dd, 15.0, 11.0	6.24, ddd, 15.0, 11.0
13	5.65, dt, 15.0, 7.5	5.65, dt, 15.0, 7.5	5.65, dt, 15.0, 7.5
**14**	**a: 2.21, m<br>b: 1.85, m**	**a: 2.21, m<br>b: 1.85, m**	**a: 2.09, m<br>b: 1.97, m**
**15**	**1.69, m**	**1.70, m**	**1.73, m**
**16**	**a: 1.41, m<br>b: 1.34, m**	**a: 1.41, m<br>b: 1.34, m**	**a: 1.54, m<br>b: 1.16, m**
17	4.00, m	4.02, m	3.98, m
**18**	**a: 1.56, m<br>b: 1.48, m**	**a: 1.57, m<br>b: 1.50, m**	**1.55, m**
19	4.01, m	4.03, m	4.02, m
**20**	**a: 3.35, dd, 14.0, 3.5<br>b: 3.33, dd, 14.0, 7.5**	**3.34, m**	**a: 3.38, m<br>b: 3.31, m**
21	2.14, d, 1.0	2.15, s	2.15, d, 1.0
22	0.85, d, 6.5	0.85, d, 6.5	0.86, d, 6.5
23	a: 4.79, s<br>b: 4.72, s	a: 4.80, s<br>b: 4.73, s	a: 4.79, s<br>b: 4.73, s
24	0.89, d, 6.5	0.88, d, 6.5	0.91, 6.5
26	2.50, dq, 8.0, 7.0	2.51, dq, 8.0, 7.0	2.51, m
27	4.88, dq, 8.0, 6.5	4.89, dq, 8.0, 6.0	4.91, dq, 8.0, 6.5
28	1.26, d, 6.5	1.27, d, 6.5	1.28, d, 6.5
29	1.14, d, 7.0	1.15, d, 7.0	1.15, d, 7.0
NH	6.55, t, 5.5	6.72, t, 5.5	6.46, br s
NH2	5.43, br s	5.43, br s	5.42, br s

Table 1. Comparison of ^{1}H NMR (500 MHz, $CDCl_3$) data for diol **2** and selective reduction products, **4** and **5.**

Position	Natural Product **2**	1,3-*anti*-Diol **5**	1,3-*syn*-Diol **4**
1	169.7	167.7	169.5
2	116.8	117.0	116.8
3	160.8	160.6	160.3
4	49.1	49.1	49.1
5	28.9	28.9	29.7
6	42.9	42.7	43.1
7	147.3	147.4	147.4
8	35.6	35.6	35.7
9	26.4	26.5	26.7
10	129.4	129.4	129.4
11	129.1	129.1	129.1
12	127.0	126.9	127.1
13	133.2	133.2	133.0
14	39.6	39.5	41.0
15	30.3	30.3	29.9
16	44.9	45.0	45.2
**17**	**68.4**	**68.5**	**70.3**
18	41.2	41.3	40.8
**19**	**66.3**	**66.3**	**72.0**
20	45.8	45.9	45.6
21	19.2	19.2	19.2
22	20.0	20.1	20.0
23	112.1	112.2	112.0
24	20.2	20.3	19.7
**25**	**175.2**	**175.2**	**174.3**
26	47.2	47.3	47.1
27	73.4	73.4	73.7
28	18.1	18.1	18.1
29	13.9	14.0	13.9
30	157.5	157.5	157.6

Table 2 Comparison of ^{13}C NMR (125 MHz, $CDCl_3$) data for diol **2** and selective reduction products, **4** and **5.**

a

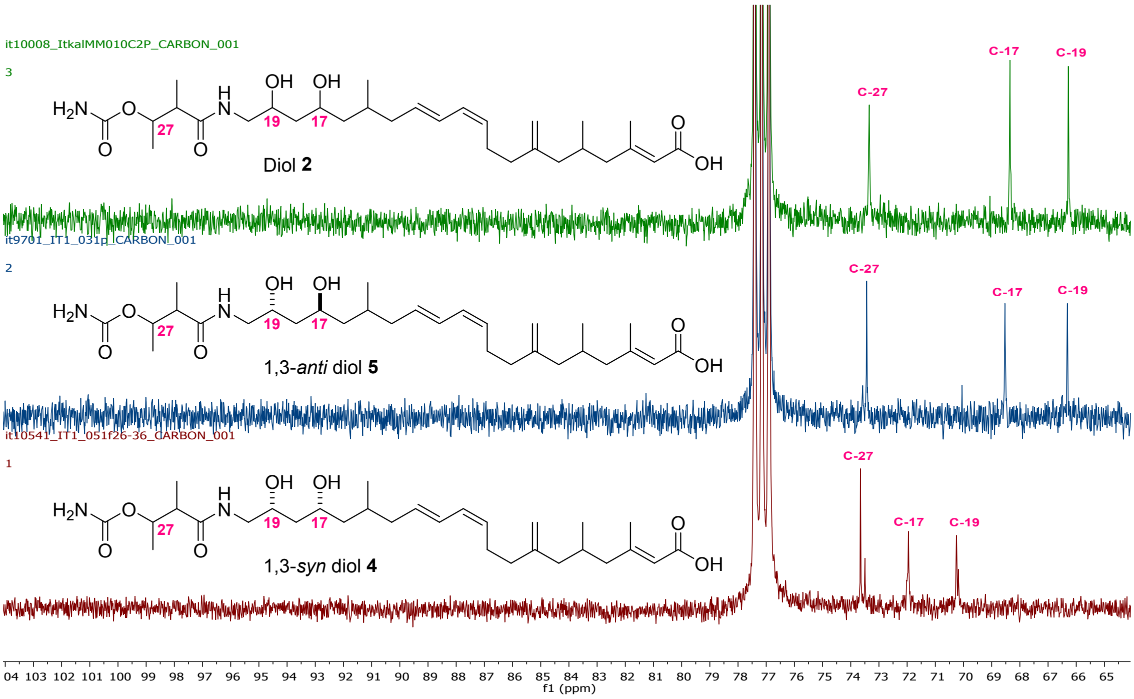

Figure S1 ^{13}C NMR comparison of natural diol **2**, 1,3-*anti*-diol **5** and 1,3-*syn*-diol **4.**

2. Synthesis of Fragment Mimics 8, 9 and S5.

(2*R*, 3*R*, 2'*R*)-Fragment mimic **8** was prepared as shown in Scheme S-1. The known[a] acid **S1** was coupled with allylamine to give amide **S2** in 77% yield. Oxidative cleavage of the alkene followed by Brown allylation using (+)-DIPCl gave alcohol **S3**, the stereochemistry of the hydroxyl group was confirmed by comparison of the (*R*)- and (*S*)-Mosher's ester derivatives. A palladium-mediated Wacker oxidation of alkene **S3** followed by deprotection of the silyl ether with 1% HCl in EtOH gave the required (2*R*, 3*R*, 2'*R*)-diol **8** (Scheme S1) for comparison with natural product **3** (Table 3 and 4 and Figures S2 and S3). A similar approach was used for the synthesis for the (2*R*, 3*R*, 2'*S*)-fragment mimic **S5** except that in this case (-)-DIPCl was used in the Brown allylation to generate the 2'S-alcohol **S4**.

(2*S*, 3*R*, 2'*R*)-Fragment mimic **9** was also synthesised by a similar strategy (Scheme S2) except in this case the precursor acid **S7** was prepared via an aldol reaction to give the known[b] syn product **S6**. Furthermore it was found that a higher yield (76%) was achieved in the deprotection of the silyl ether using AcOH, H_2O, THF compared with 1% HCl in EtOH which gave only a 20% yield.

a) P. Renuaud, D. Seebach, *Helv. Chim. Acta* **1986**, *69* 1704.
b) N. S. Trotter, S. Takahashi, T. Nakata*, Org. Lett.* **1999**, *1*, 957

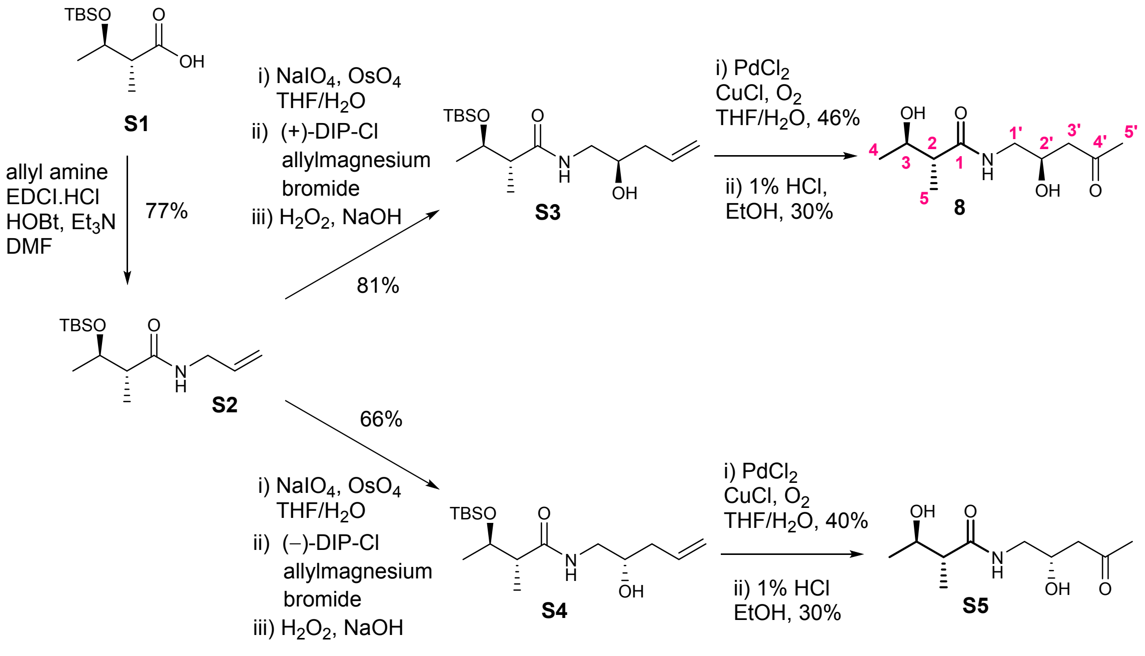

Scheme S1 Synthesis of (2*R*, 3*R*, 2’*R*)-fragment mimic **8** and (2*R*, 3*R*, 2’*S*)-fragment mimic **S5.**

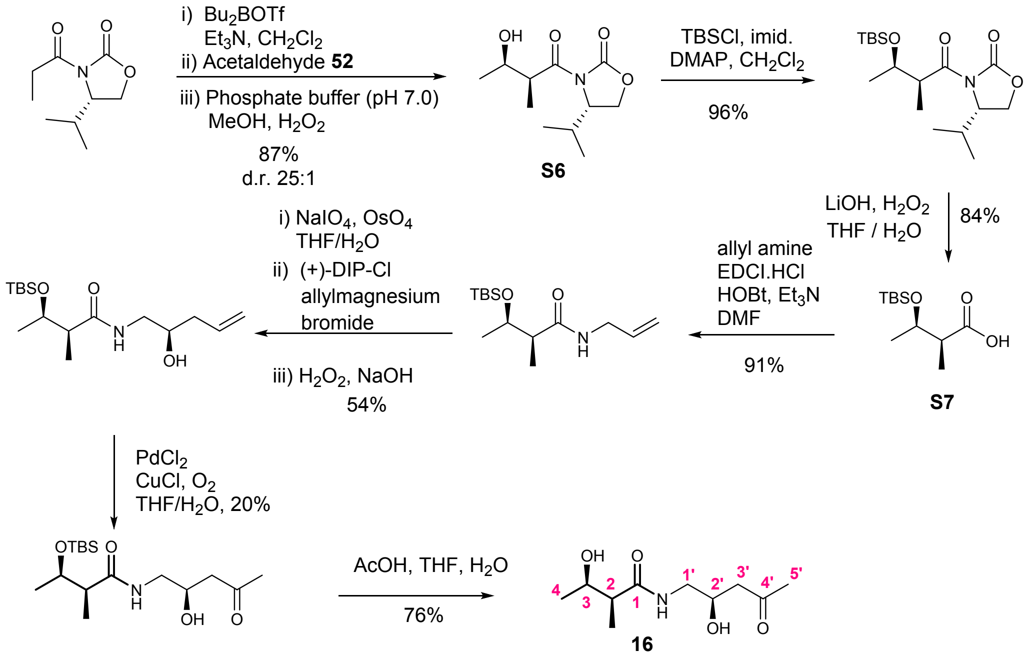

Scheme S2 Synthesis of (2*S*, 3*R*, 2’*R*)-fragment mimic **9.**

Comparison of the NMR data of the synthetic fragment mimics **8**, **9** and **S5** with the natural product **3** reveals that the best fit was with (2*R*, 3*R*, 2'*R*)-**8** (Table 3 and 4).

Position	δ_H (ppm), multiplicity, *J* (Hz)			
	Nat. Product **3**	(2*R*, 3*R*, 2'*R*)-**8**	(2*S*, 3*R*, 2'*R*)-**9**	(2*R*, 3*R*, 2'*S*)-**S5**
26/2-H	2.31, obscured m	2.31, quintet, 7.0	2.34, qd, 7.0, 5.5	2.30, quintet, 7.0
27/3-H	3.77, quintet, 6.5	3.76, sextet, 6.0	3.88, m	3.76, sextet, 6.0
28/4-H_3	1.15, d, 6.5	1.15, d, 6.5	1.09, d, 6.5	1.15, d, 6.5
29/5-H_3	1.11, d, 7.0	1.11, d, 7.0	1.10, d, 7.0	1.11, d, 7.0
20/1'-H_2	3.26, m	3.26, m	a: 3.31, ddd, 14.0, 5.5, 4.0<br>b: 3.19, dt, 14.0, 6.0	a: 3.33, ddd, 14.0, 6.0, 4.5<br>b: 3.18, ddd, 14.0, 6.5, 6.0

Table 3. Comparison of ^{1}H NMR (500 MHz, acetone-d_6) data for natural product **3** and fragment mimics **8, 9 and S5**

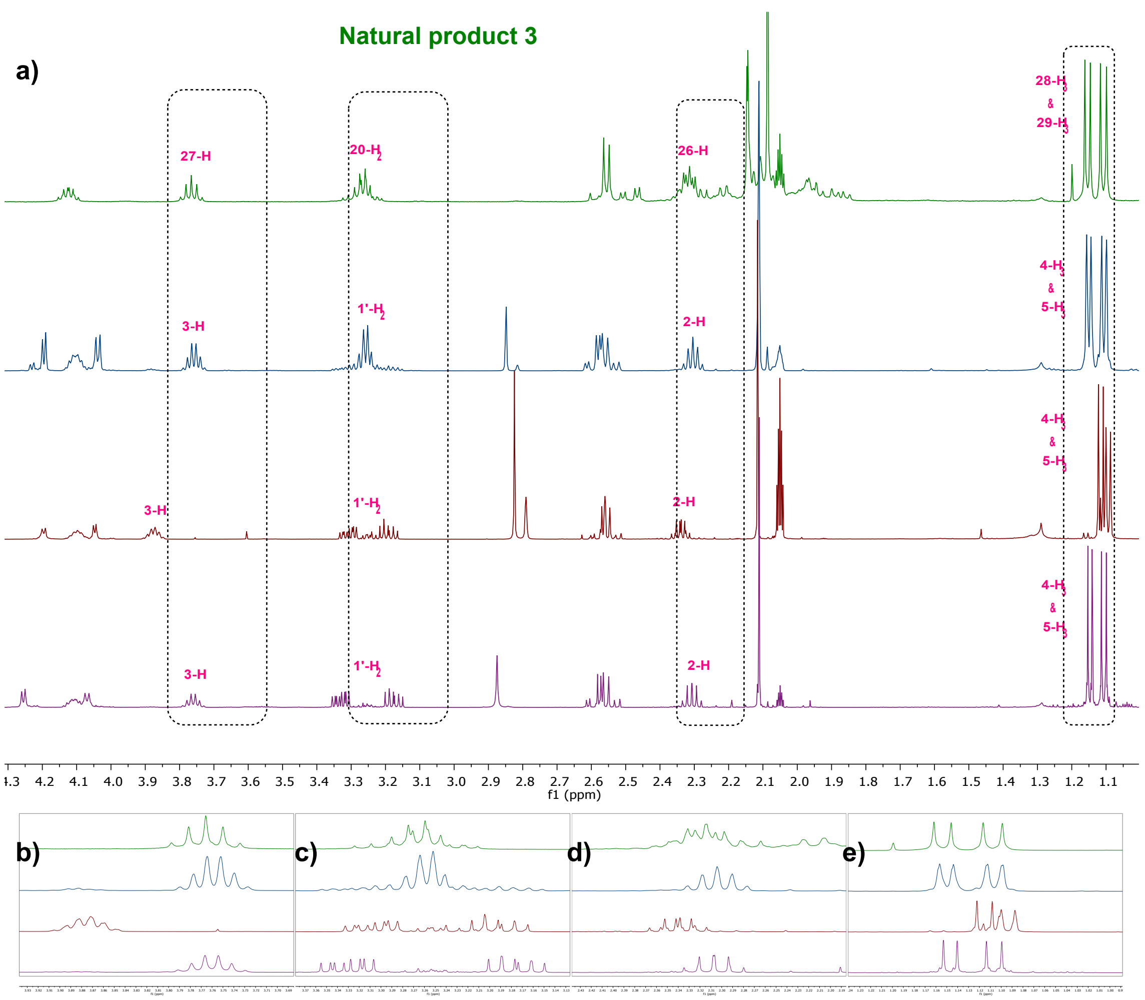

Figure S2 a) Stacked ^{1}H NMR spectra (500 MHz, acetone-d_6) of natural product **3** and synthetic fragments **8 (blue), 9 (red)** and **S5 (purple)** b) Expansion of resonances 27/3-H c) 20/1'-H_2 d) 26/2-H e) 28/4-H_3 and 29/5-H_3

Position	Nat. Product 3	(2*R*, 3*R*, 2'*R*)-8	(2*S*, 3*R*, 2'*R*)-9	(2*R*, 3*R*, 2'*S*)-S5
C-1 (25)	177.1	177.0	177.2	177.1
C-2 (26)	48.3	48.3	47.6	48.4
C-3 (27)	70.1	70.1	69.1	70.1
C-4 (28)	21.9	21.9	20.7	21.8
C-5 (29)	15.1	15.1	14.3	15.1
C-1' (20)	44.8	45.6	45.7	45.7
C-2' (19)	67.1	68.0	68.1	68.0
C-3' (18)	47.5	48.3	48.7	48.7

Table 4. Comparison of ^{13}C NMR (500 MHz, acetone-d_6) data for natural product **3** and fragment mimics **8**, **9** and **S-5**

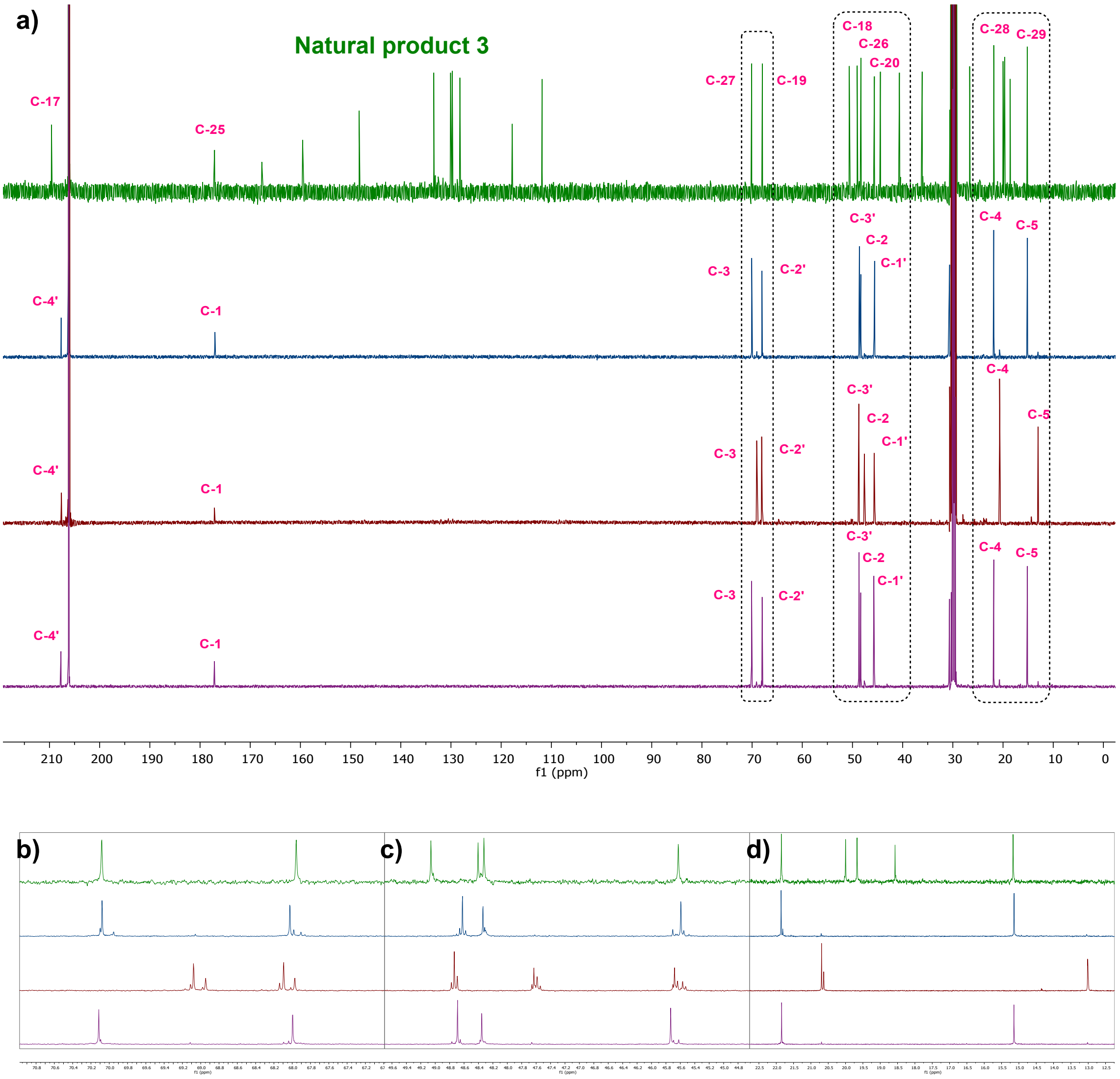

Figure S3. a) Stacked ^{13}C NMR spectra (125 MHz, acetone-d_6) of natural product **3** and fragments **8 (blue), 9 (red) and S5 (Purple)**; Expansion of resonances b) C-27/3 and C-19/2' c) C-18/3', C-26/2 and C-20/1' d) C-28/4 and C-29/5.

3. Comparison of synthetic lactone 10 with lactone (10) derived from the ozonolysis of diol 2.

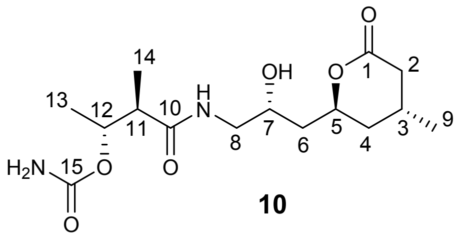

10

	^{1}H NMR (J in Hz) Isolated lactone	^{1}H NMR (J in Hz) Synthetic lactone **10**	^{13}C NMR Isolated lactone	^{13}C NMR Synthetic lactone **10**
1			175.3	175.3
2	a: 2.56, 16.0, 5.0<br>b: 2.25, dd, 16.0, 9.0	a: 2.55, dd, 16.0, 5.0<br>b: 2.23, dd, 16.0, 9.0	38.1	38.1
3	2.20, m	2.18, m	25.2	25.1
4	a: 1.84, ddd, 14.5, 9.0, 6.5<br>b: 1.67, ddd, 14.5, 5.5, 4.5	a: 1.82, m<br>b: 1.66, ddd, 14.0, 6.0, 4.5	36.4	36.4
5	4.73, m	4.72, dddd, 10.0, 9.0, 4.5, 2.5	75.8	75.8
6	a: 1.82, m<br>b: 1.56, m	a: 1.81, m<br>b: 1.54, ddd, 14.5, 10.5, 2.5	41.7	41.7
7	3.92, m	3.91, ddt, 10.5, 5.5, 2.5	67.6	67.6
8	a: 3.29, dd, 14.0, 6.0<br>b: 3.21, dd, 14.0, 5.5	a: 3.27, dd, 14.0, 6.0<br>b: 3.20, dd, 14.0, 5.5	46.6	46.6
9	1.11, d, 6.5	1.10, d, 6.5	21.5	21.5
10			177.0	177.0
11	2.53, app. quintet, 7.0	2.51, dq, 8.5, 7.0	47.5	47.5
12	4.82, dq, 8.0, 6.5	4.81, dq, 8.5, 6.5	73.6	73.6
13	1.25, d, 6.5	1.23, d, 6.5	18.0	18.0
14	1.13, d, 7.0	1.11, d, 7.0	14.1	14.1
15			159.1	159.1

Table 5 Comparison of ^{1}H NMR (500 MHz, methanol-d_4) and ^{13}C NMR (125 MHz in methanol-d_4) of lactone **10** formed via ozonolysis of natural diol **2** and synthetic lactone.

4. Comparison of the NMR data of kalimantacin A from *Alcaligenes* sp. YL-02632S and *Pseudomonas fluorescens* BCCM_ID5359.

	δ_H (ppm), multiplicity, *J* (Hz)	
Position	*Pseudomonas fluorescens* BCCM_ID9359	*Alcaligenes* sp. YL-02632S
1		
2	5.69 (d, *J* 1.0)	5.69 (s)
3		
4	2.06 (m)	2.10 (m), 2.01 (m)
5	1.89 (m)	1.89 (m)
6	2.10 (m), 1.71 (dd, *J* 13.5, 9.0)	2.08 (m), 1.75 (dd, *J* 13.5, 8.6)
7		
8	2.07 (m)	2.07 (m)
9	2.30 (m), 2.23 (m)	2.30 (m), 2.24 (m)
10	5.31 (dt, *J* 11.0, 7.5)	5.31 (dt, *J* 11.0, 7.3)
11	5.95 (t, *J* 11.0)	5.95 (t, *J* 11.0)
12	6.23 (dd, *J* 15.0, 11.0)	6.25 (dd, *J* 15.3, 11.0)
13	5.59 (dt, *J* 15.0, 7.0)	5.58 (dt, *J* 15.3, 7.3)
14	2.08 (m), 1.95 (m)	2.07 (m), 1.98 (m)
15	2.09 (m)	2.08 (m)
16	2.43 (m), 2.27 (m)	2.44 (dd, *J* 16.5, 5.0), 2.27 (m)
17		
18	2.58-2.54 (m)	2.57 (m)
19	4.17 (m)	4.17 (m)
20	3.39 (dt, *J* 14.0, 6.5), 3.32 (ddd, *J* 14.0, 5.5, 3.5)	3.35 (m)
21	2.15 (d, *J* 1.0)	2.15 (s)
22	0.86 (d, *J* 6.5)	0.86 (d, *J* 6.7)
23	4.79 (s), 4.73 (s)	4.80 (s), 4.74 (s)
24	0.89 (d, *J* 6.5)	0.89 (d, *J* 6.7)
25		
26	2.47 (m)	2.49 (m)
27	4.90 (dq, *J* 8.0, 6.5)	4.90 (m)
28	1.28 (d, *J* 6.5)	1.28 (d, *J* 6.1)
29	1.15 (d, *J* 7.0)	1.15 (d, *J* 7.5)
30		
NH_2	6.39 (br s)	5.37 (br)
NH	6.36 (t, *J* 6.0)	6.56 (br)

Table 6. Comparison of ^{1}H NMR (500 MHz, $CDCl_3$) data for kalimantacin A from *Alcaligenes* sp. YL-02633S and kal/bat from *Pseudomonas fluorescens* BCCM_ID9359.

Position	δ_C (ppm)	
	Pseudomonas fluorescens BCCM_ID9359	*Alcaligenes* sp. YL-02632S
1	169.6	169.6
2	116.8	116.7
3	160.8	160.7
4	49.1	48.9
5	29	28.9
6	43.1	43.2
7	147.2	147.1
8	35.6	35.4
9	26.3	26.1
10	129.8	129.7
11	128.9	128.7
12	127.6	127.4
13	132.4	132.2
14	40.1	40.0
15	29.4	29.2
16	50.4	50.3
17	211.0	211.5
18	46.9	46.9
19	67.4	67.2
20	44.3	44.2
21	19.1	18.9
22	20.0	19.7
23	112	111.8
24	19.8	19.6
25	174.7	174.6
26	47.0	46.8
27	73.6	73.4
28	18.2	17.9
29	13.8	13.7
30	157.4	157.2

Table 7. Comparison of ^{13}C NMR (125 MHz, $CDCl_3$) data for kalimantacin A from *Alcaligenes* sp. YL-02633S and kal/bat from *Pseudomonas fluorescens* BCCM_ID9359.

5. Experimental procedures and data

5a) General experimental details

Unless otherwise stated, all reagents were purchased from commercial suppliers and used without further purification. Anhydrous solvents were used which were dried using the Anhydrous Engineering Ltd. double alumina and alumina-copper catalysed drying columns. All moisture or air sensitive reactions were carried out in flame dried glassware under a positive pressure of N_2 using standard syringe/septa techniques. Flash column chromatography was performed on silica gel (Merck Kieselgel 60, 230-400 mesh). Thin layer chromatography was carried out on Polygram 0.2 mm silica gel TLC plates visualising with 254 nm UV light and developing with either a $KMnO_{4,}$ phosphomolybdic aicd or Vanillin dip, where appropriate.

Optical rotations were determined with the sodium D line (λ= 589 nm) using a Perkin Elmer 241 MC polarimeter. $[\alpha]_D^{22}$ values are quoted in units 10^{-1} deg cm^2 g^{-1}. Infrared (IR) spectroscopy was recorded on a Perkin Elmer Spectrum 100 FT-IR spectrometer with an ATR diamond cell irradiating between 4000 cm^{-1} and 600 cm^{-1}. Melting points were determined using an electrothermal melting point apparatus and are uncorrected. Electron impact (EI) and chemical ionisation (CI) mass spectra were recorded on a VG Analytical Autospec mass spectrometer. Methane was the ionization gas used for CI. Electrospray ionisation (ESI) mass spectra were recorded on a Bruker Daltonics micrOTOFII mass spectrometer.

NMR spectra were recorded using either a Varian VNMRS operating at 500 or 400 MHz or a JEOL ECP 400 MHz spectrometer. Chemical shifts (δ_H) are quoted in parts per million (ppm), *J* values are given in Hz and referenced to the appropriate residual solvent peak. Data reported as follows: chemical shift, integration, multiplicity (s = singlet, br s = broad singlet, d = doublet, t = triplet, q= quartet, qi = quintet, sx = sextet, hept = heptet, m = multiplet, dd = doublet of doublet, etc.), coupling constants, assignment. Chemical shifts (δ_C) are quoted in parts per million (ppm), referenced to the appropriate residual solvent peak. DEPT^{135}, COSY and HMQC were used where necessary in assigning NMR spectra.

5b) Culture conditions for growing *P. fluorescens* and isolation of 1, 2 and 3.

Method A:

Single colony preparation

Pseudomonas fluorescens (*P. fluorescens*) strain BCCM_ID9359 (or mutant strain, Δ*batM* and Δ*batF*) were grown at 28 °C on tryptose-agar (Merck) for 24 h.

Seed culture preparation

Single colonies were used to inoculate tryptose-broth (Merck) (50 mL in a 250 mL conical flasks) and incubated at 28 °C, 200 rpm for 24 h to prepare the seed culture.

Fermentation culture

Secondary stage medium (tryptose-broth supplemented with sucrose (10.3 g L^{-1}) and glycine (94.0 mg L^{-1})) in 10 × 500 mL conical flasks were inoculated with seed culture (5% *v*/*v*) and grown at 16 °C, 200 rpm for 48 h.

Extraction and purification

The culture was adjusted to pH 10 with NaOH solution (3 M) and the cells removed by centrifugation at 43,000 *g* for 20 min. The supernatant was acidified to pH 3 with HCOOH and then extracted with EtOAc (1 vol) twice. The combined organic extractions were dried ($MgSO_4$), filtered and concentrated *in vacuo* to give the crude extract as a dark brown oil. An aliquot of the crude extract was filtered through cotton and then analysed by LCMS. Purification was performed by silica chromatography to give ~80% pure material. Further purification on reverse phase (C18) silica (50% MeOH in H_2O with 0.1% HCOOH to 100% MeOH with 0.1% HCOOH) gave the desired compounds kalimantacin A and mutant analogues **2-3** in >95% purity.

Method B:

Single colony preparation

P. fluorescens BCCM_ID9359 (or mutant strain, Δ*batM* and Δ*batF*) was incubated on a tryptose-agar (Merck) at 30 ºC for 24 hours.

Seed culture preparation

90 mL of modified L-medium and 10 mL 40% *w*/*v* glucose solution in a 500 mL flask was inoculated with a single colony and incubated at 200 rpm at 25 ºC. (Modified L-medium: bacto tryptone (10 g), yeast extract (5 g), sodium chloride (5 g), and deionised water added up to 900 mL).

Fermentation culture

90 mL of fermentation media was decanted into 500 ml flasks with vigorous stirring. After sterilization, 5 ml of seed culture, 10 ml sterilized 40% *w*/*v* glucose solution, were added. The culture was inoculated at 22 ºC at 250 rpm for 48 hours. (Fermentation medium is modified L-

medium: bacto tryptone (10 g), yeast extract (5 g), sodium chloride (5 g), and deionised water added up to 900 mL).

Extraction and purification

The cells were separated from the media by centrifugation (8000 rpm for 15 min). The media was extracted with EtOAc (0.5 *v/v*) 3 times. The cell pellet was lysed with acetone 3 times, the acetone was removed *in vacuo*, and the residue extracted with EtOAc (0.5 v/v) 3 times. The combined EtOAc extracts were evaporated in *vacuo* to give a crude extract. An aliquot of crude extract was filtered with cotton and then analysed by LCMS and HPLC. Purification was performed by silica column chromatography or by HPLC chromatography.

Kalimantacin A 1

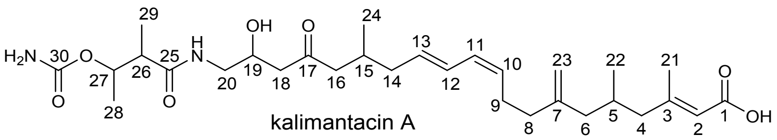

Kalimantacin A was grown and purified according to method A or method B, to give kalimantacin A as a viscous brown oil (Method A: 34 mg/L) and (Method B: 50 mg/L). $[\alpha]_D^{22}$ −20.0 (*c* 1.0, MeOH); υ_{max} (neat) / cm^{-1} 3338 (OH and NH), 2958 (CH), 2925 (CH), 1697 (C=O), 1641 (C=O), 1377, 1060; δ_H (500 MHz, $CDCl_3$) 6.39 (2H, br s, NH_2), 6.36 (1H, t, *J* 6.0, NH), 6.23 (1H, dd, *J* 15.0, 11.0, 12-H), 5.95 (1H, t, *J* 11.0, 11-H), 5.69 (1H, d, *J* 1.0, 2-H), 5.59 (1H, dt, *J* 15.0, 7.0, 13-H), 5.31 (1H, dt, *J* 11.0, 7.5, 10-H), 4.90 (1H, dq, *J* 8.0, 6.5, 27-H), 4.79 (1H, s, 23-*H*H), 4.73 (1H, s, 23-H*H*), 4.17 (1H, m, 19-H), 3.39 (1H, dt, *J* 14.0, 6.5, 20-*H*H), 3.32 (1H, ddd, *J* 14.0, 5.5, 3.5, 20-H*H*), 2.58-2.54 (2H, m, 18-H_2), 2.47 (1H, m, 26-H), 2.43 (1H, m, 16-*H*H), 2.30 (1H, m, 9-*H*H), 2.27 (1H, m, 16-H*H*), 2.23 (1H, m, 9-H*H*), 2.15 (1H, d, *J* 1.0, 21-H_3), 2.10 (1H, *overlapping* m, 6-*H*H), 2.09 (1H, *overlapping* m, 15-H), 2.08 (1H, *overlapping* m, 14-*H*H), 2.07 (2H, *overlapping* m, 8-H_2), 2.06 (2H, *overlapping* m, 4-H_2), 1.95 (1H, m, 14-H*H*), 1.89 (1H, m, 5-H), 1.71 (1H, dd, *J* 13.5, 9.0, 6-H*H*), 1.28 (3H, d, *J* 6.5, 28-H_3), 1.15 (3H, d, *J* 7.0, 29-H_3), 0.89 (3H, d, *J* 6.5, 24-H_3), 0.86 (3H, d, *J* 6.5, 22-H_3); δ_C (125 MHz, $CDCl_3$) 211.0 (C-17), 174.7 (C-25), 169.6 (C-1), 160.8 (C-3), 157.4 (C-30), 147.2 (C-7), 132.4 (C-13), 129.8 (C-10), 128.9 (C-11), 127.6 (C-12), 116.8 (C-2), 112.0 (C-23), 73.6 (C-27), 67.4 (C-19), 50.4 (C-16), 49.1 (C-4), 47.0 (C-26), 46.9 (C-18), 44.3 (C-20), 43.1 (C-6), 40.1 (C-14), 35.6 (C-8), 29.4 (C-15), 29.0 (C-5), 26.3 (C-9), 20.0 (C-22), 19.8 (C-24), 19.1 (C-21), 18.2 (C-28), 13.8 (C-29); *m/z* (ESI+) 571.34 $[M+Na]^+$; Found (ESI) 571.3354 ($C_{30}H_{48}N_2O_7Na$ requires 571.3356).

Data in accord with the literature: W. Mattheus, L. J. Gao, P. Herdewijn, B. Landuyt, J. Verhaegen, J. Masschelein, G. Volckaert, R. Lavigne, *Chem. Biol.* **2010**, *17*, 149–159.

Diol 2

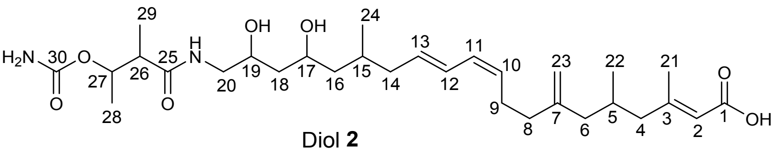

Diol **2** was isolated from *multi-mutant strain* (Δ*batM*) according to method A (23 mg/L) or method B (25 mg/L). $[\alpha]_D^{22}$ −10.4 (*c* 0.48, MeOH); υ_{max} (neat) / cm^{-1} 3345 (OH and NH), 2925 (CH), 2856 (CH), 1706 (C=O), 1648 (C=O), 1379, 1063; δ_H (500 MHz, $CDCl_3$) 6.82 (1H, m, NH), 6.23 (1H, dd, *J* 15.0, 11.0, 12-H), 5.94 (1H, d, *J* 11.0, 11-H), 5.68 (1H, d, *J* 1.5, 2-H), 5.65 (1H, dt, *J* 15.0, 7.5, 13-H), 5.48 (2H, br s, NH_2), 5.27 (1H, dt, *J* 11.0, 7.5, 10-H), 4.88 (1H, dq, *J* 8.0, 6.5, 27-H), 4.79 (1H, s, 23-*H*H), 4.72 (1H, s, 23-H*H*), 4.01 (1H, m, 19-H), 4.00 (1H, m, 17-H), 3.35 (1H, dd, *J* 14.0, 3.5, 20-*H*H), 3.33 (1H, dd, *J* 14.0, 7.5, 20-H*H*), 2.50 (1H, dq, *J* 8.0, 7.0, 26-H), 2.30 (1H, m, 9-*H*H), 2.21 (1H, *overlapping* m, 14-*H*H), 2.20 (1H, *overlapping* m, 9-H*H*), 2.14 (3H, d, *J* 1.0, 21-H_3), 2.10 (1H, *overlapping* m, 6-*H*H), 2.06 (2H, *overlapping* m, 8-CH_2), 2.05 (2H, *overlapping* m, 4-H_2), 1.88 (1H, m, 5-H), 1.84 (1H, m, 14-H*H*), 1.70 (1H, m, 15-H), 1.67 (1H, dd, *J* 14.0, 9.5, 6-H*H*), 1.56 (1H, m, 18-*H*H), 1.48 (1H, m, 18-H*H*), 1.41 (1H, m, 16-*H*H), 1.34 (1H, m, 16-H*H*), 1.26 (3H, d, *J* 6.5, 28-H_3), 1.14 (3H, d, *J* 7.0, 29-H_3), 0.89 (3H, d, *J* 6.5, 24-H_3), 0.85 (3H, d, *J* 6.5, 22-H_3); δ_C (125 MHz, $CDCl_3$) 175.2 (C-25), 169.7 (C-1), 160.8 (C-3), 157.5 (C-30), 147.3 (C-7), 133.2 (C-13), 129.4 (C-10), 129.1 (C-11), 127.0 (C-12), 116.8 (C-2), 112.1 (C-23), 73.4 (C-27), 68.4 (C-17), 66.3 (C-19), 49.1 (C-4), 47.2 (C-26), 45.8 (C-20), 44.9 (C-16), 42.9 (C-6), 41.2 (C-18), 39.6 (C-14), 35.6 (C-8), 30.3 (C-15), 28.9 (C-5), 26.4 (C-9), 20.2 (C-24), 20.0 (C-22), 19.2 (C-21), 18.1 (C-28), 13.9 (C-29); *m/z* (ESI) 573.35 $[M+Na]^+$; Found (ESI) 573.3507 ($C_{30}H_{50}O_7N_2Na$ requires 573.3510).

Data in accord with the literature: W. Mattheus, L. J. Gao, P. Herdewijn, B. Landuyt, J. Verhaegen, J. Masschelein, G. Volckaert, R. Lavigne, *Chem. Biol.* **2010**, *17*, 149–159.

Descarbamoyl diol 3

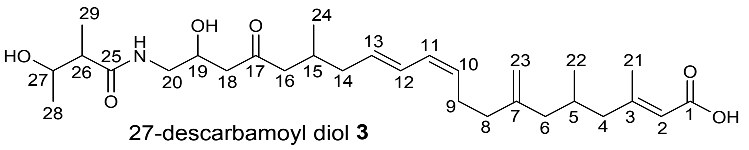

27-descarbamoyl diol **3**

27-Descarbamoyl diol **3** was isolated from *mutant strain* Δ*batF* according to method B (30 mg/L). $[\alpha]_D^{22}$ −15.6 (*c* 0.64, MeOH); υ_{max} (neat) / cm^{-1} 3351 (OH and NH), 2926 (CH), 1706 (C=O), 1642 (C=O), 1545, 1379, 1229; δ_H (500 MHz, acetone-d_6), 7.30 (1H, br s, 20-NH), 6.35 (1H, dd, *J* 15.0, 11.0, 12-H), 5.97 (1H, t, *J* 11.0, 11-H), 5.69 (1H, s, 2-H), 5.65 (1H, dt, *J* 15.0, 6.5, 13-H), 5.32 (1H, dt, *J* 11.0, 7.0, 10-H), 4.82 (1H, s, 23-*H*H), 4.76 (1H, s, 23-H*H*), 4.12 (1H, m, 19-H), 3.77 (1H, m, 27-H), 3.26 (2H, m, 20-H), 2.55 (2H, d, *J* 6.5, 18-H_2), 2.49 (1H, dd, *J* 16.5, 5.0, 16-H), 2.33 (2H, *overlapping* m, 9-H_2), 2.31 (1H, *overlapping* m, 26-H), 2.29 (1H, *overlapping* m, 16-H), 2.21 (1H, m, 4-H), 2.15 (3H, d, *J* 1.5, 21-H_3), 2.10 (2H, *overlapping* m, 8-H_2), 2.09 (1H, *overlapping* m, 6-*H*H), 2.08 (1H, *overlapping* m, 15-H), 2.07 (1H, *overlapping* m, 14-*H*H), 2.00 (1H, *overlapping* m, 14-H*H*), 1.97 (1H, *overlapping* m, 5-H), 1.94 (1H, *overlapping* m, 4-H), 1.87 (1H, m, 6-H*H*), 1.15 (3H, d, *J* 7.0, 28-H_3), 1.11 (3H, d, *J* 6.5, 29-H_3), 0.88 (3H, d, *J* 7.0, 24-H_3), 0.85 (3H, d, *J* 6.5, 22-H_3); δ_C (125 MHz, acetone-d_6), 208.5 (C-17), 176.0 (C-25), 166.9 (C-1), 158.6 (C-3), 147.4 (C-7), 132.5 (C-13), 129.2 (C-10), 128.8 (C-11), 127.3 (C-12), 116.9 (C-2), 111.0 (C-23), 69.2 (C-27), 67.1 (C-19), 49.7 (C-16), 48.3 (C-4), 47.5 (C-18), 47.4 (C-26), 44.8 (C-20), 43.5 (C-6), 39.7 (C-14), 35.1 (C-8), 29.3 (C-15), 28.7 (C-5), 25.7 (C-9), 20.9 (C-28), 19.1 (C-24), 18.7 (C-22), 17.6 (C-21), 14.2 (C-29); δ_H (500 MHz, $CDCl_3$) 6.65 (1H, t, *J* 6.0, NH), 6.25 (1H, dd, *J* 15.0, 11.0, 12-H), 5.94 (1H, t, *J* 11.0, 11-H), 5.68 (1H, d, *J* 1.0, 2-H), 5.58 (1H, dt, *J* 15.0, 7.5, 13-H), 5.33 (1H, dt, *J* 11.0, 7.5, 10-H),

4.80 (1H, s, 23-*H*H), 4.74 (1H, s, 23-H*H*), 4.15 (1H, m, 19-H), 3.85 (1H, dq, *J* 10.0, 6.5, 27-H), 3.41-3.28 (2H, m, 20-H_2), 2.67-2.52 (2H, m, 18-H_2), 2.44 (1H, dd, *J* 16.5, 5.5, 16-*H*H), 2.30 (1H, *overlapping* m, 9-*H*H), 2.25 (1H, *overlapping* m, 26-H), 2.24 (1H, *overlapping* m, 9-H*H*), 2.23 (1H, *overlapping* m, 16-H*H*), 2.14 (3H, d, *J* 1.5, 21-H_3), 2.13 (1H, *overlapping* m, 4-*H*H), 2.10 (1H, *overlapping* m, 15-H), 2.06 (2H, *overlapping* m, 8-H_2), 2.05 (1H, *overlapping* m, 6-*H*H), 2.02 (2H, *overlapping* m, 14-H_2), 1.97 (1H, *overlapping* m, 4-H*H*), 1.89 (1H, m, 5-H), 1.78 (1H, dd, *J* 14.0, 8.5, 6-H*H*), 1.23 (3H, d, *J* 6.5, 28-H_3), 1.16 (3H, d, *J* 7.0, 29-H_3), 0.90 (3H, d, *J* 6.5, 24-H_3), 0.85 (3H, d, *J* 6.5, 22-H_3); δ_C (125 MHz, $CDCl_3$) 211.7 (C-17), 177.1 (C-25), 169.5 (C-1), 161.3 (C-3), 147.3 (C-7), 132.2 (C-13), 129.9 (C-10), 128.8 (C-11), 127.7 (C-12), 116.6 (C-2), 111.8 (C-23), 70.0 (C-27), 67.3 (C-19), 50.4 (C-16), 49.0 (C-4), 48.7 (C-26), 46.9 (C-18), 44.3 (C-20), 43.6 (C-6), 40.2 (C-14), 35.6 (C-8), 29.6 (C-15), 29.1 (C-5), 26.3 (C-9), 21.4 (C-28), 19.9 (C-24), 19.8 (C-22), 19.2 (C-21), 15.2 (C-29); *m/z* (ESI) 528.3304 $[M+Na]^+$; Found 528.3304 ($C_{29}H_{47}NO_6Na$ requires 528.3296).

Data in accord with the literature: W. Mattheus, L. J. Gao, P. Herdewijn, B. Landuyt, J. Verhaegen, J. Masschelein, G. Volckaert, R. Lavigne, *Chem. Biol.* **2010**, *17*, 149–159.

5c) Preparation of degradation product, unsaturated ester 29

Methyl Ester S8

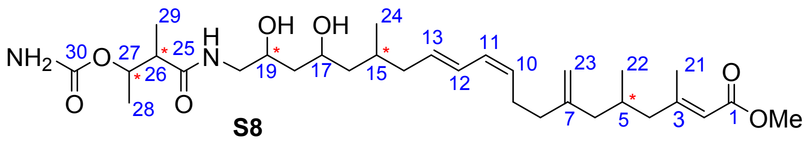

S8

To diol **2** (83 mg, 0.15 mmol), dissolved in DCM (0.24 mL) and MeOH (0.06 mL), was added trimethylsilyldiazomethane (2.0 M in Et_2O, 0.09 mL, 0.18 mmol) at 0 °C in an open topped vial. The reaction was allowed to warm to RT over 1 h, before being cooled back down to 0 °C. The reaction was then diluted with DCM (0.5 mL) and quenched with AcOH (2 drops). The solvent was removed under a stream of nitrogen, furnishing methylated diol **S8,** with an assumed 100% yield, which required no further purification; δ_H (400 MHz, $CDCl_3$) 6.86 (1H, s, NH), 6.25 (1H, dd, *J* 14.5, 11.5, 12-H), 5.98 (1H, d, *J* 11.0, 11-H), 5.73 – 5.49 (4H, m, 2-H, 13-H, NH_2), 5.28 (1H, s, 10-H), 4.86 (1H, app. pent., *J* 6.0, 27-H), 4.79 (1H, s, 23-*H*H), 4.72 (1H, s, 23-H*H*), 4.12 – 3.87 (2H, m, 17-H, 19-H), 3.67 (3H, s, OCH_3), 3.41 – 3.18 (2H, m, 20-H_2), 2.49 (1H, app. pent., *J* 7.0, 26-H), 2.34 – 2.23 (2H, m, 9-H_2), 2.24 – 2.15 (2H, m, 4-*H*H, 14-*H*H), 2.12 (3H, s, 21-H_3), 2.05 – 1.95 (3H, obscured m, 8-H_2, 6-*H*H), 1.95 – 1.74 (4H, m, 4-H*H*, 5-H, 6-H*H*, 14-H*H*), 1.72 – 1.63 (1H, m, 15-H), 1.62 – 1.53 (1H, m, 18-*H*H), 1.48 (1H, m, 18-H*H*), 1.44 – 1.32 (2H, m, 16-H_2), 1.25 (3H, d, *J* 6.0, 28-H_3), 1.13 (3H, d, *J* 7.0, 29-H_3), 0.88 (3H, d, *J* 6.5, 24-H_3), 0.81 (3H, d, *J* 5.5, 22-H_3); δ_C (125 MHz, $CDCl_3$) 175.2 (C-25), 167.3 (C-1), 159.6 (C-3), 157.5 (C-30), 147.3 (C-7), 133.1 (C-13), 129.4 (C-10), 128.9 (C-11), 127.2 (C-12), 116.6 (C-2), 111.4 (C-23), 73.3 (C-27), 68.4 (C-17), 66.9 (C-19), 51.0 (OCH_3), 48.7 (C-4), 47.1 (C-26), 45.9 (C-20), 44.7 (C-16), 44.3 (C-6), 40.9 (C-18), 40.2 (C-14), 35.6 (C-8), 30.2 (C-15), 29.1 (C-5), 26.0 (C-9), 20.2 (C-24), 19.5 (C-22), 18.8 (C-21), 18.1 (C-28), 14.0 (C-29). HRMS (ESI) 565.3833 $[M+H]^+$ ($C_{31}H_{53}N_2O_7$ requires 565.3847).

Methyl (*E*)-3,5-dimethyl-7-methyleneundeca-2,10-dienoate (-)-29

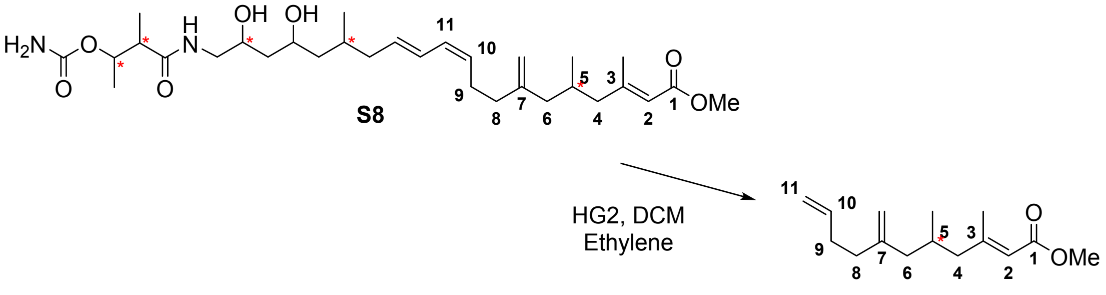

(-)-29

Methyl ester **S8** (85 mg, 0.15 mmol) was dissolved in DCM (26 mL). Hoveyda-Grubbs catalyst 2nd generation (39 mg, 61.8 µmol) was then added, before ethylene was bubbled through the solution for 20 min. Light was the excluded and the reaction, kept under an atmosphere of ethylene, was stirred at room temperature for 48 h. TLC analysis showed that the reaction was incomplete; therefore further Hoveyda-Grubbs catalyst 2nd generation (39 mg, 61.8 µmol) was added to the reaction mixture. Light was again excluded, the atmosphere of ethylene replaced before the reaction was left stirring for a further 72 h. The solvent was removed under reduced pressure and flash chromatography (1% Et_2O in P.E.) yielded ester **(-)-29** as a colourless oil (6.9 mg, 19%); $[\alpha]_D$ -6.8 (*c* 0.29, $CHCl_3$); IR v_{max} (neat)/cm^{-1} 2955, 2923, 2853, 1727, 1461, 1379, 1151; δ_H (500 MHz, $CDCl_3$) 5.83 (1H, ddt, *J* 17.0, 10.0, 6.5, 10-H), 5.67 (1H, s, 2-H), 5.04 (1H, ddt, *J* 17.0, 2.0, 1.5, 11-H*H*), 4.98 (1H, ddt, *J* 10.0, 2.0, 1.0, 11-H*H*), 4.81 (1H, m, 7-C*H*H), 4.75 (1H, m, 7-CH*H*), 3.70 (3H, s, OC*H*$_3$), 2.27 – 2.17 (3H, m, 9-H_2, 4-H*H*), 2.15 (3H, d, *J* 1.5, 3-CH_3), 2.13 – 2.05 (2H, m, 8-H_2), 2.02 (1H, dd, *J* 13.0, 5.5, 6-HH), 1.96 – 1.81 (3H, m, 5-H, 4-H*H*, 6-H*H*), 0.87 – 0.77 (3H, d, *J* 6.5, 5-CH_3); δ_c (125 MHz, $CDCl_3$) 167.2 (C-1), 159.5 (C-3), 147.3 (C-7), 138.5 (C-10), 116.7 (C-2), 114.8 (C-11), 111.3 (CH_2-7), 50.9 (OMe), 48.7 (C-4), 44.3 (C-6), 35.1 (C-8), 32.1 (C-9), 29.1 (C-5), 19.6 (5-CH_3), 18.8 (3-CH_3). Found (ESI) 259.1673 ($C_{15}H_{24}O_2Na$ requires 259.1668).

4-(*tert*-Butyldimethylsilyloxy)butanoic acid S9

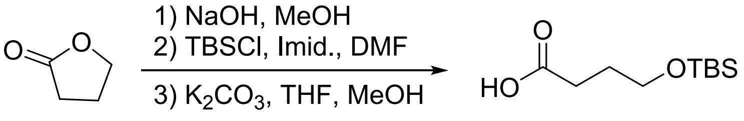

S9

γ-Butyrolactone (3.00 g, 34.9 mmol) was dissolved in MeOH (35 mL) before NaOH (1.53 g, 38.3 mmol) was added at room temperature. After stirring for 3 h, the solvent was removed *in vacuo.* The resulting white solid was then suspended in DMF (70 mL) before being cooled to 0 °C. Imidazole (7.83 g, 115 mmol) and TBSCl (11.6 g, 76.7 mmol) were added, the reaction mixture was allowed to warm to room temperature stirred overnight. H_2O (100 mL) was added, the layers were separated and the aqueous layer extracted with hexane (3 x 100 mL). The combine organic layers were dried over $MgSO_4$ and concentrated *in vacuo.* The crude di-protected product was then dissolved in MeOH (35 mL) and THF (35 mL) followed by the addition of K_2CO_3 (9.63 g, 69.7 mmol in 116 mL H_2O). The reaction was stirred overnight then acidified to pH 2 with 1M HCl (ca. 60 mL) at 0 °C. The mixture was extracted with Et_2O (4 x 150 mL) and the combined organic layers dried over Na_2SO_4, filtered and concentrated *in vacuo*, giving carboxylic acid **S9** (8.15 g, quant.), with no further purification, as a colourlesss oil; δ_H (400 MHz, $CDCl_3$) 3.68 (2H, t, *J* 6.0, 4-H_2), 2.48 (2H, t, *J* 7.0, 2-H_2), 1.93 – 1.77 (2H, m, 3-H_2), 0.90 (9H, s, SiC(C*H*$_3$)$_3$), 0.06 (6H, s, Si(C*H*$_3$)$_2$); δ_C (100 MHz, $CDCl_3$) 179.3 (C-1), 62.3 (C-4), 31.0 (C-2), 27.7 (C-3), 26.0 (SiC(*C*H$_3$)$_3$), 18.4 (Si*C*(CH$_3$)$_3$), -5.3 (Si(*C*H$_3$)$_2$).

R.-G. Ren, C.-M. Li. Z.-Y. Mao, B.-G. Wei, *Tetrahedron Lett.* **2014**, *55*, 6903.

(*S*)-4-Benzyl-3-(4'-(*tert*-butyldimethylsilyloxy)butanoyl)oxazolidin-2-one S10

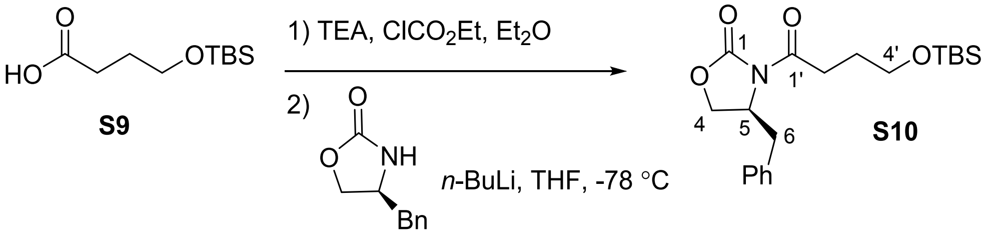

To a solution of carboxylic acid **S9** (3.80 g, 17.4 mmol) in Et_2O (170 mL) was added triethyamine (2.79 mL, 20.0 mmol) dropwise, under nitrogen. The solution was stirred for 15 min, cooled to 0 °C, before ethyl chloroformate (1.67 mL, 17.4 mmol) was added. The reaction mixture was warmed to room temperature and stirred for 1 h. During this time, in a separate flask, *n*-BuLi (1.61 M, 2.0 mmol) was added dropwise, at -78 °C and under N_2, to a solution of (*4S*)-benzyloxazolidione (3.09 g, 17.4 mmol) in THF (20 mL). The oxazolidinone solution was then added dropwise to the carboxylic acid at -78 °C. The reaction mixture was stirred at -78 °C for 1 h, then at room temperature for 3 h. The reaction was quenched with sat. $NH_4Cl_{(aq.)}$ (40 mL), H_2O (50 mL) was added and the layers separated. The aqueous layer was extracted with Et_2O (3 x 100 mL), the combined organic layers were washed with brine (100 mL), dried over $MgSO_4$ and concentrated *in vacuo*. Flash chromatography (10% EtOAc in P.E.) yielded **S10** as a colourless oil (4.88 g, 74%); $[\alpha]_D^{21}$ +33.68 (*c* 0.95, $CHCl_3$) [Lit. $[\alpha]_D^{21}$ +32.32 (*c* 0.95, $CHCl_3$)]; δ_H (400 MHz, $CDCl_3$) 7.37-7.26 (3H, m, ArH), 7.24-7.18 (2H, m, ArH), 4.66 (1H, m, 5-H), 4.27 – 4.08 (2H, m, 4-H_2), 3.71 (1H, t, *J* 6.5, 4'-H_2), 3.29 (1H, dd, *J* 13.5, 3.5, 6-H*H*), 3.01 (2H, app. dt, *J* 7.0, 1.0 Hz, 2'-H_2), 2.78 (1H, dd, *J* 13.5, 9.5, 6-*H*H), 1.97 – 1.85 (2H, m, 3'-H_2), 0.90 (9H, s, SiC(C*H*$_3$)$_3$), 0.06 (6H, s, Si(CH$_3$)$_2$).
R. Bajpai, F. Yang, D. P. Curran, *Tetrahedron Lett.* **2007**, *48*, 7965

(*S*)-4-Benzyl-3-((*S*)-4-(*tert*-butyldimethylsilyloxy)-2-methylbutanoyl)oxazolidin-2-one 30

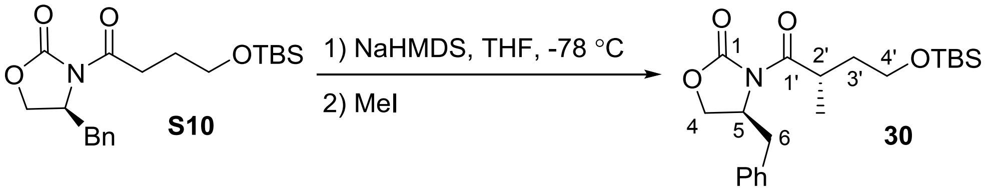

NaHMDS (2.0 M in THF, 8.83 mL, 17.6 mmol) was added dropwise to **S10** (4.76 g, 12.6 mmol) in THF (30 mL) at -78 °C under a nitrogen atmosphere. The reaction was stirred for 1 h at -78 °C, before MeI (3.93 mL, 63.0 mmol) was added and stirred for a further 3 h. The reaction was quenched with acetic acid (0.84 mL) before being warmed to room temperature. The mixture was then diluted with EtOAc (30 mL) and washed with water (30 mL). The aqueous layer was then extracted with EtOAc (3 x 20 mL), the combined organic layers were washed with brine (40 mL), dried over $MgSO_4$ and concentrated *in vacuo*. Flash chromatography (10% EtOAc in P.E.) yielded **30** as a white solid (3.46 g, 70%); m.p. 51-53 °C, Lit. 51-52 °C; [Lit. $[\alpha]_D^{21}$ +59.83 (*c* 0.43, $CHCl_3$)]; δ_H (400 MHz, $CDCl_3$) 7.36-7.27 (3H, m, ArH), 7.23-7.19 (2H, m, ArH), 4.66 (1H, m, 5-H), 4.20-4.13 (2H, m, 4-H_2), 3.98-3.77 (1H, m, 2'-H), 3.76 – 3.57 (2H, m, 4'-H_2), 3.26 (1H, dd, *J* 13.5, 3.0, 6-*H*H), 2.77 (1H, dd, 13.5, 9.5, 6-H*H*), 2.13 -1.93 (1H, m, 3'-*H*H), 1.65 (1H, m, 3'-H*H*), 1.26 (3H, d, *J* 4.5, 2'-CH_3), 0.87 (9H, s, $SiC(CH_3)_3$), 0.03 (6H, s, $Si(CH_3)_2$).

R. Bajpai, F. Yang, D. P. Curran, *Tetrahedron Lett.* **2007**, *48*, 7965

(*S*)-4-(*tert*-Butyldimethylsilyloxy)-2-methylbutan-1-ol 31

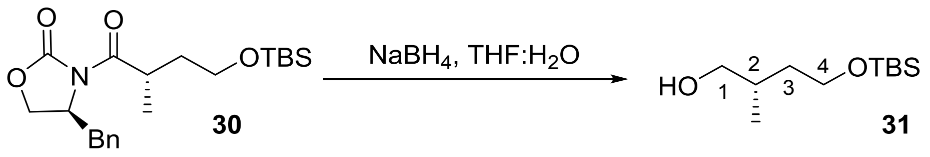

Alkylated product **30** (3.59 g, 9.17 mmol) was dissolved in THF/H_2O (3:1, 230 mL) before the addition of $NaBH_4$ (1.73 g, 45.8 mmol) at room temperature. The reaction was stirred overnight before being quenched with NH_4Cl (100 mL) and the phases separated. The aqueous layer was extracted with EtOAc (3 x 50 mL), the combined organic layers were washed with brine (100 mL), dried over $MgSO_4$ and concentrated *in vacuo*. Flash chromatography (10% EtOAc in P.E.) yielded **31** as a colourless oil (1.93 g, 96%); $[\alpha]_D^{21}$ -7.0 (*c* 1.0, $CHCl_3$) [Lit. $[\alpha]_D^{21}$ -10.0 (*c* 1.0, $CHCl_3$)]; IR v_{max} (neat)/cm^{-1} 3355, 2954, 2928, 2857, 1253, 1091; δ_H (400 MHz, $CDCl_3$) 3.76 (1H m, 4-H*H*), 3.66 (1H, m, 4-*H*H), 3.52 (1H, dd, *J* 11.0, 4.5, 1-*H*H), 3.41 (1H, dd, *J* 11.0, 7.0, 1-H*H*), 1.82 (1H, m, 2-H), 1.54 (2H, app. q, *J* 6.0, 3-H_2), 0.92 (9H, s, $SiC(CH_3)_3$), 0.07 (6H, s, $Si(CH_3)_2$); δ_C (100 MHz, $CDCl_3$) 68.3 (C-4), 61.9 (C-1), 37.6 (C-3), 34.6 (C-2), 26.0 ($SiC(CH_3)_3$), 18.4 ($SiC(CH_3)_3$), 17.5 (2-CH_3), -5.3 ($Si(CH_3)_2$). HRMS (ESI) 219.1775 [M+H] ($C_{11}H_{27}NO_2$ requires 219.1775).
Y. Matseuda, S. Xu, E.-I Negishi, *Tetrahedron Lett.* **2015**, *56*, 3346.

(*S*)-4-(*tert*-Butyldimethylsilyloxy)-1-iodo-2-methylbutane 32

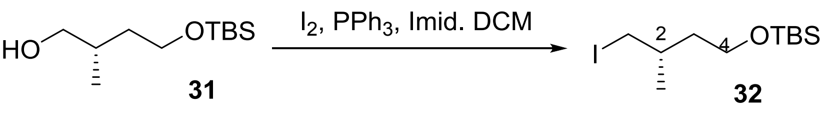

Iodine (0.25 g, 0.97 mmol) was added to a solution of PPh_3 (0.25 g, 0.97 mmol) and imidazole (0.09 g, 1.38 mmol) in DCM (1.5 mL) under a nitrogen atmosphere. A solution of alcohol **31** (0.10 g, 0.46 mmol) in DCM (3.5 mL) was then added and the reaction was stirred overnight at room temperature. The reaction was quenched with sat. $Na_2S_2O_{3(aq)}$ (10 mL) and extracted with DCM (3 x 5 mL), the combined organic layers were dried over $MgSO_4$ and concentrated *in vacuo*. Flash chromatography (1% Et_2O in P.E.) yielded **32** as a colourless oil (103 mg, 69%); $[\alpha]_D^{21}$ +5.0 (*c* 1.0, $CHCl_3$) [Lit. $[\alpha]_D^{21}$ +5.0 (*c* 1.0, $CHCl_3$)]; IR ν_{max} (neat)/cm^{-1} 2955, 2928, 2586, 1254, 1096, 831, 773; δ_H (400 MHz, $CDCl_3$) 3.64 (2H, m, 4-H_2), 3.28 (1H, dd, *J* 9.5, 4.5, 1-*H*H), 3.20 (1H, dd, *J* 9.5, 5.5, 1-H*H*), 1.71 – 1.56 (2H, m, 3-H_2), 1.43 (1H, m, 2-H), 0.99 (3H, d, *J* 6.5, 2-CH_3), 0.89 (9H, s, SiC(CH_3)$_3$), 0.05 (6H, s, Si(CH_3)$_2$); δ_C (100 MHz, $CDCl_3$) 60.8 (C-4), 39.3 (C-3), 31.5 (C-2), 26.1 (SiC(*C*H_3)$_3$), 20.8 (2-CH_3), 18.4 (SiC(*C*H_3)$_3$), 18.3 (C-1), -5.2 (Si(*C*H_3)$_2$).

Y. Matseuda, S. Xu, E.-I Negishi, *Tetrahedron Lett*. **2015**, *56*, 3346.

Methyl (*R*,*E*)-7-hydroxy-3,5-dimethylhept-2-enoate 33

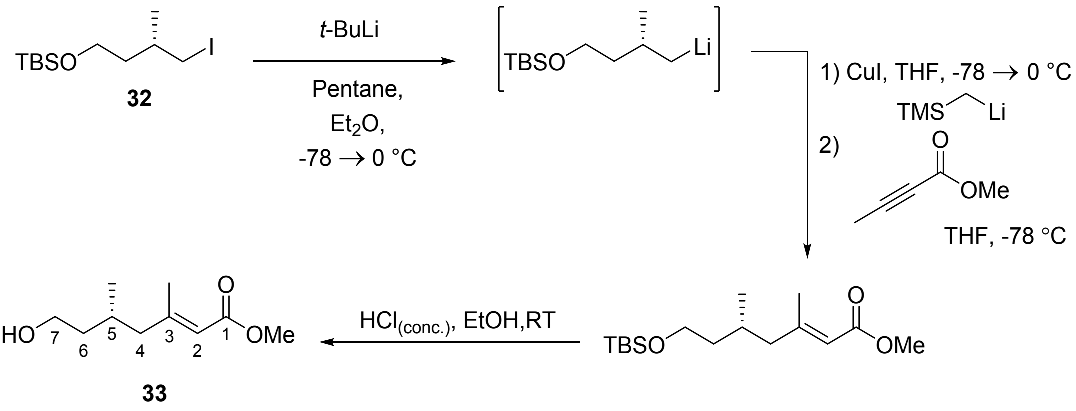

To alkyl iodide **32** (1.13 g, 3.44 mmol) in pentane (9 mL) and ether (15 mL) was added *t*-BuLi (1.6 M, 4.73 mL, 7.57 mol) at -78 °C under a nitrogen atmosphere. After stirring for 15 min, the reaction was warmed to 0 °C before THF (0.56 mL) was added. The reaction was further stirred for 15 min, cooled to -40 °C, then added dropwise to a solution of trimethylsilylmethylcopper at -78 °C. [Trimethylsilylmethylcopper was prepared as follows: To a suspension of purified CuI (0.66 g, 3.44 mmol) in THF (30 mL), was added trimethylsilylmethyllithium (1.0 M in pentane, 3.44 mL, 3.44 mmol), at -78 °C. The solution was then warmed to 10 °C, and stirred for 10 min]. After the addition of the alkyl lithium to the cuprate, the solution was warmed to 0 °C, and after stirring for 10 min a homogenous dark grey solution formed. The solution was then cooled back down to -78 °C, and solution of methyl 2-butynoate (0.34 mL, 3.44 mL) in THF (4 mL) was added. After stirring for 2 h the reaction was quenched with sat. aq. NH_4Cl (10 mL) and left to warm to room temperature over 1 h. Diethyl ether (20 mL) and water (10mL) were added and the phases seprated. The organic phases were washed with water (2 x 10 mL) and brine (10 mL), before

being dried over $MgSO_4$ and concentrated *in vacuo* to give an oil which was used directly in the next step.

Note: If diethyl ether is used as the solvent, instead of THF, for the formation of the mixed cuprate and subsequent addition, a mixture of E:Z (5:1) diastereomers is observed.

The product from above was dissolved in ethanol (70 mL) and conc. HCl (10 drops) was added. The reaction was left to stir at room temperature overnight before water (30 mL) and EtOAc (30 mL) were added. The phases was separated and the aqueous phase was extracted with EtOAc (2 x 30 mL), the combined organic layers were then dried over $MgSO_4$ and concentrated *in vacuo*. Flash chromatography (20% EtOAc in P.E.) yielded **33** as a colourless oil (0.45 g, 71% over three steps); $[\alpha]_D^{21}$ +5.0 (*c* 1.0, $CHCl_3$); IR ν_{max} (neat)/cm^{-1} 3399, 2952, 2978, 1716, 1644, 1435, 1223, 1149; δ_H (400 MHz, $CDCl_3$) 5.65 (1H, *J* 1.0, 2-H), 3.75   3.62 (2H, m, 7-H_2), 3.67 (3H, s, OCH_3), 2.22 – 2.14 (1H, m, 4-H*H*), 2.13 (3H, d, *J* 1.0, 3-CH_3), 2.01 – 1.83 (2H, m, 4-H*H*, 5-H), 1.65 – 1.53 (1H, m, 6-H*H*), 1.36 (1H, m, 6-*H*H), 0.87 (3H, d, *J* 6.5, 5-CH_3); δ_C (100 MHz, $CDCl_3$) 167.2 (C-1), 159.1 (C-3), 116.8 (C-2), 60.9 (C-7), 50.9 (OCH_3), 49.1 (C-4), 39.7 (C-6), 27.7 (C-5), 19.5 (5-CH_3), 18.7 (3-CH_3); HRMS (ESI) 209.1146 $[M+Na]^+$ ($C_{10}H_{18}NaO_3$ requires 209.1148).

Methyl (*R*,*E*)-3,5-dimethyl-7-oxoundeca-2,10-dienoate 35

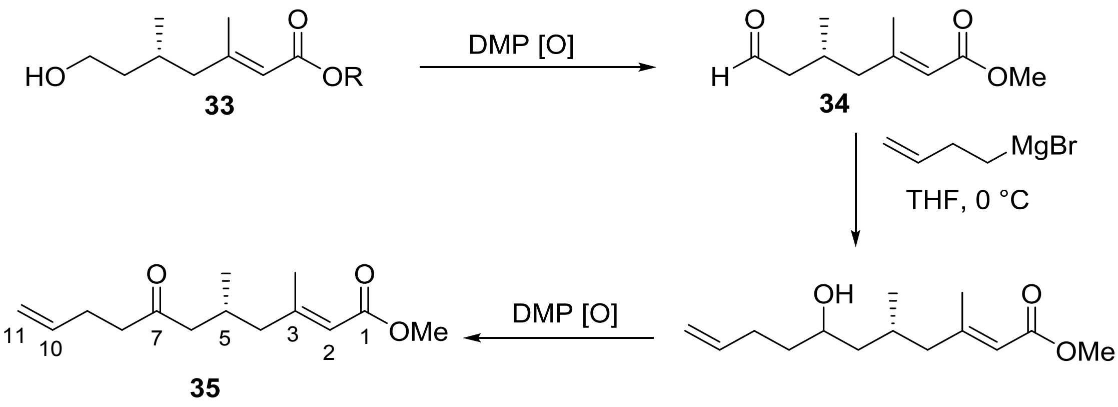

Alcohol **33** (0.30 g, 1.61 mmol) was dissolved in DCM (16 mL). DMP (0.35 M in DCM, 9.20 mL, 3.20 mmol) was then added and the reaction stirred at room temperature for 2 h, before being quenched with oa 1:1 solution of sat. $Na_2S_2O_{3(aq)}$ and sat. $NaHCO_{3(aq)}$ (10 mL). The phases were separated and the aqueous phase extracted with DCM (3 X 10 mL), the combined organic layers were dried over $MgSO_4$ and concentrated *in vacuo*. The resulting aldehyde **34** was taken on to the next step without further purification.

But-3-en-1-ylmagnesium bromide (1.0 M in THF, 1.85 mL, 1.85 mmol) was added dropwise to the crude aldehyde **34** dissolved in THF (8 mL) at 0 °C. The reaction was left to warm to room temperature over 1 h. The reaction was then quenched with sat. aq. NH_4Cl (5 mL), the phases separated and the aqueous phase extracted with Et_2O (3 x 10 mL). The combined organic layers were dried over $MgSO_4$ and concentrated *in vacuo* and the resulting alcohol was taken on to the next step without further purification. Alcohol (0.30 g, 1.61 mmol) was dissolved in DCM (16 mL). DMP (0.35 M in DCM, 9.20 mL, 3.2 mmol) was then added and the reaction stirred at room

temperature for 2 h, before being quenched with a 1:1 solution of sat. $Na_2S_2O_{3(aq)}$ and sat. $NaHCO_{3(aq)}$ (10 mL). The phases were separated and the aqueous phase extracted with DCM (3 X 10 mL). The combined organic layers were dried over $MgSO_4$ and concentrated *in vacuo*. Flash chromatography (10% EtOAc in P.E.) yielded ketone **35** as a colourless oil (0.197 g, 52% over three steps); $[\alpha]_D^{21}$ +6.0 (*c* 1.0, $CHCl_3$); IR ν_{max} (neat)/cm^{-1} 2952, 1713, 1643, 1435, 1223, 1149; δ_H (400 MHz, $CDCl_3$) 5.78 (1H, m, 10-H), 5.64 (1H, m, 2-H), 5.02 (1H, d, J 17.0, 11-H*H*), 4.97 (1H, d, J 10.5, 11-*H*H), 3.68 (3H, s, OCH_3), 2.47 (2H, t, *J* 7.5, 8-H_2), 2.41 – 2.18 (5H, m, 5-H, 6-H_2, 9-H_2), 2.14 (3H, s, 3-CH_3), 2.13 – 2.07 (1H, m, 4-H*H*), 1.95 (1H, dd, *J* 13.0, 7.5, 4-*H*H), 0.87 (3H, d, *J* 6.0, 5-CH_3); δ_C (100 MHz, $CDCl_3$) 209.4 (C-7), 167.0 (C-1), 158.6 (C-3), 137.2 (C-10), 117.1 (C-2), 115.4 (C-11), 51.0 (OCH_3), 49.6 (C-6), 48.5 (C-4), 42.6 (C-8), 27.8 (C-9), 27.1 (C-5), 20.00 (5-*C*H_3), 18.7 (3-*C*H_3); HRMS (ESI) 261.1453 $[M+Na]^+$ ($C_{14}H_{22}NaO_3$ requires 261.1461).

Methyl (*S*,*E*)-3,5-dimethyl-7-methyleneundeca-2,10-dienoate (+)-29

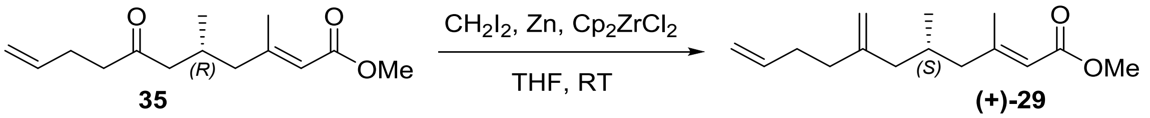

Ketone **35** (0.17 g, 0.73 mmol) was dissolved in THF (3 mL) under a nitrogen atmosphere. Zinc dust (0.38 g, 5.85 mmol) and zirconocene dichloride (0.37 g, 1.28 mmol) were then added, followed by diiodomethane (0.13 mL, 1.61 mmol). The reaction was stirred for 24 h at room temperature, before being quenched with sat. aq. NH_4Cl (5 mL) and extracted with EtOAc (3 x 10 mL). Purification by flash chromatography (2% Et_2O in P.E.) yielded alkene **(+)-29** (47 mg, 27%) as a yellow oil. The spectral data were in good accordance with those reported for degradation product **(-)-29**; $[\alpha]_D^{21}$ +5.0 (*c* 1.0, $CHCl_3$).

5d) Selective reductions of β-keto esters and acetonide formation

Synthesis of 1,3-*anti*-diol 5

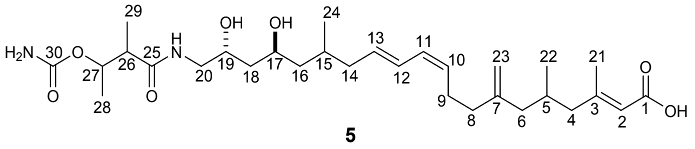

Glacial acetic acid (2 mL) was added to a solution of tetramethylammonium triacetoxyborohydride[a] (108 mg, 0.41 mmol) in dry acetonitrile (2 mL) and the mixture stirred at room temperature under an atmosphere of nitrogen for 0.5 h. The borohydride solution was then added, by cannula, to a solution of kalimantacin A **1** (28 mg, 0.051 mmol) in dry acetonitrile (1 mL) at −20 °C and stirred for 5 h. The reaction was quenched with Rochelle's salt (0.5 M, 5 mL) and allowed to warm to room temperature whilst vigorously stirring for 3 h. The reaction mixture was extracted with CH_2Cl_2 (4 × 20 mL). The combined organic layers were washed with brine (20 mL), dried ($MgSO_4$), filtered and concentrated *in vacuo*. The crude material was purified by silica column chromatography (1% to 10% MeOH in CH_2Cl_2 with 0.5% CH_3COOH) to

give 1,3-*anti*-diol **5** as a yellow oil (21 mg, 75%). $[\alpha]_D^{22}$ −10.0 (*c* 0.4, MeOH); υ_{max} (neat) / cm^{-1} 3344 (OH and NH), 2924 (CH), 2854 (CH), 1706 (C=O), 1647 (C=O), 1379, 1060; δ_H (500 MHz, $CDCl_3$) 6.72 (1H, t, *J* 5.5, NH), 6.24 (1H, dd, *J* 15.0, 11.0, 12-H), 5.96 (1H, t, *J* 11.0, 11-H), 5.70 (1H, br. s, 2-H), 5.67 (1H, m, 13-H), 5.43 (2H, s, NH_2), 5.29 (1H, dt, *J* 11.0, 7.5, 10-H), 4.89 (1H, dq, *J* 8.0, 6.0, 27-H), 4.80 (1H, s, 23-*H*H), 4.73 (1H, s, 23-H*H*), 4.03 (2H, m, 17-H, 19-H), 3.34 (2H, m, 20-H_2), 2.51 (1H, dq, *J* 8.0, 7.0, 26-H), 2.31 (1H, m, 9-*H*H), 2.25 (1H, m, 14-*H*H), 2.21 (1H, *overlapping* m, 9-H*H*), 2.15 (3H, s, 21-H_3), 2.12 (1H, *overlapping* m, 6-*H*H), 2.07 (2H, *overlapping* m, 8-H_2), 2.06 (2H, *overlapping* m, 4-H_2), 1.90 (1H, *overlapping* m, 5-H), 1.85 (1H, m, 14-H*H*), 1.72 (1H, m, 15-H), 1.70 (1H, m, 6-H*H*), 1.57 (1H, m, 18-*H*H), 1.50 (1H, m, 18-H*H*), 1.43 (1H, m, 16-*H*H), 1.34 (1H, m, 16-H*H*), 1.28 (3H, d, *J* 6.0, 28-H_3), 1.15 (3H, d, *J* 7.0, 29-H_3), 0.92 (3H, d, *J* 7.0, 22-H_3), 0.88 (3H, d, *J* 6.5, 24-H_3); δ_C (125 MHz, $CDCl_3$) 175.2 (C-25), 169.7 (C-1), 160.6 (C-3), 157.5 (C-30), 147.4 (C-7), 133.2 (C-13), 129.4 (C-10), 129.1 (C-11), 126.9 (C-12), 117.0 (C-2), 112.2 (C-23), 73.4 (C-27), 68.5 (C-17), 66.3 (C-19), 49.1 (C-4), 47.3 (C-26), 45.9 (C-20), 45.0 (C-16), 42.7 (C-6), 41.3 (C-18), 39.5 (C-14), 35.6 (C-8), 30.3 (C-15), 28.9 (C-5), 26.5 (C-9), 20.3 (C-24), 20.1 (C-22), 19.2 (C-21), 18.1 (C-28), 14.0 (C-29); *m/z* (ESI) 573.35 $[M+Na]^+$; Found (ESI) 573.3495 ($C_{30}H_{50}O_7N_2Na$ requires 573.3510).

a) D. A. Evans, K. T. Chapman, E. M. Carreira, *J. Am. Chem. Soc.*, **1988**, *110*, 3560.

Synthesis of 1,3-*syn*-diol 4

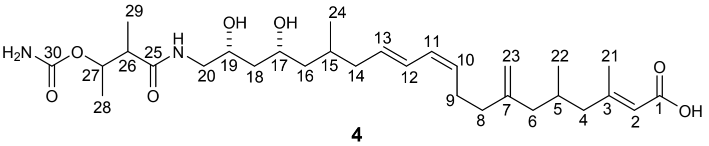

4

Kalimantacin A **1** (10 mg, 0.018 mmol) was dissolved in THF (3 mL) and H_2O (1 mL) and cooled to −78 °C under argon. Diethylmethoxy borane (2.6 µL, 20 µmol) was added and the solution stirred for 30 min at −78 °C, before adding $NaBH_4$ (0.80 mg, 22 µmol).[a] After 2 h the reaction was quenched with glacial acetic acid (1 mL) and partitioned between H_2O (7 mL) and EtOAc (8 mL). After extraction, the aqueous layer was extracted with EtOAc (2 × 8 mL) and the combined organic extractions were washed with brine (10 mL), dried ($MgSO_4$), filtered and concentrated *in vacuo*. Purification by silica column chromatography (2% to 5% MeOH in CH_2Cl_2 with 0.5% CHCOOH) gave 1,3-*syn*-diol **4** as a colourless oil (9.1 mg, 91%). $[\alpha]_D^{21}$ −5.9 (*c* 0.3, MeOH); υ_{max} (neat) / cm^{-1} 3343 (OH and NH), 2924 (CH), 2854 (CH), 1706 (C=O), 1644 (C=O), 1455, 1378, 1234; δ_H (500 MHz, $CDCl_3$) 6.46 (1H, br s, NH), 6.24 (1H, ddd, *J* 15.0, 11.0, 1.5, 12-H), 5.95 (1H, t, *J* 11.0, 11-H), 5.69 (1H, br s, 2-H), 5.65 (1H, dt, *J* 15.0, 7.5, 13-H), 5.42 (2H, br s, NH_2), 5.30 (1H, dt, *J* 11.0, 7.5, 10-H), 4.91 (1H, dq, *J* 8.0, 6.5, 27-H), 4.79 (1H, s, 23-*H*H) 4.73 (1H, s, 23-H*H*), 4.02 (1H, m, 19-H), 3.98 (1H, m, 17-H), 3.38 (1H, m, 20-*H*H) 3.31 (1H, m, 20-H*H*), 2.51 (1H, m, 26-H), 2.32 (1H, m, 9-*H*H), 2.21 (1H, *overlapping* m, 9-H*H*), 2.15 (3H, d, *J* 1.0, 21-H_3), 2.10 (1H, *overlapping* m, 6-*H*H), 2.09 (1H, *overlapping* m, 14-*H*H) 2.08 (1H, *overlapping* m, 4-*H*H), 2.07 (2H, *overlapping* m, 8-H_2) 2.04 (1H, *overlapping* m, 4-H*H*), 1.97 (1H, *overlapping* m, 14-H*H*), 1.89 (1H, *overlapping* m, 5-H), 1.73 (1H, m, 6-H*H*), 1.73 (1H, m, 15-H), 1.55 (2H, m, 18-H_2), 1.54 (1H, m, 16-*H*H), 1.28 (3H, d, *J* 6.5, 28-H_3), 1.16 (1H, m, 16-*H*H) 1.15 (3H, d, *J* 7.0, 29-H_3), 0.91 (3H, d, *J* 6.5, 24-H_3), 0.86 (3H, d, *J* 6.5, 22-H_3); δ_C (125 MHz, $CDCl_3$) 174.3 (C-25), 169.5 (C-1), 160.9 (C-3), 157.6 (C-30),

147.4 (C-7), 133.0 (C-13), 129.4 (C-10), 129.1 (C-11), 127.1 (C-12), 116.8 (C-2), 112.0 (C-23), 73.7 (C-27), 72.0 (C-19), 70.3 (C-17), 49.1 (C-4), 47.1 (C-26), 45.6 (C-20), 45.2 (C-16), 43.1 (C-6), 41.0 (C-14), 40.8 (C-18), 35.7 (C-8), 29.9 (C-15), 29.7 (C-5), 26.7 (C-9), 20.0 (C-22), 19.7 (C-24), 19.2 (C-21), 18.1 (C-28), 13.9 (C-29); *m/z* (ESI) 573.35 $[M+Na]^+$; Found (ESI) 573.3515 ($C_{30}H_{50}O_7N_2Na$ requires 573.3510).

a) K. Narasaka, F. C. Pai *Tetrahedron*, **1984**, *40*, 2233.

General procedure for acetonide formation

PPTS (1 crystal) was added to a solution of the diol in 2,2-dimethoxypropane (2.0 mL) and CH_2Cl_2 (1.0 mL) at room temperature under nitrogen. After 16 h the reaction was diluted with CH_2Cl_2 (5 mL) and H_2O (5 mL) and the phases separated. The aqueous phase was extracted with CH_2Cl_2 (3 × 5 mL) and the combined organic extracts dried ($MgSO_4$), filtered and concentrated *in vacuo* to give the corresponding acetonide.

Preparation of *anti*-1,3-diol acetonide 7

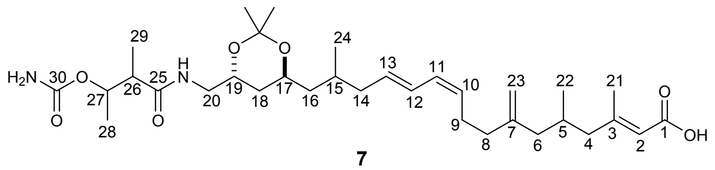

7

Diol **5** (17.0 mg, 30.9 µmol) was subjected to the conditions described above for the general procedure for acetonide formation. Acetonide **7** was isolated as a colourless oil (14.0 mg, 78%). $[\alpha]^{22}_{D}$ −12.0 (*c* 0.4, $CDCl_3$); δ_H (500 MHz, $CDCl_3$) 6.25 (1H, ddd, *J* 15.0, 11.0, 1.0, 12-H), 6.16 (1H, dd, *J* 6.5, 5.0, NH), 5.95 (1H, t, *J* 11.0, 11-H), 5.68 (1H, s, 2-H), 5.62 (1H, dt, *J* 15.0, 7.5, 13-H), 5.30 (1H, dt, *J* 11.0, 7.5, 10-H), 5.07 (2H, br s, NH_2), 4.91 (1H, *app.* quintet, *J* 6.5, 27-H), 4.81 (1H, d, *J* 1.5, 23-*H*H), 4.74 (1H, s, 23-H*H*), 3.90 (1H, m, 19-H), 3.86 (1H, m, 17-H), 3.57 (1H, ddd, *J* 14.0, 7.0, 3.0, 20-*H*H), 3.11 (1H, ddd, *J* 14.0, 8.0, 5.0, 20-H*H*), 2.52 (1H, *app.* quintet, *J* 7.0, 26-H), 2.29 (2H, m, 9-H_2), 2.17 (1H, *overlapping* m, 4-*H*H), 2.14 (3H, d, *J* 1.0, 21-H_3), 2.13 (1H, *overlapping* m, 14-*H*H), 2.06 (2H, *overlapping* m, 8-H_2), 2.04 (1H, *overlapping* m, 6-*H*H), 1.94 (1H, *overlapping* m, 14-*H*H), 1.93 (1H, *overlapping* m, 4-H*H*), 1.90 (1H, *overlapping* m, 5-H), 1.82 (1H, dd, *J* 13.5, 8.0, 6-H*H*), 1.66 (1H, m, 15-H), 1.55 (2H, m, 18-H_2), 1.40 (2H, m, 16-H_2), 1.35 (3H, s, $C(CH_3)$), 1.33 (3H, s, $C(CH_3)$), 1.27 (3H, d, *J* 6.5, 28-H_3), 1.15 (3H, d, *J* 7.0, 29-H_3), 0.89 (3H, d, *J* 6.5, 24-H_3), 0.84 (3H, d, *J* 6.0, 22-H_3); δ_C (125 MHz, $CDCl_3$) 173.7 (C-25), 170.2 (C-1), 161.2 (C-3), 156.9 (C-30), 147.3 (C-7), 133.0 (C-13), 129.4 (C-10), 129.0 (C-11), 127.2 (C-12), 116.8 (C-2), 111.6 (C-23), 100.6 (*C*$(CH_3)_2$), 73.2 (C-27), 66.2 (C-19), 65.1 (C-17), 48.9 (C-4), 46.5 (C-26), 43.9 (C-6), 43.4 (C-20), 42.6 (C-16), 40.1 (C-14), 35.7 (C-8), 35.6 (C-18), 29.9 (C-15), 29.1 (C-5), 26.1 (C-9), 25.1 (C(*C*H_3)), 24.9 (C(*C*H_3)), 20.2 (C-24), 19.7 (C-22), 19.1 (C-21), 17.5 (C-28), 13.5 (C-29); *m/z* (ESI) 590.39 $[M+Na]^+$; Found (ESI) 590.3943 ($C_{33}H_{54}O_7N_2Na$ requires 590.3931).

Preparation of *syn*-1,3-diol acetonide 6

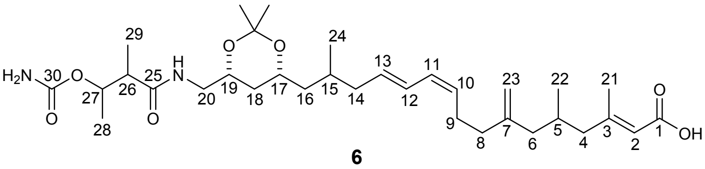

6

Diol **4** (5.0 mg, 9.1 µmol) was subjected to the conditions described above for the general procedure for acetonide formation. Acetonide **6** was isolated as a colourless oil (5 mg, 92%). $[\alpha]_D^{22}$ +8.2 (*c* 0.4, $CDCl_3$); δ_H (500 MHz, $CDCl_3$) 6.24 (1H, ddd, *J* 15.0, 11.0, 1.0, 12-H) 6.10 (1H, t, *J* 5.5, NH), 5.95 (1H, t, *J* 11.0, 11-H), 5.69 (1H, s, 2-H), 5.63 (1H, dt, *J* 15.0, 7.5 13-H), 5.29 (1H, dt, *J* 11.0, 7.5, 10-H), 5.11 (2H, br s, NH_2), 4.88 (1H, m, 27-H), 4.79 (1H, s, 23-*H*H), 4.73 (1H, s, 23-H*H*), 4.01 (1H, m, 19-H), 3.98 (1H, m, 17-H), 3.34 (2H, m, 20-H_2), 2.47 (1H, m, 26-H), 2.30 (1H, m, 9-*H*H), 2.22 (1H, *overlapping* m, 9-H*H*), 2.15 (3H, s, 21-H_3) 2.13 (1H, *overlapping* m, 4-*H*H), 2.11 (1H, *overlapping* m, 14-*H*H), 2.10 (1H, *overlapping* m, 6-*H*H) 2.05 (2H, *overlapping* m, 8-H_2), 2.01 (1H, *overlapping* m, 4-H*H*), 1.97 (1H,*overlapping* m, 14-H*H*), 1.89 (1H, *overlapping* m, 5-H), 1.76 (1H, *overlapping* m, 15-H), 1.75 (1H, *overlapping* m, 6-H*H*), 1.52 (2H, m, 18-*H*H), 1.41 (1H, m, 16-*H*H), 1.38 (1H, m, 16-H*H*), 1.31 (3H, d, *J* 6.5, 28-H_3), 1.16 (3H, d, *J* 7.0, 29-H_3), 1.11 (1H, m, 18-H*H*), 0.89 (3H, d, *J* 6.5, 24-H_3), 0.85 (3H, d, *J* 6.5, 22-H_3); δ_C (125 MHz, $CDCl_3$) 173.8 (C-25), 169.7 (C-1), 160.8 (C-3), 157.2 (C-30), 147.4 (C-7), 133.1 (C-13), 129.4 (C-10), 129.0 (C-11), 127.0 (C-12), 116.8 (C-2), 112.0 (C-23), 98.9 ($C(CH_3)_2$), 73.7 (C-27), 68.4 (C-19), 66.6 (C-17), 49.1 (C-4), 47.0 (C-26), 43.6 (C-20), 43.2 (C-6), 43.0 (C-18), 40.7 (C-14), 35.4 (C-8), 34.3 (C-16), 30.3 ($C(CH_3)_2$), 29.0 (C-5), 28.9 (C-15), 26.3 (C-9), 19.9 ($C(CH_3)_2$), 19.5 (C-22), 19.0 (C-21), 17.8 (C-28), 14.3 (C-24), 13.9 (C-29); *m/z* (ESI) 590.39 $[M+Na]^+$; Found (ESI) 590.3941 ($C_{33}H_{54}O_7N_2Na$ requires 590.3931).

Preparation of *anti*-1,3-diol acetonide 7 from diol 2

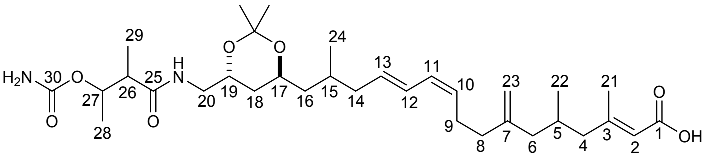

Diol **2** (23.0 mg, 41.7 µmol) was subjected to the conditions described above for the general procedure for acetonide formation. Acetonide was isolated as a colourless oil (16 mg, 64%). Data same as for 1,3-*anti* acetonide **7**.

5e) Preparation of synthetic fragments 8, 9 and S5

(2*R*, 3*R*)-*N*-Allyl-3-(*tert*-butyldimethylsilyloxy)-2-methylbutanamide S2

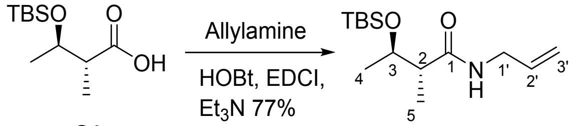

S1 **S2**

Acid **S1** (500 mg, 2.15 mmol) and allylamine (0.3 mL, 4.3 mmol) were dissolved in anhydrous DMF (9 mL) under an atmosphere of nitrogen. The reaction was cooled to 0 °C and then followed by adding EDCI·HCl (618 mg, 3.2 mmol), HOBt (58 mg, 0.4 mmol) and triethylamine (0.5 mL, 3.2 mmol). The reaction mixture was warm up to room temperature and stirred for 15 hours. After the reaction completed azeotrope of DMF with toluene (3 × 60 mL) at 60 °C under the vacuum. The crude residue was dilute with 1 N citric acid (2 mL), extract with EtOAc (3 × 10 mL). The organic extracts was washed with saturated aqueous $NaHCO_3$ (30 mL) and brine (30 mL) and then dried over $MgSO_4$, filtered, and concentrated *in vacuo.* The crude residue was purified by flash column chromatography (SiO_2, 10% EtOAc in petroleum ether 40-60 °C) to give amide **S2** (447 mg, 77%) as a yellow oil. $[\alpha]_D^{21}$ −13 (*c* 1.0, $CHCl_3$); ν_{max} (neat)/cm^{-1} 3302 (NH), 2929 (CH), 1640 (C=O), 1544, 1254, 1114, 836; δ_H (400 MHz, $CDCl_3$) 6.61 (1H, s, NH), 5.83 (1H, m, 2'-H), 5.15 (2H, m, 3'-H_2), 3.88 (3H, m, 3-H and 1'-H_2), 2.28 (1H, qd, *J* 7.0, 4.0, 2-H). 1.22 (3H, d, *J* 6.5, 4-CH_3), 1.20 (3H, d, *J* 7.0, 5-CH_3), 0.89 (9H, s, $SiC(CH_3)_3$), 0.09 (3H, s, $SiCH_3$), 0.07 (3H, s, $SiCH_3$); δ_C (100 MHz, $CDCl_3$) 175.0 (C-1), 134.6 (C-2'), 116.5 (C-3'), 70.8 (C-3), 49.3 (C-2), 41.7 (C-1'), 26.0 ($SiC(CH_3)_3$), 22.2 (C-5), −4.3 ($SiCH_3$), −4.9 ($SiCH_3$); Found (ESI): 294.1870 $[MNa]^+$, (required $C_{14}H_{29}O_2NNaSi$ 294.1860).

(2*R*,3*R*)-3-(*tert*-Butyldimethylsilyloxy)-2-methyl-*N*-(2'*R*-hydroxypent-4'-ene)butanamide S3

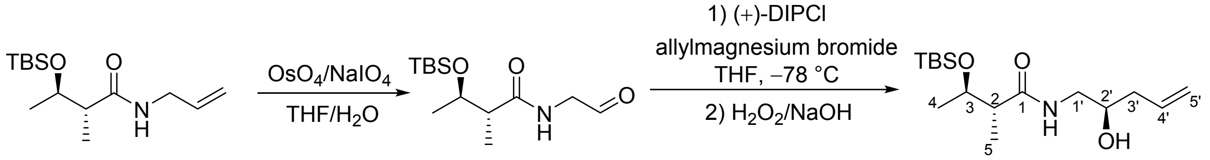

S2 **S3**

A single crystal of osmium tetroxide (cat.) was added to a stirred solution of olefin **S2** (2.60 g, 9.4 mmol) and sodium *m*-periodate (9.00 g, 42.3 mmol) in THF/H_2O (1:1, 20 mL). The mixture was stirred at room temperature until TLC monitoring shows no starting material left (about 1 h). Then the reaction mixture was diluted with water (10 mL) and acidified to pH 2 with 1 M HCl solution. The mixture was extracted with EtOAc (3 × 30 mL) and the combined organic layers were washed with brine (30 mL), dried over $MgSO_4$, filtered and concentrated *in vacuo* to give aldehyde (300 mg) as a dark oil which was used immediately in the next step.

(+)-B-Chlorodiisopinocampheylborane (527 mg, 1.76 mmol) was dissolved in anhydrous THF (9 mL) under an atmosphere of nitrogen at −78 °C. A solution of allylmagnesium bromide (1.0 M in

Et_2O, 1.76 mL, 1.76 mmol) was added dropwise. The reaction mixture was stirred for 1 hour at −78 °C before being allowed to warm up to room temperature over 1 h. The solution was re-cooled to −78 °C and a solution of crude aldehyde (300 mg) in dry THF (3 mL) was added dropwise. The reaction mixture was stirred at −78 °C for 1.75 h before being allowed to warm up to room temperature. The reaction was quenched by the addition of a preformed mixture of NaOH solution (1.5 mL, 3 M aq.) and hydrogen peroxide (0.45 ml, 30 % aq. sol.). The mixture was stirred for 30 min before addition of EtOAc (30 mL) and water (15 mL). The layers were separated and the aqueous phase was extracted with EtOAc (4 × 20 mL). The combined organic extracts were washed with water (30 mL) and brine (30 mL), then dried over $MgSO_4$, filtered, and concentrated *in vacuo*. The crude residue was purified by flash column chromatography (SiO_2, 20% EtOAc in petroleum ether 40-60 °C) to give alcohol **S3** (279.3 mg, 81%) as a light yellow oil; $[\alpha]_D^{21}$ −2.0 (*c* 1.0, $CHCl_3$); ν_{max} (neat)/cm^{-1} 3320 (OH, NH), 2929 (CH), 1642 (C=O), 1543, 1252, 1115, 834; δ_H (400 MHz, $CDCl_3$) 6.98 (1H, s, NH), 5.81 (1H, m, 4'-H), 5.14 (2H, m, 5'-H_2), 3.89 (1H, m, 3-H), 3.76 (1H, m, 2'-H), 3.54 (1H, ddd, *J* 14.0, 7.0, 3.0, 1'-*H*H), 3.09 (1H, ddd, *J* 14.0, 7.0, 5.0, 1'-H*H*), 2.33 (1H, m, 2-H), 2.24 (2H, m, 3'-H_2), 1.23 (3H, d, *J* 6.5, 4-H_3), 1.20 (3H, d, *J* 7.0, 5-H_3), 0.9 (9H, s, $SiC(CH_3)_3$), 0.1 (3H, s, $SiCH_3$), 0.08 (3H, s, $SiCH_3$); δ_C (100 MHz, $CDCl_3$) 176.8 (C-1), 134.3 (C-4'), 118.4 (C-5'), 70.8 (C-1'), 70.7 (C-2'), 49.1 (C-2), 45.2 (C-3), 39.6 (C-3'), 26.0 ($SiC(CH_3)_3$), 22.2 (C-4), 18.1 ($SiC(CH_3)_3$), 16.5 (C-5), −4.2 ($SiCH_3$), −4.9 ($SiCH_3$); Found (ESI): 338.2134 [MNa^+], (required $C_{16}H_{33}O_3NNaSi$ 338.2122).

Mosher's ester derivatives of homoallylic alcohol S3

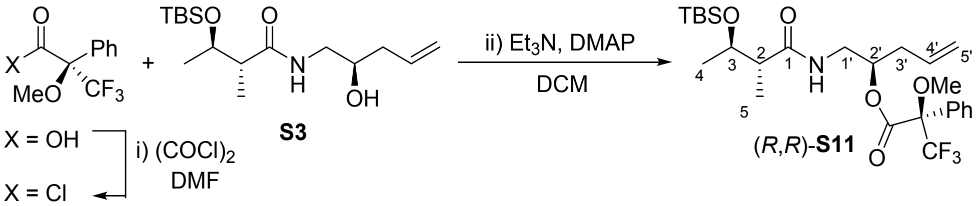

Oxalyl chloride (0.21 mL, 2.38 mmol) was added to a stirred solution of (*R*)-(+)-*α*-methoxy-*α*-(trifluoromethyl)phenylacetic acid (111 mg, 0.48 mmol), and *N*,*N*-dimethylformamide (63 mmL, 0.71 mmol) in anhydrous hexane (20 mL) at room temperature under an atmosphere of nitrogen. The reaction mixture was stirred for 1 h before the hexane layer was removed by pipette from the oily Vilsmeier salt at the bottom. The hexane solution was concentrated *in vacuo* to give the acid chloride as a colourless oil. The acid chloride in anhydrous DCM (2 mL) was added to a solution of alcohol **S3** (30 mg, 95 µmol), Et_3N (0.4 mL, 3.0 mmol) and DMAP (9 mg, 0.072 mmol) in anhydrous DCM (5 mL) at room temperature under an atmosphere of nitrogen. The mixture was stirred until TLC indicated to the alcohol had been consumed for 5 h before being diluted by the addition of DCM (20 mL). The solution was washed with H_2O (20 mL) and saturated aqueous $NaHCO_3$ (20 mL) before being dried over $MgSO_4$, filtered and concentrated *in vacuo*. The crude product was purified by flash column chromatography (SiO_2, 10% EtOAc in petroleum ether 40-60 °C) to give ester (*R*,*R*)-**S11** (40 mg, 79%) as a pale yellow oil; ν_{max} (neat)/cm^{-1} 3323 (NH), 2956 (CH), 2930 (CH), 1747 (C=O), 1651 (C=O), 1452, 1251, 1167, 1016, 834; δ_H (300 MHz, $CDCl_3$) 7.52-7.41 (5H, m, Ar), 6.38 (1H, s, NH), 5.65 (1H, m, 4'-H), 5.23 (1H, m, 2'-H), 5.05 (2H, m, 5'-H_2), 3.87 (1H, m, 3-H), 3.63 (2H, m, 1'-H_2), 3.52 (3H, s, OMe), 2.37 (2H, m, 3'-H_2), 2.18 (1H, m, 2-H), 1.13 (3H, d, *J* 7.0, 4-H_3), 1.09 (3H, d, *J* 7.0, 5-H_3), 0.87 (9H, s, $SiC(CH_3)_3$), 0.06 (3H, s, $SiCH_3$), 0.03 (3H, s, $SiCH_3$).

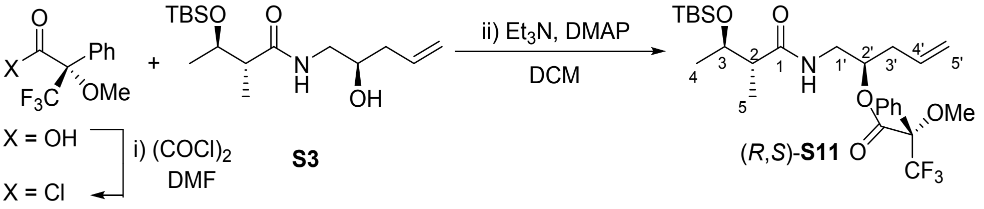

The above procedure was repeated using (*S*)-(−)-*α*-methoxy-*α*-(trifluoromethyl)phenylacetic acid (111 mg, 0.48 mmol). The crude residue was purified by flash column chromatography (SiO_2, 10% EtOAc in petroleum ether 40-60 °C) to give ester (*R*,*S*)-**S11** (45 mg, 89%) as a pale yellow oil; $[\alpha]_D^{23}$ −36.8 (*c* 0.7, $CHCl_3$); ν_{max} (neat)/cm^{-1} 3330 (NH), 2955 (CH), 2931 (CH), 1747 (C=O), 1653 (C=O), 1451, 1251, 1166, 1119, 1016, 835; δ_H (400 MHz, $CDCl_3$) 7.53-7.41 (5H, m, Ar), 6.01 (1H, s, NH), 5.76 (1H, m, 4'-H), 5.21 (1H, m, 2'-H), 5.14 (2H, m, 5'-H_2), 3.85 (1H, quint., *J* 12.5, 6.0, 3-H) 3.56 (3H, s, OMe), 2.44 (2H, m, 3'-H_2), 2.06 (1H, m, 2-H), 1.12 (3H, d, *J* 6.5, 4-H_3), 1.08 (3H, d, *J* 7.0, 5-H_3), 0.85 (9H, s, $SiC(CH_3)_3$), 0.05 (3H, s, $SiCH_3$), 0.01 (3H, s, $SiCH_3$); Found (ESI): 554.2525 [MNa^+], (required $C_{26}H_{40}O_5NF_3SiNa$ 554.2520).

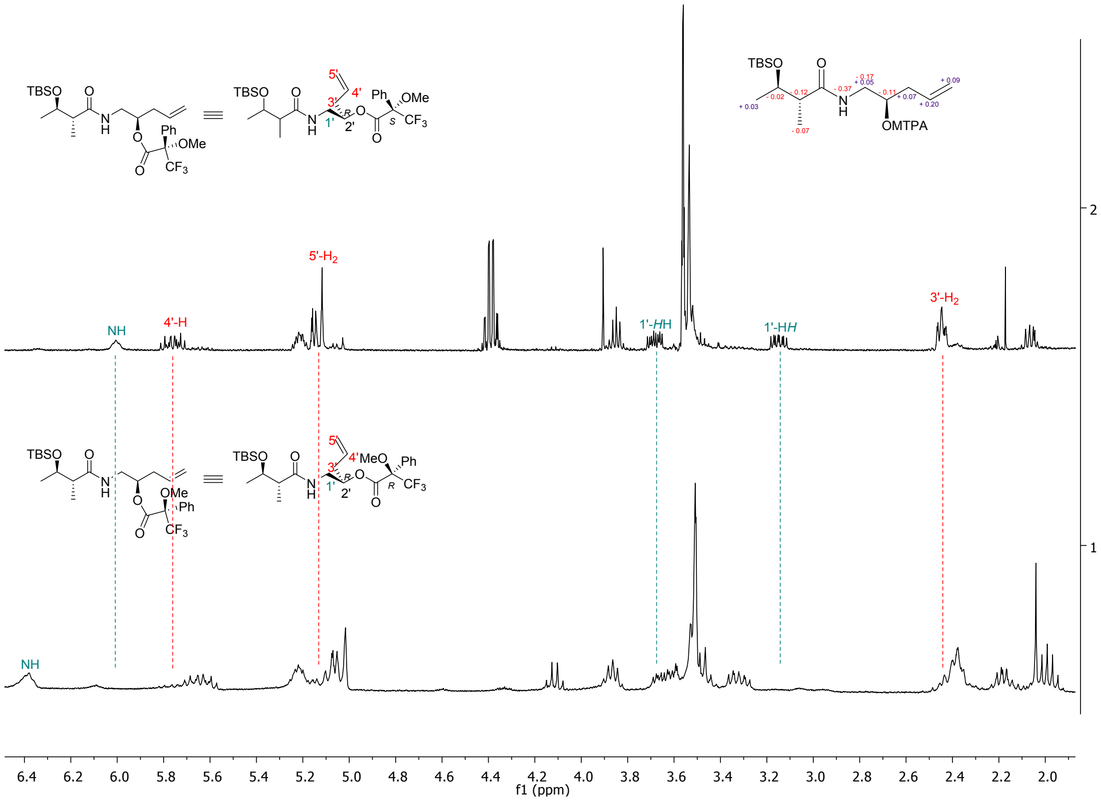

Figure S4. Comparison of ^{1}H NMR (400 MHz, $CDCl_3$) spectrum of Mosher's ester derivatives of (*R*, *R*)-**S11** and (*R*, *S*)-**S11**.

(2*R*, 3*R*)-3-(*tert*-Butyldimethylsilyloxy)-2-methyl-*N*-(2'*R*-hydroxypent-4'one)butanamide S12

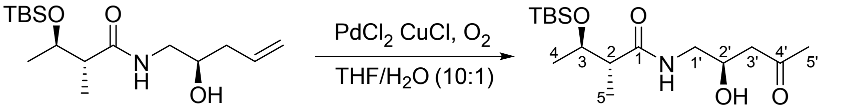

S3 **S12**

$PdCl_2$ (19 mg, 0.11 mmol) and CuCl (106 mg, 1.07 mmol) was added to a stirred solution in 19 mL THF: H_2O (10:1) under an atmosphere pure O_2. Alcohol **S3** (337 mg, 1.07 mmol) was dissolved in THF (2 mL) added dropwise to the mixture. The reaction mixture was stirred for 3 hours at room temperature and then filtered through Celite. The filtrate was extracted with DCM (3 × 40 mL). The combined organic layers were dried over $MgSO_4$, filtered and concentrated *in vacuo*. The crude residue was purified by flash column chromatography (SiO_2, 25% EtOAc in petroleum ether 40-60 °C) to give hydroxyl ketone **S12** (164 mg, 46%) as a light yellow oil. $[\alpha]_D^{21}$ −1.0 (*c* 1.0, $CHCl_3$); ν_{max} (neat)/cm^{-1} 3325 (OH, NH), 2955 (CH), 2929 (CH), 1712 (C=O), 1645 (C=O), 1544, 1251, 1114, 835; δ_H (400 MHz, $CDCl_3$) 6.93 (1H, br. s, NH), 4.11 (1H, m, 2'-H), 3.89 (1H, m, 3-H), 3.51 (1H, ddd, *J* 14.0, 7.0, 3.5, 1'-H*H*), 3.11 (1H, ddd, *J* 14.0, 7.0, 5.0, 1'-*H*H), 2.61 (2H, m, 3'-H_2), 2.28 (1H, m, 2-H), 2.18 (3H, s, 5'-H_3), 1.21 (3H, d, *J* 6.5, 4-H_3), 1.19 (3H, d, *J* 7.0, 5-H_3) 0.9 (9H, s, $SiC(CH_3)_3$), 0.09 (3H, s, $SiCH_3$), 0.07 (3H, s, $SiCH_3$); δ_C (100 MHz, $CDCl_3$) 209.3 (C-4'), 176.3 (C-1), 70.6 (C-2'), 67.5 (C-3), 49.2 (C-2), 47.4 (C-3'), 44.4 (C-1'), 30.9 (C-5'), 25.9 ($SiC(CH_3)_3$), 22.1 (C-4), 18.07 ($Si\mathit{C}(CH_3)_3$), 16.4 (C-5), −4.3 ($SiCH_3$) and −4.9 ($SiCH_3$); Found (ESI): 354.2081 [MNa^+], (required $C_{16}H_{33}O_4NNaSi$ 354.2071).

(2*R*, 3*R*)-3-Hydroxy-2-methyl-*N*-(2'*R*-hydroxypent-4'-one)butanamide 8

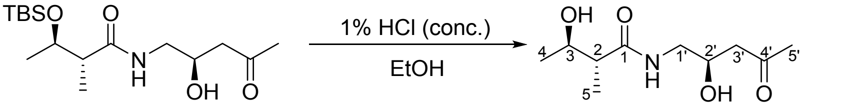

S12 **8**

A solution of amide **S12** (100 mg, 0.3 mmol) in ethanol (3 mL) was added concentrated HCl (5 drops), to adjust the pH to 2, and the reaction was stirred at room temperature for 2 h. The reaction was quenched with saturated aqueous $NaHCO_3$ (3 mL) and the layers were separated. The aqueous phase was extracted into EtOAc (3 × 30 mL). The organic layers were combined, dried over $MgSO_4$, filtered, and concentrated *in vacuo*. The crude residue was purified by flash column chromatography (SiO_2, 5% MeOH in DCM) to give diol **8** (25 mg, 30%) as a colourless oil; $[\alpha]_D^{21}$ −4.0 (c 0.5, $CHCl_3$); ν_{max} (neat)/cm^{-1} 3317 (OH, NH), 2972 (CH), 2931 (CH), 1707 (C=O), 1641 (C=O), 1544, 1370, 1100, 916; δ_H (500 MHz, Acetone-d_6) 7.27 (1H, s, NH), 4.19 (1H, d, *J* 4.7, 3-OH), 4.10 (1H, m, 2'-H), 4.04 (1H, d, *J* 6.0, 2'-OH), 3.76 (1H, h, *J* 6.0, 3-H), 3.27 (2H, m, 1'-H_2), 2.57 (2H, m, 3'-H_2), 2.30 (1H, quint., *J* 7.0, 2-H), 2.11 (3H, s, 5'-H_3) 1.15 (3H, d, *J* 6.3, 4-H_3), 1.11 (3H, d, *J* 7.0, 5-H_3); δ_C (125 MHz, Acetone-d_6) 207.7 (C-4'), 177.0 (C-1), 70.1 (C-3), 68.0 (C-2'), 48.6 (C-3'), 48.3 (C-2), 45.6 (C-1'), 30.7 (C-5'), 21.9 (C-4), 15.1 (C-5); Found (ESI): 240.1215 [MNa^+], (required $C_{10}H_{19}O_4NaN$ 240.1206).

(2*R*,3*R*)-3-(*tert*-Butyldimethylsilyloxy)-2-methyl-*N*-(3'*R*-hydroxypent-4'-ene)butanamide S4

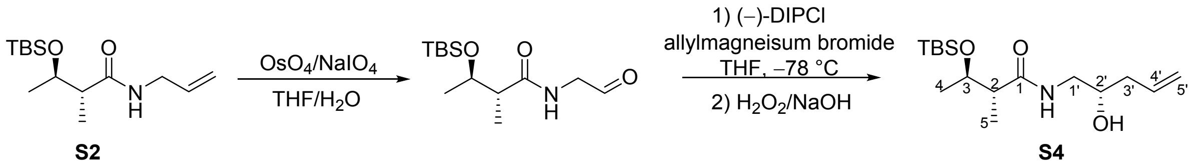

A single crystal of osmium tetroxide (cat.) was added to a stirred solution of olefin **S2** (3.56 g, 12.9 mmol) and sodium *m*-periodate (12.3 g, 60 mmol) in THF/H_2O (1:1, 20 mL). The mixture was stirred at room temperature until TLC monitoring shows no starting material left (about 1 h). Then the reaction mixture was diluted with water (10 mL) and acidified to pH 2 with 1 M HCl solution. The mixture was extracted with EtOAc (3 × 30 mL) and the combined organic layers were washed with brine (30 mL), dried over $MgSO_4$, filtered and concentrated *in vacuo* to give aldehyde (412 mg) as a dark oil which was used immediately in the next step.

A stirred solution of (–)-B-chlorodiisopinocampheylborane (773 mg, 2.42 mmol) in anhydrous THF (10 mL) was cooled to −78 °C under an atmosphere of nitrogen. A solution of allylmagnesium bromide (1.0 M in Et_2O, 2.4 mL, 2.4 mmol) was added dropwise. The reaction mixture was stirred for 1 h at −78 °C before being allowed to warm up to room temperature over a further 1 h. The solution was re-cooled to −78 °C before aldehyde (412 mg, 1.51 mmol) in dry THF (3 mL) was added dropwise. The reaction was stirred at −78 °C for 1.75 h before being allowed to warm up to room temperature. The reaction was quenched by the addition of a preformed mixture of NaOH (2 mL, 3 M aq.) and hydrogen peroxide (0.6 mL, 30% aq. Sol.). The reaction mixture was stirred for 30 min before addition of EtOAc (40 mL) and water (20 mL). The layers were separated and the aqueous phase was extracted with EtOAc (4 × 30 mL). The combined organic extracts were washed with water (40 mL) and brine (40 mL) and then dried over $MgSO_4$, filtered, and concentrated *in vacuo* to give a yellow oil. The crude residue was purified by flash column chromatography (SiO_2, 20% EtOAc in petroleum ether 40-60 °C) to give alcohol **S4** as a light yellow oil (246 mg, 66%); $[\alpha]_D^{21}$ −7.1 (*c* 0.28, $CHCl_3$); ν_{max} (neat)/cm^{-1} 3321 (OH, NH), 2956 (CH), 2928 (CH), 1643 (C=O), 1543, 1252, 1115, 835; δ_H (400 MHz, $CDCl_3$) 6.98 (1H, s, NH), 5.81 (1H, m, 4'-H), 5.14 (2H, m, 5'-H_2), 3.89 (1H, m, 3-H), 3.75 (1H, m, 2'-H), 3.38 (1H, ddd, *J* 14.1, 6.8, 3.0, 1'-*H*H), 3.25 (1H, ddd, *J* 14.0, 7.1, 5.1, 1'-H*H*), 2.31 (1H, m, 2-H), 2.24 (2H, m, 3'-H_2), 1.22 (3H, d, *J* 6.5, 4-H_3), 1.20 (3H, d, *J* 7.0, 5-H_3), 0.9 (9H, s, $SiC(CH_3)_3$), 0.1 (3H, s, $SiCH_3$), 0.08 (3H, s, $SiCH_3$); δ_C (100 MHz, $CDCl_3$) 176.91 (C-1), 134.29 (C-4'), 118.38 (C-5'), 71.02 (C-1'), 70.65 (C-2'), 49.16 (C-2), 45.21 (C-3), 39.68 (C-3'), 25.95 ($SiC(CH_3)_3$), 22.13 (C-4), 18.08 ($Si\mathit{C}(CH_3)_3$), 16.52 (C-5), −4.26 ($SiCH_3$) and −4.89 ($SiCH_3$); Found (ESI): 338.2124 [MNa^+], (required $C_{16}H_{33}O_3NaNSi$ 338.2122).

Mosher's ester derivatives of alcohol S4

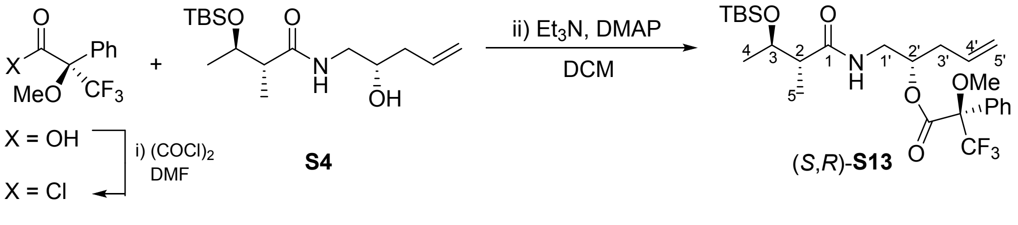

Oxalyl chloride (0.21 mL, 2.38 mmol) was added to a stirred solution of (*R*)-(+)-*α*-methoxy-*α*-(trifluoromethyl)phenylacetic acid (111 mg, 0.48 mmol), and *N*,*N*-dimethylformamide (60 mL, 0.71 mmol) in dry hexane (20 mL) at room temperature under an atmosphere of nitrogen. The mixture was stirred for 1 h before the hexane layer was removed from the oily Vilsmeier salt at the bottom. The hexane solution was concentrated *in vacuo* to give the acid chloride as a colourless oil. The acid chloride was added to a solution of the alcohol **S4** (30 mg, 95 µmol), Et_3N (0.4 mL, 2.85 mmol) and DMAP (9 mg, 72 µmol) in dry DCM (5 mL) at room temperature under an atmosphere of nitrogen. The reaction mixture was stirred until TLC indicated the alcohol had been consumed (3 h) before being diluted with DCM (20 mL). The solution was washed with water (20 mL) and saturated aqueous $NaHCO_3$ (20 mL) and then dried over $MgSO_4$, filtered, and concentrated *in vacuo*. The crude residue was purified by flash column chromatography (SiO_2, 10% EtOAc in petroleum ether 40-60 °C) to give ester (*S*,*R*)-**S13** (37 mg, 73%) as a pale yellow oil; ν_{max} (neat)/cm^{-1} 3322 (NH), 2955 (CH), 2930 (CH), 1748 (C=O), 1652 (C=O), 1451, 1251, 1167, 835; δ_H (300 MHz, $CDCl_3$) 7.55-7.52 (2H, m, Ar), 7.43-7.38 (3H, m, Ar), 6.03 (1H, s, NH), 5.77 (1H, m, 4'-H), 5.21 (1H, m, 2'-H), 5.13 (2H, m, 5'-H_2), 3.87 (1H, quint., *J* 6.0, 3-H), 3.53 (3H, d, *J* 1.0, OMe), 3.46 (2H, m, 1'-H_2), 2.43 (2H, m, 3'-H_2), 2.14 (1H, m, 2-H), 1.15 (3H, d, *J* 6.5, 4-H_3), 1.04 (3H, d, *J* 7.0, 5-H_3), 0.87 (9H, s, $SiC(CH_3)_3$), 0.07 (3H, s, $SiCH_3$), 0.03 (3H, s, $SiCH_3$); Found (ESI): 554.2516 [MNa^+], (required $C_{26}H_{40}O_5NF_3SiNa$ 554.2520).

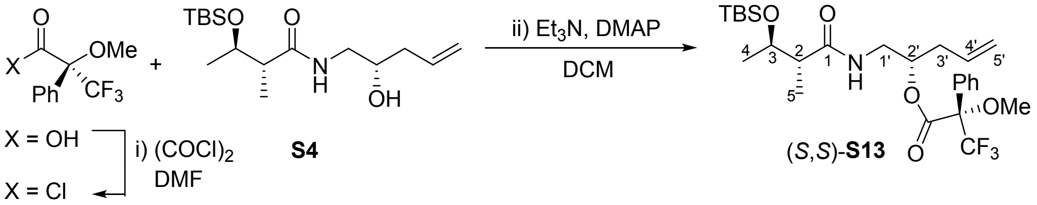

The above reaction was repeated using (*S*)-(−)-*α*-methoxy-*α*-(trifluoromethyl)phenylacetic acid (111 mg, 0.48 mmol).The crude residue was purified by flash column chromatography (SiO_2, 10% EtOAc in petroleum ether 40-60 °C) to give ester (*S*,*S*)-**S13** as a pale yellow oil (33 mg, 66%); ν_{max} (neat)/cm^{-1} 3325 (NH), 2954 (CH), 2929 (CH), 1747 (C=O), 1656 (C=O), 1451, 1252, 1167, 836; δ_H (300 MHz, $CDCl_3$) 7.54-7.51 (2H, m, Ar), 7.43-7.38 (3H, m, Ar), 6.32 (1H, s, NH), 5.65 (1H, m, 4'-H), 5.21 (1H, m, 2'-H), 5.05 (2H, m, 5'-H_2), 3.88 (1H, quint., *J* 6.0, 3-H), 3.51 (3H, d, *J* 1.0, OCH_3), 3.51 (2H, m, 1'-H_2), 2.38 (2H, m, 3'-H_2), 2.21 (1H, m, 2-H), 1.16 (3H, d, *J* 6.5, 4-H_3), 1.11 (3H, d, *J* 7.0, 5-H_3), 0.87 (9H, s, $SiC(CH_3)_3$), 0.07 (3H, s, $SiCH_3$), 0.04 (3H, s, $SiCH_3$).

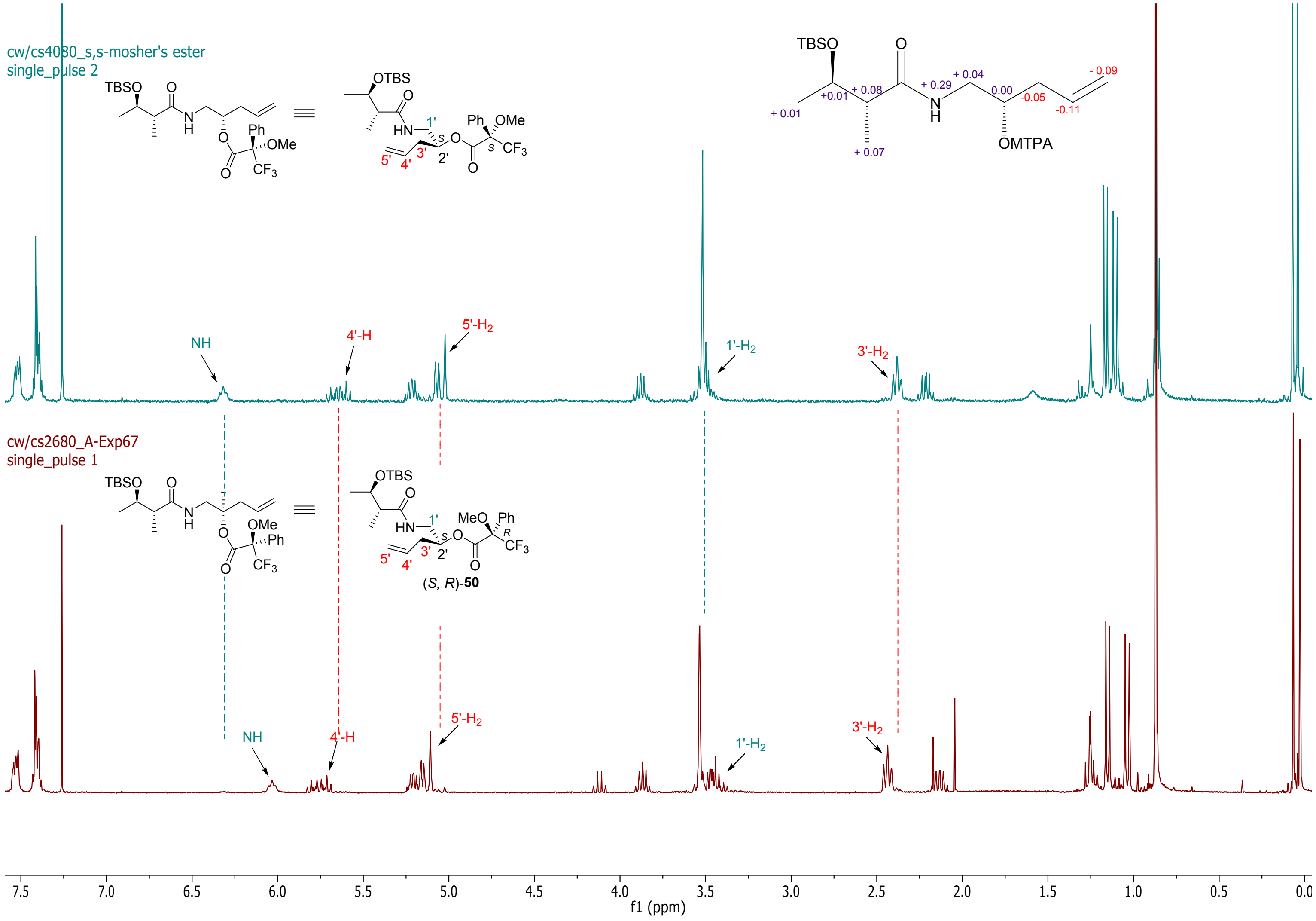

Figure S5. Comparison of ^{1}H NMR (400 MHz, $CDCl_3$) spectrum of Mosher's ester derivatives of (*S*, *R*)-**S13** and (*S*, *S*)-**S13**

(2*R*, 3*R*)-3-(*tert*-butyldimethylsilyloxy)-2-methyl-*N*-(2'*R*-hydroxypent-4'one)butanamide S14

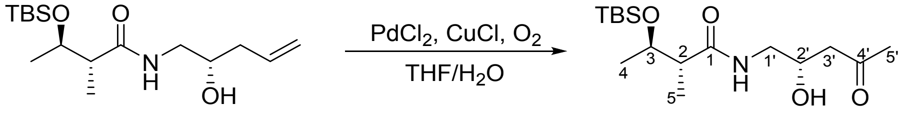

S4 **S14**

To a stirred solution of $PdCl_2$ (12 mg, 0.068mmol) and CuCl (67 mg, 0.68mmol) in 10:1 THF: H_2O (16 mL) under an atmosphere of pure O_2, was added unsaturated alcohol **S4** (216 mg, 1.07mmol) in THF (2 mL). The reaction was allowed to stir for 3 hours at room temperature before being filtered through Celite. The filtrate was extracted with DCM (3 × 40 mL), and the combined organic layers were dried over $MgSO_4$, filtered and concentrated *in vacuo*. The crude product was purified by flash column chromatography (SiO_2, 10-25% EtOAc in petroleum ether 40-60 °C) to give a yellow oil **S14** (101 mg, 45%); $[\alpha]_D^{21}$ −29 (*c* 1.0, $CHCl_3$); ν_{max} (neat)/cm^{-1} 3343 (OH, NH), 2926 (CH), 2853 (CH), 1712 (C=O), 1645 (C=O), 1544, 1251, 1110, 836; δ_H (400 MHz, $CDCl_3$) 6.89 (1H, s, NH), 4.10 (1H, m, 2'-H), 3.88 (1H, m, 3-H), 3.36 (1H, ddd, *J* 14.0, 6.0, 3.5, 1'-H*H*), 3.26 (1H, ddd, *J* 14.0, 6.5, 6.0, 1'-*H*H), 2.61 (2H, d, *J* 6.0, 3'-H_2), 2.28 (1H, qd, *J* 4.5, 7.0, 2-H), 2.17 (3H, s, 5'-H_3), 1.20 (3H, d, *J* 6.5, 4-H_3), 1.19 (3H, d, *J* 7.5, 5-H_3), 0.9 (9H, s, $SiC(CH_3)_3$), 0.09 (3H, s, $SiCH_3$), 0.07 (3H, s, $SiCH_3$); Found (ESI): 354.2080 [MNa^+], (required $C_{16}H_{33}O_4NaNSi$ 354.2071).

(2*R*, 3*R*)-3-hydroxy-2-methyl-*N*-(2'*R*-hydroxypent-4'-one)butanamide S5

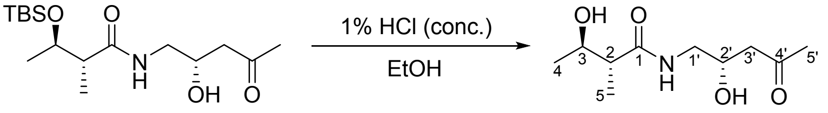

S14 **S5**

To a solution of amide **S14** (100 mg, 0.3 mmol) in ethanol (3 mL) was added concentrated HCl (5 drops), to adjust the pH to 2, and the mixture stirred at room temperature for 1 h. The reaction was quenched with saturated aqueous $NaHCO_3$ (3 mL) and then extracted into EtOAc (3 × 30 mL). The organic layers were combined, dried over $MgSO_4$, filtered and concentrated *in vacuo*. The colourless oil was purified by flash column chromatography (5% MeOH in DCM) to give alcohol **S5** as a colourless oil (25 mg, 30%); $[\alpha]_D^{22}$ −23 (*c* 0.3 , $CHCl_3$); ν_{max} (neat)/cm^{-1} 3321 (OH, NH), 2971 (CH), 2923 (CH), 1708 (C=O), 1644 (C=O), 1545, 1371, 1110, 913; δ_H (500 MHz, Acetone-d_6) 7.31 (1H, s, NH), 4.26 (1H, d, *J* 5.0, 2'-OH), 4.11 (1H, m, 2'-H), 4.07 (1H, d, *J* 6.0, 3-OH), 3.76 (1H, sextet, *J* 6.0, 3-H), 3.33 (1H, ddd, *J* 14.0, 6.0, 4.5, 1'-*H*H), 3.18 (1H, ddd, *J* 14.0, 6.5, 6.0, 1'-H*H*), 2.57 (2H, m, 3'-H_2), 2.30 (1H, quint., *J* 7.0, 2-H), 2.11 (3H, s, 5'-H_3) 1.15 (3H, d, *J* 6.5, 4-H_3), 1.11 (3H, d, *J* 7.0, 5-H_3); δ_C (125 MHz, Acetone-d_6) 207.7 (C-4'), 177.13 (C-1), 70.1 (C-3), 68.0 (C-2'), 48.7 (C-3'), 48.4 (C-2), 45.7 (C-1'), 30.7 (C-5'), 21.8 (C-4), 15.1 (C-5); Found (ESI): 240.1210 [MNa^+], (required $C_{10}H_{19}O_4NaN$ 240.1206).

(*S*)-3-((2*S*, 3*R*)-3-Hydroxy-2-methylbutanoyl)-4-isopropyloxazolidin-2-one S6

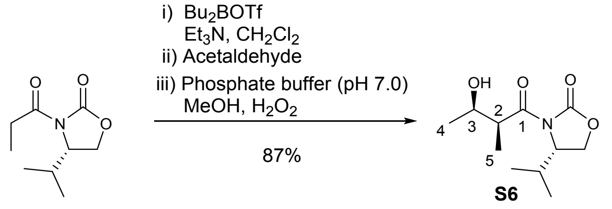

Propionyloxazolidone (3.60 g, 19.45 mmol) was dissolved in dry CH_2Cl_2 (34 mL) and cooled to −78 °C under nitrogen. Bu_2BOTf (39.0 mL, 1 M in CH_2Cl_2, 38.9 mmol) was added slowly followed after 10 min by the dropwise addition of Et_3N (6.8 mL, 48.62 mmol). The solution was stirred for 1 h at −78 °C followed by 100 min at 0 °C. The solution was cooled to −78 °C, and a −78 °C solution of acetaldehyde (11 mL, 194.48 mmol) in dry CH_2Cl_2 (3 mL) was added slowly. After stirring for 2 h at −78 °C then for 90 min at 0 °C, pH 7.0 phosphate buffer (100 mL) and MeOH (100 mL) were added, and the resulting mixture was stirred vigorously in an ice bath as 30% H_2O_2 (50 mL) was added dropwise. After 30 min at ~10-15 °C, the solvent was removed *in vacuo* and the residue was partitioned between saturated $NaHCO_{3(aq)}$ solution (20 mL) and CH_2Cl_2 (30 mL). The aqueous layer was extracted with CH_2Cl_2 (2 × 30 mL) and the combined organic layers were washed with saturated $NaHCO_{3(aq)}$ solution (30 mL), dried ($MgSO_4$) and concentrated *in vacuo* to give a crude yellow oil. The crude material was purified by silica column chromatography (20% to 40% EtOAc in P.E.) giving the aldol adduct **S6** as a colourless oil (3.83 g, 87%). $[\alpha]_D^{22}$ +68.4 (*c* 1.05, $CHCl_3$), [Lit., $[\alpha]_D^{28}$ +75.7 (*c* 1.05, $CHCl_3$)]; δ_H (400 MHz, $CDCl_3$) 4.46 (1H, ddd, *J* 8.5, 4.0, 3.0, NC*H*), 4.29 (1H, dd, *J* 9.0, 8.5, -OC*H*H), 4.22 (1H, dd, *J* 9.0, 3.0, -OCH*H*), 4.13 (1H, dq, *J* 6.5, 3.0, 3-H), 3.73 (1H, dq, *J* 7.0, 3.0, 2-H), 3.01 (1H, br s, OH), 2.34 (1H, qqd, *J* 7.0, 7.0, 4.0, C*H*(CH_3)$_2$), 1.24 (3H, d, *J* 7.0, 5-H_3), 1.17 (3H, d, *J* 6.5, 4-H_3), 0.91 (3H, d, *J* 7.0, CH(C*H*$_3$)$_2$), 0.87 (3H, d, *J* 7.0, CH(C*H*$_3$)$_2$); δ_C (100 MHz, $CDCl_3$) 177.8 (C-1), 153.8 (OC(O)N), 67.4 (C-3), 63.5 (OCH_2), 58.4 (NCH), 43.2 (C-2), 28.5 *C*H(CH_3)$_2$, 19.7 (C-4), 18.0 (CH(*C*H$_3$)$_2$), 14.8 (CH(*C*H$_3$)$_2$), 10.9 (C-5);

^1H and ^{13}C NMR data in accordance with the literature: N. S. Trotter, S. Takahashi, T. Nakata, *Org. Lett.* **1999**, *1*, 957

(*S*)-3-((2*S*, 3*R*)-3-(*tert*-Butyldimethylsilyloxy)-2-methylbutanoyl)-4-isopropyloxazolidin-2-one S15

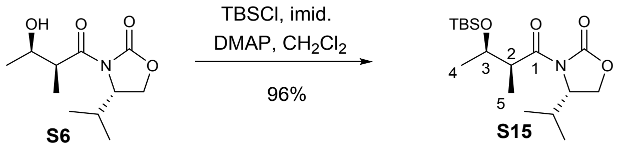

To a stirred solution of aldol adduct **S6** (2.30 g, 10.1 mmol) in dry CH_2Cl_2 (100 mL) under nitrogen, TBSCl (3.03 mg, 20.1 mmol), imidazole (2.74 g, 40.2 mmol) and 3 crystals of DMAP were added and the resulting solution stirred for 16 h. The reaction mixture was quenched with saturated $NaHCO_{3(aq)}$ solution (80 mL) and then extracted in CH_2Cl_2 (3 × 100 mL). The combined organic layers were washed with brine (80 mL), dried ($MgSO_4$), filtered and concentrated *in vacuo*. The resulting crude product was purified by flash column chromatography (2% to 5% EtOAc in P.E.) to give the protected β-hydroxy oxazolidone **S15** as a colourless oil (3.32 g, 96%). $[\alpha]_D^{22}$ +65.8 (*c* 0.76, $CHCl_3$); υ_{max} (neat) / cm^{-1} 2958 (CH), 2930 (CH), 2884 (CH), 2857 (CH), 1778 (C=O), 1697 (C=O); δ_H (400 MHz, $CDCl_3$) 4.40

(1H, dt, *J* 7.5, 3.5, -NCH), 4.25-4.18 (2H, m, -OCH_2), 4.04 (1H, quintet, *J* 6.0, 3-H), 3.83 (1H, pentet, *J* 7.0, 2-H), 2.37 (1H, qd, *J* 7.0, 4.0, C*H*$(CH_3)_2$), 1.19 (3H, d, *J* 7.0, 5-H_3) 1.15 (3H, d, *J* 6.0, 4-H_3), 0.91 (3H, d, *J* 7.0, CH(CH_3)$_2$), 0.87 (3H, d, *J* 7.0, CH(CH_3)$_2$), 0.87 (9H, s, SiC$(CH_3)_3$), 0.04 (3H, s, $SiCH_3$), 0.02 (3H, s, $SiCH_3$); δ_C (100 MHz, $CDCl_3$) 175.5 (C-1), 153.9 (NC(O)O), 69.6 (C-3), 63.3 (OCH_2), 58.9 (NCH), 45.2 (C-2), 28.6 (C$(CH_3)_2$), 25.9 (Si*C*$(CH_3)_3$) 22.0 (C-4), 18.2 (SiC(*C*H_3)$_3$), 14.8 (SiC(*C*H_3)$_3$), 13.4 (C-5), −4.3 ($SiCH_3$), −4.9 ($SiCH_3$); *m/z* (ESI) 366.21 $[M+Na]^+$; Found (ESI) 366.2071 (requires $C_{17}H_{33}O_4NSiNa$ 366.2073).

(2*S*, 3*R*)-3-(*tert*-Butyldimethylsilyloxy)-2-methylbutanoic acid S7

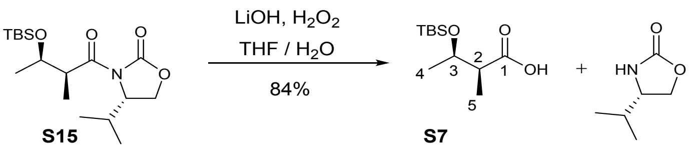

A solution of protected aldol adduct **S15** (3.25 mg, 9.46 mmol) in THF/H_2O (120 mL/40 mL) was cooled to 0 °C. H_2O_2 (30% *w/w*, 5.4 mL, 47.30 mmol) was added followed by LiOH (453 mg, 18.92 mmol). The reaction mixture was allowed to warm to room temperature and stirred overnight. Excess peracid was quenched with $Na_2SO_{3(aq)}$ (1.5 M) and THF was removed *in vacuo*. The resulting solution was acidified to pH 2 with HCl (2 M) and extracted in CH_2Cl_2 (3 × 80 mL). The combined organic extracts were washed with brine (50 mL), dried ($MgSO_4$), filtered and concentrated *in vacuo* to a give a clear oil. The crude material was purified by flash column chromatography (10% EtOAc in 90% P.E. with 0.1% CHCOOH) to give the protected β-hydroxy acid **S7** as a colourless oil (1.85 g, 84%). $[\alpha]_D^{22}$ +23.3 (*c* 0.6, $CHCl_3$); υ_{max} (neat) / cm^{-1} 2956 (CH), 2930 (CH), 2887 (CH), 2858 (CH), 1705 (C=O); δ_H (400 MHz, $CDCl_3$) 4.08 (1H, qd, *J* 6.0, 5.0, 3-H), 2.57 (1H, m, 2-H), 1.18 (3H, d, *J* 6.0, 4-H_3), 1.14 (3H, d, *J* 7.0, 5-H_3), 0.90 (9H, s, SiC$(CH_3)_3$), 0.11 (3H, s, $SiCH_3$), 0.10 (3H, s, $SiCH_3$); δ_C (100 MHz, $CDCl_3$), 177.0 (C-1), 70.3 (C-3), 46.4 (C-2), 25.8 (SiC(*C*H_3)$_3$), 20.0 (C-4), 18.1 (Si*C*$(CH_3)_3$), 12.2 (C-5), −4.4 ($SiCH_3$), −5.0 ($SiCH_3$); *m/z* (ESI) 255.14 $[M+Na]^+$; Found (ESI) 255.1387 (requires $C_{11}H_{24}O_3SiNa$ 255.1382). The chiral auxiliary was recovered using an eluent system of 80% EtOAc in petrol to give oxazolidinone as a white solid (856 mg, 70%);

^1H and ^{13}C NMR data in accordance with the literature: D. A. Evans, J. Bartroli, T. L. Shih, *J. Am. Chem. Soc.* **1981**, *103*, 2127.

(2*S*, 3*R*)-*N*-allyl-3-(*tert*-butyldimethylsilyloxy)-2-methylbutanamide S16

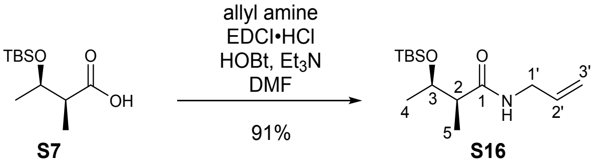

Acid **S7** (935 mg, 4.02 mmol) was dissolved in anhydrous DMF (20 mL) and cooled to 0 °C under nitrogen. Allylamine (0.60 mL, 8.05 mmol), EDCI.HCl (1.16 g, 6.03 mmol) and HOBt (109 mg, 0.805 mmol) were added followed by the dropwise addition of Et_3N (0.8 mL, 6.03 mmol). The reaction mixture was allowed to warm to room temperature and stirred for 12 h. The DMF was removed *in*

vacuo and the remaining crude yellow oil was diluted with EtOAc (20 mL). The organic layer was washed with citric acid (1.0 M, 10 mL), saturated $NaHCO_{3(aq)}$ solution (10 mL), brine (10 mL), dried ($MgSO_4$) and concentrated *in vacuo* to give a white solid. Further purification by flash column chromatography (40% EtOAc in P.E) gave amide **S16** as a white solid (992 mg, 91%). $[\alpha]_D^{23}$ +9.0 (*c* 1.0, $CHCl_3$); m.p. 94-96 °C; υ_{max} (neat) / cm^{-1} 3247, 3074, 2956 (CH), 2929 (CH), 2886 (CH), 2857 (CH), 1654 (C=O), 1631 (C=O), 1097; δ_H (400 MHz, $CDCl_3$) 6.50 (1H, br s, NH), 5.84 (1H, ddt, *J* 17.0, 10.0, 5.5, 2'-H), 5.20-5.11 (2H, m, 3'-H_2), 3.95 (1H, *overlapping* m, 1'-*H*H), 3.94 (1H, *overlapping* m, 3-H), 3.81 (1H, ddt, 15.5, 5.5, 1.5, 1'H*H*), 2.42 (1H, qd, *J* 7.0, 4.5, 2-H), 1.12 (3H, d, *J* 6.0, 4-H_3), 1.10 (3H, d, *J* 7.0, 5-H_3); δ_C (100 MHz, $CDCl_3$) 173.8 (C-1), 134.6 (C-2'), 116.3 (C-3'), 71.0 (C-3), 47.4 (C-2), 41.8 (C-1'), 25.9 (SiC(CH$_3$)$_3$), 19.4 (C-4), 18.1 (SiC(CH$_3$)$_3$), 13.3 (C-5), −4.4 ($SiCH_3$), −4.9 ($SiCH_3$); *m/z* (ESI) 294.18 $[M+Na]^+$; Found (ESI) 294.1860 ($C_{14}H_{29}O_2NSiNa$ requires 294.1849).

(2*S*, 3*R*)-3-(*tert*-Butyldimethylsilyloxy)-*N*-((*R*)-2'-hydroxypent-4'-en-1-yl)-2-methylbutanamide S17

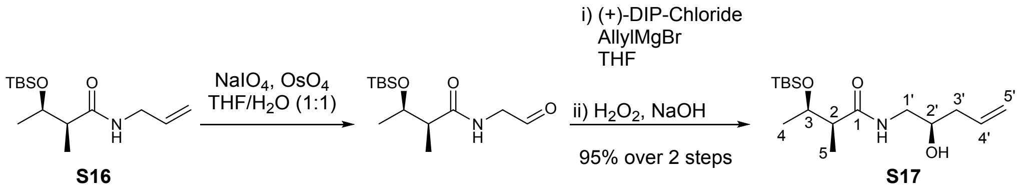

To a solution of amide **S16** (200 mg, 0.737 mmol) in THF (6 mL) and H_2O (6 mL) were added $NaIO_4$ (709 mg, 3.32 mmol) and a single crystal of OsO_4. The resulting reaction mixture was stirred for 1 h at room temperature and then diluted with H_2O (5 mL), acidified with HCl (1 M) to pH ~ 4 and extracted with EtOAc (3 × 15 mL). The combined organic layers were washed with brine (15 mL), dried ($MgSO_4$), filtered and concentrated *in vacuo* to give aldehyde as a dark yellow oil which was used in the next step without further purification. Allylmagnesium bromide (1.0 M in ether, 1.2 mL, 1.2 mmol) was added dropwise to a solution of (+)-B-chlorodiisopinocampheylborane (378 mg, 1.18 mmol) in anhydrous THF (9 mL) at −78 °C under an atmosphere of nitrogen. The solution was stirred at −78 °C for 1 h and then allowed to warm to room temperature and stirred for 1 h before re-cooling to −78 °C for the addition of aldehyde (202 mg, 0.737 mmol) in anhydrous THF (9 mL). The resulting reaction mixture was stirred at −78 °C for 2 h then allowed to warm to room temperature and stirred for 1 h. The reaction was quenched with a preformed mixture of NaOH (3 M, 0.5 mL) and H_2O_2 (30% *w/w*, 0.5 mL) and stirred for 1 h before diluting with H_2O (10 mL) and EtOAc (15 mL). The organic layer was extracted and the aqueous layer extracted with EtOAc (3 × 15 mL). The combined organic layers were washed with brine (15 mL), dried ($MgSO_4$), filtered and concentrated *in vacuo* to give a brown oil. Purification by flash column chromatography (10% to 50% EtOAc in P.E.) gave alcohol **S17** as a clear oil (190 mg, 95%). $[\alpha]_D^{19}$ +3.0 (*c* 1.0, $CHCl_3$); υ_{max} (neat) / cm^{-1} 3316 (OH), 2957 (CH), 2857 (CH), 1643 (C=O), 1540, 1462, 1254, 1102; δ_H (400 MHz, $CDCl_3$) 6.82 (1H, br s, NH), 5.81 (1H, *app* ddt, *J* 17.0, 10.0, 7.0, 4'-H), 5.15-5.09 (2H, m, 5'-H_2), 3.92 (1H, qd, *J* 6.0, 4.5, 3-H), 3.75 (1H, m, 2'-H), 3.53 (1H, ddd, *J* 14.0, 7.0, 3.0, 1'-*H*H), 3.10 (1H, ddd, *J* 14.0, 7.0, 5.0, 1'-H*H*), 2.41 (1H, qd, *J* 7.0, 4.5, 2-H), 2.24 (2H, m, 3'H_2), 1.11 (3H, d, *J* 6.0, 4-H_3), 1.09 (3H, d, *J* 7.0, 5-H_3), 0.89 (9H, s, SiC$(CH_3)_3$), 0.08 (6H, s, Si$(CH_3)_2$; δ_C (100 MHz, $CDCl_3$) 175.5 (C-1), 134.2 (C-4'), 118.2 (C-5'), 71.0 (C-3), 70.8 (C-2'), 47.5 (C-2), 45.3 (C-1'), 39.6 (C-3'), 25.9 (SiC(CH$_3$)$_3$), 19.5 (C-5), 18.1 (SiC(CH$_3$)$_3$, 13.3 (C-4),

−4.37 (SiCH$_3$), −4.89 (SiCH$_3$); *m/z* (ESI) 338.21 [M+Na]$^+$; Found (ESI) 338.2122 ($C_{16}H_{33}O_3NSiNa$ requires 338.2117).

Mosher's ester derivatives of alcohol S17

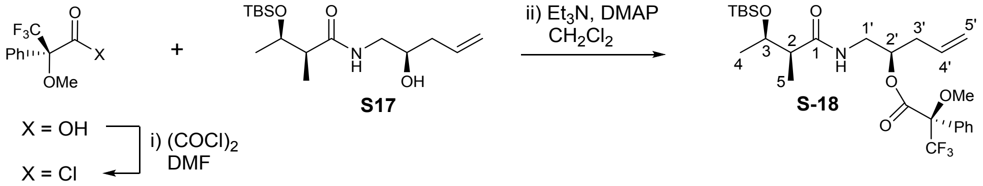

Oxalyl chloride (0.08 mL, 0.95 mmol) was added to a stirred solution of (*S*)-(-)-methyoxy-α-(trifluoromethyl)phenylacetic acid (44.0 mg, 0.19 mmol) and *N*,*N*-dimethylformamide (20 µL, 0.29 mmol) in dry hexane (10 mL) at room temperature under nitrogen. The mixture was stirred for 1 h before the hexane layer was removed from the oily Vilsmeier salt at the bottom. The hexane solution was concentrated *in vacuo* to give the acid chloride as a clear oil. The acid chloride was added to a solution of the alcohol **S17** (12 mg, 0.038 mmol), triethylamine (15.0 µL, 0.114 mmol) and DMAP (3 mg, 0.03 mmol) in dry CH_2Cl_2 (2 mL) at room temperature under nitrogen. The mixture was stirred for 2 h and then diluted with CH_2Cl_2 (10 mL). The solution was washed with water (5 mL), saturated $NaHCO_{3(aq)}$ (5 mL), dried ($MgSO_4$), filtered and concentrated *in vacuo*. The crude product was purified by flash column chromatography (5 to 50% Et_2O in P.E.) to give ester **S18** as a clear oil (15 mg, 75%). δ_H (400 MHz, CDCl$_3$) 7.55-7.52 (2H, m, Ar-H), 7.43-7.38 (3H, m, Ar-H), 6.12 (1H, br t, *J* 6.0, NH), 5.76 (1H, *app.* ddt, *J* 17.0, 10.0, 7.0, 4'-H), 5.20 (1H, *app.* dq, *J* 6.5, 4.0, 2'-H), 5.17-5.11 (2H, m, 5'-H$_2$), 3.90 (1H, qd, *J* 6.5, 5.5, 3-H), 3.58 (1H, ddd, *J* 14.5, 6.5, 4.0, 1'-*H*H), 3.53 (3H, br s, OMe), 3.34 (1H, ddd, *J* 14.5, 7.0, 6.0, 1'-H*H*), 2.46-2.42 (2H, m, 3'-H$_2$), 2.20 (1H, qd, *J* 7.0, 5.5, 2-H), 1.06 (3H, d, *J* 6.5, 4-H$_3$), 1.04 (3H, d, *J* 7.0, 5-H$_3$), 0.89 (9H, s, SiC(CH$_3$)$_3$), 0.07 (3H, s, SiCH$_3$), 0.06 (3H, s, SiCH$_3$); δ_F (282.2 MHz, CDCl$_3$) −71.36.

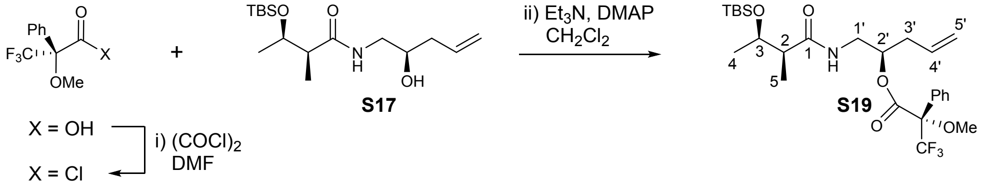

Procedure as above using (*S*)-(-)-methyoxy-α-(trifluoromethyl)phenylacetic acid to give ester **S19** (12 mg, 80%). δ_H (400 MHz, CDCl$_3$) 7.54-7.52 (2H, m, Ar-H), 7.41-7.39 (3H, m, Ar-H), 6.36 (1H, br t, *J* 5.5, NH), 5.65 (1H, *app.* ddt, *J* 17.0, 10.0, 7.0, 4'-H), 5.21 (1H, qd, *J* 6.5, 4.0, 2'-H), 5.05 (2H, m, 5-H$_2$), 3.92 (1H, qd, *J* 6.5, 5.5, 3-H), 3.57 (1H, ddd, *J* 14.5, 6.5, 4.0, 1'-*H*H), 3.52 (3H, br s, OMe), 3.50 (1H, partly obscured m, 1'-H*H*), 2.40-2.36 (2H, m, 3'-H$_2$), 2.29 (1H, qd, *J* 7.0, 5.0, 2-H), 1.26 (9H, s, SiC(CH$_3$)$_3$), 1.08 (3H, d, *J* 6.5, 4-H$_3$), 1.07 (3H, d, *J* 7.0, 5-H$_3$), each 0.07 (each 3H, each s, SiCH$_3$); δ_F (282.2 MHz, CDCl$_3$) −71.52.

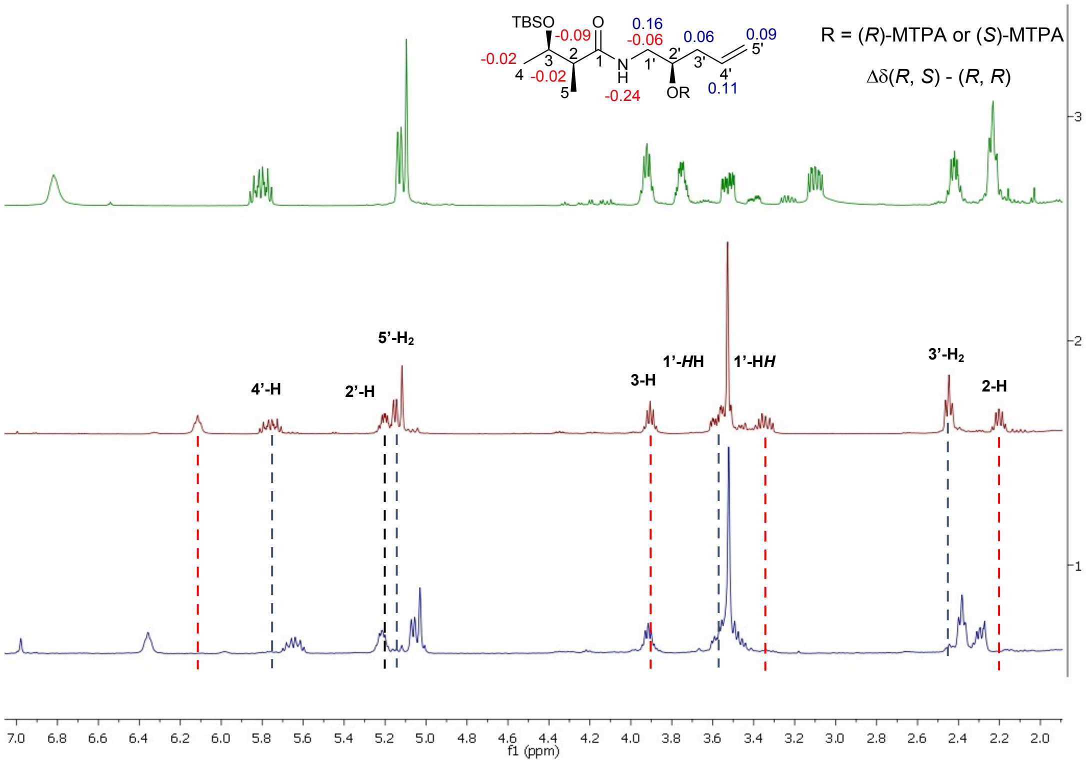

Figure S6 ^{1}H-NMR spectra for Mosher's ester derivatives **S18**, **S19** and alcohol **S17.**

(2*S*, 3*R*)-3-Hydroxy-*N*-((*R*)-2'-hydroxy-4'-oxopentyl)-2-methylbutanamide 9

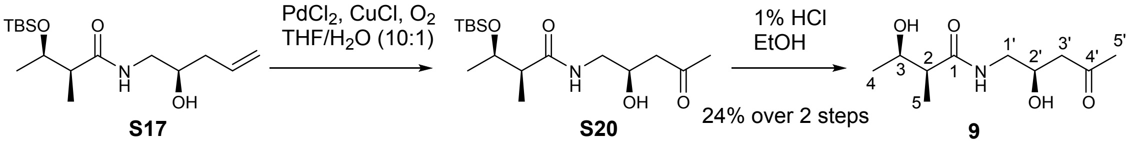

To a solution of $PdCl_2$ (14 mg, 10 mol%) in THF (17.0 mL) and water (1.8 mL) was added CuCl (77 mg, 0.773 mmol), under an atmosphere of nitrogen. The system was evacuated before installing a balloon of oxygen followed by alcohol **S17** (244 mg, 0.773 mmol) in THF (1 mL). The reaction mixture was stirred at rt for 3.5 h and then filtered through a pad of Celite® and washed through with THF (3 × 10 mL). Further purification by flash column chromatography (50% to 100% EtOAc in P.E.) gave ketone **S20** as a yellow oil. To a solution of ketone **S20** (31 mg, 0.094 mmol) in EtOH (5 mL) was added concentrated HCl until the pH ~ 2. After stirring at rt for 3 h the reaction was quenched with saturated $NaHCO_{3(aq)}$ (10 mL) and water (5 mL) and then extracted with EtOAc (5 × 30 mL). The combined organic layers were dried ($MgSO_4$), filtered and concentrated *in vacuo* to give a clear oil. Purification by preparative thin layer chromatography (SiO_2, MeOH/CH_2Cl_2, 1:10) gave alcohol **9** as a clear oil (5.1 mg, 24% over 2 steps). $[\alpha]_D^{19}$ +8.0 (*c* 1.0, $CHCl_3$); υ_{max} (neat) / cm^{-1} 3351 (OH), 2974 (CH), 2927 (CH), 1706 (C=O), 1635 (C=O), 1364, 1092; δ_H (500 MHz, acetone-d_6) 5.35 (1H, br t, *J* 5.0, NH), 4.19 (1H, d, *J* 5.0, OH), 4.10 (1H, m, 2'-H), 4.03 (1H, d, *J* 4.0, OH), 3.88 (1H, m, 3-H), 3.31 (1H, ddd, *J* 14.0, 5.5, 4.0, 1'-*H*H), 3.19 (1H, *app.* dt, 14.0, 6.0, 1'-H*H*), 2.56 (2H, m, 3'-H_2), 2.34 (1H, qd, *J* 7.0, 5.5, 2-H), 2.12 (3H, s, 5'-H_3), 1.10 (3H, d, *J* 7.0, 5-H_3), 1.09 (3H, d, *J* 6.5, 4-H_3); δ_C (125 MHz,

acetone-d_6) 206.7 (C-4'), 176.2 (C-1), 68.2 (C-3), 67.2 (C-2'), 47.8 (C-3'), 46.7 (C-2), 44.8 (C-1'), 29.6 (C-5'), 19.8 (C-4), 12.1 (C-5); *m/z* (ESI) 240.12 $[M+Na]^+$; Found (ESI) 240.1206 $[M+Na]^+$ ($C_{10}H_{19}O_4NNa$ requires 240.1209).

5f) Oxidative cleavage of diol 2 and enantioselective synthesis of lactone 10

Oxidative cleavage of diol 2

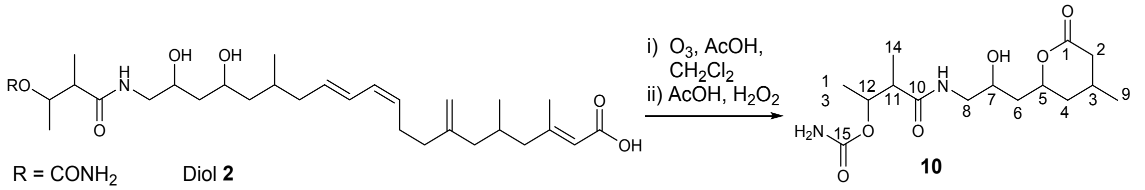

Glacial acetic acid (0.2 mL) was added to a solution of diol **2** (70 mg, 0.13 mmol) in CH_2Cl_2 (4 mL) cooled to −78 °C. Ozone was bubbled through the solution until a greyish blue colour persisted. The resultant solution was allowed to warm to room temperature and the CH_2Cl_2 was subsequently removed by a steady flow of nitrogen. The reaction mixture was taken up in glacial acetic acid (2 mL) and H_2O_2 (30% *w*/*w*, 1.2 mL) and refluxed overnight. Excess H_2O_2 was quenched with saturated $Na_2SO_{3(aq)}$ (10 mL) and the solution extracted with EtOAc (3 × 15 mL). The combined organic layers were washed with brine (10 mL), dried ($MgSO_4$), filtered and concentrated *in vacuo*. The crude extract was subjected to HPLC chromatography on a Phenomenex Luna column (250 x 4.6 mm, 5µm, 40 ºC, flow 1.5 mL/min) eluted with isocratic wash of 10% MeOH in water for 1 minute followed by a gradient of 10% to 50% MeOH in water over 17 min to yield isolated lactone **10** as a colourless oil (4.0 mg, 10%, Rt: 12.1). $[\alpha]_D^{22}$ +25.0 (*c* 0.16, MeOH); υ_{max} (neat) / cm^{-1} 3338 (OH and NH), 2966 (CH), 2932 (CH), 1705 (C=O), 1646 (C=O), 1549 (C=O), 1382, 1323, 1238; δ_H (500 MHz, methanol-d_4) 4.82 (1H, dq, *J* 8.0, 6.5, 12-H), 4.73 (1H, m, 5-H), 4.59 (1H, br s, NH), 3.92 (1H, m, 7-H), 3.29 (1H, dd, *J* 14.0, 6.0, 8-*H*H), 3.21 (1H, dd, *J* 14.0, 5.5, 8-H*H*), 2.56 (1H, dd, *J* 16.0, 5.0, 2-*H*H), 2.53 (1H, *app.* quintet, *J* 7.0, 11-H), 2.25 (1H, dd, *J* 16.0, 9.0, 2-H*H*), 2.20 (1H, m, 3-H), 1.84 (1H, *J* 14.5, 9.0, 6.5, 4-*H*H), 1.82 (1H, m, 6-*H*H), 1.67 (1H, ddd, *J* 14.5, 5.5, 4.5, 4-H*H*), 1.56 (1H, m, 6-H*H*), 1.25 (3H, d, *J* 6.5, 13-H_3), 1.13 (3H, d, *J* 7.0, 14-H_3), 1.11 (3H, d, *J* 6.5, 9-H_3); δ_C (125 MHz, methanol-d_4) 177.0 (C-10), 175.3 (C-1), 159.1 (C-15), 75.8 (C-5), 73.6 (C-12), 67.6 (C-7), 47.5 (C-11), 46.6 (C-8), 41.7 (C-6), 38.1 (C-2), 36.4 (C-4), 25.2 (C-3), 21.5 (C-9), 18.0 (C-13), 14.1 (C-14); δ_H (500 MHz, $CDCl_3$) 6.40 (1H, br t, *J* 6.5, NH), 5.11 (2H, br s, NH_2), 4.92 (1H, dq, *J* 7.5, 6.5, 12-H), 4.67 (1H, m, 5-H), 3.97 (1H, m, 7-H), 3.50 (1H, ddd, *J* 14.0, 6.5, 3.0, 8-H*H*), 3.20 (1H, ddd, *J* 14.0, 7.0, 5.5, 8-*H*H), 2.57 (1H, dd, *J* 20.0, 9.0, 2-*H*H), 2.54 (1H, *app.* quintet, *J* 7.0, 11-H), 2.19 (1H, partly *obs*. m, 3-H), 2.18 (1H, *obs*. m, 2-H*H*), 1.81 (1H, m, 4-*H*H), 1.75(1H, m, 6-*H*H), 1.65 (1H, m, 6-H*H*), 1.61 (1H, m, 4-H*H*), 1.28 (3H, d, *J* 6.5, 13-H_3), 1.17 (3H, d, *J* 7.0, 14-H_3), 1.10 (3H, d, *J* 6.5, 9-H_3); δ_C (100 MHz, $CDCl_3$) 175.4 (C-10), 173.0 (C-1), 156.9 (C-15), 74.4 (C-5), 73.1 (C-12), 68.2 (C-7), 47.5 (C-11), 46.1 (C-8), 40.3 (C-6), 37.5 (C-2), 35.6 (C-4), 24.1 (C-3), 21.6 (C-9), 18.31 (C-13), 14.2 (C-14); *m/z* (ESI+) 353.1691 $[M+Na]^+$; Found (ESI) 353.1683 (requires $C_{15}H_{26}O_6N_2Na$ 353.1691).

(1*R*, 2*S*)-*N*-[(3'*S*)-methylhex-5'-enyl]-bornane-10,2-sultam 14

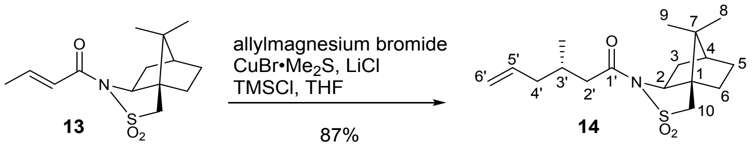

CuBr·Me_2S (6.64 g, 32.3 mmol) and LiCl (1.37 g, 32.3 mmol) in dry THF (50 mL) were pre-stirred for 0.5 h at room temperature before adding a solution of allylmagnesium bromide (1.0 M in Et_2O, 30.1 mL, 30.1 mmol) in dry THF (30 mL) at -78 °C under nitrogen.[a] TMSCl (4.1 mL, 32.3 mmol) was added immediately followed by the addition of crotonyl sultam **13** (6.10 g, 21.5 mmol) in dry THF (40 mL). The reaction mixture was stirred at −78 °C for 4 h before quenching with NH_4Cl/NH_4OH (pH 8, 36 mL) and warming to room temperature overnight. H_2O (40 mL) and Et_2O (40 mL) were added and the layers separated. The aqueous phase was extracted with Et_2O (3 × 50 mL) and the combined organic layers washed with water (2 × 50 mL), brine (50 mL), dried ($MgSO_4$), filtered and concentrated *in vacuo*. Purification by column chromatography (2-5% EtOAc in P.E.) gave alkene **14** as a white solid and as a single diastereomer (6.10 g, 87%). m.p. 74-75 °C; $[\alpha]_D^{21}$ +82.0 (*c* 1.0, $CHCl_3$), [for enantiomer Lit.[b] $[\alpha]_D$ −75.3 (*c* 3.4, $CHCl_3$)]; υ_{max} (neat) / cm^{-1} 3013 (CH), 2961 (CH), 2929 (CH), 2876 (CH), 1683 (C=O), 1386, 1311; δ_H (400 MHz, $CDCl_3$) 5.75 (1H, m, 5'-H), 5.01 (2H, m, 6'-H_2), 3.86 (1H, *app.* t, *J* 6.5, 2-H), 3.47 (1H, d, *J* 14.0, 10-*H*H), 3.41 (1H, d, *J* 14.0, 10-H*H*), 2.75 (1H, dd, *J* 16.0, 6.0, 2'*H*H), 2.47 (1H, dd, *J* 16.0, 7.5, 2'-H*H*), 2.21 (1H, *app.* octet, *J* 6.5, 3'-H), 2.12-1.97 (4H, m, 3-H_2, 4'-H_2), 1.94-1.84 (3H, m, 4-H, 5-*H*H, 6-*H*H), 1.43-1.29 (2H, m, 5-H*H*, 6-H*H*), 1.14 (3H, s, CH_3), 0.96 (3H, br s, 3'-CH_3), 0.94 (3H, s, CH_3); δ_C (100 MHz, $CDCl_3$) 171.6 (C-1'), 136.6 (C-5'), 116.7 (C-6'), 65.4 (C-2), 53.2 (C-10), 48.4 (C-1), 47.9 (C-7), 42.2 (C-2'), 41.1 (C-4'), 38.7 (C-3), 33.0 (C-6), 29.8 (C-3'), 26.6 (C-5), 20.9 (CH_3), 20.0 (CH_3), 19.7 (CH_3); *m/z* (ESI) 326.18 $[M+H]^+$; Found (ESI) 326.1789 ($C_{17}H_{27}ON_3S$ requires 326.1784).

a) B. Lipshutz, C. Hackmann B. H, *J. Org. Chem.* **1994**, *59*, 7437; T. Serge, T. Synthesis, L. A. Paquette, *Synthesis* **2002**, *2002*, 2–7
b) E. Owusu-Ansah, A. C. Durow, J. R. Harding, A. C. Jordan, S. J. O'Connell, C. L. Willis, *Org. Biomol. Chem.* **2011**, *9*, 265.

(1*R*, 2*S*)-*N*-[(3'*S*)-methylhexan-5'-ol]-bornane-10,2-sultam 15

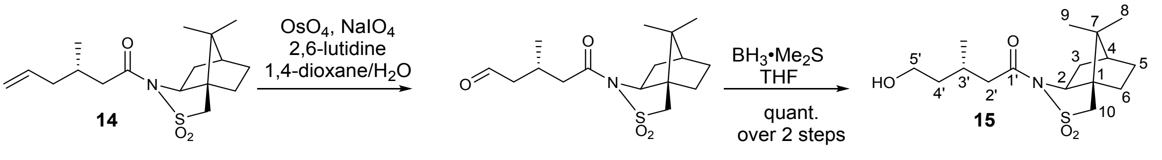

Osmium tetroxide (1 crystal) was added to a solution of alkene **14** (4.70 g, 14.4 mmol) and 2,6-lutidine (3.40 mL, 28.9 mmol) in 1,4-dioxane (160 mL) and H_2O (130 mL) at room temperature. $NaOI_4$ (12.35 g, 57.76 mmol) was added and the resulting suspension stirred at room temperature for 2 h.

The mixture was diluted with H_2O (50 mL) and then extracted with CH_2Cl_2 (3 × 100 mL). The combined organic phases were dried ($MgSO_4$), filtered and concentrated *in vacuo* to afford a black oil, which was used directly in the next step without further purification. Borane-dimethyl sulfide complex (2.0 M in THF, 7.9 mL, 15.9 mmol) was added dropwise over 5 min to a solution of the crude aldehyde in dry THF (160 mL) at 0 °C under nitrogen. The reaction mixture was stirred for 20 min at 0 °C and then quenched carefully with saturated NH_4Cl solution (100 mL) and extracted with EtOAc (3 × 100 mL). The combined organics were washed with brine (100 mL), dried ($MgSO_4$) and concentrated *in vacuo* to give a black oil. The crude product was purified by column chromatography (10 to 20% EtOAc in P.E.) to afford alcohol **15** as a colourless oil (4.74 g, quant.). $[\alpha]_D^{21}$ +77.0 (*c* 1.0, $CHCl_3$); υ_{max} (neat) / cm^{-1} 3493 (OH), 2963 (CH), 2925 (CH), 2879 (CH), 1692 (C=O), 1456, 1325, 1132; δ_H (400 MHz, $CDCl_3$) 3.88 (1H, *app.* t, *J* 6.5, 2-H), 3.65 (2H, dt, *J* 6.5, 6.5, 5'-H_2), 3.49 (1H, d, *J* 14.0, 10-*H*H), 3.43 (1H, d, *J* 14.0, 10-H*H*), 2.83 (1H, dd, *J* 16.0, 6.5, 2'-*H*H), 2.46 (1H, dd, *J* 16.0, 7.0, 2'-H*H*), 2.30 (1H, *app.* octet, *J* 7.0, 3'-H), 2.11-2.06 (2H, m, 3-H_2), 1.96 (4H, *overlapping* m, 4-H. 5-*H*H. 6-*H*H), 1.59 (1H, m, 4'-*H*H), 1.48 (1H, m, 4'-H*H*), 1.44-1.30 (2H, m, 5-H*H*, 6-H*H*), 1.15 (3H, s, 8 or 9-H_3), 1.00 (3H, d, *J* 7.0, 3'-CH_3), 0.97 (3H, s, 8 or 9-H_3); δ_C (100 MHz, $CDCl_3$) 171.8 (C-1'), 65.4 (C-2), 60.4 (C-5'), 53.2 (C-10), 48.5 (C-1), 47.9 (C-7), 44.8 (C-4), 42.5 (C-2'), 39.9 (C-4'), 38.7 (C-3), 33.0 (C-6), 26.6 (C-3'), 26.6 (C-5), 21.0 (CH_3), 20.5 (CH_3), 20.0 (CH_3); *m/z* (ESI) 330.17 $[M+H]^+$; Found (ESI) 330.1736 ($C_{16}H_{28}NO_4S$ requires 330.1734).

(1*R*, 2*S*)-*N*-[(3'*R*)-5'-*tert*-Butyldimethylsilyloxy-3'-methylhexan-5'-ol]-bornane-10,2-sultam S21

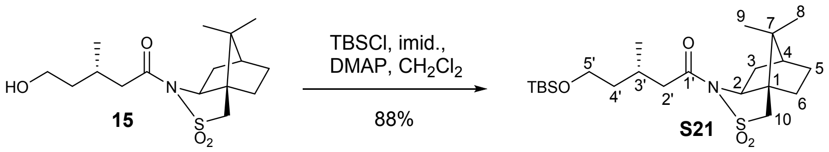

DMAP (160 mg, 1.33 mmol) was added to a stirred solution of alcohol **15** (4.39 g, 13.3 mmol), TBSCl (4.02 g, 26.7 mmol) and imidazole (1.81 g, 26.7 mmol) in dry CH_2Cl_2 (33 mL) under an atmosphere of nitrogen. After 18 h stirring at room temperature the reaction was quenched with saturated $NH_4Cl_{(aq)}$ (30 mL) and extracted in CH_2Cl_2 (3 × 30 mL). The combined organic layers were washed with brine (30 mL), dried ($MgSO_4$), filtered and concentrated *in vacuo* to give a yellow oil. Further purification by column chromatography (10 to 20% EtOAc in P.E.) gave silyl ether **S21** as a white solid (5.08 g, 88%). m.p. 72-74 °C; $[\alpha]_D^{21}$ + 57.0 (*c* 1.0, $CHCl_3$); υ_{max} (neat) / cm^{-1} 2947 (CH), 2927 (CH), 2885 (CH), 2858 (CH), 1674 (C=O), 1334, 1063; δ_H (400 MHz, $CDCl_3$). 3.86 (1H, *app.* t, *J* 6.5, 2-H), 3.64 (2H, m, 5'H_2), 3.47 (1H, d, *J* 14.0, 10-*H*H), 3.41 (1H, d, *J* 14.0, 10-H*H*), 2.22 (1H, *app.* oct, *J* 6.5, 3'-H), 2.13-2.03 (2H, m, 3-H_2), 1.95-1.83 (3H, m, 4-H, 5-*H*H, 6-*H*H), 1.59 (1H, m, 4'-*H*H), 1.47-1.30 (3H, m, 4'-H*H*, 5-H*H*, 6-H*H*), 1.14 (3H, s, CH_3), 0.97 (3H, d, *J* 6.5, 3'-CH_3), 0.96 (3H, s, CH_3), 0.87 (9H, s, $SiC(CH_3)_3$), 0.03 (6H, s, 2 × $SiCH_3$); δ_C (100 MHz, $CDCl_3$) 171.5 (C-1'), 65.3 (C-2), 61.3 (C-5'), 53.2 (C-10), 48.4 (C-7), 47.9 (C-1), 44.8 (C-4), 43.0 (C-2'), 39.5 (C-4'), 38.7 (C-3), 33.0 (C-6), 27.2 (C-3'), 26.6 (C-5), 26.1 ($SiC(CH_3)_3$), 21.0 (CH_3), 20.0 (3-CH_3), 19.8 (CH_3), 18.4 (SiC), −5.2 (2 × $SiCH_3$); *m/z* (ESI) 444.26 $[M+H]^+$; Found (ESI) 444.2592 ($C_{22}H_{42}NO_4SSi$ requires 444.2598).

(4*R*, 6*S*)-8-(*tert*-Butyldimethylsilyloxy)-6-methyloct-1-en-4-ol 16

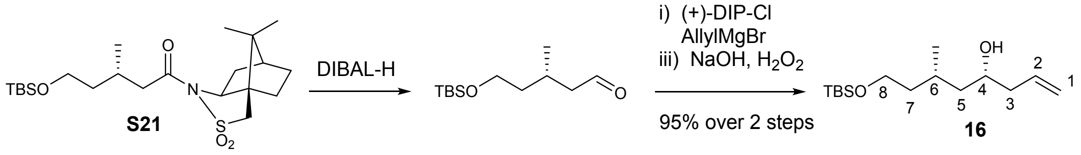

TBS ether **S21** (3.33 g, 7.50 mmol) was dissolved in dry CH_2Cl_2 (50 mL) and cooled to –78 °C under an atmosphere of nitrogen. Di*iso*butylaluminium hydride (1.0 M, 11.25 mL, 11.25 mmol) was added dropwise over 30 min. After stirring for 1 h at –78 °C, the reaction was quenched with MeOH (1 mL) and poured into a saturated aqueous solution of Rochelle's salt (50 mL) and then stirred vigorously until two clear layers formed. The aqueous phase was extracted with CH_2Cl_2 (3 × 50 mL) and then the organic phases were combined, dried ($MgSO_4$), filtered and concentrated *in vacuo*. The residue was triturated with petrol and the auxiliary was filtered off to leave aldehyde as a clear liquid (1.73 g, quant.). Aldehyde was used in the next step without further purification. Allylmagnesium bromide (1.0 M in ether, 12.0 mL, 12.0 mmol) was added dropwise to a solution of (+)-B-chlorodi*iso*pinocampheylborane (3.85 g, 12.0 mmol) in dry THF (60 mL) cooled to −78 °C under an atmosphere nitrogen. The reaction mixture was stirred for 1 h at –78 °C before being allowed to warm to room temperature over 1 h. The solution was re-cooled to –78 °C and then treated dropwise with a solution of aldehyde (1.61 g, 7.50 mmol) in dry THF (25 mL). The reaction was stirred at –78 °C for 2 h before being allowed to warm to room temperature over 1 h. The reaction was cooled to 0 °C and then quenched by the addition of a preformed mixture of an $NaOH_{(aq)}$ solution (3 M, 10.0 mL) and hydrogen peroxide (30% *w*/*w*, 10.0 mL) The mixture was allowed to warm to room temperature and stirred for 2 h before the addition of EtOAc (60 mL) and H_2O (60 mL). The layers were separated and the aqueous phase extracted with EtOAc (4 × 50 mL). The combined organic extracts were washed with water (60 mL), brine (40 mL), dried ($MgSO_4$), filtered and concentrated *in vacuo*. The crude product was purified by column chromatography (1 to 3 % EtOAc in P.E.) to give alcohol **16** as a colourless oil (1.39 g, 95% over 2 steps). $[\alpha]_D^{21}$ −6.0 (*c* 1.0, $CHCl_3$); υ_{max} (neat) / cm^{-1} 3358 (OH), 2955 (CH), 2928 (CH), 2858 (CH), 1641 (C=C), 1472, 1254, 1092; δ_H (400 MHz, $CDCl_3$) 5.83 (1H, m, 2-H), 5.11 (2H, m, 1-H_2), 3.77 (1H, m, 4-H), 3.64 (2H, m, 8-H_2), 2.26 (1H, m, 3-*H*H), 2.12 (1H, m, 3-H*H*), 1.89 (1H, br m, OH), 1.76 (1H, m, 6-H), 1.62 (1H, m, 7-*H*H), 1.38 (2H, m, 5-H_2) 1.32 (1H, m, 7-H*H*), 0.93 (3H, d, *J* 6.5, 6-CH_3) 0.89 (9H, s, $SiC(CH_3)_3$), 0.05 (6H, s, 2 × $SiCH_3$); δ_C (100 MHz, $CDCl_3$) 135.1 (C-1), 118.1 (C-2), 68.7 (C-4), 61.6 (C-8), 44.4 (C-5), 42.2 (C-3), 39.4 (C-7), 26.6 (C-6), 26.1 ($SiC(CH_3)_3$), 20.8 (6-CH_3), 18.5 (SiC), −5.1 ($SiCH_3$), −5.2 ($SiCH_3$); *m/z* (ESI) 273.22 $[M+H]^+$; Found (ESI) 273.2240 ($C_{15}H_{33}O_2Si$ requires 273.2244).

Mosher's ester derivatives of alcohol 16

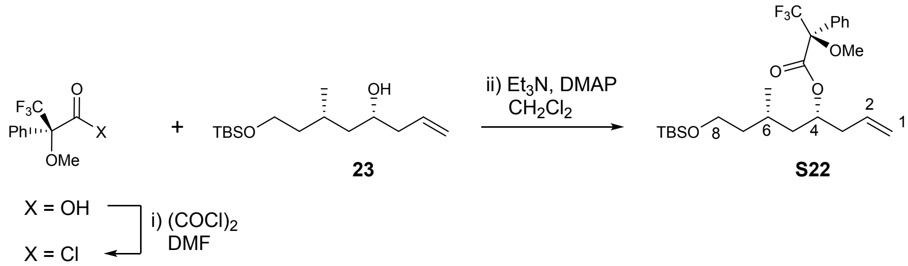

Oxalyl chloride (0.20 mL, 2.75 mmol) was added to a stirred solution of (*S*)-(−)-methyoxy-α-(trifluoromethyl)phenylacetic acid (129 mg, 0.550 mmol) and *N*,*N*-dimethylformamide (60 µL, 0.83 mmol) in dry hexane (10 mL) at room temperature under nitrogen. The mixture was stirred for 1 h before the hexane layer was removed from the oily Vilsmeier salt at the bottom. The hexane solution was concentrated *in vacuo* to give the acid chloride as a clear oil. The acid chloride was added to a solution of the alcohol **16** (30 mg, 0.11 mmol), triethylamine (0.5 mL, 3.3 mmol) and DMAP (10 mg, 0.08 mmol) in dry CH_2Cl_2 (5 mL) at room temperature under nitrogen. The mixture was stirred for 2 h and then diluted with CH_2Cl_2 (10 mL). The solution was washed with water (5 mL), saturated $NaHCO_{3(aq)}$ (5 mL), dried ($MgSO_4$), filtered and concentrated *in vacuo*. The crude product was purified by flash column chromatography (5 to 50% Et_2O in P.E.) to give ester **S22** as a clear oil (50 mg, 86%). δ_H (400 MHz, $CDCl_3$) 7.56-7.52 (2H, m, Ar-H), 7.42-7.37 (3H, m, Ar-H), 5.77 (1H, m, 2-H), 5.24 (1H, *app*. quintet, 4-H), 5.10 (2H, m, 1-H_2), 3.64-3.56 (2H, m, 8-H_2), 3.55 (3H, br s, OMe), 2.49-2.32 (2H, m, 3-H_2), 1.57-1.45 (5H, m, 5-H_2, 6-H, 7-H_2), 0.89 (9H, s, $SiC(CH_3)_3$), 0.86 (3H, d, *J* 6.5, 6-CH_3), 0.04 (6H, s, 2 × $SiCH_3$); δ_F (282.2 MHz, $CDCl_3$) −71.21.

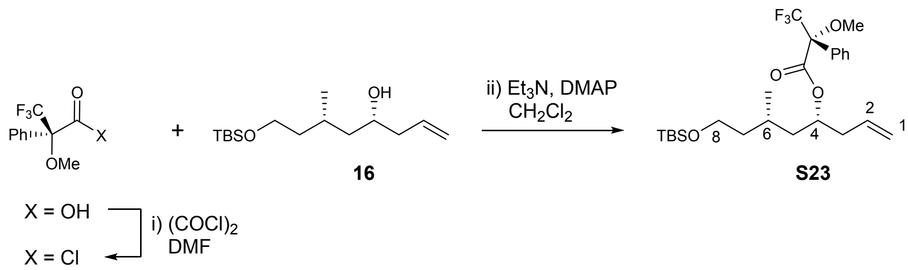

The above procedure was repeated using (*R*)-(−)-methyoxy-α-(trifluoromethyl)phenylacetic acid to give ester **S23** (48 mg, 83%). δ_H (400 MHz, $CDCl_3$) 7.56-7.52 (2H, m, Ar-H), 7.42-7.37 (3H, m, Ar-H), 5.62 (1H, m, 2-H), 5.23 (1H, *app*. quintet, 4-H), 5.00 (2H, m, 1-H_2), 3.67-3.58 (2H, m, 8-H_2), 3.54 (3H, br s, OMe), 2.44-2.26 (2H, m, 3-H_2), 1.69-1.49 (5H, m, 5-H_2, 6-H, 7-H_2), 0.93 (3H, d, *J* 6.5, 6-CH_3), 0.88 (9H, s, $SiC(CH_3)_3$), 0.04 (6H, s, 2 × $SiCH_3$); δ_F (282.2 MHz, $CDCl_3$) −71.31.

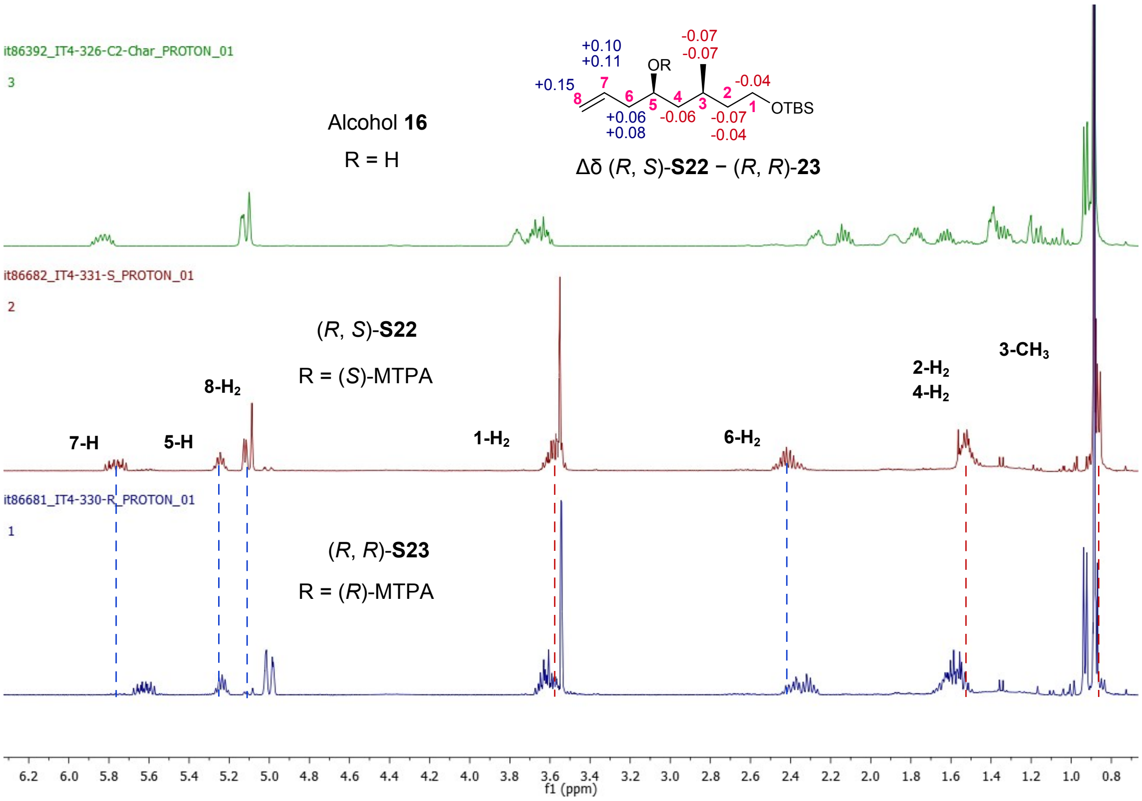

Figure S7. ^{1}H- NMR comparison of Mosher's ester derivatives **S22**, **S23** and alcohol **23**

(4*S*, 6*S*)-4,8-Di(*tert*-butyldimethylsilyloxy)-6-methyl-oct-1-ene S24

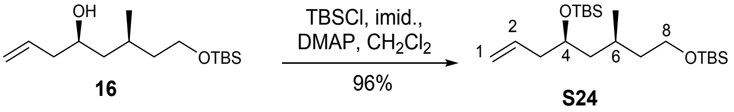

Alcohol **16** (2.80 g, 10.28 mmol) was dissolved in CH_2Cl_2 (25 mL) at room temperature under an atmosphere of nitrogen. Imidazole (1.40 g, 20.6 mmol), TBSCl (2.30 g, 15.4 mmol) and DMAP (126 mg, 1.03 mmol) were added and the resulting reaction mixture stirred for 18 h before the reaction was quenched with saturated $NH_4Cl_{(aq)}$ solution (25 mL) and the aqueous layer extracted with CH_2Cl_2 (3 × 25 mL). The combined organic layers were dried ($MgSO_4$), filtered and concentrated *in vacuo* to give a crude clear oil. Purification by column chromatography (5% EtOAc in P.E.) gave protected alcohol S24 as a clear liquid (3.82 g, 96%). $[\alpha]_D^{21}$ − 8.0 (*c* 1.0, $CHCl_3$); υ_{max} (neat) / cm^{-1} 2955 (CH), 2929 (CH), 2857 (CH), 1473, 1462, 1253; δ_H (400 MHz, $CDCl_3$) 5.82 (1H, m, 2-H), 5.04 (1H, d, *J* 15.0, 1-*H*H), 5.03 (1H, d, *J* 12.0, 1-H*H*), 3.79 (1H, *app.* quintet, *J* 6.0, 4-H), 3.68-3.58 (2H, m, 8-H_2), 2.24 (1H, m, 3-*H*H), 2.17 (1H, m, 3-H*H*), 1.69-1.54 (2H, m, 6-H, 7-*H*H), 1.43 (1H, m, 5-*H*H), 1.30 (1H, m, 7-H*H*), 1.28 (1H, m, 5-H*H*), 0.90-0.88 (21H, m, 6-CH_3, 2 × $SiC(CH_3)_3$), 0.05 (6H, s, 2 × $SiCH_3$), 0.04 (6H, s, 2 × $SiCH_3$); δ_C (100 MHz, $CDCl_3$) 135.5 (C-2), 116.8 (C-1), 70.3 (C-4), 61.4 (C-8), 44.9 (C-5), 42.0 (C-3), 40.3 (C-7), 26.4 (C-6), 26.2 ($SiC(CH_3)_3$), 26.1 ($SiC(CH_3)_3$), 20.4 (6-CH_3), 18.5 ($SiC(CH_3)_3$), 18.3 ($SiC(CH_3)_3$), −4.1 ($SiCH_3$), −4.3 ($SiCH_3$), −5.1 ($SiCH_3$), −5.1 ($SiCH_3$); *m/z* (ESI) 387.31; Found 387.3105 (ESI) ($C_{21}H_{47}O_2Si_2$ requires 387.3109).

(2*R*, 4*S*, 6*S*)-4,8-Di*(tert*-butyldimethylsilyloxy)-1,2-epoxy-6-methyloctane 17 and (2*S*, 4*S*, 6*S*)-4,8-di*(tert*-butyldimethylsilyloxy)-1,2-epoxy-6-methyloctane 18

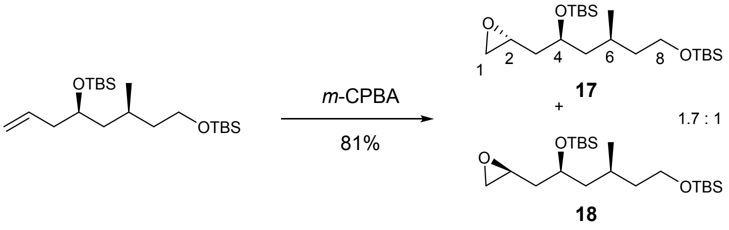

Alkene **S24** (6.04 mg, 15.6 mmol) was dissolved in dry CH_2Cl_2 (40 mL) and cooled to 0 °C under an atmosphere of nitrogen. *m*-CPBA (3.23 g, 18.7 mmol) was added and the reaction mixture stirred at 0 °C for 4 h and then 1 h at room temperature. The reaction was diluted with EtOAc (100 mL) and washing consecutively with a saturated $Na_2SO_{3(aq)}$ solution (60 mL), a saturated $NaHCO_{3(aq)}$ solution (60 mL) and then brine (60 mL). The organic phase was dried (Na_2SO_4), filtered and concentrated *in vacuo* to give a white oily residue. The crude material was purified by column chromatography (1 to 2% EtOAc in P.E.) to give an inseparable mixture (1.7:1) of epoxides **17** and **18** as a colourless liquid (5.11 g, 81%). No $[\alpha]_D$ was recorded as the product contains a mixture of diastereomers; due to overlapping signals in the ^1H NMR spectra, the reported spectral data is for the mixture of diastereomers **17** and **18**, unless otherwise stated; υ_{max} (neat) / cm^{-1} 2954 (CH), 2928 (CH), 2857 (CH), 1472, 1463, 1254; δ_H (400 MHz, $CDCl_3$) 3.98-3.91 (1H, m, 4-H), 3.68-3.58 (2H, m, 8-H_2) 3.08-3.02 (1H, m, 2-H), 2.79 (0.63H, *app.* t, *J* 4.5, epoxide **17** 1-*H*H), 2.75 (0.37H, *app.* t, *J* 4.5, epoxide **18** 1-*H*H), 2.49 (0.63H, dd, *J* 5.0, 3.0, epoxide **17** 1-H*H*), 2.44 (0.37H, dd, *J* 5.0, 3.0, epoxide **18** 1-H*H*), 1.74-1.46 (5H, m, 3-H_2, 5-*H*H, 6-H, 7-*H*H), 1.42-1.25 (2H, m, 5-H*H*, 7-H*H*), 0.90-0.88 (21H, m, 6-CH_3, 2 × $SiC(CH_3)_3$), 0.08-0.04 (12H, m, 4 × $SiCH_3$); δ_C (100 MHz, $CDCl_3$) 68.9, 68.6 (C-4), 61.4, 61.2 (C-8), 50.0, 49.6 (C-2), 48.0, 46.9 (C-1), 45.9, 45.3 (C-5), 40.7, 40.3, 40.3, 40.2 (C-3, C-7), 26.5, 26.4 (C-6), 26.2, 26.1 ($SiC(CH_3)_3$), 26.0, 26.0 ($SiC(CH_3)_3$), 20.3, 20.1 (6-CH_3), 18.5, 18.2 ($Si\mathit{C}(CH_3)_3$), −4.16, −4.24 ($SiCH_3$), −4.32, −4.45 ($SiCH_3$), −5.11, −5.14 ($SiCH_3$); *m/z* (ESI) 403.31 $[M+H]^+$; Found (ESI) 403.3058 ($C_{21}H_{47}O_3Si_2$ requires 403.3058).

(2*R*, 4*S*, 6*S*)-1-Azido-4,8-di*(tert*-butyldimethylsilyloxy)-6-methyloctan-2-ol 20

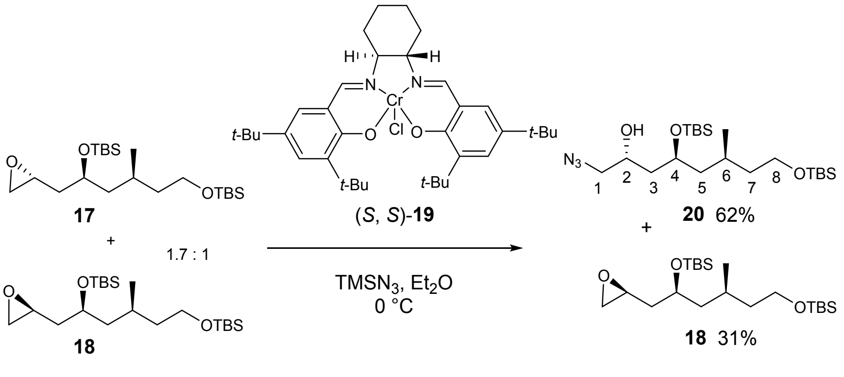

A mixture of epoxides **17** and **18** (1.25 g, 3.10 mmol) was dissolved in Et_2O and cooled to 0 °C under an atmosphere of nitrogen. The catalyst (*S*, *S*)-**I** (2 mol%)[a] was added and stirred for 10 min at 0 °C and then $TMSN_3$ (220 µL, 1.71 mmol) was added and the reaction stirred for 120-168 h at 0-4 °C in a thermostatted isopropanol bath. After complete consumption of epoxide **17** (determined by ^1H NMR

analysis of an aliquot of the crude reaction mixture) the solvent was removed *in vacuo* from a 0 °C ice bath, and the crude reaction mixture immediately purified by column chromatography (1 to 2% EtOAc in P.E.) to give azide **20** as a yellow oil (0.99 g, 62%) and epoxide **18** as a yellow oil (0.40 g, 32%). Data for azide **20**; $[\alpha]^{21}_{D}$ +9.0 (*c* 1.0, $CHCl_3$); υ_{max} (neat) / cm^{-1} 3454 (OH), 2954 (CH), 2929 (CH), 2857 (CH), 2101 (N=N=N), 1472, 1253, 1094; δ_H (400 MHz, $CDCl_3$) 4.16-4.09 (2H, m, 2-H, 4-H), 3.76 (1H, br s, OH), 3.64 (2H, m, 8-H_2), 3.27 (1H, dd, *J* 12.5, 4.0, 1-*H*H), 3.21 (1H, dd, *J* 12.5, 6.0, 1-H*H*), 1.76 (1H, ddd, *J* 14.5, 10.5, 3.5, 3-*H*H), 1.66 (1H, ddd, *J* 13.5, 9.0, 4.0, 5-*H*H), 1.58-1.47 (3H, m, 3-H*H*, 6-H, 7-*H*H), 1.42-1.31 (2H, m, 5-H*H*, 7-H*H*), 0.89-0.87 (19H, *overlapping* m, 6-CH_3, 2 × $SiC(CH_3)_3$), 0.11 (3H, s, $SiCH_3$), 0.09 (3H, s, $SiCH_3$), 0.04 (6H, s, $Si(CH_3)_2$); δ_C (100 MHz, $CDCl_3$) 69.8 (C-4), 67.9 (C-2), 60.9 (C-8), 57.2 (C-1), 43.4 (C-5), 40.8 (C-7), 37.8 (C-3), 26.5 (C-6), 26.1 ($SiC(CH_3)_3$), 25.9 ($SiC(CH_3)_3$), 19.7 (CH_3), 18.4 ($SiC(CH_3)_3$), 18.1 ($SiC(CH_3)_3$), −4.4 ($SiCH_3$), −4.6 ($SiCH_3$), −5.2 ($SiCH_3$), −5.1 ($SiCH_3$); *m/z* (ESI) 468.30 $[M+Na]^+$; Found (ESI) 468.3048 ($C_{21}H_{47}N_3O_3Si_2Na$ requires 468.3054). Data for epoxide **18**; $[\alpha]^{21}_{D}$ + 0.0 (*c* 1.0, $CHCl_3$); υ_{max} (neat) / cm^{-1} 2954 (CH), 2928 (CH), 2857 (CH), 1472, 1463, 1254; δ_H (400 MHz, $CDCl_3$) 3.94 (1H, *app.* quintet, *J* 6.0, 4-H), 3.69-3.58 (2H, m, 8-H_2), 3.08-3.03 (1H, m, 2-H), 2.74 (1H, *app.* t, *J* 4.5, 1-*H*H), 2.44 (1H, dd, *J* 5.0, 2.5, 1-H*H*), 1.74-1.50 (5H, m, 3-H_2, 5-*H*H, 6-H, 7-*H*H), 1.42-1.24 (2H, m, 5-H*H*, 7-H*H*), 0.90-0.89 (21H, m, 6-CH_3, 2 × $SiC(CH_3)_3$), 0.06 ($SiCH_3$), 0.05 ($SiCH_3$), 0.04 (2 × $SiCH_3$); δ_C (100 MHz, $CDCl_3$) 68.8 (C-4), 61.3 (C-8), 49.6 (C-2), 46.9 (C-1), 45.3 (C-5), 40.3, 40.2 (C-3, C-7), 26.5 (C-6), 26.1 ($SiC(CH_3)_3$), 26.0($SiC(CH_3)_3$), 20.3 (6-CH_3), 18.5 ($SiC(CH_3)_3$), 18.2 ($SiC(CH_3)_3$), −4.2 ($SiCH_3$), −4.3 ($SiCH_3$), −5.1 ($SiCH_3$), −5.2 ($SiCH_3$); *m/z* (ESI) 425.29 $[M+H]^+$; Found (ESI) 425.2881 ($C_{21}H_{47}O_3Si_2Na$ requires 425.2878).

a) L. E. Martinez, J. L. Leighton, D. H. Carsten, E. N. Jacobsen, *J. Am. Chem. Soc.* **1995**, *117*, 5897–5898.

2,4-Acetonide of (2*R*, 4*S*, 6*S*)-1-azido-4,8-di(*tert*-butyldimethylsilyloxy)-6-methyloctan-2-ol 21

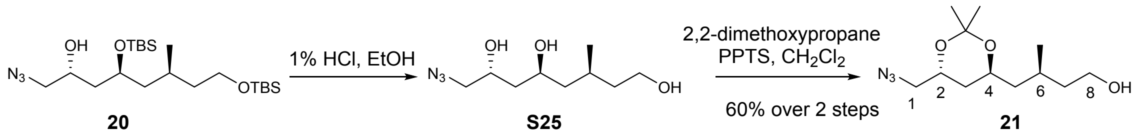

20 **S25** **21**

Concentrated HCl (1%, 12 M, 50 µL) was added to a solution of azide **20** (93 mg, 209 mmol) in EtOH (5 mL) and stirred at room temperature for 3 h. The reaction mixture was quenched with saturated $NaHCO_{3(aq)}$ solution (10 mL) and extracted with EtOAc (3 × 20 mL), the combed organic layers were washed with brine (20 mL), dried ($MgSO_4$), filtered and concentrated *in vacuo* to give triol S25 as a yellow oil which was used in the next step without further purification. Azide **S25** (45 mg, 0.21 mmol) was dissolved in dry CH_2Cl_2 (1 mL) and 2,2-dimethoxypropane (2 mL) was added followed by 2 crystals of pyridinium *p*-toluenesulfonate. After 3 h the reaction was diluted with CH_2Cl_2 (10 mL) and water (10 mL). The organic layer was extracted and the aqueous phase subsequently extracted with CH_2Cl_2 (2 × 10 mL). The organic fractions were combined, dried ($MgSO_4$), filtered and concentrated *in vacuo*. The crude material was purified by column chromatography (20 to 50% EtOAc in P.E.) to give acetonide **21** as a colourless oil (32 mg, 60%). $[\alpha]^{21}_{D}$ −2.0 (*c* 1.0, $CHCl_3$); υ_{max} (neat) / cm^{-1} 3326 (OH), 2926 (CH), 2096 (N=N), 1439, 1276, 1054; δ_H (400 MHz, $CDCl_3$) 4.03 (1H, m, 2-H), 3.93 (1H, m, 4-H), 3.67 (2H, m, 8-H_2), 3.25 (1H, dd, *J* 13.0, 7.5 ,1-*H*H), 3.18 (1H, dd, *J* 13.0, 3.5, 1-H*H*), 2.03 (1H, br s, OH), 1.75 (1H, m, 6-H), 1.69-1.39 (6H, m, 3-H_2, 5-H_2, 7-H_2), 1.37 (6H, s, $C(CH_3)_2$), 0.93 (3H, d, *J* 6.5, 6-

CH_3); δ_C (100 MHz, $CDCl_3$) 100.9 (*C*(CH_3)$_2$), 66.5 (C-2), 64.9 (C-4), 61.0 (C-8), 54.4 (C-1), 42.9 (C-5), 39.6 (C-7), 35.7 (C-3), 26.4 (C-6), 24.8 (C(*C*H_3)), 24.7 (C(*C*H_3)), 20.3 (CH_3); *m/z* (ESI) 280.16 $[M+Na]^+$; Found (ESI) 280.1624 ($C_{12}H_{23}N_3O_3Na$ requires 280.1637).

(2*R*, 4*S*, 6*S*)-1-Azido-2,4,8-tri*(tert*-butyldimethylsilyloxy)-6-methyloctanane S26

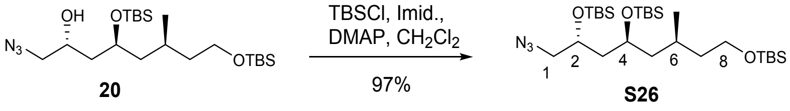

Imidazole (271 mg, 3.98 mmol), TBSCl (449 mg, 2.98 mmol) and DMAP (24 mg, 0.20 mmol) were added to a solution of azide **20** (1.03 g, 1.99 mmol) in dry CH_2Cl_2 (5 mL) at room temperature under an atmosphere of nitrogen. After 18 h the reaction was quenched with saturated aqueous NH_4Cl solution (10 mL) and the aqueous layer extracted with CH_2Cl_2 (3 × 15 mL). The combined organic layers were dried ($MgSO_4$), filtered and concentrated *in vacuo* to give a crude yellow oil. Purification by column chromatography (2% Et_2O in P.E.) gave protected alcohol **S26** as a clear liquid (1.06 g, 97%). $[\alpha]_D^{21}$ +12.0 (*c* 1.0, $CHCl_3$); υ_{max} (neat) / cm^{-1} 2955 (CH), 2929 (CH), 2887 (CH), 2857 (CH), 2101 (N=N=N), 1472, 1463, 1253; δ_H (400 MHz, $CDCl_3$) 3.90 (1H, m, 2-H), 3.83 (1H, m, 4-H), 3.62 (2H, m, 8-H_2), 3.34 (1H, dd, *J* 12.5, 4.0, 1-*H*H), 3.13 (1H, dd, *J* 12.5, 6.0, 1-H*H*), 1.68 (1H, ddd, *J* 14.0, 6.0, 4.0 , 3-*H*H), 1.64-1.49 (3H, m, 3-H*H*, 6-H, 7-*H*H), 1.45-1.26 (3H, m,5-H_2, 7-H*H*), 0.91-0.86 (30H, m, 6-CH_3, 3 × $SiC(CH_3)_3$) 0.12 (3H, s, $SiCH_3$), 0.10 (3H, s, $SiCH_3$), 0.07 (3H, s, $SiCH_3$), 0.06 (3H, s, $SiCH_3$), 0.05 (6H, s, 2 × $SiCH_3$); δ_C (100 MHz, $CDCl_3$) 70.2 (C-2), 68.6 (C-4), 61.2 (C-8), 57.8 (C-1), 46.1 (C-5), 43.3 (C-3), 40.7 (C-7), 26.2 (SiC(*C*H_3)$_3$), 26.1 (SiC(*C*H_3)$_3$), 26.0 (SiC(*C*H_3)$_3$), 19.9 (6-CH_3), 18.5 (Si*C*(CH_3)$_3$), 18.2 (Si*C*(CH_3)$_3$), 18.1 (Si*C*(CH_3)$_3$), −3.5 ($SiCH_3$), −4.2 ($SiCH_3$), −4.3 ($SiCH_3$), −5.1 ($SiCH_3$), −5.2 ($SiCH_3$); *m/z* (ESI) 560.41 $[M+H]^+$; Found (ESI) 560.4091 ($C_{27}H_{62}O_3N_3Si_3$ requires 560.4093).

(2*R*, 4*S*, 6*S*)-1-Amino-2,4,8-tri*(tert*-butyldimethylsilyloxy)-6-methyloctanane 22

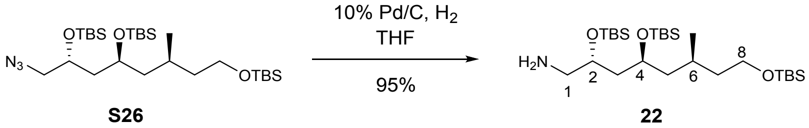

10% Pd/C (174 mg) was added to a solution of azide **S26** (1.74 g, 3.11 mmol) in THF (8 mL) and the vessel flushed with H_2 (1 atm). The reaction was stirred at room temperature for 3 h. The reaction mixture was filtered through a short pad of Celite® and then washed through with CH_2Cl_2 (100 mL). The solvent was removed *in vacuo* to give amine **22** as a yellow oil (1.66 g, 95%). $[\alpha]_D^{20}$ +15.0 (*c* 1.0, $CHCl_3$); υ_{max} (neat) / cm^{-1} 2954 (CH), 2929 (CH), 2886 (CH), 2857 (CH), 1472, 1463, 1253; δ_H (400 MHz, $CDCl_3$) 3.82 (1H, m, 4-H), 3.73 (1H, m, 2-H), 3.62 (2H, m, 8-H_2), 2.75 (1H, dd, *J* 13.0, 4.0, 1-*H*H), 2.61 (1H, dd, *J* 13.0, 5.0, 1-H*H*), 1.67-1.25 (7H, m, 3-H_2, 5-H_2, 6-H, 7-H_2), 0.89-0.87 (30H, m, 6-CH_3, 3 × $SiC(CH_3)_3$), -0.08 ($SiCH_3$), -0.07 ($SiCH_3$), -0.06 ($SiCH_3$), -0.05 ($SiCH_3$), -0.04 (2 × $SiCH_3$); δ_C (100 MHz, $CDCl_3$) 71.9 (C-4), 68.7 (C-2), 61.3 (C-8), 48.7 (C-1), 46.1 (CH_2), 43.2 (CH_2), 40.6 (CH_2), 26.5 (C-6), 26.2 (SiC(*C*H_3)$_3$), 26.1 (SiC(*C*H_3)$_3$), 26.0 (SiC(*C*H_3)$_3$), 20.0 (6-CH_3), 18.5 (Si*C*(CH_3)$_3$), 18.2 (2 × Si*C*(CH_3)$_3$), −3.5 ($SiCH_3$), −3.8 ($SiCH_3$), −4.0 ($SiCH_3$), −4.1 ($SiCH_3$), −5.1 ($SiCH_3$), −5.2 ($SiCH_3$); *m/z* (ESI) 534.42 $[M+H]^+$; Found (ESI) 534.4185 ($C_{27}H_{64}NO_3Si_3$ requires 534.4189).

Synthesis of PMB protected alcohol 23

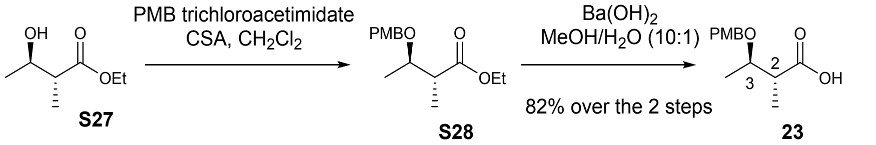

4-Methoxybenzyl trichloroacetimidate (4.4 mL, 6.8 mmol) and CSA (80 mg, 0.34mmol) were added to a solution of ester **S27** (500 mg, 3.42 mmol) in dry CH_2Cl_2 (17 mL). The solution was stirred a room temperature for 24 h. Water (20 mL) was added, the layers separated and the aqueous layer extracted with CH_2Cl_2 (3 × 20 mL). The combined organic extracts were washed with saturated $NaHCO_{3(aq)}$ (10 mL), dried ($MgSO_4$), filtered and concentrated *in vacuo*. Purification by flash column chromatography (10% EtOAc in P.E.) gave ester **S28** as a mixture with PMB_2O which was used in the next step without further purification. $Ba(OH)_2$ (2.69 g, 15.7 mmol) was added to a solution of ester **S28** (837 mg, 3.14 mmol) in a mixture of MeOH (29 mL) and H_2O (3 mL). The reaction was stirred at room temperature for 5 h. The reaction mixture was acidified with HCl (2 M) until the pH ~ 2 and then the aqueous layer was extracted with EtOAc (5 × 50 mL). The combined organic layers were dried ($MgSO_4$), filtered and concentrated *in vacuo*. Purification by column chromatography (10 to 50% EtOAc in P.E.) gave acid **23** as a yellow oil (669 mg, 82% over the 2 steps). $[\alpha]_D^{20}$ −37.0 (*c* 1.0, $CHCl_3$); υ_{max} (neat) / cm^{-1} 3000 (OH), 2976 (CH), 2936 (CH), 2837 (CH), 1704 (C=O), 1613, 1512; δ_H (400 MHz, $CDCl_3$) 7.24 (2H, d, *J* 8.5, ArH), 6.85 (2H, d, *J* 8.5, ArH), 4.54 (1H, d, *J* 12.0, ArC*H*H), 4.43 (1H, d, *J* 12.0, ArCH*H*), 3.78 (3H, s, OMe), 3.77 (1H, *overlapping* m, 3-H), 2.67 (1H, *app.* quintet, *J* 7.0, 2-H), 1.21 (3H, d, *J* 6.5, 4-H_3), 1.17 (3H, d, *J* 7.0, 2-CH_3); δ_C (100 MHz, $CDCl_3$) 180.4 (C-1), 159.3 (ArC), 130.3 (ArC), 129.5 (ArCH), 113.9 (ArCH), 76.0 (C-3), 70.9 ($ArCH_2O$), 55.4 (OMe), 45.6 (C-2), 16.6 (C-4), 12.7 (2-CH_3); *m/z* (ESI) 261.11 $[M+Na]^+$; Found (ESI) 261.1091 ($C_{13}H_{18}O_4N_a$ requires 261.1097).

(10*R*, 11*R*)-11-(*p*-Methoxybenzyloxy)-10-methyl-*N*-((3*S*, 5*S*, 7*R*)-1,5,7-tri*(tert*butyldimethylsilyloxy)-3-methyloctyl)butamide 24

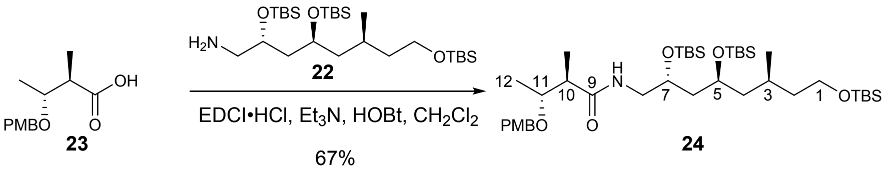

Acid **23** (85 mg, 0.32 mmol), amine **22** (188 mg, 0.35 mmol), EDCI.HCl (74 mg, 0.38 mmol) were dissolved in DMF (2 mL) and cooled to 0 °C before adding HOBt (8 mg, 0.064 mmol) followed by the dropwise addition of Et_3N (50 µL, 0.38 mmol). The reaction was allowed to warm to room temperature and stirred for 12 h. Azeotropic removal of DMF with toluene (5 × 50 mL) gave a crude oil which was taken up in EtOAc (20 mL) and citric acid (1 M, 15 mL). The aqueous layer was extracted with EtOAc (3 × 20 mL) and then the combined organic phases were washed with saturated $NaHCO_{3(aq)}$ solution (20 mL), brine (20 mL), dried ($MgSO_4$), filtered and concentrated *in vacuo* to give a crude orange oil. Purification by column chromatography (10 to 20% EtOAc in P.E.)

gave amide **24** as a clear oil (162 mg, 67%). $[\alpha]_D^{22}$ +8.0 (*c* 1.0, $CHCl_3$); υ_{max} (neat) / cm^{-1} 2954 (CH), 2928 (CH), 2884 (CH), 2856 (CH), 1656 (C=O), 1248, 1092; δ_H (400 MHz, $CDCl_3$) 7.22 (2H, d, *J* 8.5, ArH), 6.85 (2H, d, *J* 8.5, ArH), 6.27 (1H, t, *J* 5.5, NH), 4.53 (1H, d, *J* 11.0, ArC*H*H), 4.37 (1H, d, *J* 11.0, ArCH*H*), 3.88 (1H, m, 7-H), 3.83 (1H, m, 5-H), 3.78 (3H, s, OMe), 3.68-3.56 (3H, m, 1-H_2, 11-H), 3.42 (1H, dt, *J* 13.5, 4.5, 8-*H*H), 3.21 (1H, dt, *J* 13.5, 5.0, 8-H*H*), 2.32 (1H, quintet, *J* 7.0, 10-H), 1.64-1.52 (3H, *overlapping* m, 2-*H*H, 3-H, 6-*H*H), 1.45 (1H, m, 6-H*H*), 1.37-1.25 (3H, m, 2-H*H*, 4-H_2), 1.19 (3H, d, *J* 6.0, 12-H_3), 1.14 (3H, d, *J* 7.0, 10-CH_3), 0.89-0.86 (30H, *overlapping* m, 3-CH_3, 3 × $SiC(CH_3)_3$), 0.08 (6H, s, 2 × $SiCH_3$), 0.07 (6H, s, 2 × $SiCH_3$), 0.03 (6H, s, 2 × $SiCH_3$); δ_C (100 MHz, $CDCl_3$) 174.9 (C-9), 159.3 (ArC), 130.6 (ArC), 129.5 (ArCH), 113.9 (ArCH), 76.5 (C-11), 71.2 ($ArCH_2$), 69.1 (C-7), 68.4 (C-5), 61.4 (C-1), 55.4 (OMe), 48.2 (C-10), 45.5 (C-8), 46.1 (C-4), 43.6 (C-6), 40.5 (C-2), 26.6 (C-3), 26.2 ($SiC(\mathit{C}H_3)_3$), 26.1 ($SiC(\mathit{C}H_3)_3$), 26.0 ($SiC(\mathit{C}H_3)_3$), 19.9 (3-CH_3), 18.5 ($Si\mathit{C}(CH_3)_3$), 18.2 ($Si\mathit{C}(CH_3)_3$), 18.1 ($Si\mathit{C}(CH_3)_3$), 17.3 (C-12), 14.8 (10-CH_3), −3.4 ($SiCH_3$), −3.7 ($SiCH_3$), −4.0 ($SiCH_3$), −4.2 ($SiCH_3$), −5.1 ($SiCH_3$), −5.2 ($SiCH_3$); *m/z* (ESI) 754.53 $[M+H]^+$; Found (ESI) 754.5283 ($C_{40}H_{80}NO_6Si_3$ requires 754.5288).

(10*R*, 11*R*)-11-(*p*-Methoxybenzyl)oxy)-10-methyl-*N*-((3*S*, 5*S*, 7*R*)-5,7-di*(tert*-butyldimethylsilyloxy)-1-hydroxy-3-methyl-octyl)butamide S29

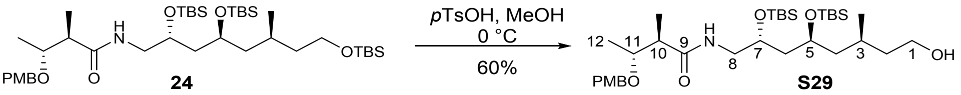

*p*TsOH (14 mg, 0.15 mmol) was added to a solution of amide **24** (1.12 g, 1.48 mmol) in MeOH (15 mL) at 0 °C. The solution was stirred for 30 mins, quenched with solid $NaHCO_3$, filtered and concentrated *in vacuo* to give a crude yellow oil. Further purification by column chromatography (10 to 100% EtOAc in P.E.) gave alcohol **S29** as a colourless oil (568 mg, 60%). $[\alpha]_D^{23}$ +1.0 (*c* 1.0, $CHCl_3$); υ_{max} (neat) / cm^{-1} 3362 (OH), 2954 (CH), 2929 (CH), 2856 (CH), 1652 (C=O), 1614, 1514, 1249, 1067; δ_H (400 MHz, $CDCl_3$) 7.22 (2H, d, *J* 8.5, ArH), 6.85 (2H, d, *J* 8.5, ArH), 6.30 (1H, t, *J* 5.0, NH), 4.53 (1H, d, *J* 11.0, ArC*H*H), 4.37 (1H, d, *J* 11.0, ArCH*H*), 3.89 (1H, m, 7-H), 3.83 (1H, m, 5-H), 3.79 (3H, s, OMe), 3.65 (1H, m, 11-H), 3.57 (2H, t, *J* 6.5, 1-H_2), 3.42 (1H, dt, *J* 13.5, 4.5, 8-*H*H), 3.21 (1H, dt, *J* 13.5, 5.5, 8-H*H*), 2.29 (1H, quintet, *J* 7.0, 10-H), 1.69-1.39 (6H, m, 3-H, 2-H_2, 4-*H*H, 6-H_2), 1.25 (1H, m, 4-H*H*), 1.19 (3H, d, *J* 6.0, 12-H_3), 1.13 (3H, d, *J* 7.0, 10-CH_3), 0.89 (3H, d, *J* 6.5, 3-CH_3), 0.88 (9H, s, $SiC(CH_3)_3$), 0.87 (9H, s, $SiC(CH_3)_3$), 0.07 (3H, s, $SiCH_3$), 0.06 (9H, s, 3 × $SiCH_3$); δ_C (100 MHz, $CDCl_3$) 175.2 (C-9), 159.3 (ArC), 130.6 (ArC), 129.5 (ArCH), 113.9 (ArCH), 76.4 (C-11), 71.3 ($ArCH_2$), 68.8 (C-7), 68.4 (C-5), 60.2 (C-1), 55.4 (OMe), 48.2 (C-10), 45.5 (C-8), 44.9 (C-4), 43.2 (C-6), 39.9 (C-2), 26.3 (C-3), 26.1 ($SiC(\mathit{C}H_3)_3$), 25.9 ($SiC(\mathit{C}H_3)_3$), 20.3 (3-CH_3), 18.2 ($Si\mathit{C}(CH_3)_3$), 18.1 ($Si\mathit{C}(CH_3)_3$), 17.3 (C-12), 14.8 (10-CH_3), −3.9 ($Si\mathit{C}H_3$), −4.0 ($Si\mathit{C}H_3$), −4.3 ($Si\mathit{C}H_3$), −4.4 ($Si\mathit{C}H_3$); *m/z* (ESI) 640.44 $[M+H]^+$; Found (ESI) 640.4402 ($C_{34}H_{66}NO_6Si_2$ requires 640.4423).

(3*S*, 5*S*, 7*R*)-5,7-Di*(tert*-butyldimethylsilyloxy)-8-(10*R*, 11*R*)-11-(*p*-methoxybenzyloxy)-10-methylbutamido-3-methyloctanoic acid 25

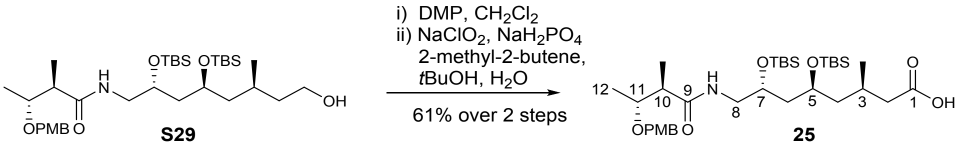

Dess-Martin periodinane (3.10 mL, 1.07 mmol) was added to a solution of alcohol **S29** (274 mg, 0.429 mmol) in CH_2Cl_2 (14 mL) at room temperature under an atmosphere of nitrogen. After 1 h, saturated $NaHCO_{3(aq)}$ solution (15 mL) was added and the product extracted in CH_2Cl_2 (3 × 20 mL). The combined organic layers were dried ($MgSO_4$), filtered and concentrated *in vacuo*. Further purification by column chromatography (20% EtOAc in P.E.) gave the corresponding aldehyde as a clear oil. A solution of $NaClO_2$ (261 mg, 2.89 mmol) and NaH_2PO_4 (347 mg, 2.89 mmol) in H_2O (5 mL) was added to a solution of the aldehyde in ^{t}BuOH (18 mL) and 2-methyl-2-butene (2 mL) at 0 °C. After 1 h, the reaction was quenched with pH 7 phosphate buffer (20 mL) and extracted with CH_2Cl_2 (3 × 20 mL). Further purification by column chromatography (20 to 50% EtOAc in P.E. with 0.5% CH_3COOH) gave acid **25** as a clear oil (172 mg, 61%). $[\alpha]_D^{23}$ −27.0 (*c* 0.67, $CHCl_3$); υ_{max} (neat) / cm^{-1} 3354 (OH), 2954 (CH), 2929 (CH), 2856 (CH), 1712 (C=O), 1642 (C=O), 1514, 1379, 1249; δ_H (400 MHz, $CDCl_3$) 7.22 (2H, d, *J* 8.5, ArH), 6.85 (2H, d, *J* 8.5, ArH), 6.39 (1H, t, *J* 6.0, NH), 4.54 (1H, d, *J* 11.0, ArC*H*H), 4.37 (1H, d, *J* 11.0, ArCH*H*), 3.88-3.71 (2H, m, 5-H, 7-H), 3.79 (3H, s, OMe), 3.65 (1H, quintet, *J* 6.5, 11-H), 3.51 (1H, ddd, *J* 13.5, 6.0, 4.0, 8-*H*H), 3.08 (1H, dt, *J* 13.5, 5.5, 8-H*H*), 2.34- 2.18 (3H, m, 2-H_2, 10-H), 2.03 (1H, sextet, *J* 6.5, 3-H), 1.66-1.44 (3H, m, 4-H_2, 6-*H*H), 1.33 (1H, m, 6-H*H*), 1.19 (3H, d, *J* 6.0, 12-H_3), 1.14 (3H, d, *J* 7.0, 10-CH_3), 0.99 (3H, d, *J* 6.5, 3-CH_3), 0.88 (9H, s, $SiC(CH_3)_3$), 0.87 (9H, s, $SiC(CH_3)_3$), 0.08 (3H, s, $SiCH_3$), 0.07 (3H, s, $SiCH_3$), 0.06 (3H, s, $SiCH_3$), 0.05 (3H, s, $SiCH_3$); δ_C (100 MHz, $CDCl_3$) 175.9 (C-9), 175.5 (C-1), 159.3 (ArC), 130.5 (ArC), 129.6 (ArCH), 114.0 (ArCH), 76.7 (C-11), 71.3 ($ArCH_2$), 69.0 (C-7), 68.5 (C-5), 55.4 (OMe), 48.2 (C-10), 45.4 (C-8), 44.3 (C-4), 43.8 (C-6), 41.9 (C-2), 27.5 (C-3), 26.0 ($SiC(CH_3)_3$), 25.9 ($SiC(CH_3)_3$), 21.2 (3-CH_3), 18.2 ($SiC(CH_3)_3$), 18.1 ($SiC(CH_3)_3$), 17.4 (C-12), 14.7 (10-CH_3), −3.8 ($SiCH_3$), −4.0 ($SiCH_3$), −4.2 ($SiCH_3$), −4.2 ($SiCH_3$); *m/z* (ESI) 654.42 $[M+H]^+$; Found (ESI) 654.4211 ($C_{34}H_{64}NO_7Si_2$ requires 654.4216).

PMB protected lactone 26

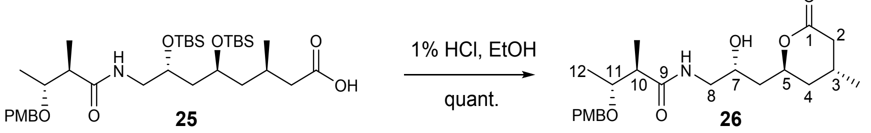

Concentrated HCl (12 M, 150 µL) was added to a solution of acid **25** (439 mg, 0.671 mmol) in ethanol (15 mL) and stirred at room temperature for 3 h. The reaction mixture was concentrated *in vacuo* to give a crude pink oil which was purified by column chromatography (50 to 100% EtOAc in P.E.) to give lactone **26** as a colourless oil (273 mg, quant.). $[\alpha]_D^{23}$ +14.0 (*c* 1.0, $CHCl_3$); υ_{max} (neat) / cm^{-1} 3348 (OH), 2959 (CH), 2931 (CH), 2883 (CH), 2840 (CH), 1722 (C=O), 1645 (C=O), 1513, 1245, 1070; δ_H (400 MHz, $CDCl_3$) 7.23 (2H, d, *J* 8.5, ArH), 6.87 (2H, d, *J* 8.5, ArH), 6.65 (1H, br m, NH), 4.62 (1H, m, 5-H), 4.53 (1H, d, *J* 11.0, ArC*H*H), 4.36 (1H, d, *J* 11.0, ArCH*H*), 3.98 (1H, m, 7-H), 3.79 (3H, s, OMe), 3.63 (1H,

quintet, *J* 6.5, 11-H), 3.37 (1H, ddd, *J* 14.0, 6.0, 3.0, 8-*H*H), 3.23 (1H, dt, *J* 14.0, 6.5, 8-H*H*), 2.54 (1H, dd, *J* 16.0, 9.5, 2-*H*H), 2.34 (1H, quintet, *J* 7.0, 10-H), 2.19-2.09 (2H, m, 2-H*H*, 3-H), 1.78-1.50 (4H, m, 4-H_2, 6-H_2), 1.21 (3H, d, *J* 6.0, 12-CH_3), 1.14 (3H, d, *J* 7.0, 10-CH_3), 1.07 (3H, d, *J* 6.0, 3-CH_3); δ_C (100 MHz, $CDCl_3$) 177.2 (C-9), 172.7 (C-1), 159.4 (ArC), 130.3 (ArC), 129.6 (ArCH), 114.0 (ArCH), 76.8 (C-11), 74.2 (C-5), 71.3 (ArCH_2O), 68.1 (C-7), 55.4 (OMe), 48.0 (C-10), 46.5 (C-8), 40.7 (C-6), 37.5 (C-2), 35.7 (C-4), 24.1 (C-3), 21.6 (3-CH_3), 17.5 (C-12), 15.0 (10-CH_3); *m/z* (ESI) 408.24 $[M+H]^+$; Found (ESI) 408.2365 ($C_{22}H_{34}NO_6$ requires 408.2381).

Protected lactone S30

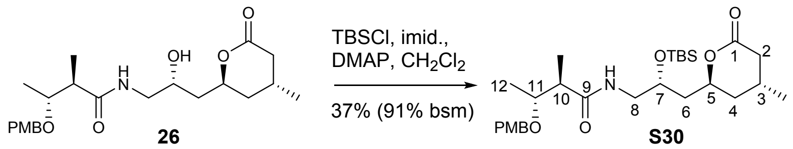

Imidazole (69 mg, 1.0 mmol), TBSCl (91 mg, 0.61 mmol) and DMAP (7 mg, 0.06 mmol) were added to a solution of lactone **26** (206 mg, 0.506 mmol) in dry CH_2Cl_2 (2 mL) at room temperature under an atmosphere of nitrogen. After 24 h the reaction was quenched with saturated $NH_4Cl_{(aq)}$ solution (10 mL) and the aqueous layer extracted with CH_2Cl_2 (3 × 15 mL). The combined organic layers were dried ($MgSO_4$), filtered and concentrated *in vacuo* to give a crude clear oil. Purification by column chromatography (80% EtOAc in P.E.) gave protected alcohol **S30** as a clear oil (97 mg, 37%) followed by starting lactone **26** as a colourless oil (112 mg, 54%) which was recycled in the same reaction. $[\alpha]_D^{23}$ +43.0 (*c* 1.0, $CHCl_3$); υ_{max} (neat) / cm^{-1} 2955 (CH), 2929 (CH), 2856 (CH), 1733 (C=O), 1652 (C=O), 1513, 1247, 1108; δ_H (400 MHz, $CDCl_3$) 7.22 (2H, d, *J* 8.5, ArH), 6.84 (2H, d, *J* 8.5, ArH), 6.23 (1H, br m, NH), 4.53 (1H, d, *J* 11.0, ArC*H*H), 4.42 (1H, m, 5-H), 4.36 (1H, d, *J* 11.0, ArCH*H*), 4.09 (1H, m, 7-H), 3.78 (3H, s, OMe), 3.65 (1H, quintet, *J* 6.5, 11-H), 3.42 (1H, ddd, *J* 14.0, 7.0, 3.0, 8-H*H*), 3.22 (1H, dt, *J* 14.0, 5.0, 8-H*H*), 2.53 (1H, m, 2-*H*H), 2.28 (1H, quintet, *J* 7.0, 10-H), 2.17-2.03 (2H, m, 2-H*H*, 3-H), 1.66-1.50 (3H, m, 4-*H*H, 6-H_2), 1.35 (1H, ddd, *J* 14.0, 6.0, 4.0, 4-H*H*), 1.19 (3H, d, *J* 6.0, 12-CH_3), 1.14 (3H, d, *J* 7.0, 10-CH_3), 1.02 (3H, d, *J* 6.5, 3-CH_3), 0.87 (9H, s, SiC(*C*H_3)$_3$), 0.08 (3H, s, SiCH_3), 0.06 (3H, s, SiCH_3); δ_C (100 MHz, $CDCl_3$) 175.3 (C-9), 172.3 (C-1), 159.3 (ArC), 130.7 (ArC), 129.3 (ArCH), 113.9 (ArCH), 76.8 (C-11), 73.4 (C-5), 71.1 (ArCH_2O), 67.0 (C-7), 55.4 (OMe), 48.5 (C-10), 45.0 (C-8), 40.8 (C-6), 37.5 (C-2), 35.5 (C-4), 25.9 (SiC(*C*H_3)$_3$), 24.1 (C-3), 21.5 (3-CH_3), 18.1 (Si*C*(CH_3)$_3$), 17.4 (C-12), 15.2 (10-CH_3), −4.4 (SiCH_3), −4.8 (SiCH_3); *m/z* (ESI) 522.32 $[M+H]^+$; Found (ESI) 522.3243 ($C_{28}H_{48}NO_6Si$ requires 522.3245).

TBS protected lactone 27

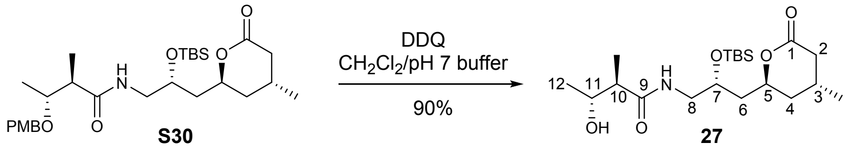

DDQ (35 mg, 0.15 mmol) was added to a solution of amide **S30** (53 mg, 0.10 mmol) in CH_2Cl_2 (1.6 mL) and pH 7 phosphate buffer (0.1 mL) cooled to 0 °C. The reaction mixture was allowed to warm to room temperature and stirred for 1 h before quenching with pH 7 phosphate buffer (10 mL) and

extracting in CH_2Cl_2 (3 × 10 mL). The combined organic extracts were washed with brine (15 mL), dried ($MgSO_4$), filtered and concentrated *in vacuo* to give a crude pink oil. Further purification by flash column chromatography (80% EtOAc in P.E.) gave amide **27** as a clear oil (37 mg, 90%). $[\alpha]_D^{22}$ +50.0 (*c* 1.0, $CHCl_3$); υ_{max} (neat) / cm^{-1} 3344 (OH), 2962 (CH), 2935 (CH), 2858 (CH), 1730 (C=O), 1645 (C=O), 1464, 1256, 1114, 918; δ_H (400 MHz, $CDCl_3$) 6.10 (1H, br t, *J* 5.0, NH), 4.49 (1H, m, 5-H), 4.11 (1H, m, 7-H), 3.83 (1H, *app.* quintet, *J* 6.5, 11-H), 3.44 (1H, ddd, *J* 14.0, 7.0, 3.0, 8-*H*H), 3.29 (1H, br s, OH), 3.21 (1H, ddd, *J* 14.0, 5.5, 4.5, 8-H*H*), 2.55 (1H, dd, *J* 16.0, 4.5, 2-*H*H), 2.21-2.08 (3H, m, 2-H*H*, 3-H, 10-H), 1.80-1.68 (2H, m, 4-*H*H, 6-*H*H), 1.64-1.51 (2H, m, 4-H*H*, 6-H*H*), 1.21 (3H, d, *J* 6.5, 12-H_3), 1.18 (3H, d, *J* 7.0, 10-CH_3), 1.07 (3H, d, *J* 6.5, 3-CH_3), 0.88 (9H, s, 3-CH_3), $SiC(CH_3)_3$), 0.10 (3H, s, $SiCH_3$), 0.07 (3H, s, $SiCH_3$); δ_C (100 MHz, $CDCl_3$) 176.1 (C-9), 172.3 (C-1), 73.4 (C-5), 69.8 (C-11), 67.0 (C-7), 48.2 (C-10), 44.9 (C-8), 41.0 (C-6), 37.5 (C-2), 35.6 (C-4), 25.9 ($SiC(CH_3)_3$), 24.1 (C-3), 21.6 (C-11), 21.5 (3-CH_3), 18.1 ($SiC(CH_3)_3$), 15.5 (10-CH_3), −4.4 ($SiCH_3$), −4.7 ($SiCH_3$); *m/z* (ESI) 402.27 $[M+H]^+$; Found (ESI) 402.2656 ($C_{20}H_{40}NO_5Si$ requires 402.2670).

Carbamate lactone 28

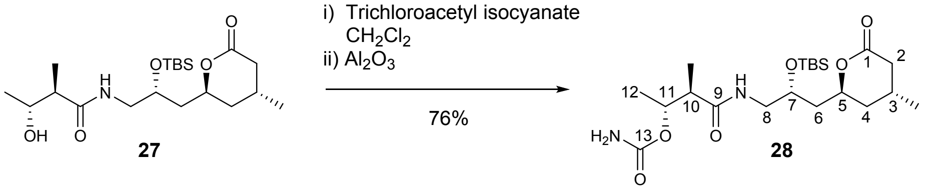

Trichloroacetyl isocyanate (47 µL, 0.39 mmol) was added to a solution of lactone **27** (105 mg, 0.261 mmol) in CH_2Cl_2 (5 mL) at room temperature under an atmosphere of nitrogen. After 2 h, Al_2O_3 was added and stirring was continued for another 3 h before dry loading the product on alumina onto a column of silica. Flash chromatography (80% EtOAc in P.E.) gave lactone **28** as a colourless oil (88 mg, 76%). $[\alpha]_D^{23}$ +58.0 (*c* 1.0, $CHCl_3$); υ_{max} (neat) / cm^{-1} 3338 (NH), 2959 (CH), 2928 (CH), 2860 (CH), 1719(C=O), 1654 (C=O), 1527, 1467, 1380, 1324, 1065; δ_H (400 MHz, $CDCl_3$) 5.96 (1H, br t, *J* 6.0, NH), 4.90 (2H, br s, NH_2), 4.87 (1H, sextet, *J* 6.5, 11-H), 4.50 (1H, tdd, *J* 9.5, 4.0, 2.5, 5-H), 4.03 (1H, ddt, *J* 9.5, 6.5, 3.0, 7-H), 3.49 (1H, ddd, *J* 13.5, 7.0, 3.0, 8-*H*H), 3.13 (1H, ddd, *J* 13.5, 6.5, 5.0, 8-H*H*), 2.55 (1H, dd, *J* 16.0, 7.0, 2-*H*H), 2.45 (1H, *app.* quintet, *J* 7.0, 10-H), 2.16 (1H, m, 3-H), 2.12 (1H, dd, *J* 16.0, 9.0, 2-H*H*), 1.81-1.73 (2H, m, 4-*H*H, 6-*H*H), 1.60-1.52 (2H, m, 4-H*H*, 6-H*H*), 1.25 (3H, d, *J* 6.5, 12-H_3), 1.13 (3H, d, *J* 7.0, 10-CH_3), 1.07 (3H, d, *J* 6.5, 3-CH_3), 0.88 (9H, s, $SiC(CH_3)_3$), 0.12 (3H, s, $SiCH_3$), 0.07 (3H, s, $SiCH_3$); δ_C (100 MHz, $CDCl_3$) 173.7 (C-9), 172.4 (C-1), 156.4 (C-13), 73.6 (C-5), 73.0 (C-11), 67.4 (C-7), 46.7 (C-10), 45.0 (C-8), 41.1 (C-6), 37.5 (C-2), 35.6 (C-4), 25.9 ($SiC(CH_3)_3$), 24.1 (C-3), 21.5 (3-CH_3), 18.1 ($SiC(CH_3)_3$), 17.7 (C-12), 13.6 (10-CH_3), −4.4 ($SiCH_3$), −4.8 ($SiCH_3$); *m/z* (ESI) 445.27 $[M+H]^+$; Found (ESI) 445.2718 ($C_{21}H_{41}N_2O_6Si$ requires 445.2728).

Synthetic lactone 10

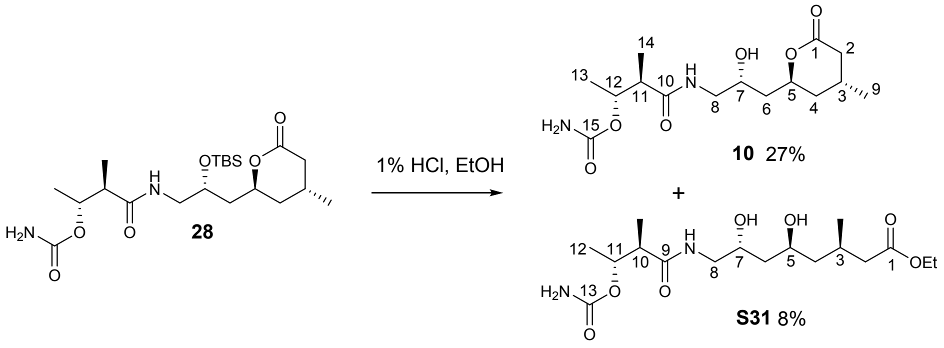

Concentrated HCl (12 M, 30 µL) was added to a solution of lactone **28** (78 mg, 0.19 mmol) in ethanol (3 mL) and stirred at room temperature for 3 h. The reaction mixture was concentrated *in vacuo* to give a clear oil which was purified by column chromatography (5 to 10% MeOH in CH_2Cl_2) to give a mixture of lactone **10** and ethyl ester **S31**. Further purification by HPLC [15 to 60 MeCN:H_2O] gave lactone **10** as a colourless oil (17 mg, 27%) and ester **S31** as a clear oil (6 mg, 8%). Lactone **10** ; $[\alpha]_D^{22}$ +25.0 (*c* 0.16, MeOH); υ_{max} (neat) / cm^{-1} 3340 (OH), 3196 (NH), 2965 (CH), 2932 (CH), 2880 (CH), 1705 (C=O), 1647 (C=O), 1548 (C=O), 1380, 1323, 1238, 1064; δ_H (500 MHz, methanol-d_4) 4.81 (1H, dq, *J* 8.5, 6.5, 12-H), 4.72 (1H, dddd, *J* 10.0, 9.0, 4.5, 2.5, 5-H), 3.91 (1H, ddt, *J* 10.5, 5.5, 2.5, 7-H), 3.27 (1H, dd, *J* 14.0, 6.0, 8-*H*H), 3.20 (1H, dd, *J* 14.0, 5.5, 8-H*H*), 2.55 (1H, dd, *J* 16.0, 5.5, 2-*H*H), 2.51 (1H, dq, *J* 8.5, 7.0, 11-H), 2.23 (1H, dd, *J* 16.0, 9.0, 2-H*H*), 2.18 (1H, m, 3-H), 1.85-1.78 (2H, m, 4-*H*H, 6-*H*H), 1.66 (1H, ddd, *J* 14.0, 6.0, 4.5, 4-H*H*), 1.54 (1H, ddd, *J* 14.5, 10.5, 2.5, 6-H*H*), 1.23 (3H, d, *J* 6.5, 13-H_3), 1.11 (3H, d, *J* 7.0, 14-H_3), 1.10 (3H, d, *J* 6.5, 9-H_3); δ_C (125 MHz, methanol-d_4) 177.0 (C-10), 175.3 (C-1), 159.1 (C-15), 75.8 (C-5), 73.6 (C-12), 67.6 (C-7), 47.5 (C-11), 46.6 (C-8), 41.7 (C-6), 38.1 (C-2), 36.4 (C-4), 25.1 (C-3), 21.5 (C-9), 18.0 (C-13), 14.1 (C-14); *m/z* (ESI) 331.19 $[M+H]^+$; Found (ESI) 331.1852 ($C_{15}H_{27}N_2O_6$ requires 331.1864). Ester **S31**; $[\alpha]_D^{22}$ +12.0 (*c* 0.16, $CHCl_3$); υ_{max} (neat) / cm^{-1} 3340 (NH/OH), 2926 (CH), 2856 (CH), 1708 (C=O), 1645 (C=O) ,1551 (C=O), 1378, 1322, 1095; δ_H (500 MHz, $CDCl_3$) 6.42 (1H, t, *J* 6.0, NH), 5.09 (2H, br s, NH_2), 4.88 (1H, dq, *J* 8.0, 6.5, 11-H), 4.14 (2H, q, *J* 7.0, OCH_2CH_3), 3.98 (1H, tt, *J* 7.5, 3.5, 7-H), 3.90 (1H, m, 5-H), 3.43 (1H, ddd, *J* 14.0, 6.5, 3.5, 8-*H*H), 3.27 (1H, ddd, *J* 14.0, 7.0, 5.5, 8H*H*), 2.50 (1H, sextet, *J* 7.0, 10-H), 2.34 (1H, dd, *J* 15.5, 7.0, 2-*H*H), 2.22 (1H, dd, *J* 15.5, 6.0, 2-H*H*), 2.17 (1H, m, 3-H), 1.62 (1H, ddd, *J* 14.5, 8.0, 3.0, 6-*H*H), 1.59-1.51 (2H, m, 4-*H*H, 6-H*H*), 1.36 (1H, ddd, *J* 14.0, 8.0, 4.5, 4-H*H*), 3.54 (3H, d, *J* 6.5, 12-H_3), 1.26 (3H, t, *J* 7.0, OCH_2CH_3), 1.16 (3H, d, *J* 7.0, 10-CH_3), 0.99 (3H, d, *J* 6.5, 3-CH_3); δ_C (125 MHz, $CDCl_3$) 174.9, 174.3, 156.7 (C-13), 73.2 (C-11), 68.9 (C-7), 66.7 (C-5), 60.8 (OCH_2CH_3), 47.2 (C-10), 45.8 (C-8), 45.1 (C-4), 41.3 (C-2), 40.5 (C-6), 26.7 (C-3), 21.3 (3-CH_3), 18.1 (C-12), 14.4 (OCH_2CH_3), 14.1 (10-CH_3); *m/z* (ESI) 377.23 $[M+H]^+$; Found (ESI) 377.2296 ($C_{17}H_{33}N_2O_6$ requires 377.2282).

5g) Spectra

Kalimantacin A 1 ^{1}H NMR (500 MHz, $CDCl_3$)

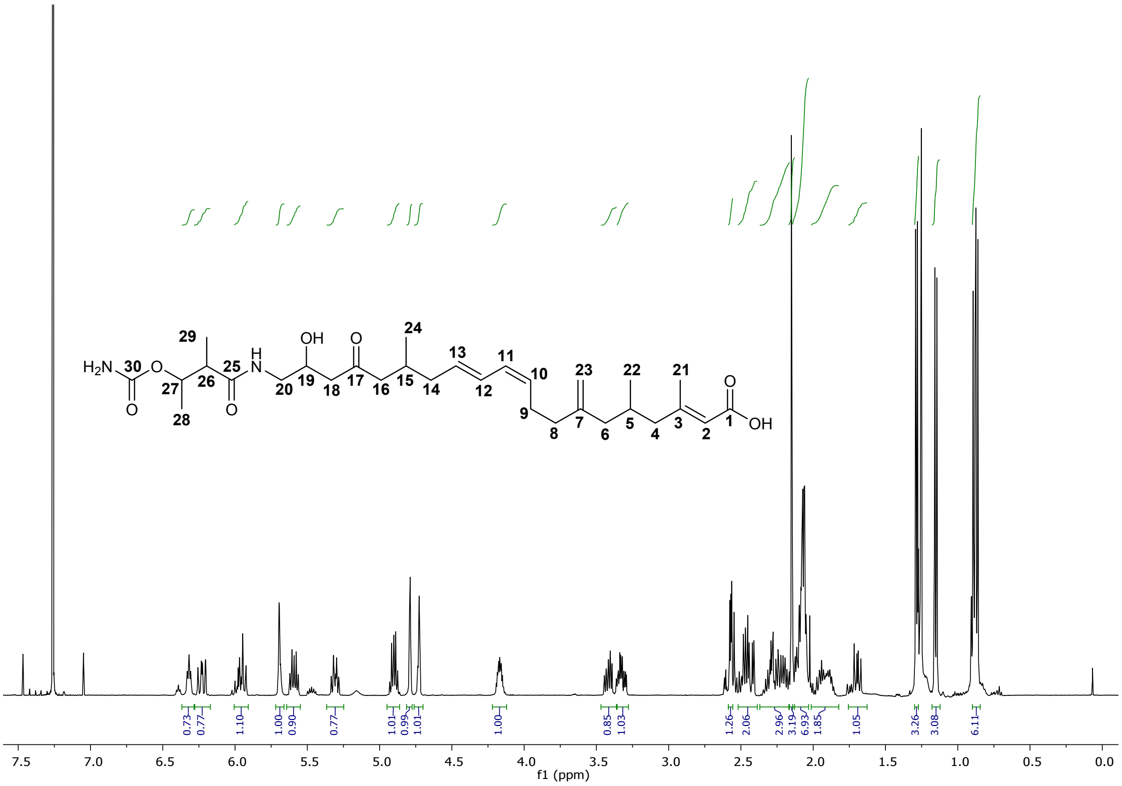

^{13}C NMR (125 MHz, $CDCl_3$)

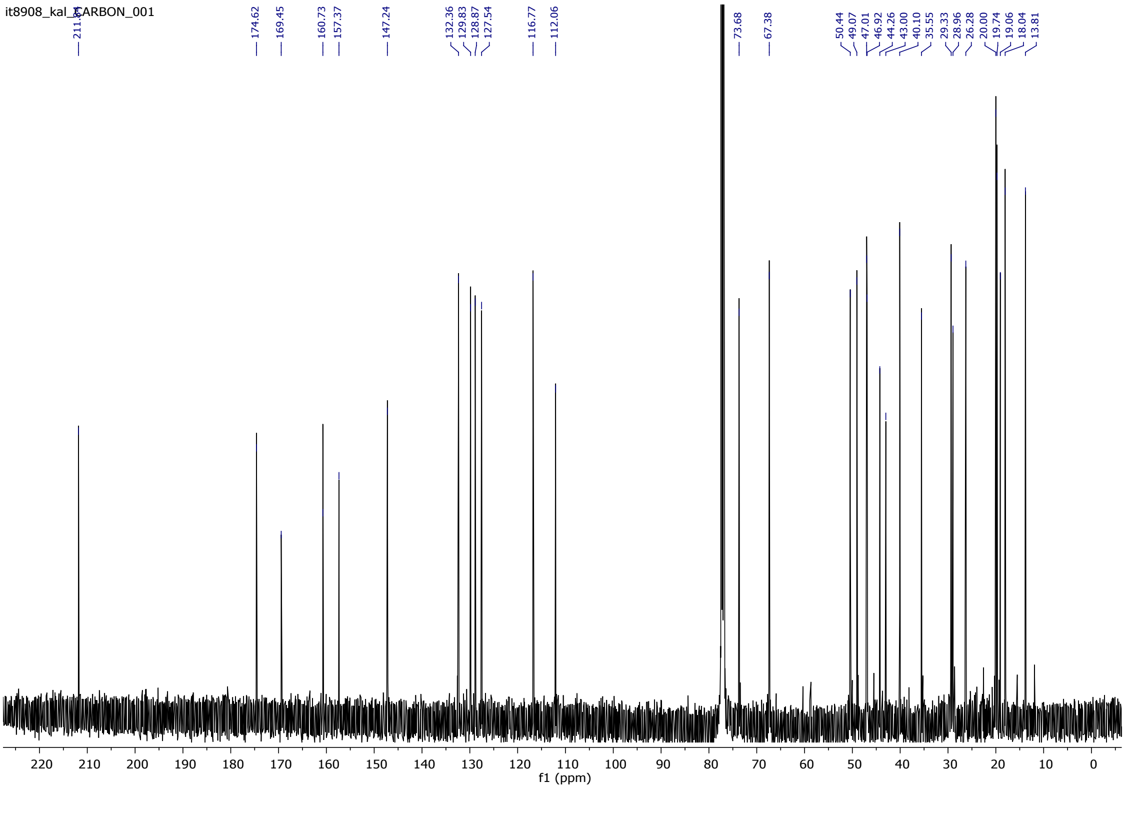

Diol 2

^{1}H NMR (500 MHz, $CDCl_3$)

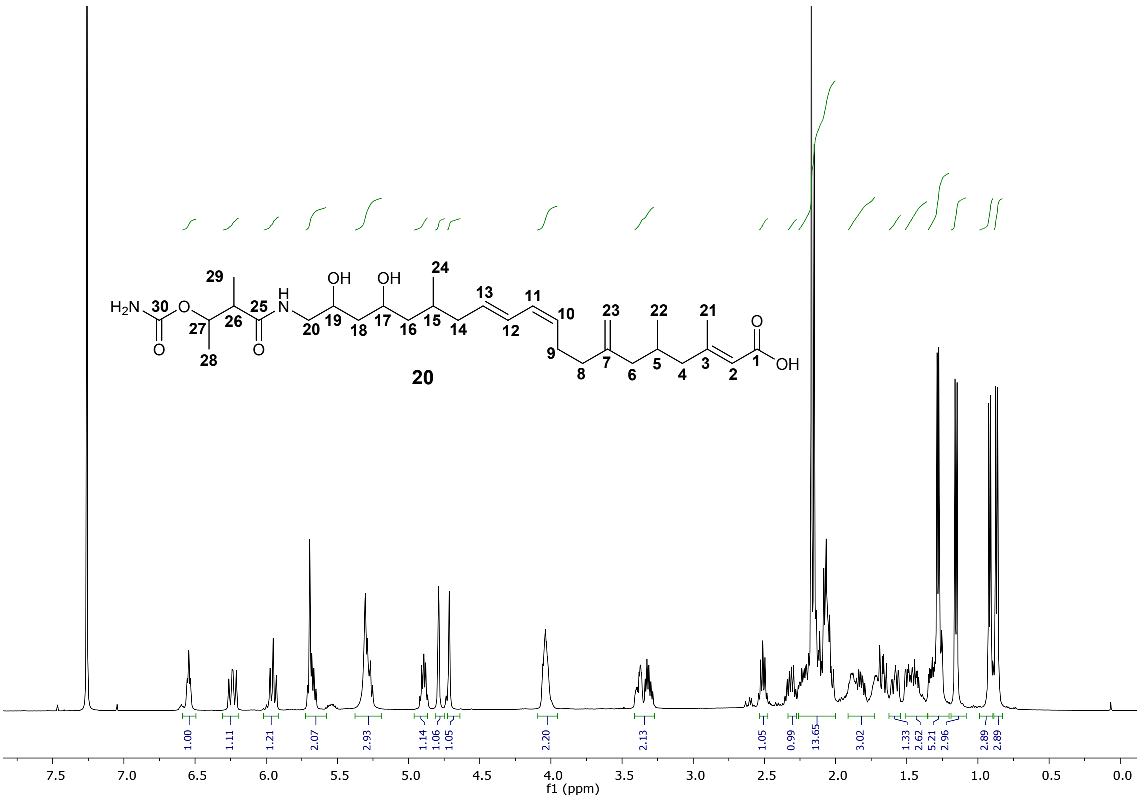

^{13}C NMR (125 MHz, $CDCl_3$)

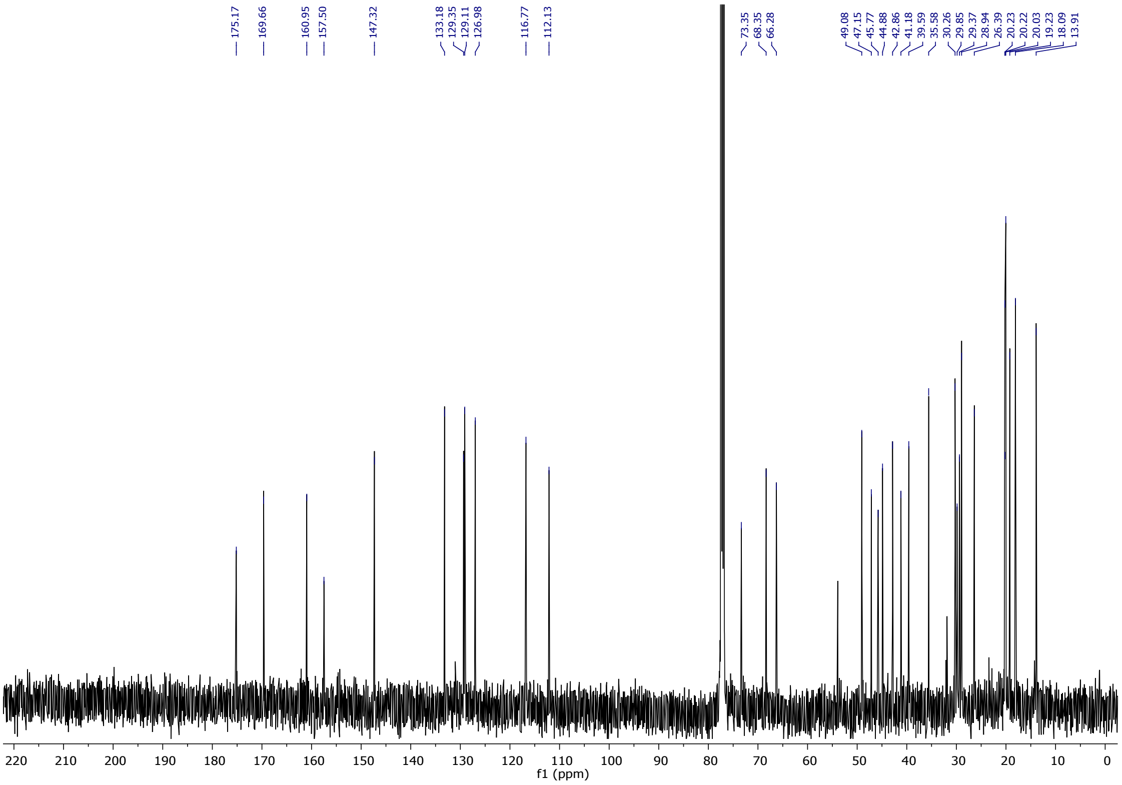

27-Descarbamoyl diol 3 ^{1}H NMR (500 MHz, $CDCl_3$)

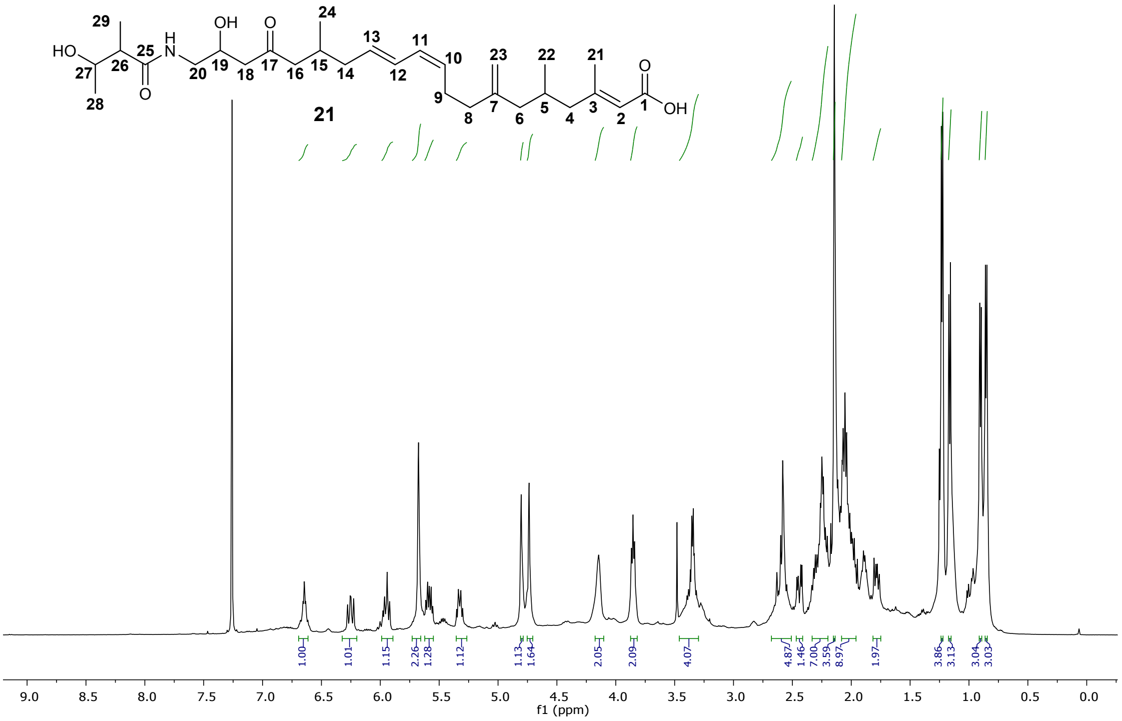

^{13}C NMR (125 MHz, $CDCl_3$)

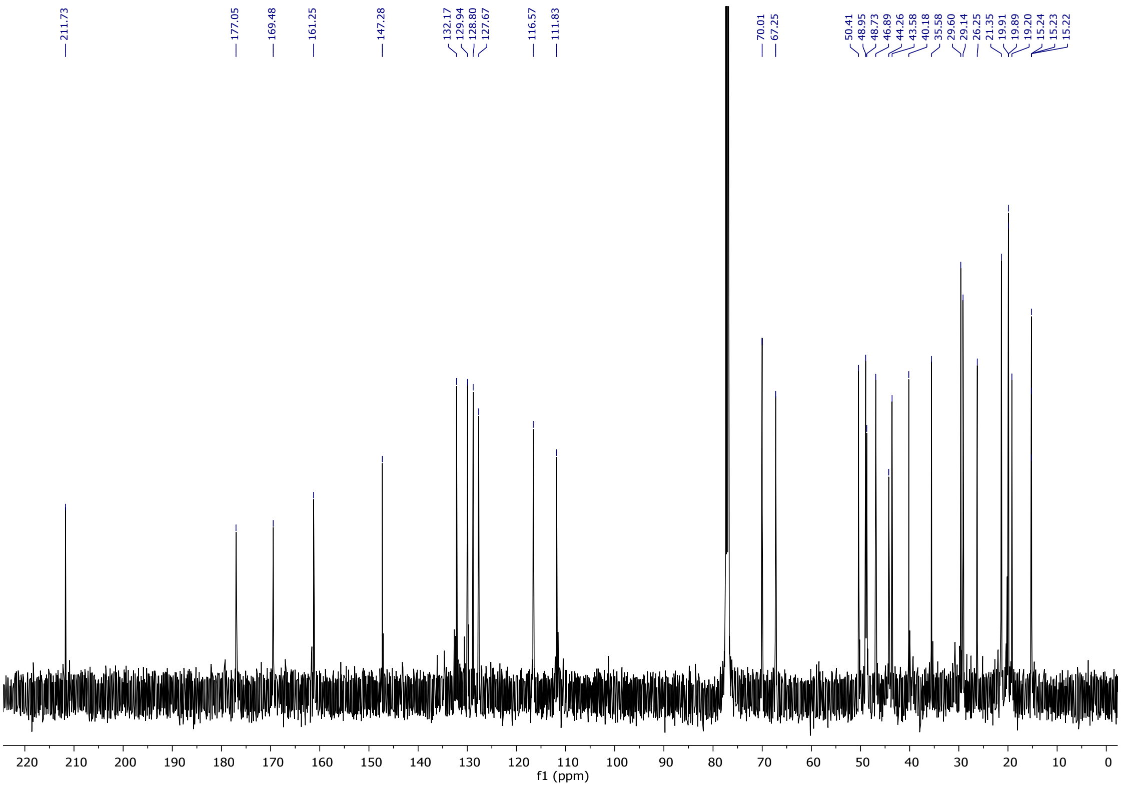

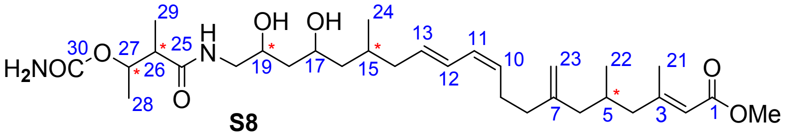

S8

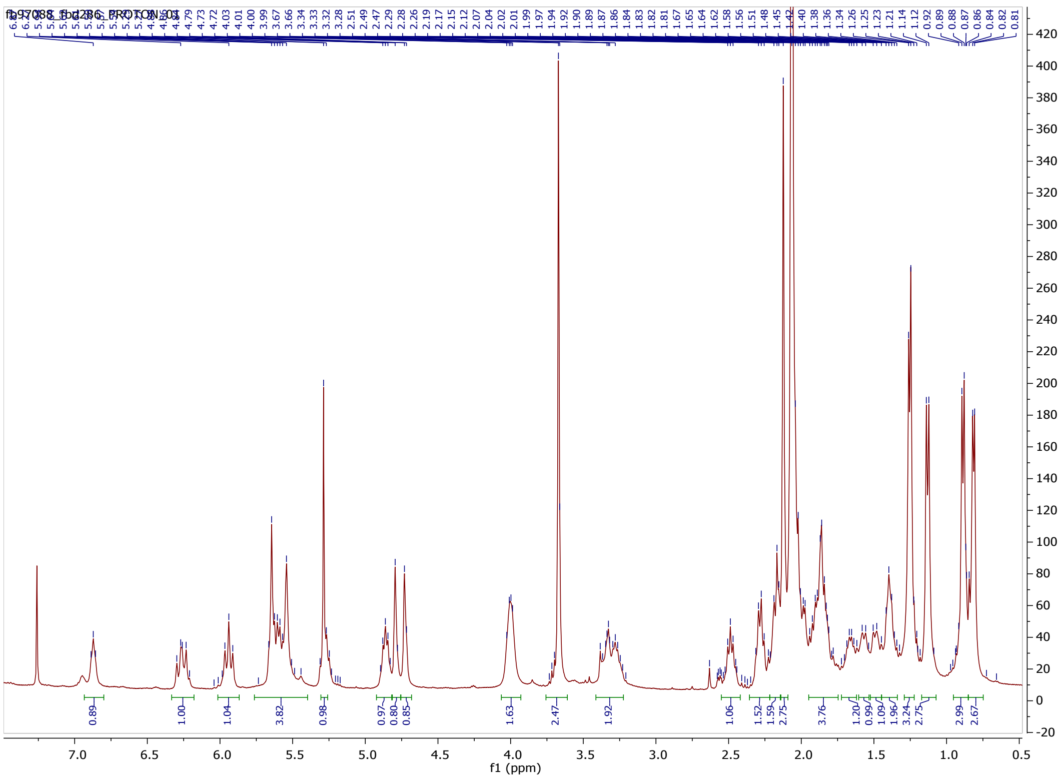
fb97088_fbd286_PROTON_01
f1 (ppm)

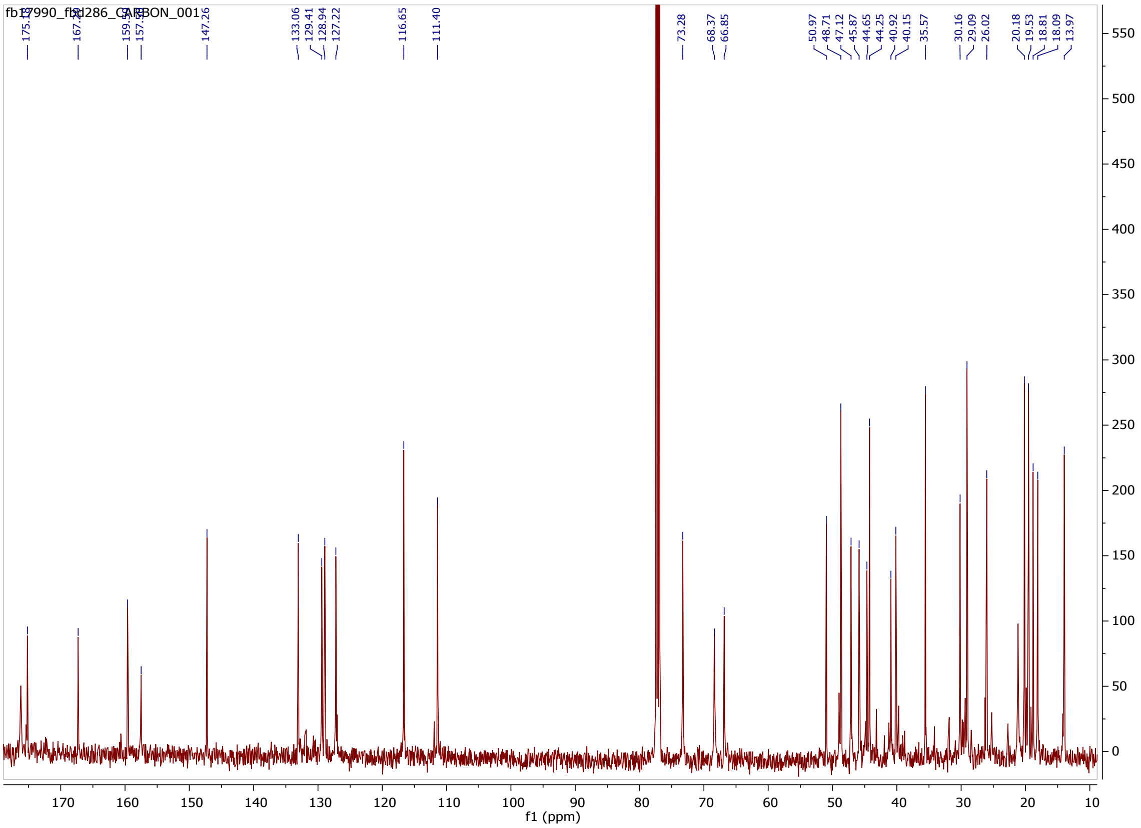
fb17990_fbd286_CARBON_001
f1 (ppm)

Degradation Product (-)-29

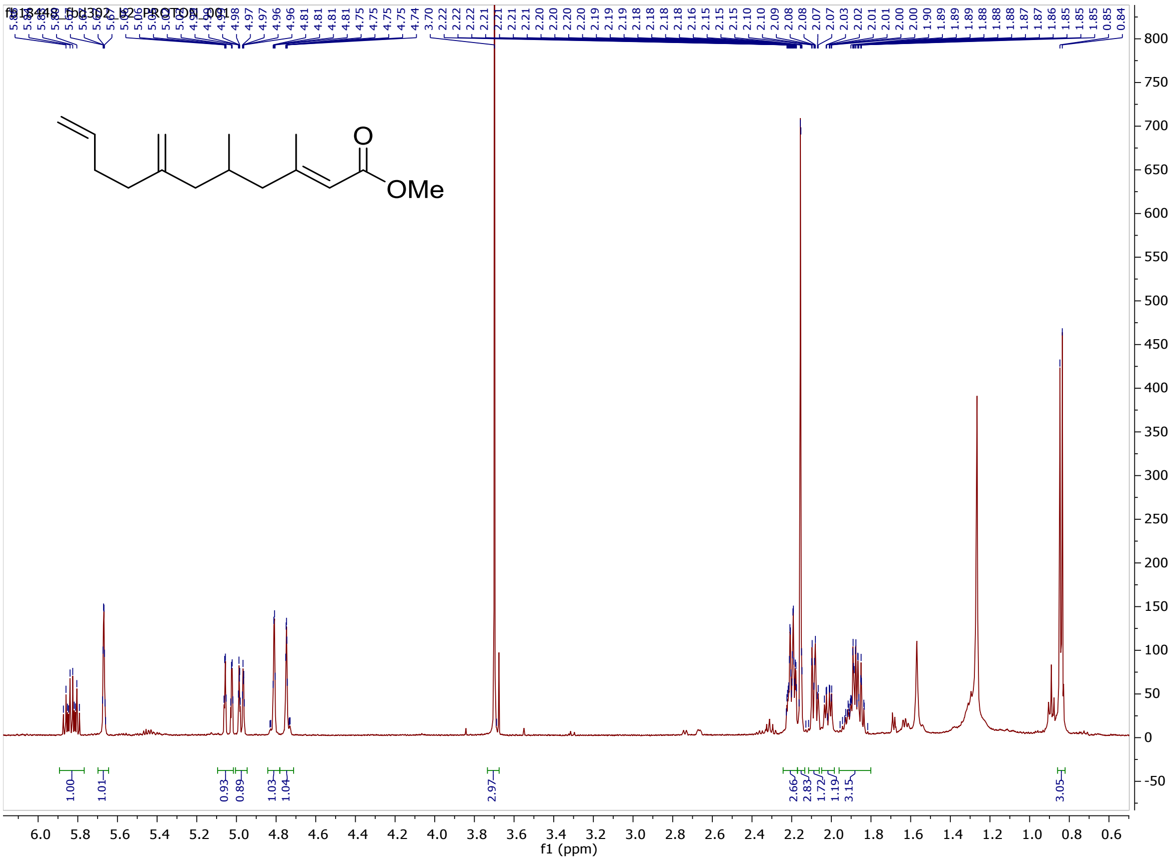

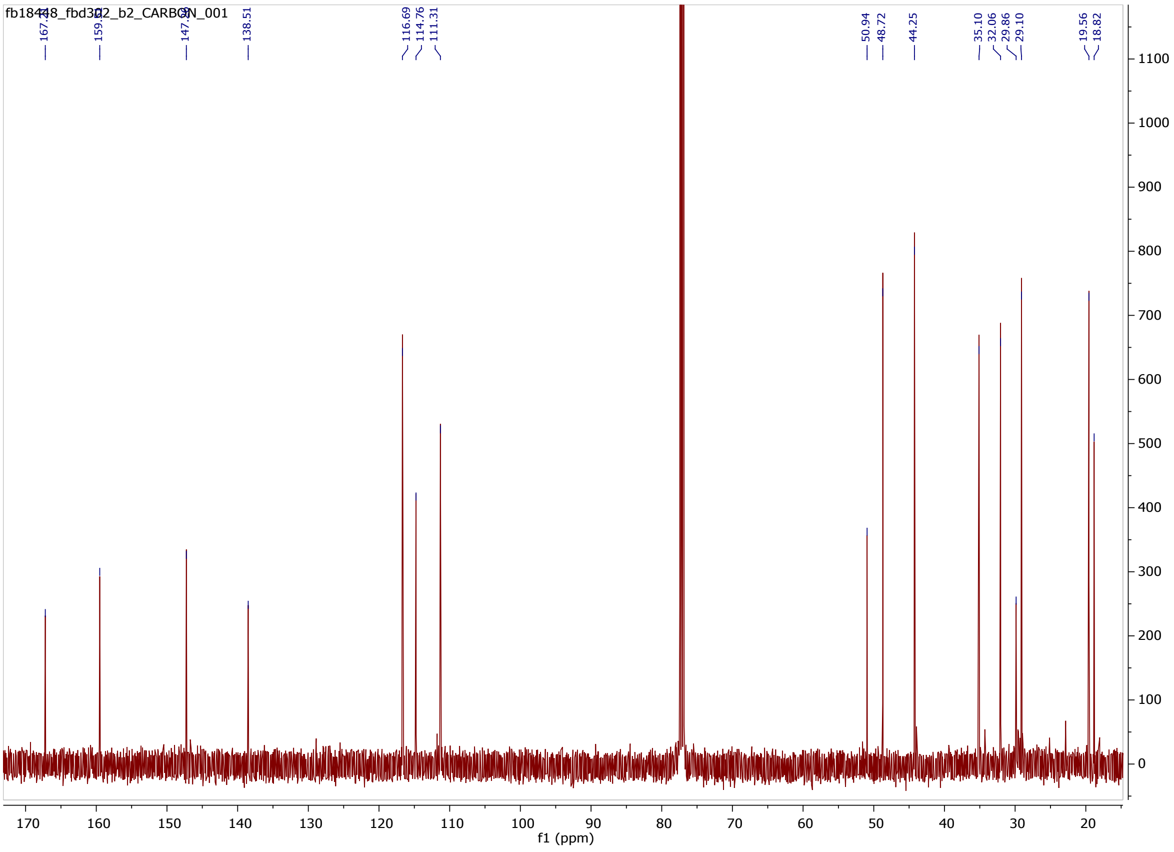

Methyl (*R*,*E*)-7-hydroxy-3,5-dimethylhept-2-enoate 33

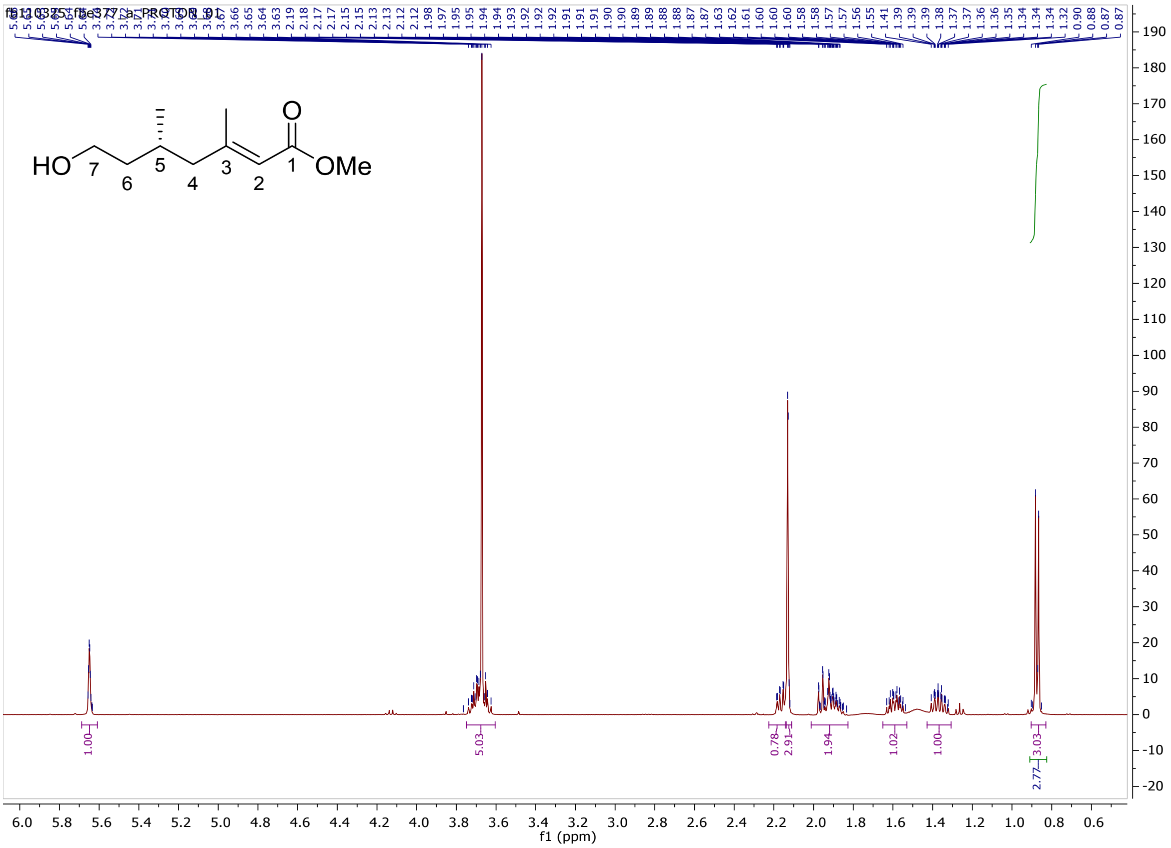

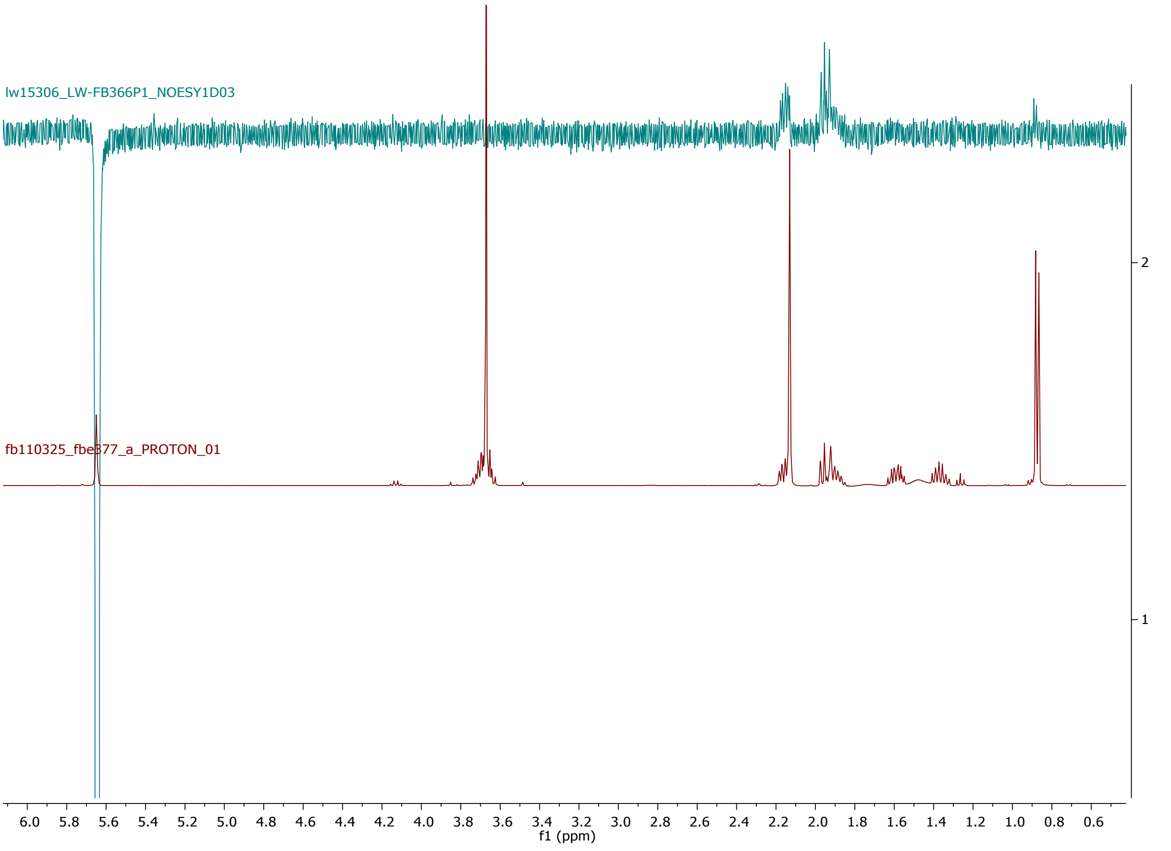

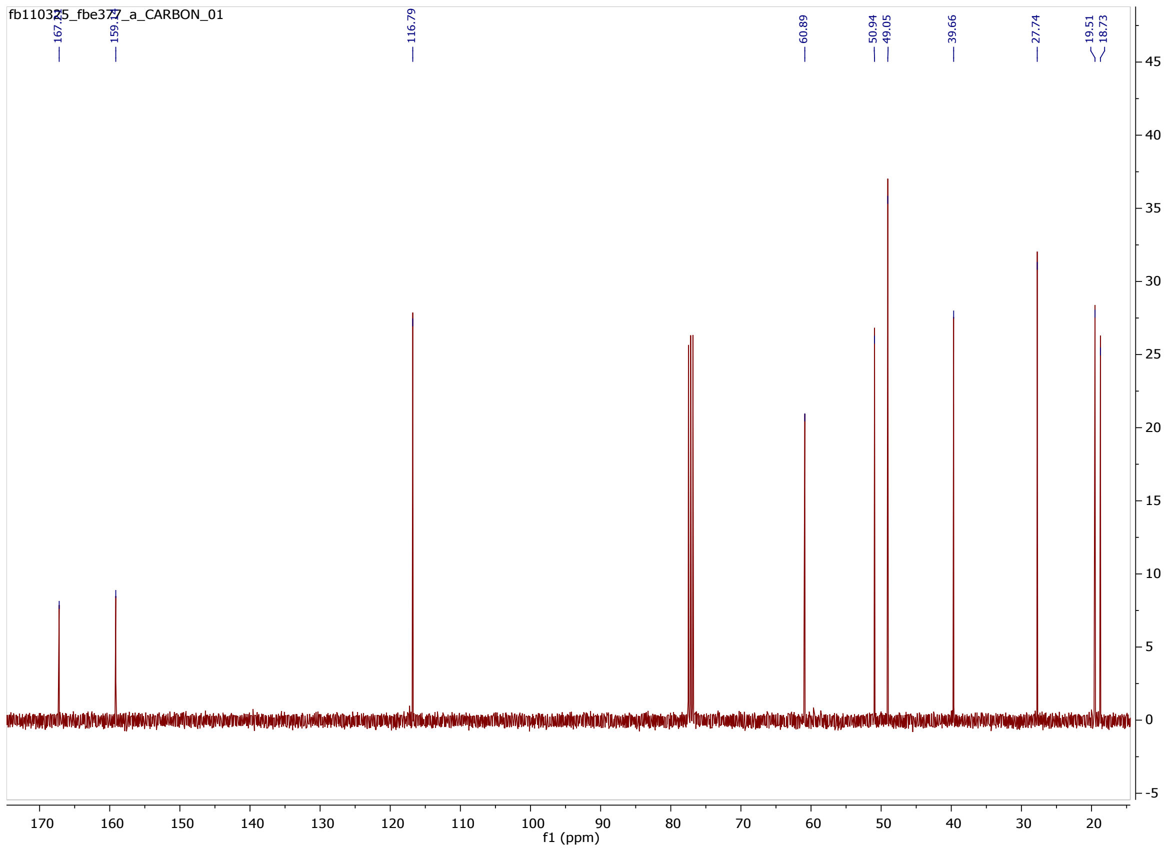
fb110325_fbe377_a_CARBON_01
116.79
60.89
50.94
49.05
39.66
27.74
19.51
18.73
45
40
35
30
25
20
15
10
5
0
-5
170
160
150
140
130
120
110
100
90
80
70
60
50
40
30
20
f1 (ppm)

Methyl (*R*,*E*)-3,5-dimethyl-7-oxoundeca-2,10-dienoate 35

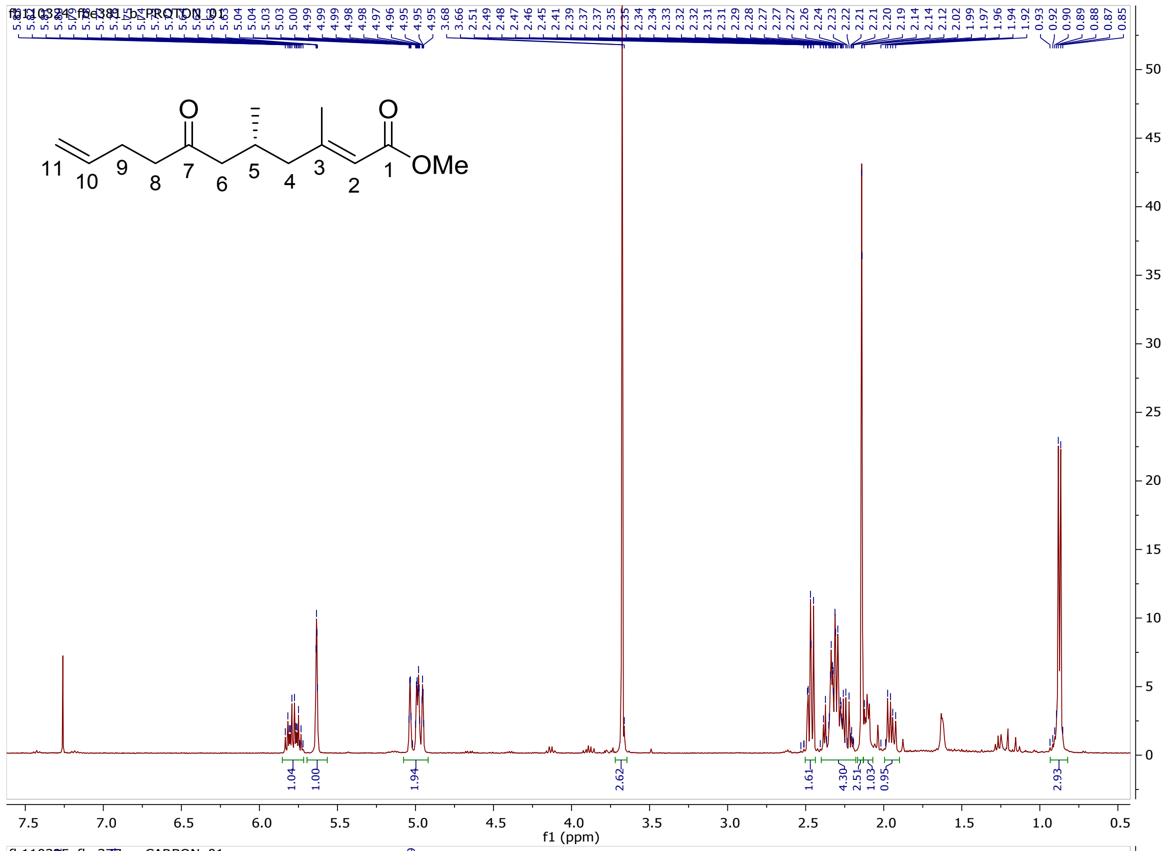

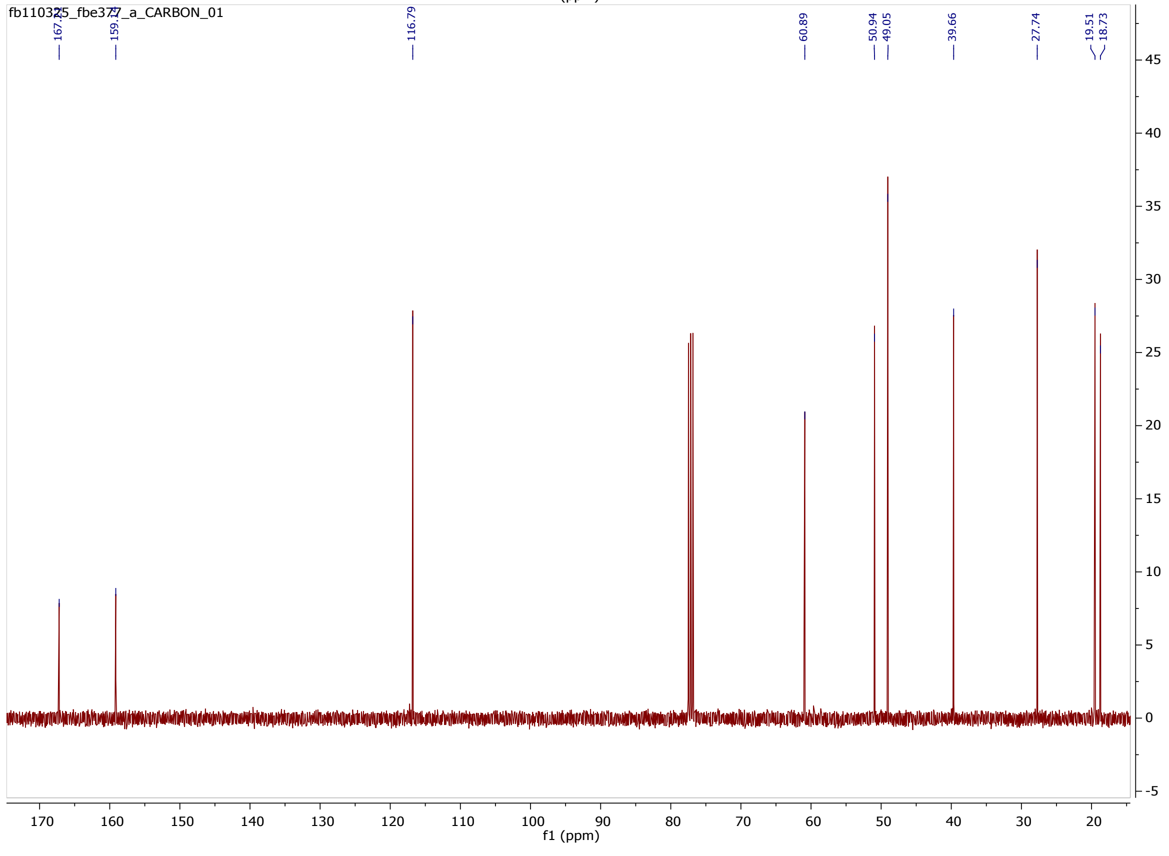

Synthetic Methyl Ester (+)-29

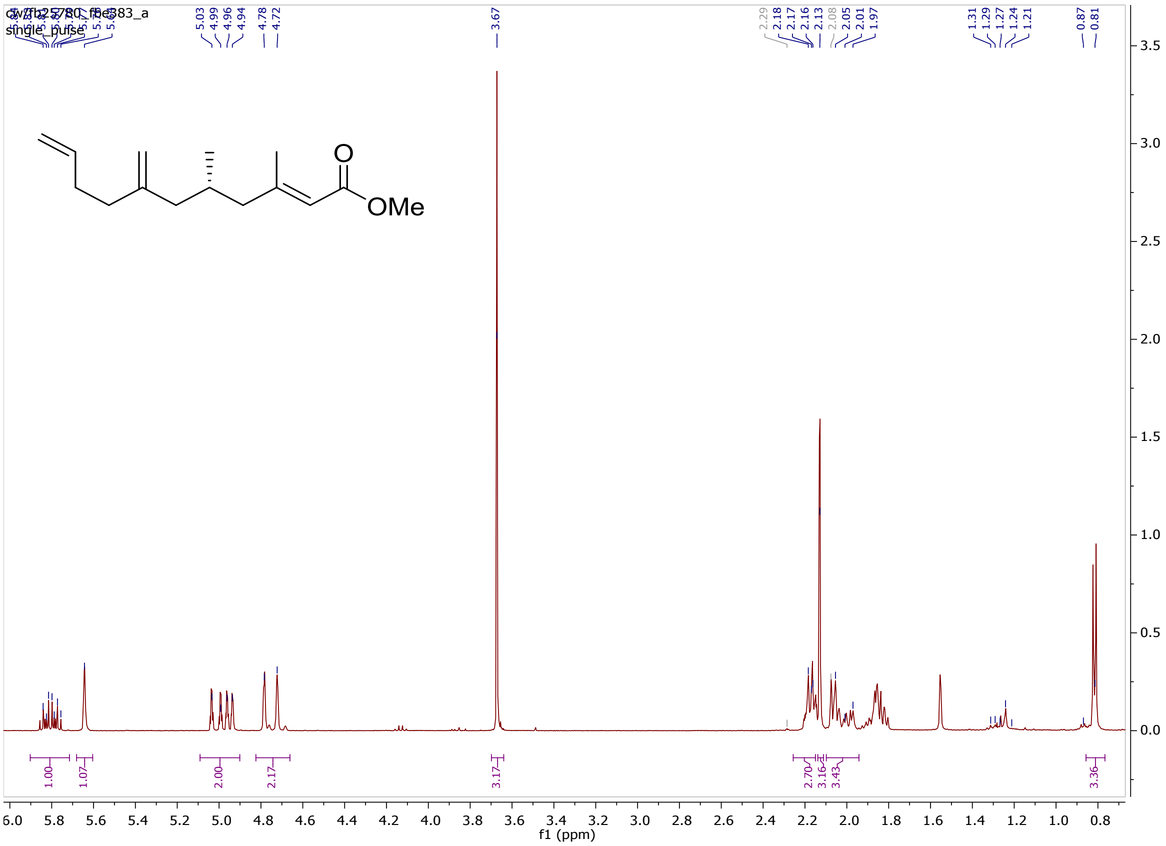

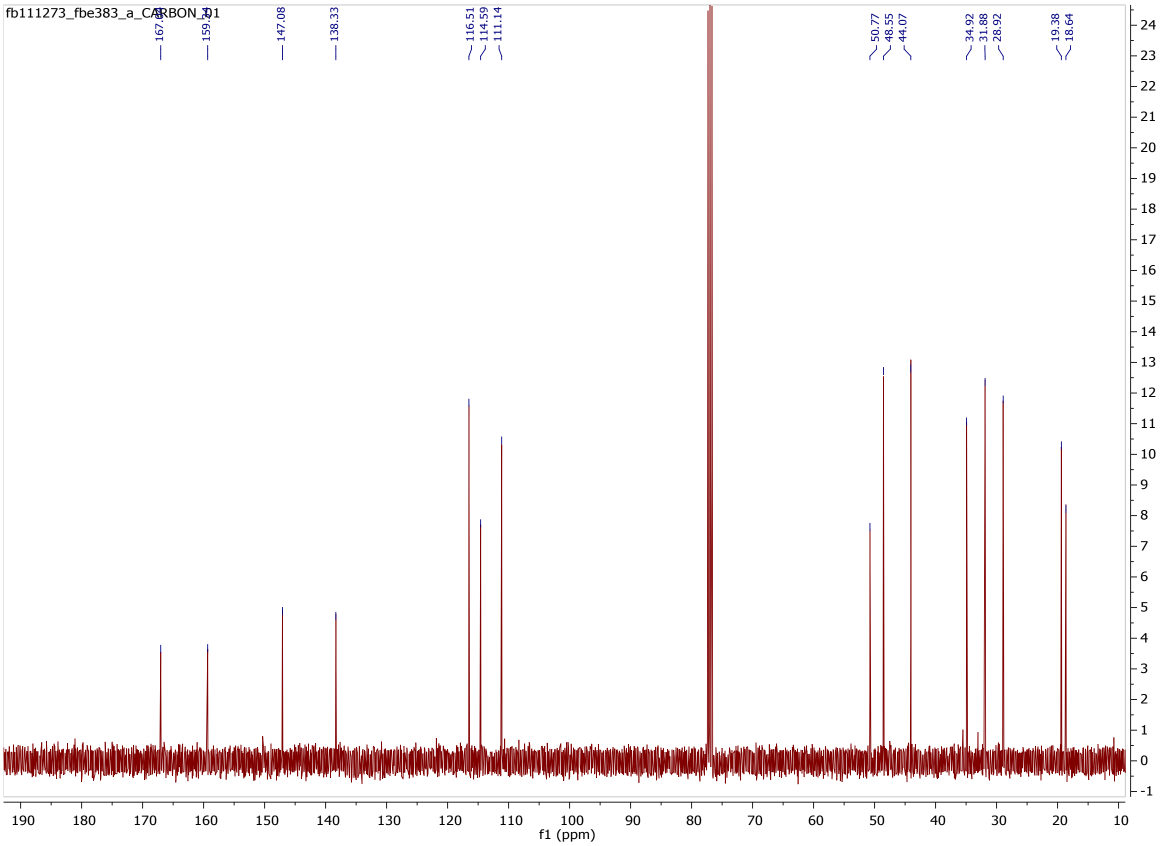

17-Hydroxy acetonide 7

^{1}H NMR (500 MHz, $CDCl_3$)

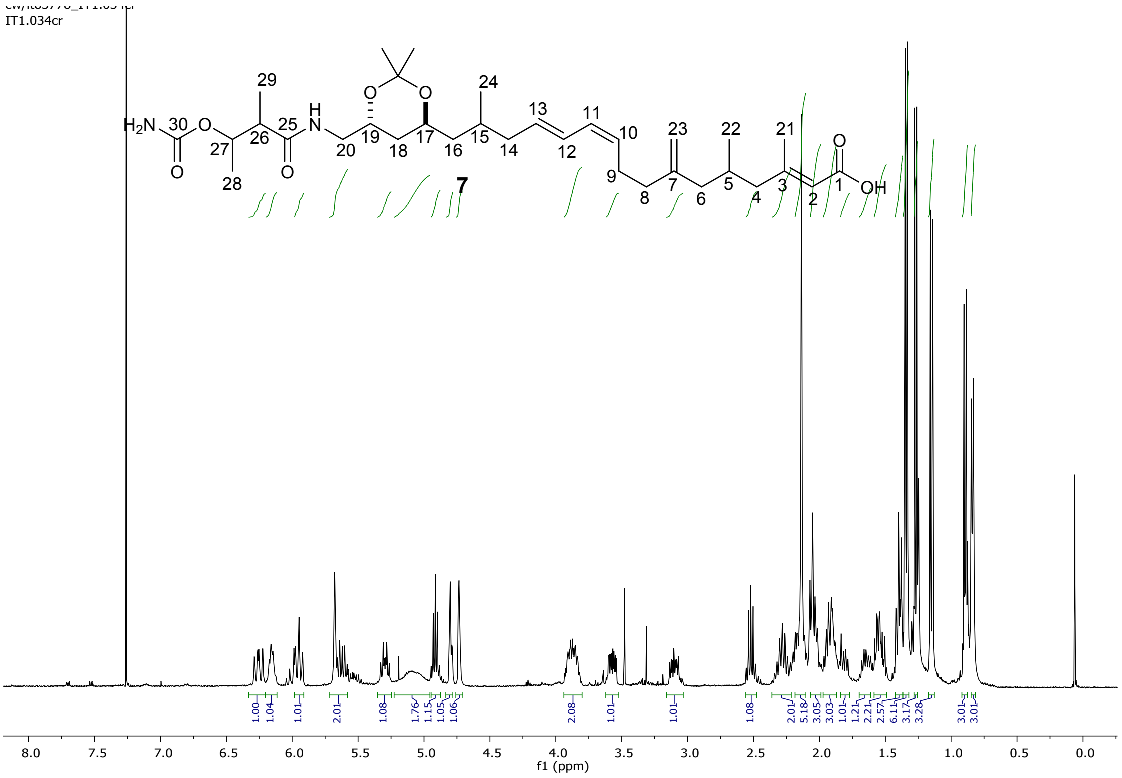

^{13}C NMR (125 MHz, $CDCl_3$)

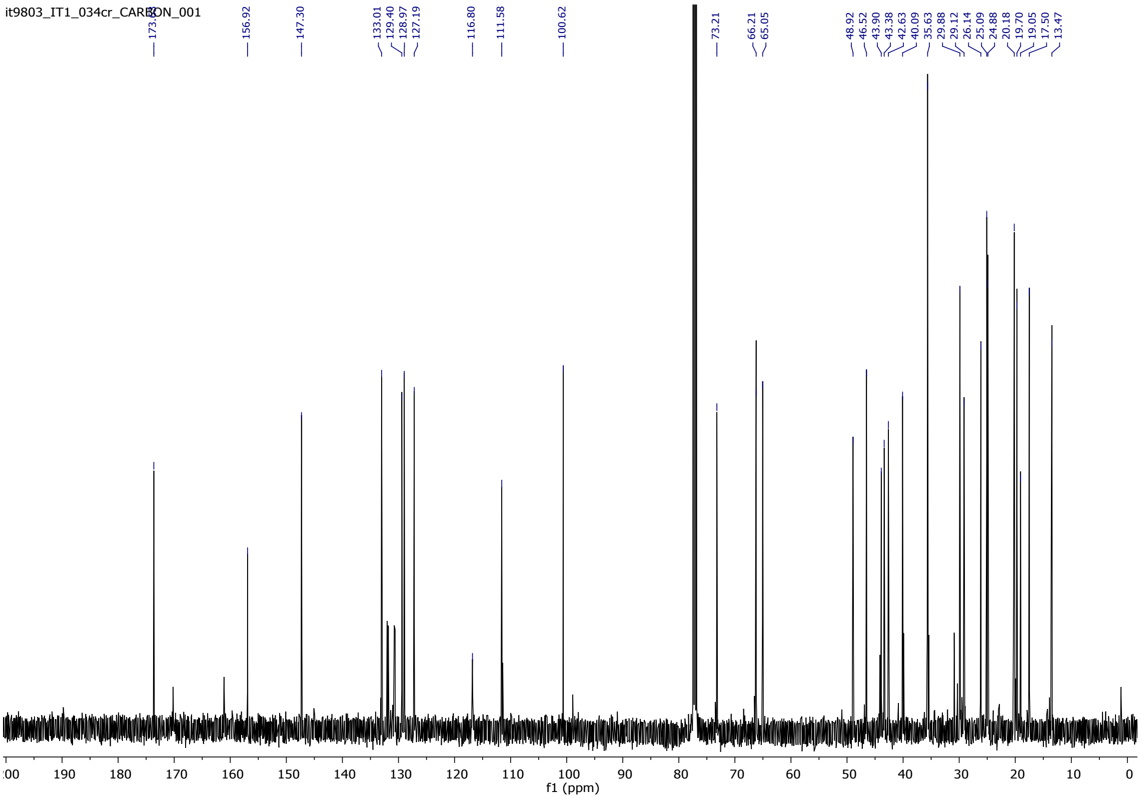

(2*S*, 3*R*)-3-(*tert*-Butyldimethylsilyloxy)-2-methylbutanoic acid S1

^{1}H NMR (400 MHz, $CDCl_3$)

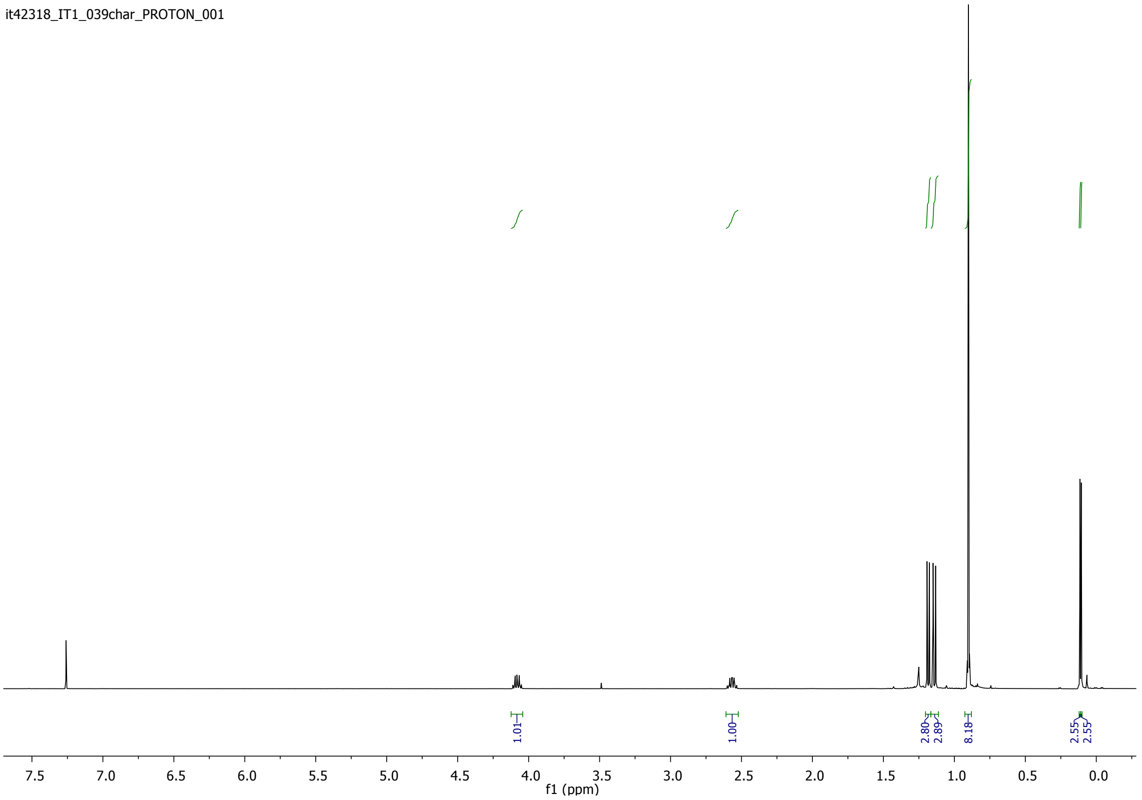

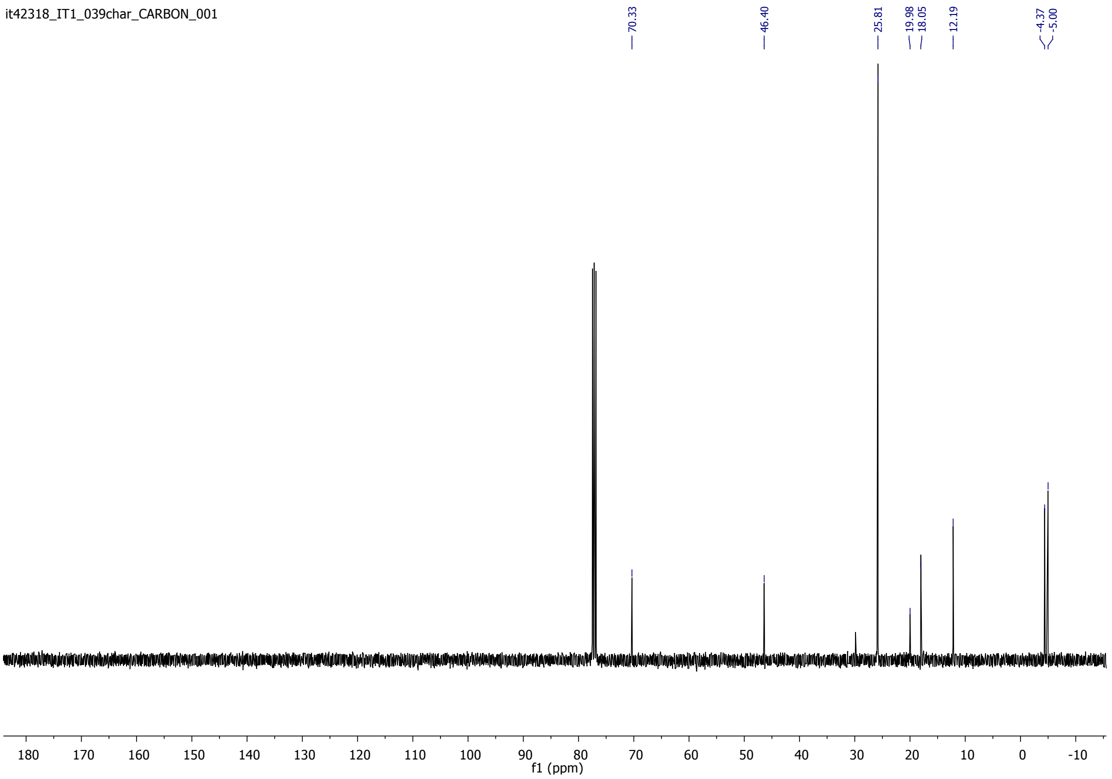

(2*S*, 3*R*)-3-(*tert*-Butyldimethylsilyloxy)-*N*-((*R*)-2'-hydroxypent-4'-en-1-yl)-2-methylbutanamide S16

^{1}H NMR (400 MHz, $CDCl_3$)

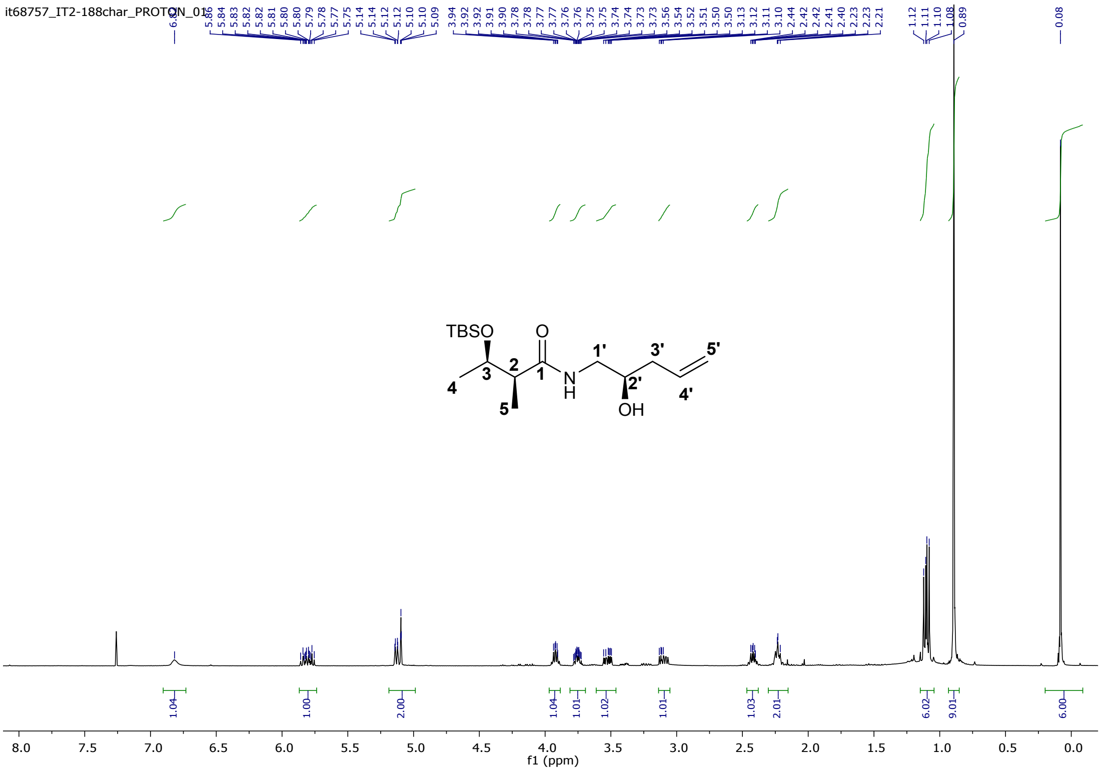

^{13}C NMR (125 MHz, $CDCl_3$)

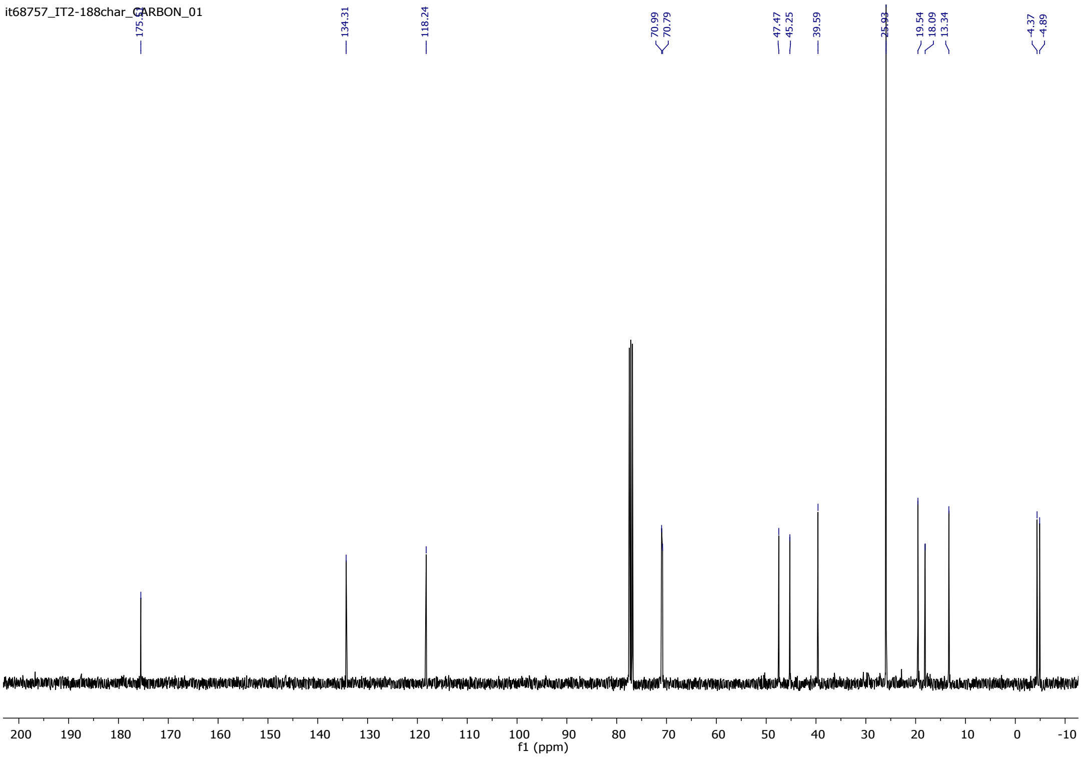

(2*S*, 3*R*)-3-Hydroxy-*N*-((*R*)-2'-hydroxy-4'-oxopentyl)-2-methylbutanamide 9

^{1}H NMR (500 MHz, acetone-d$_6$)

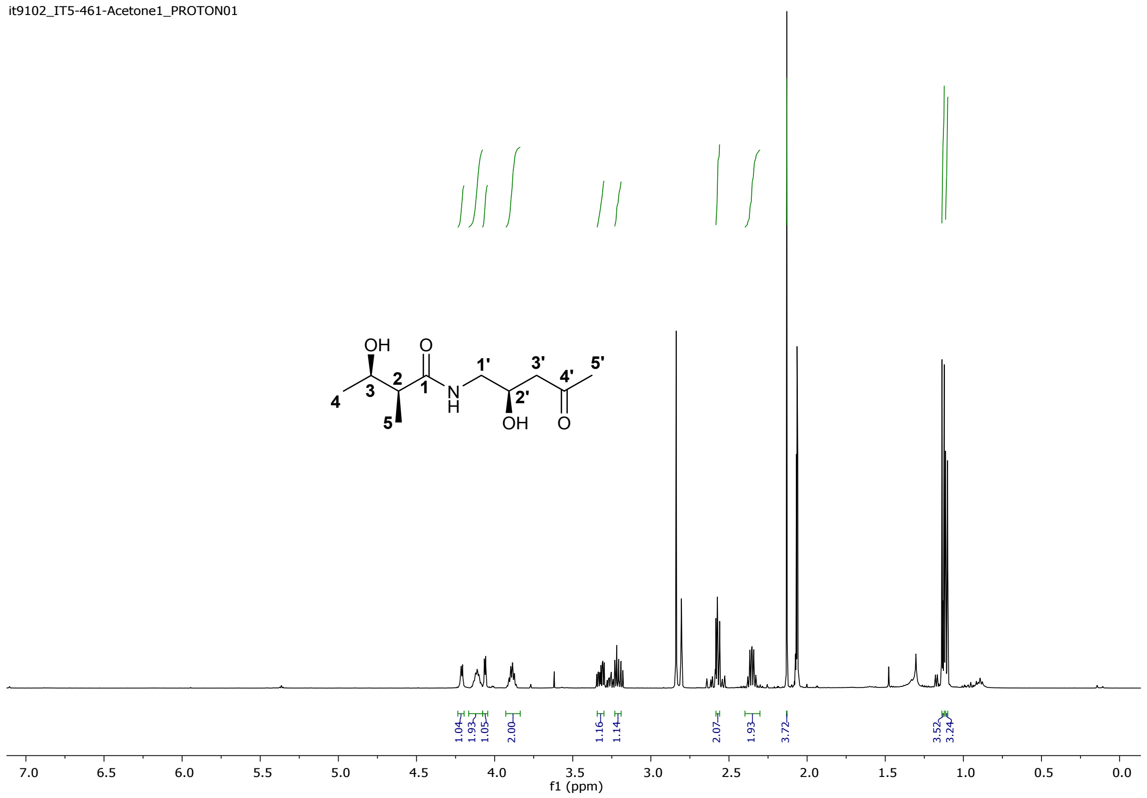

^{13}C NMR (125 MHz, acetone-d$_6$)

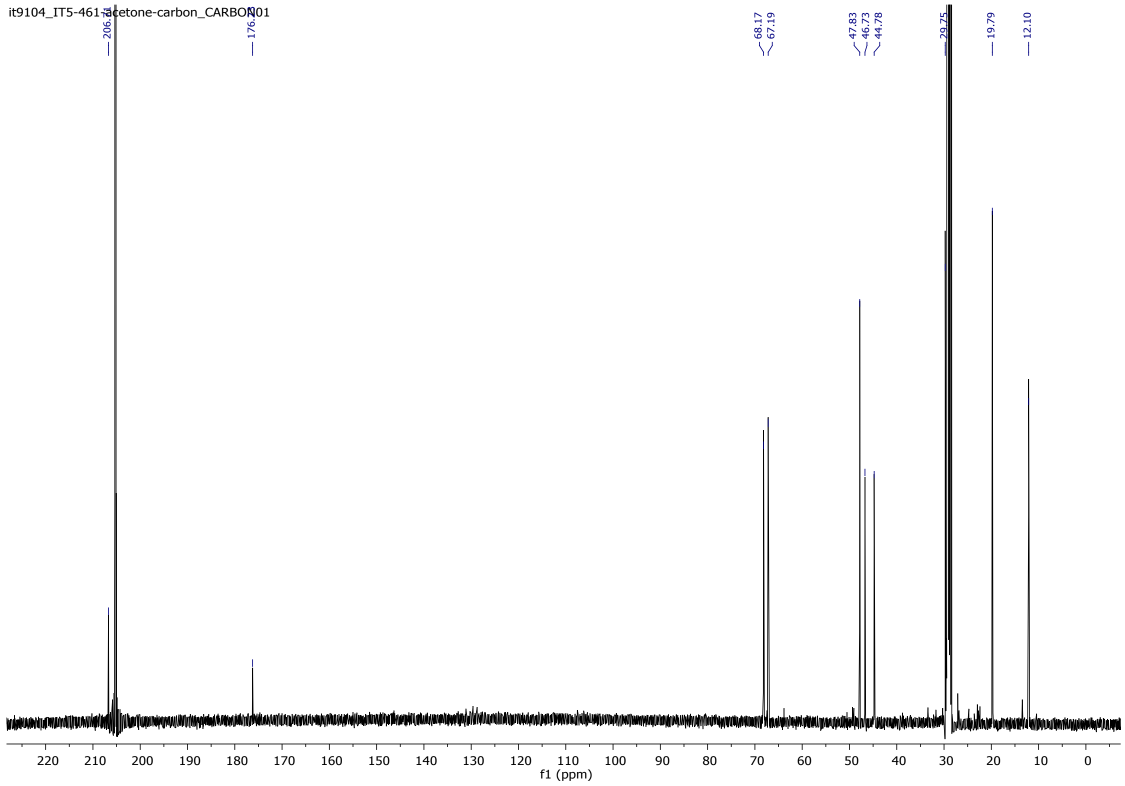

Isolated lactone 10 from ozonolysis of 2

^{1}H NMR (500 MHz, methanol-d_4)

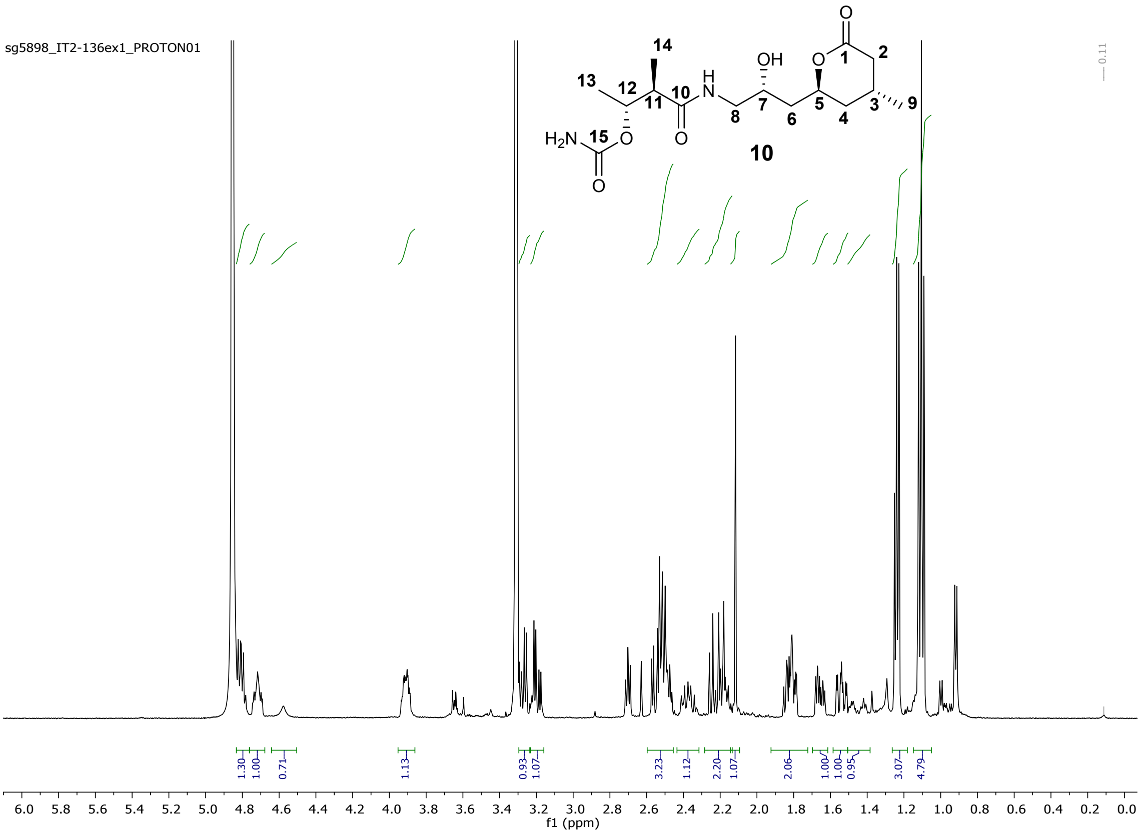

^{13}C NMR (125 MHz, methanol-d4)

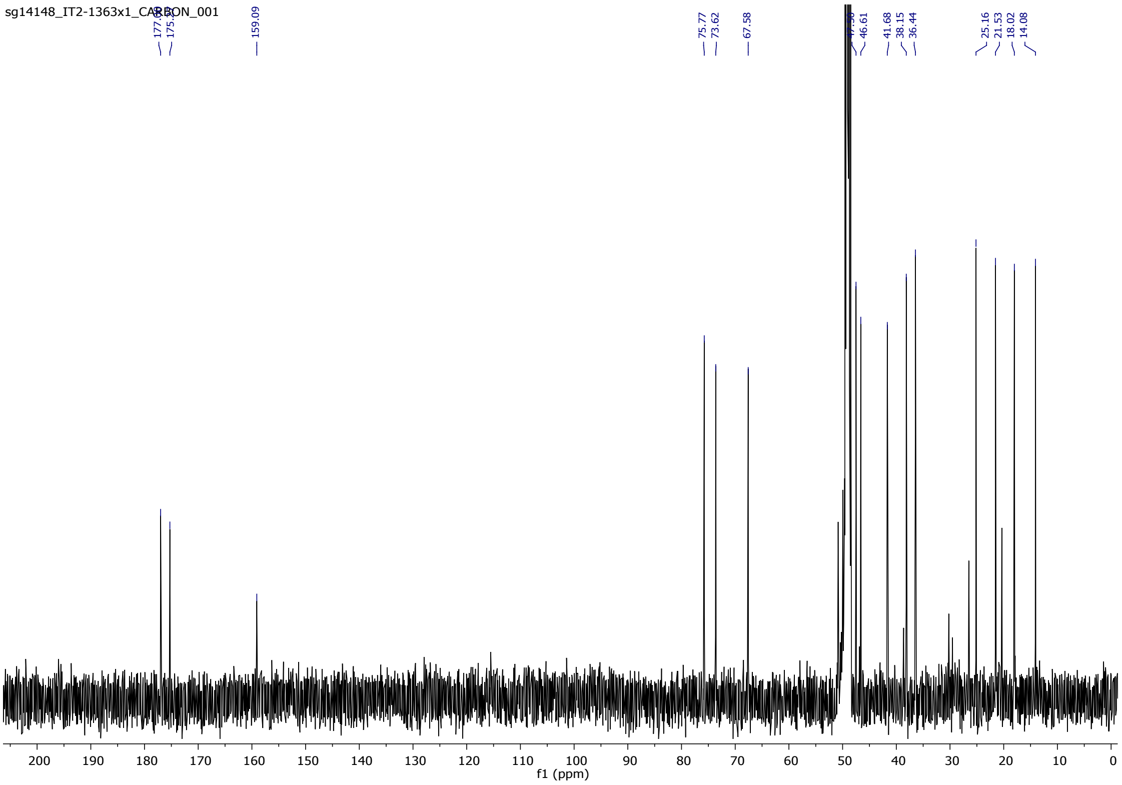

Synthetic lactone 10

^{1}H NMR (500 MHz, Methanol-d_4)

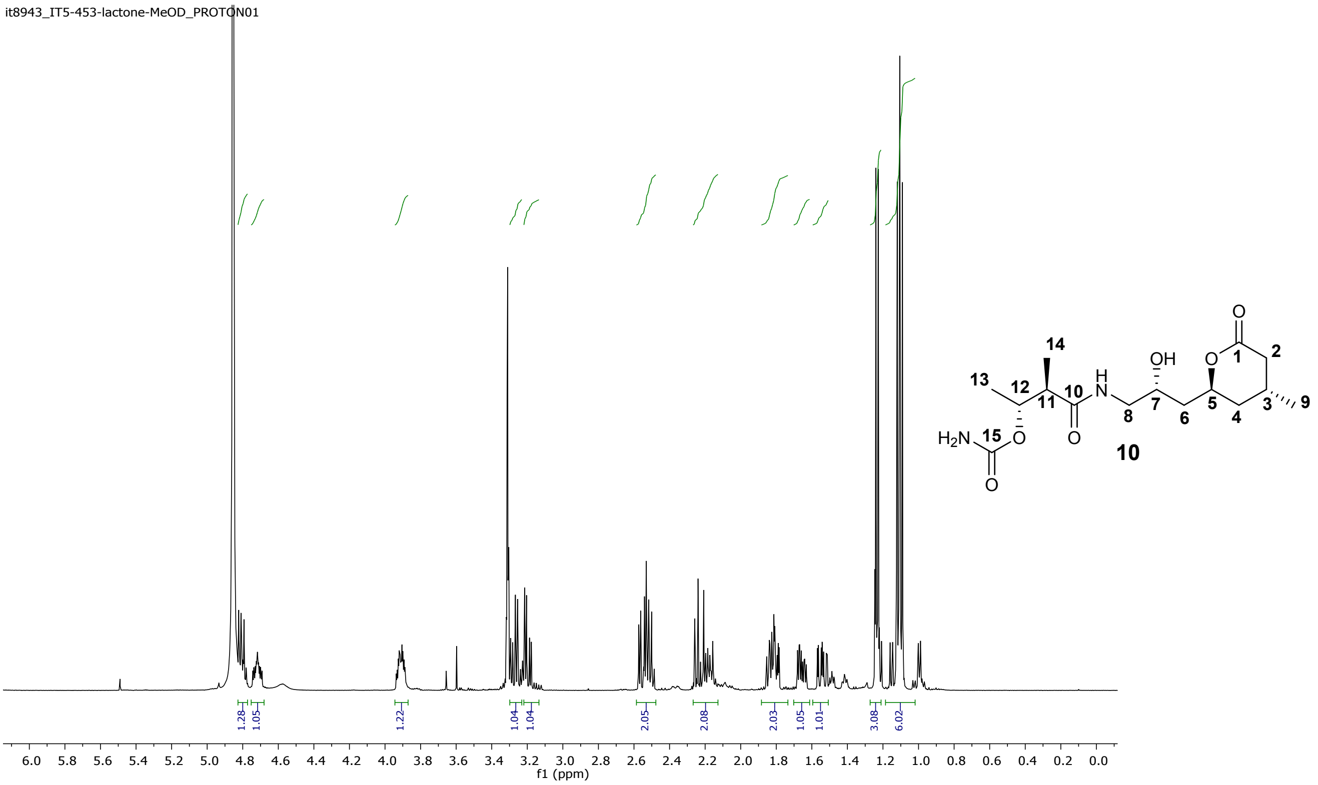

^{13}C NMR (125 MHz, methanol-d_4)

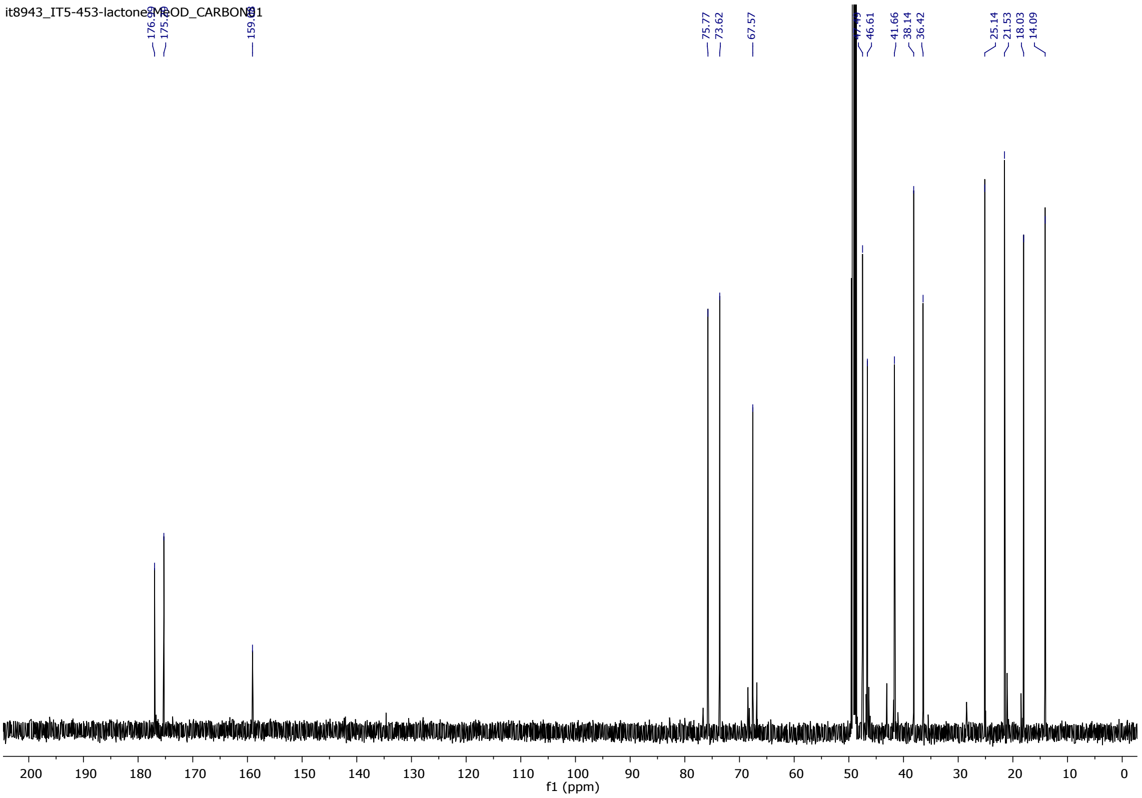

(1*R*, 2*S*)-*N*-[(3'*R*)-5'-*tert*-Butyldimethylsilyloxy-3'-methylhexan-5'-ol]-bornane-10,2-sultam S21

^{1}H NMR (400 MHz, $CDCl_3$)

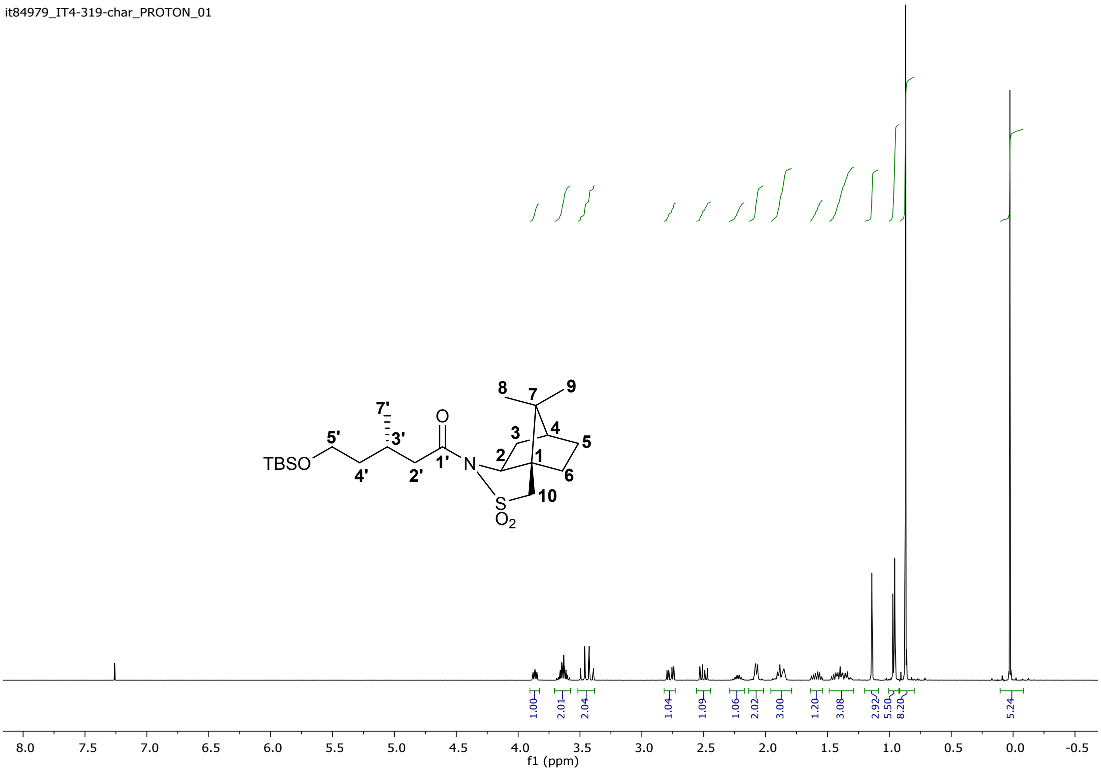

^{13}C NMR (125 MHz, $CDCl_3$)

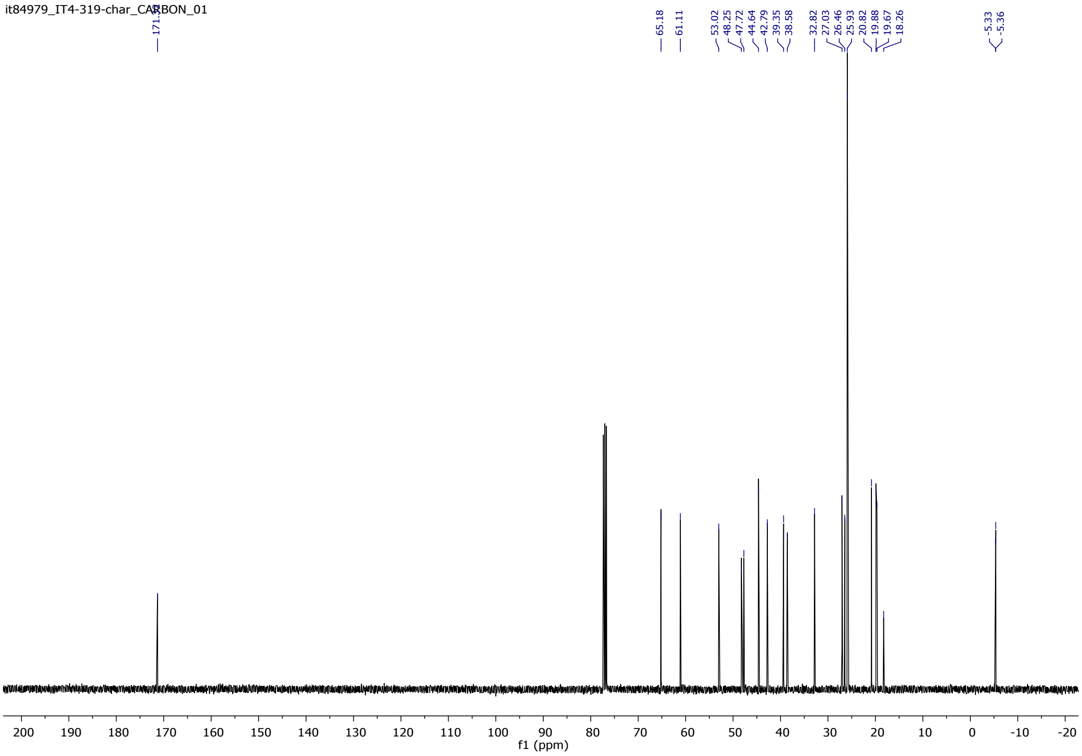

(4*R*, 6*S*)-8-((*tert*-butyldimethylsilyl)oxy)-6-methyloct-1-en-4-ol 16

^{1}H NMR (400 MHz, $CDCl_3$)

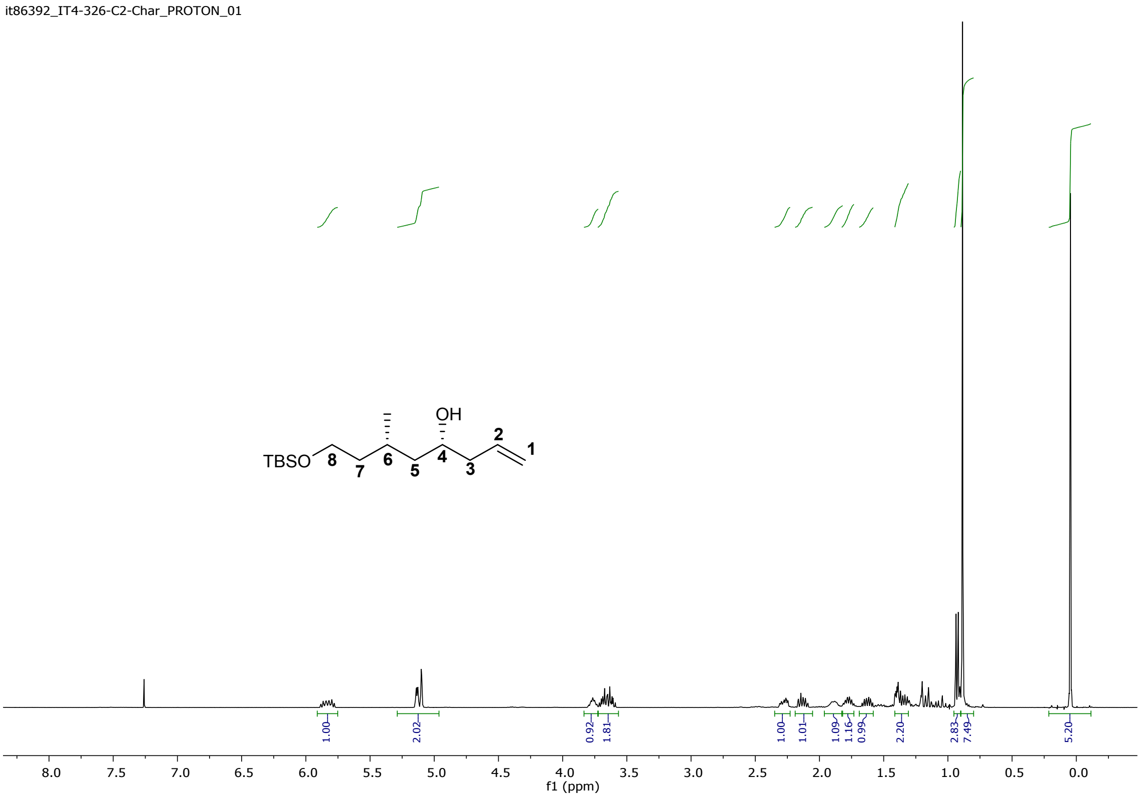

^{13}C NMR (125 MHz, $CDCl_3$)

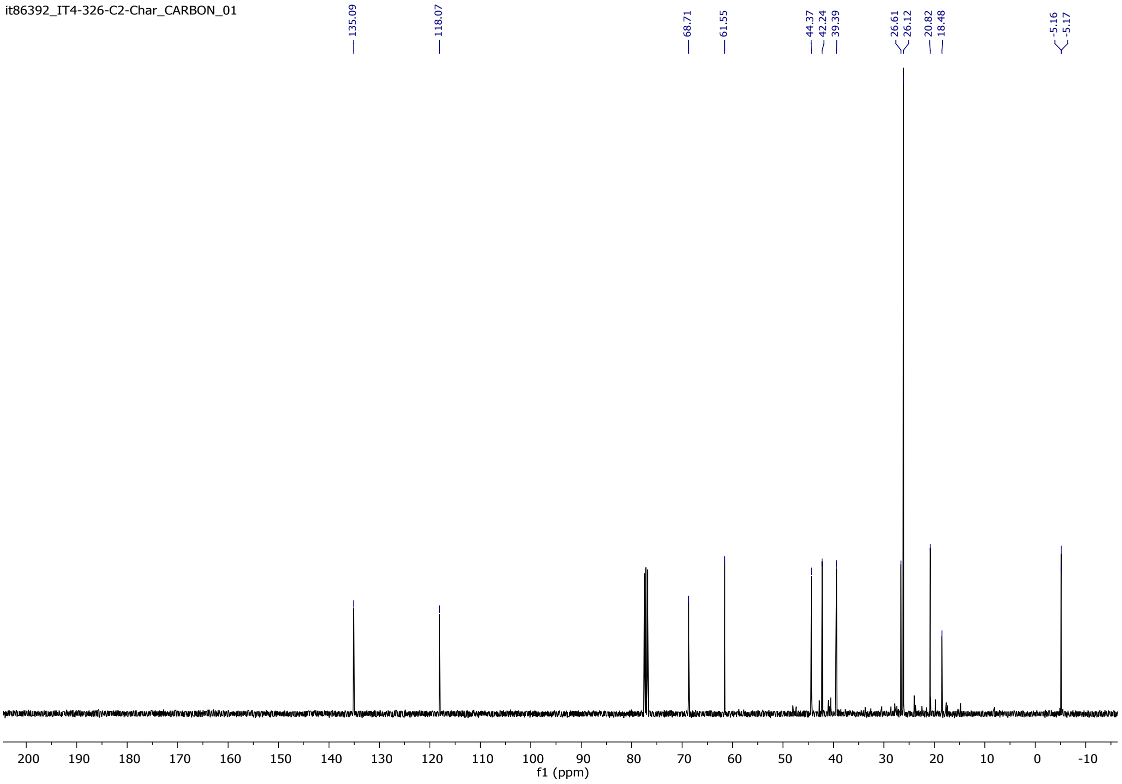

(4*S*, 6*S*)-4,8-Di(*tert*-butyldimethylsilyloxy)-6-methyl-oct-1-ene S24

^{1}H NMR (400 MHz, $CDCl_3$)

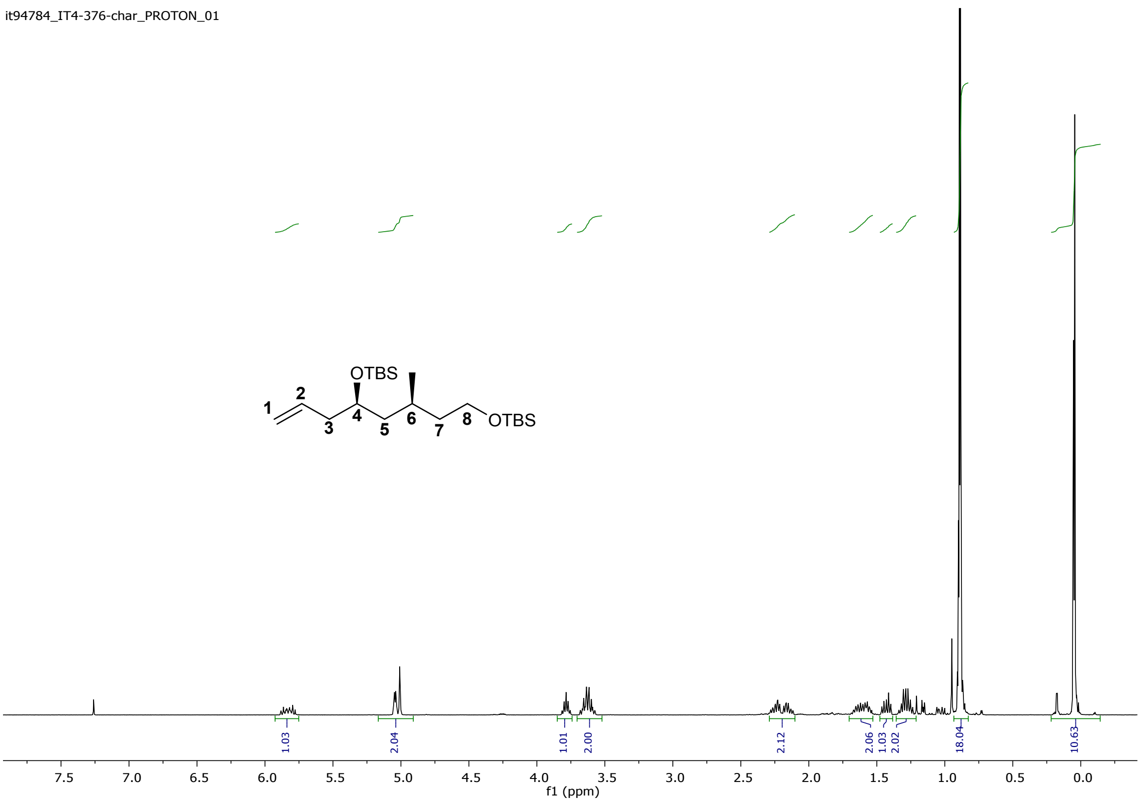

^{13}C NMR (125 MHz, $CDCl_3$)

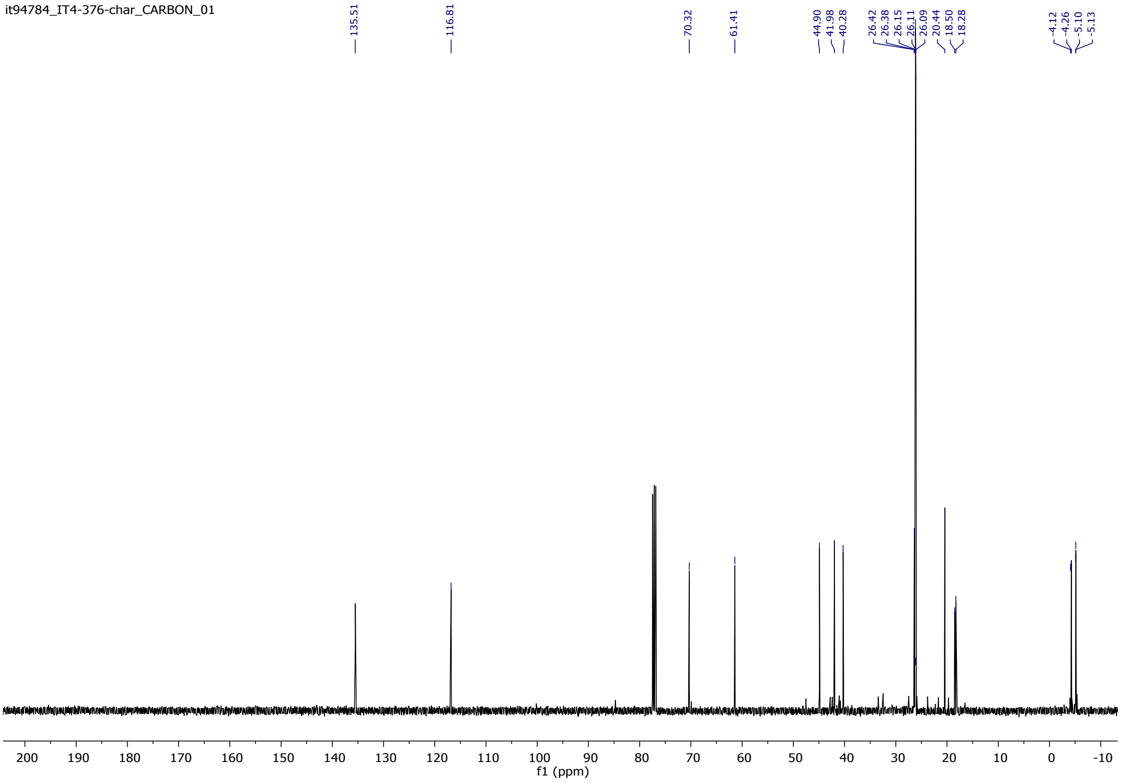

(2*R*, 4*S*, 6*S*)-4,8-Di(*tert*-butyldimethylsilyloxy)-1,2-epoxy-6-methyloctane 17 and (2*S*, 4*S*, 6*S*)-4,8-di(*tert*-butyldimethylsilyloxy)-1,2-epoxy-6-methyloctane 18

^{1}H NMR (400 MHz, $CDCl_3$)

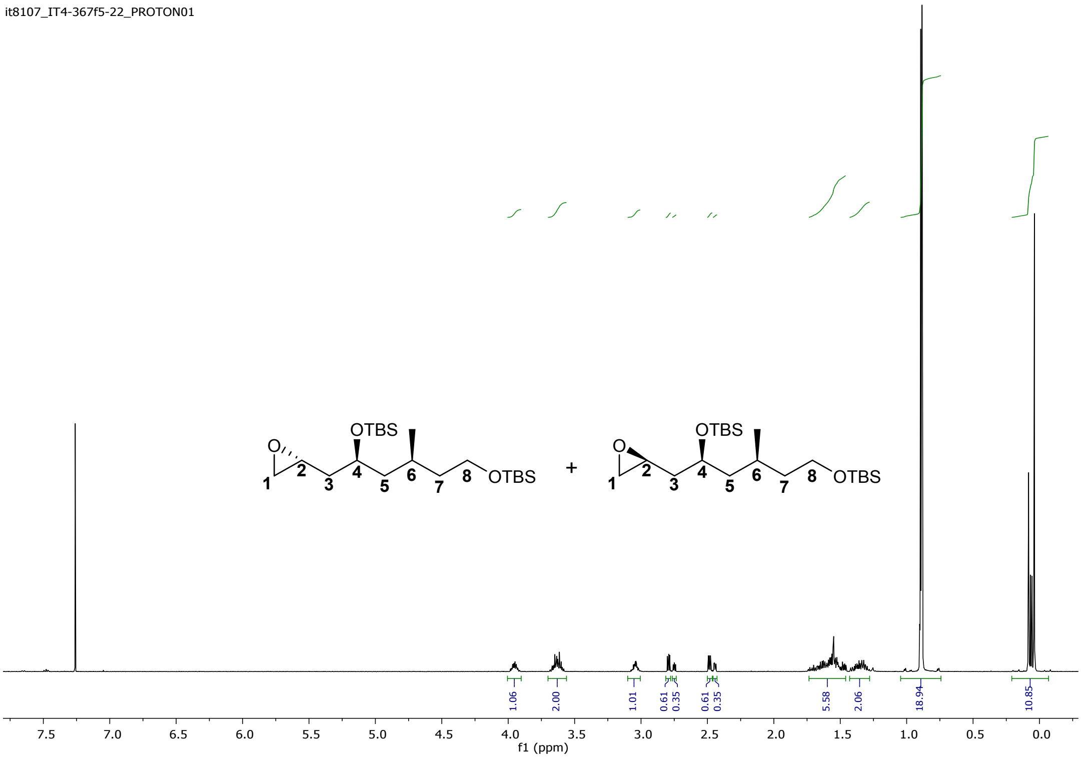

^{13}C NMR (125 MHz, $CDCl_3$)

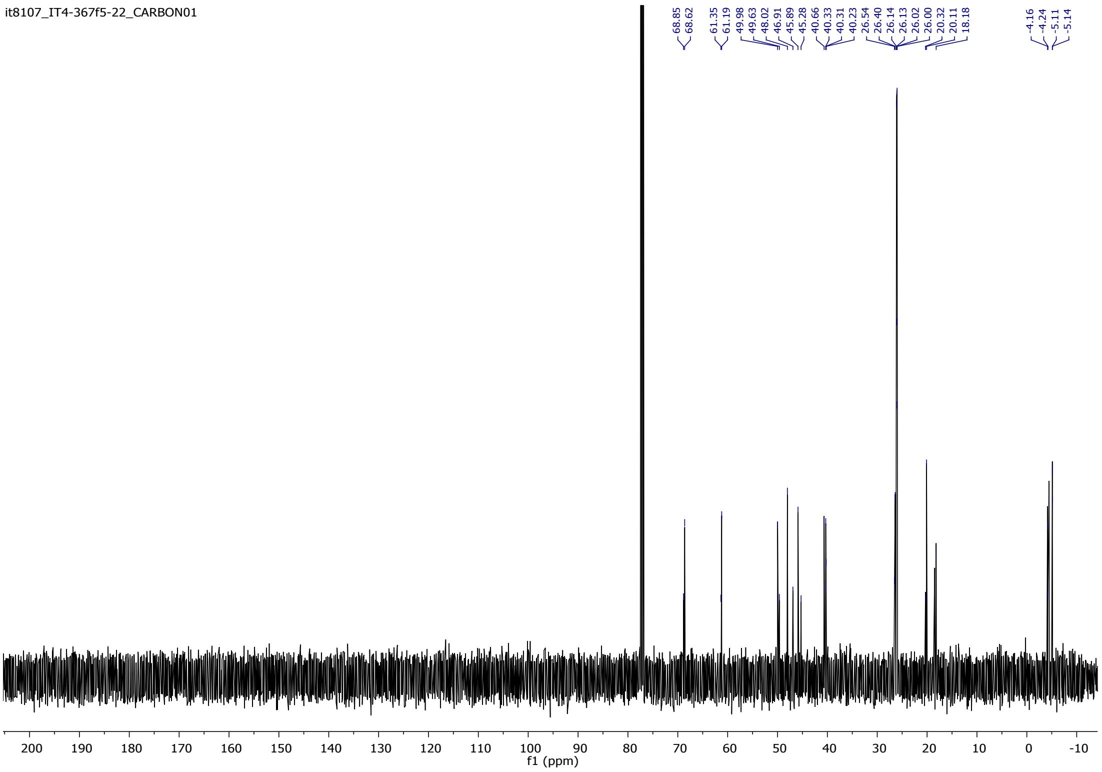

(2*R*, 4*S*, 6*S*)-1-Azido-4,8-di(*tert*-butyldimethylsilyloxy)-6-methyloctan-2-ol 20

^{1}H NMR (400 MHz, $CDCl_3$)

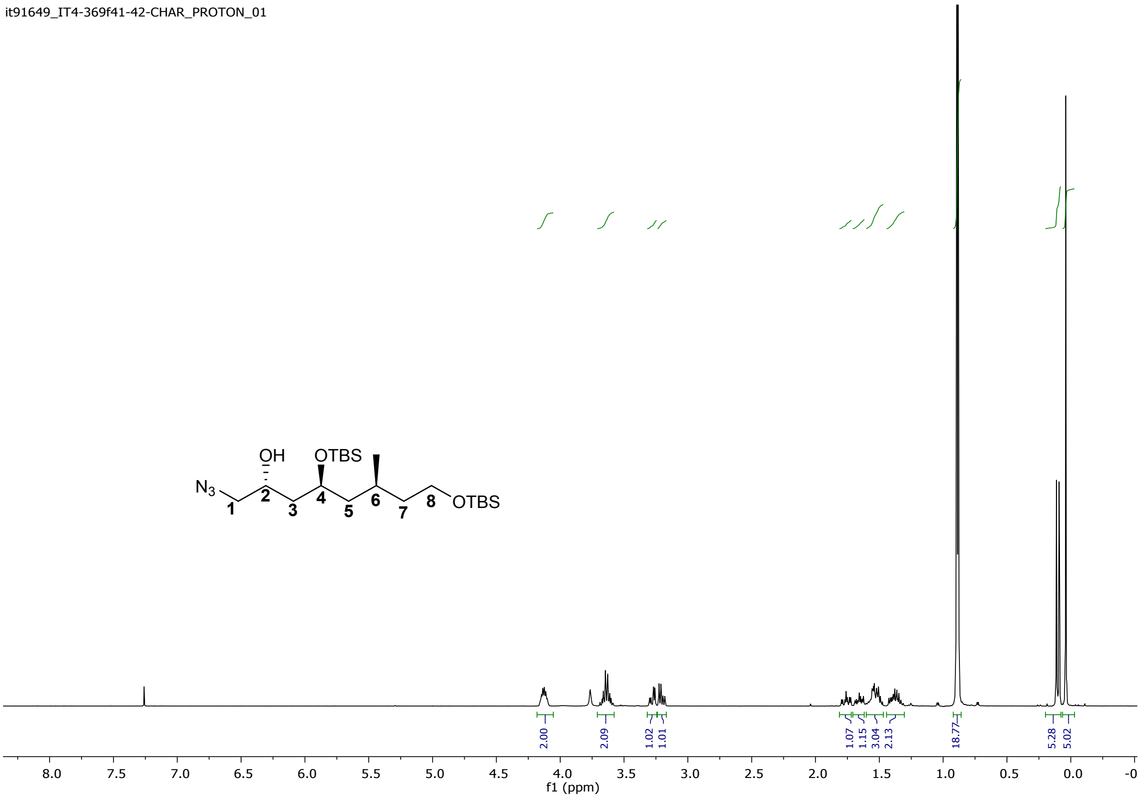

^{13}C NMR (125 MHz, $CDCl_3$)

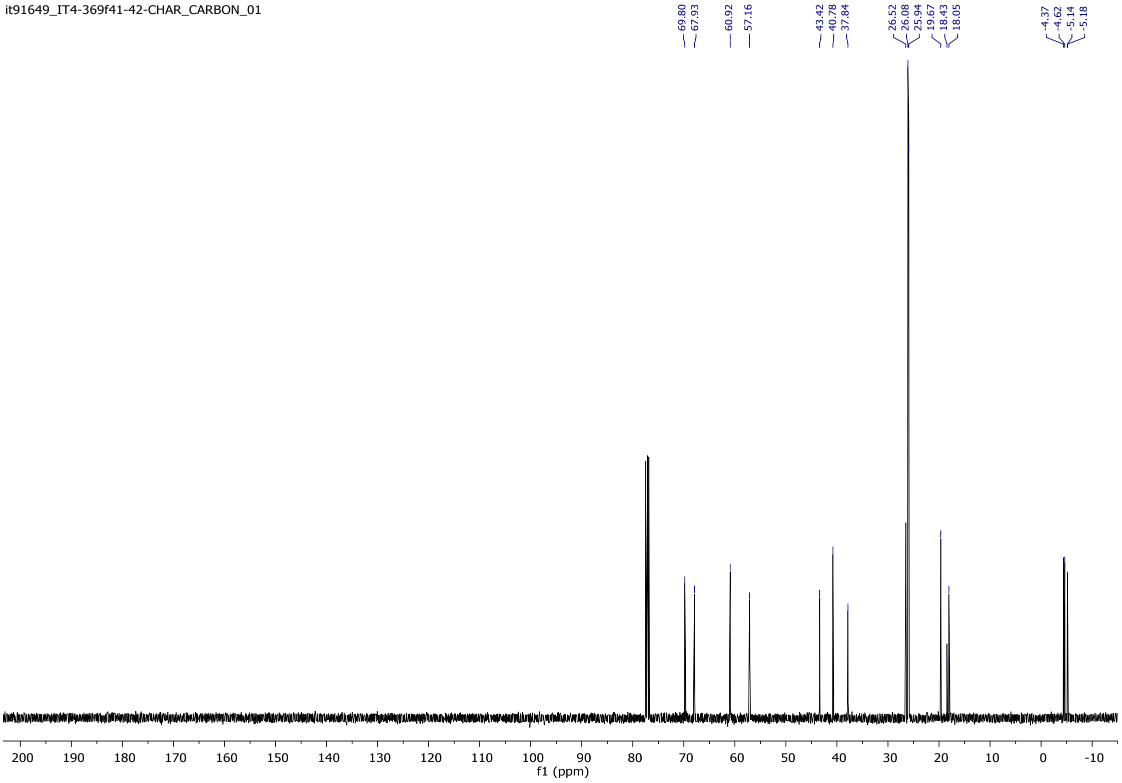

(2*S*, 4*S*, 6*S*)-4,8-di(*tert*-butyldimethylsilyloxy)-1,2-epoxy-6-methyloctane 18

^{1}H NMR (400 MHz, $CDCl_3$)

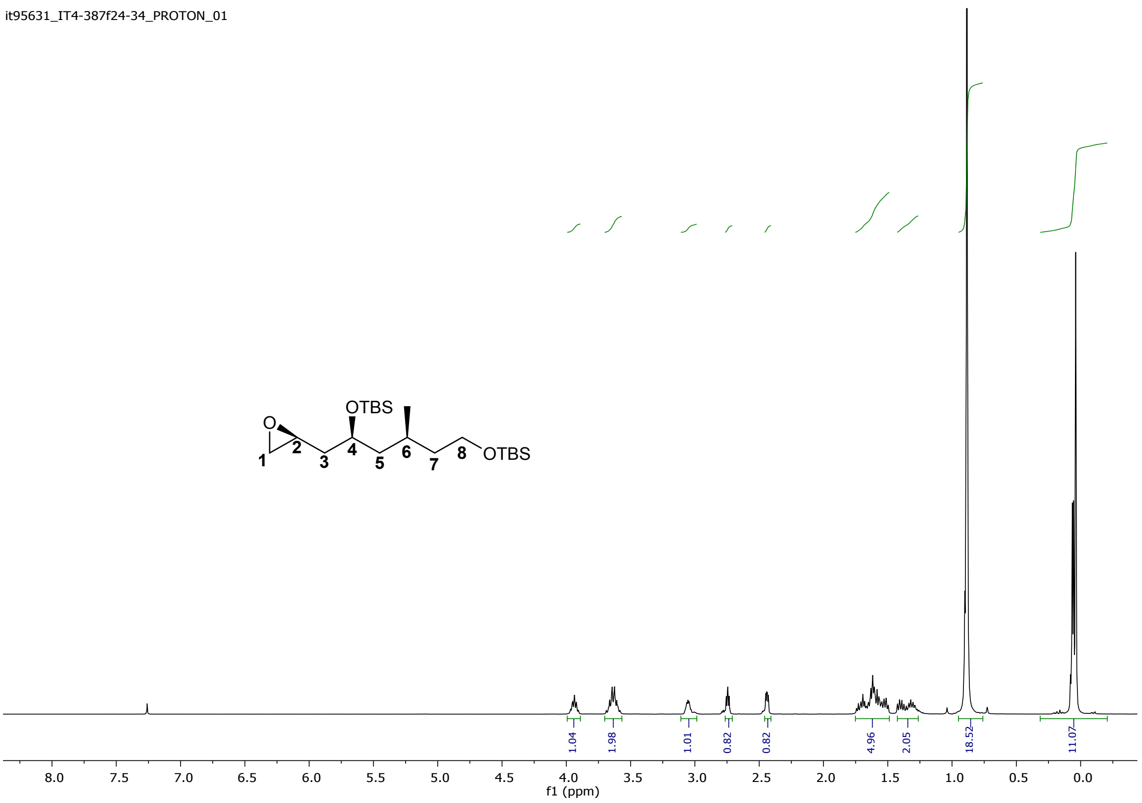

^{13}C NMR (125 MHz, $CDCl_3$)

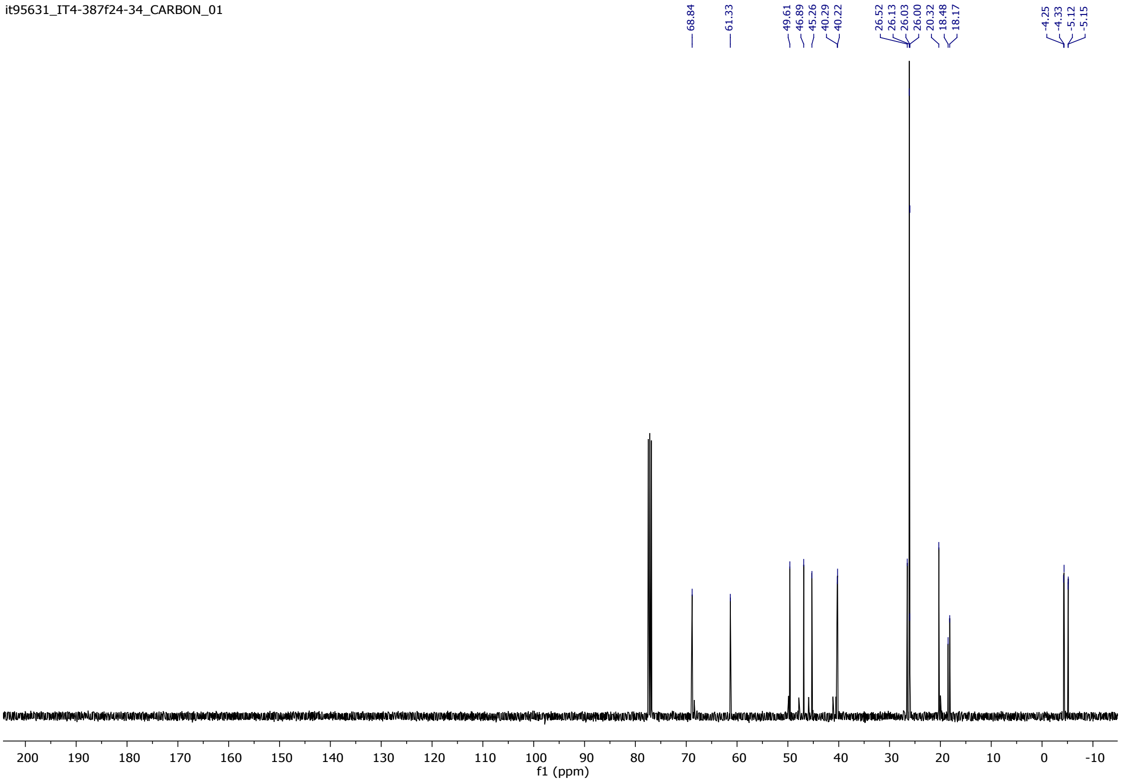

(2*R*, 4*S*, 6*S*)-1-Azido-2,4,8-tri*(tert*-butyldimethylsilyloxy)-6-methyloctanane S26

^{1}H NMR (400 MHz, $CDCl_3$)

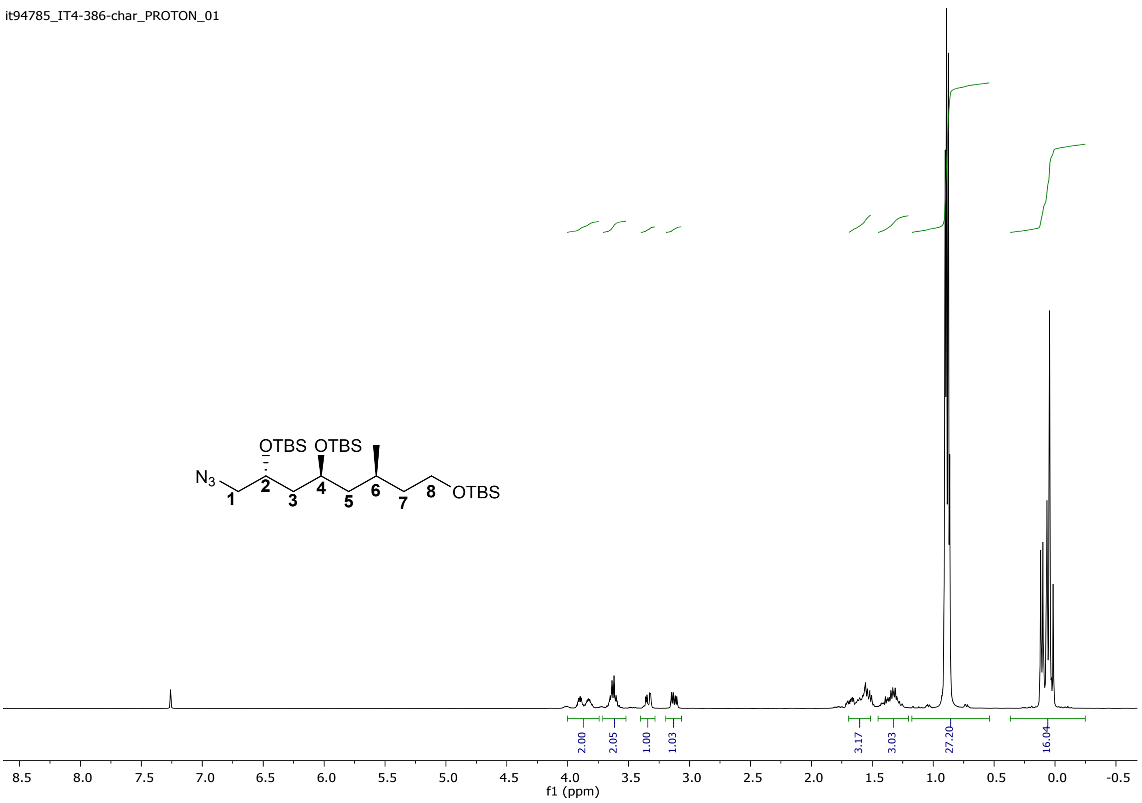

^{13}C NMR (125 MHz, $CDCl_3$)

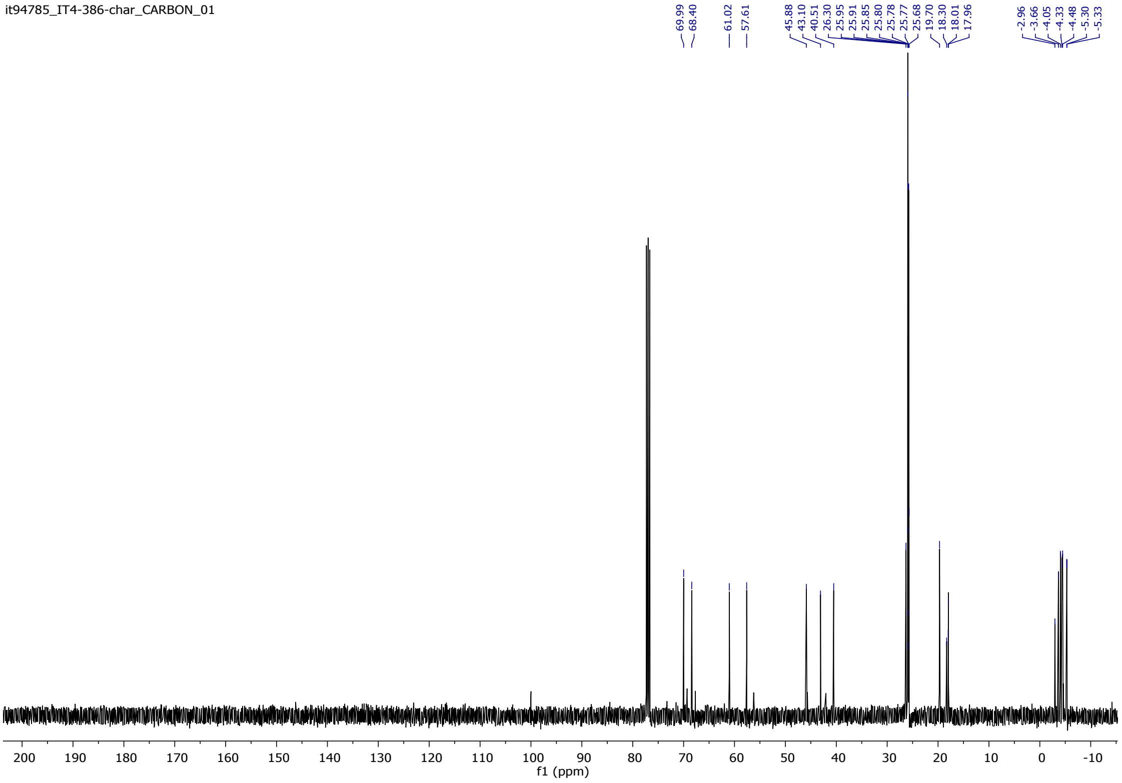

(2*R*, 4*S*, 6*S*)-1-Amino-2,4,8-tri*(tert*-butyldimethylsilyloxy)-6-methyloctanane 22

^{1}H NMR (400 MHz, $CDCl_3$)

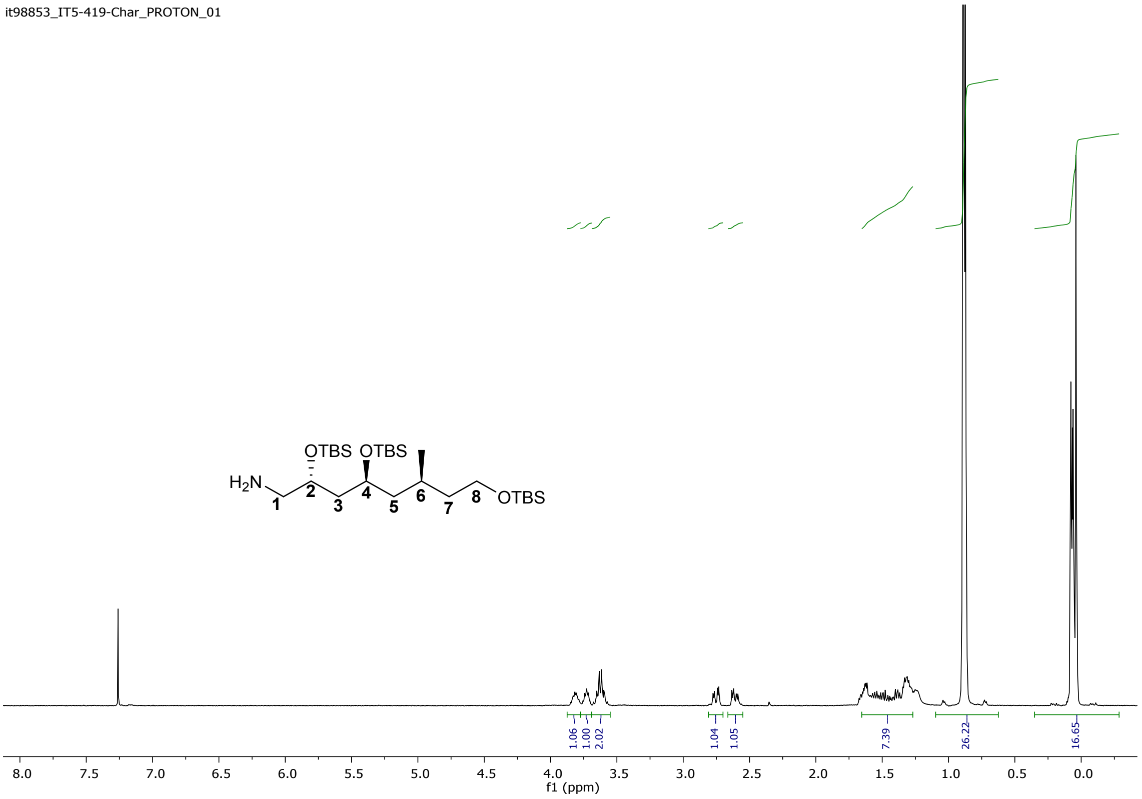

^{13}C NMR (125 MHz, $CDCl_3$)

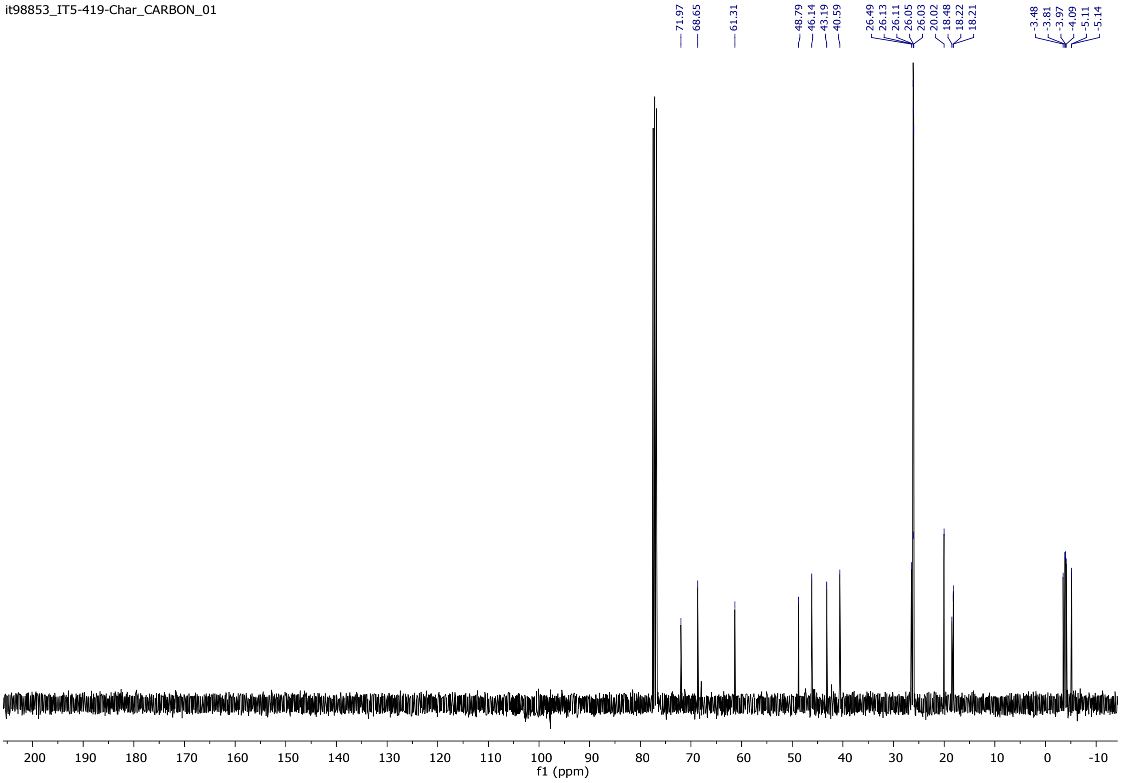

PMB protected acid 23

^{1}H NMR (400 MHz, $CDCl_3$)

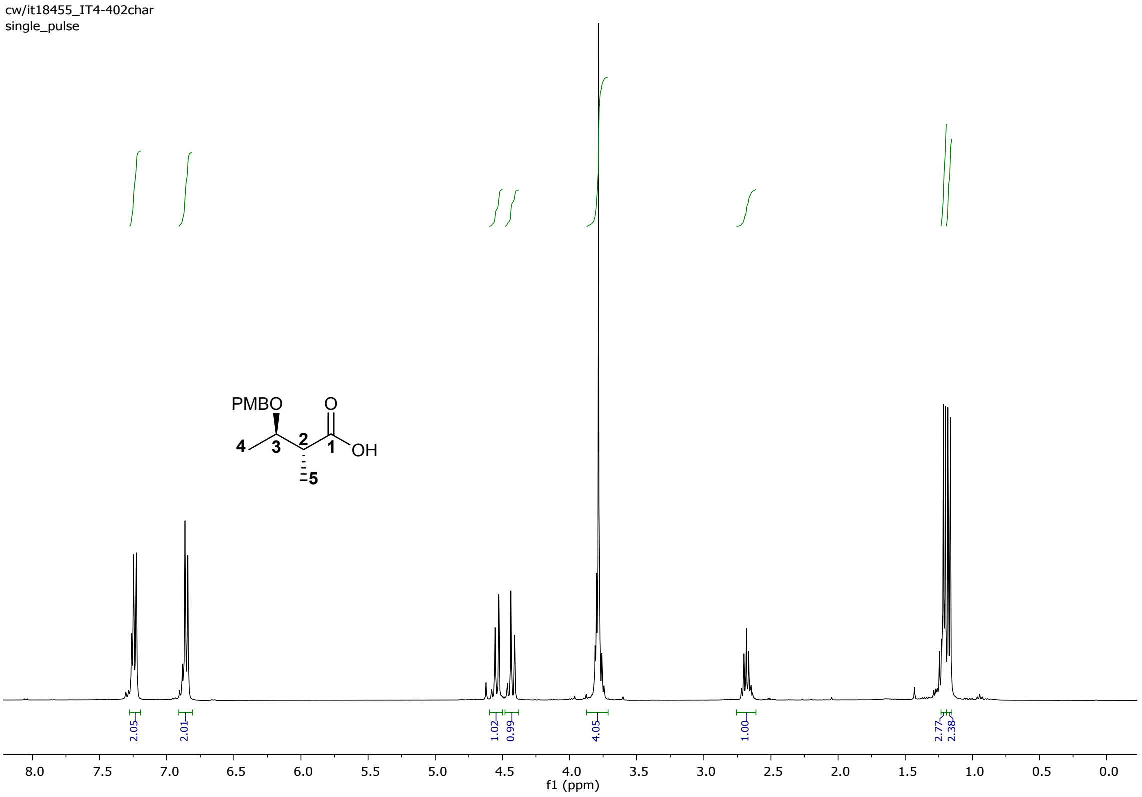

^{13}C NMR (125 MHz, $CDCl_3$)

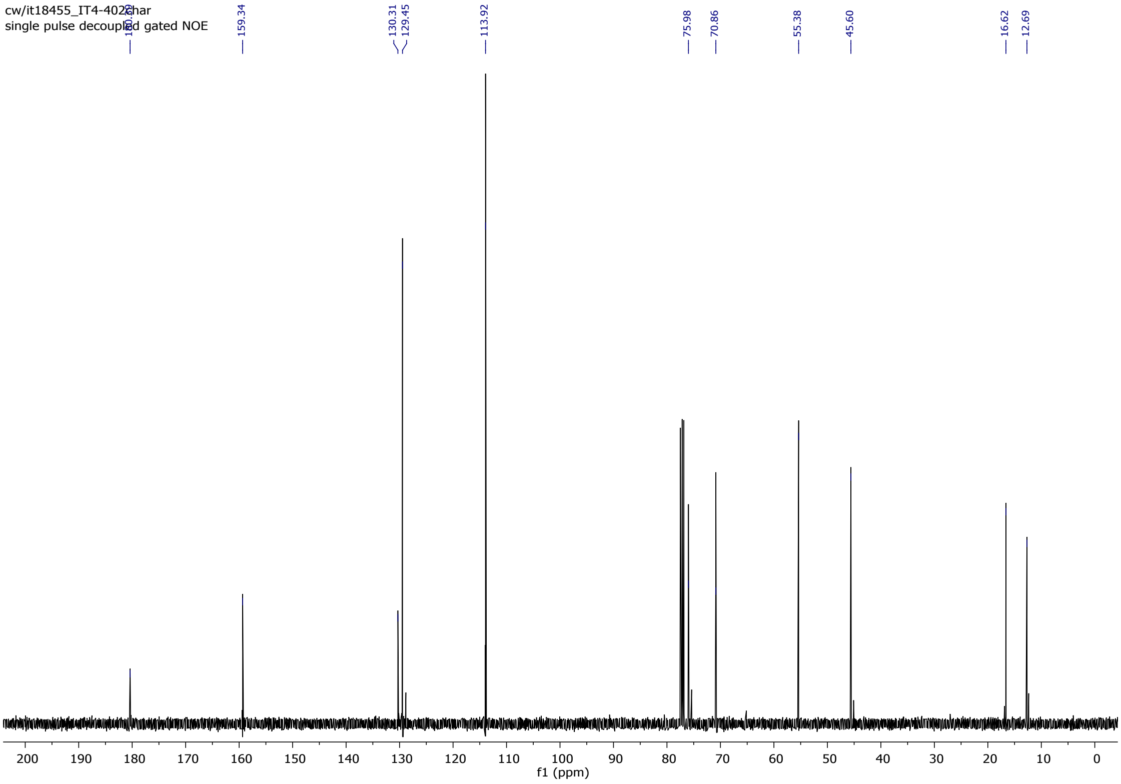

(10*R*, 11*R*)-11-(*p*-Methoxybenzyloxy)-10-methyl-*N*-((3*S*, 5*S*, 7*R*)-1,5,7-tri*(tert*-butyldimethylsilyloxy)-3-methyloctyl)butamide 24

^{1}H NMR (400 MHz, $CDCl_3$)

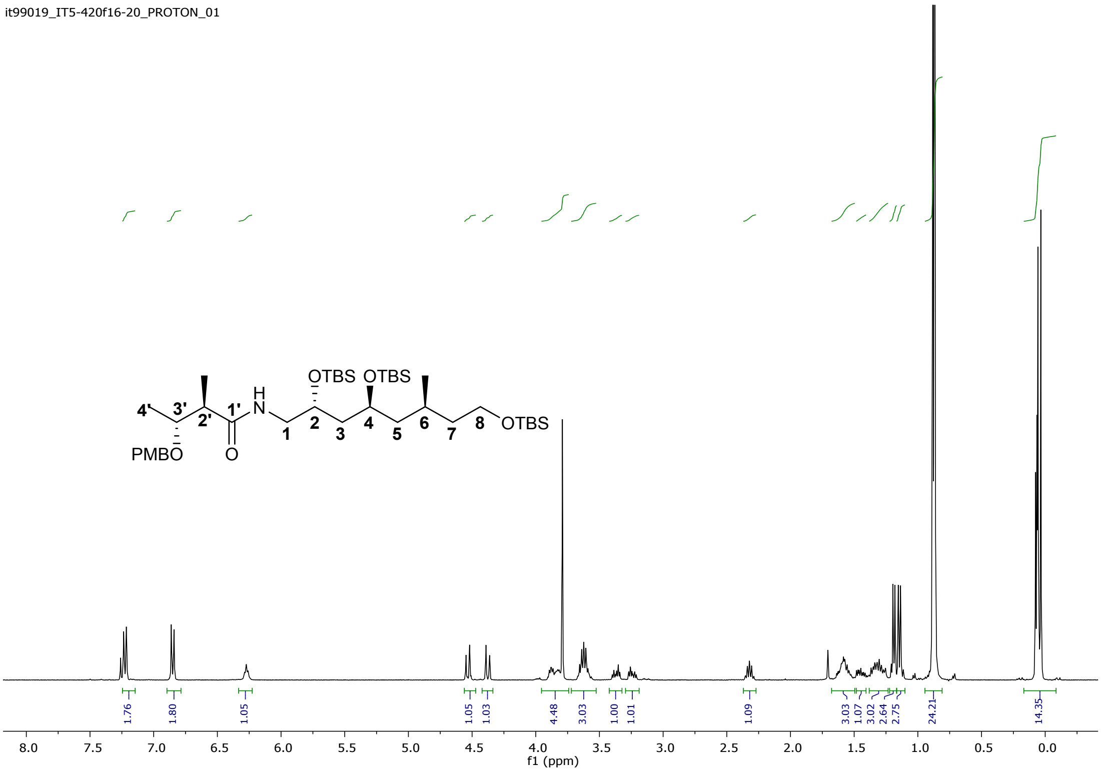

^{13}C NMR (125 MHz, $CDCl_3$)

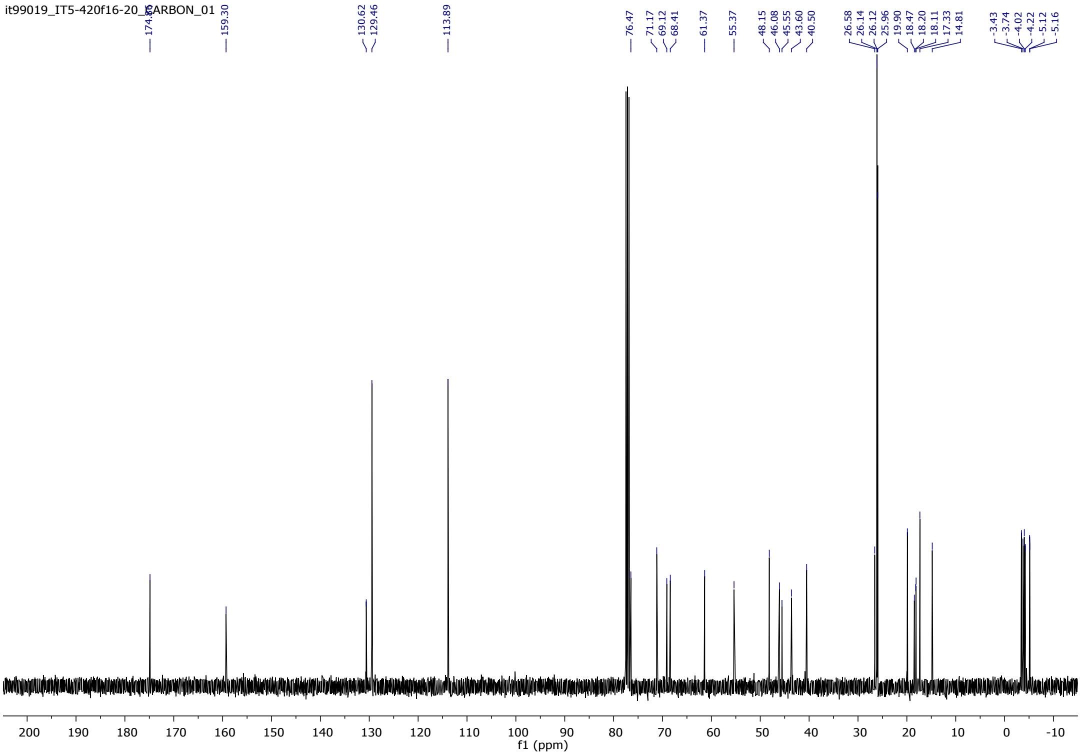

(10*R*, 11*R*)-11-(*p*-Methoxybenzyl)oxy)-10-methyl-*N*-((3*S*, 5*S*, 7*R*)-5,7-di*(tert*-butyldimethylsilyloxy)-1-hydroxy-3-methyl-octyl)butamide S29

^{1}H NMR (400 MHz, $CDCl_3$)

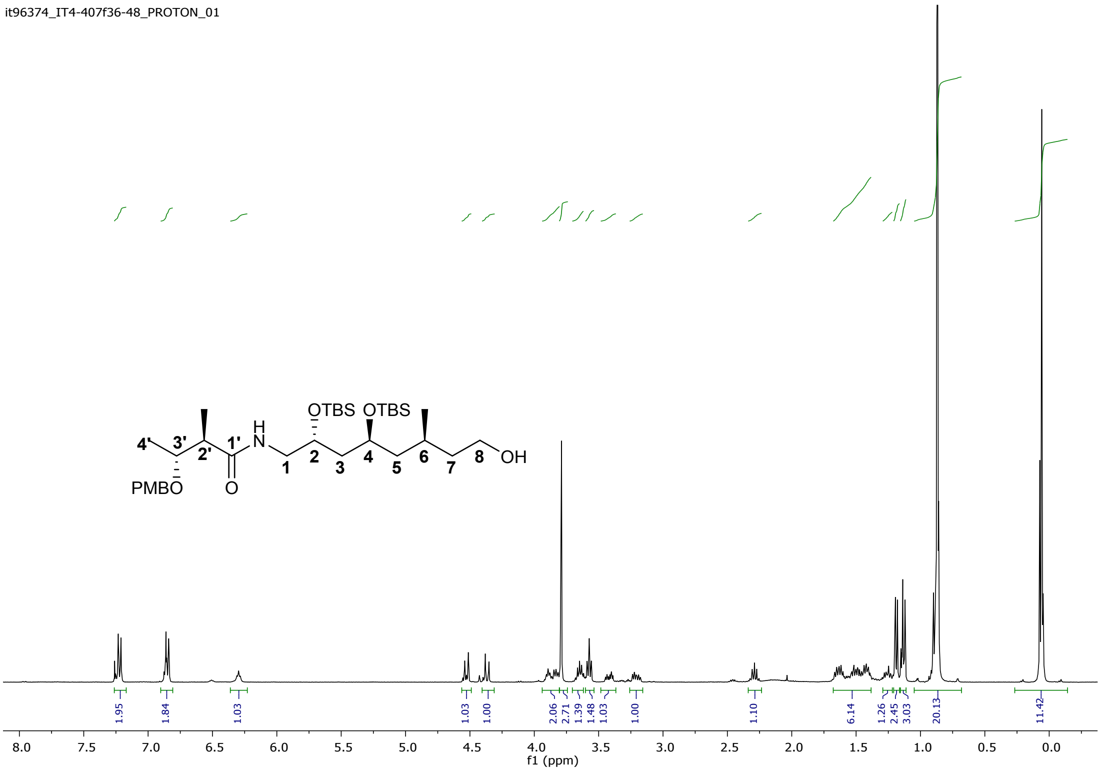

^{13}C NMR (125 MHz, $CDCl_3$)

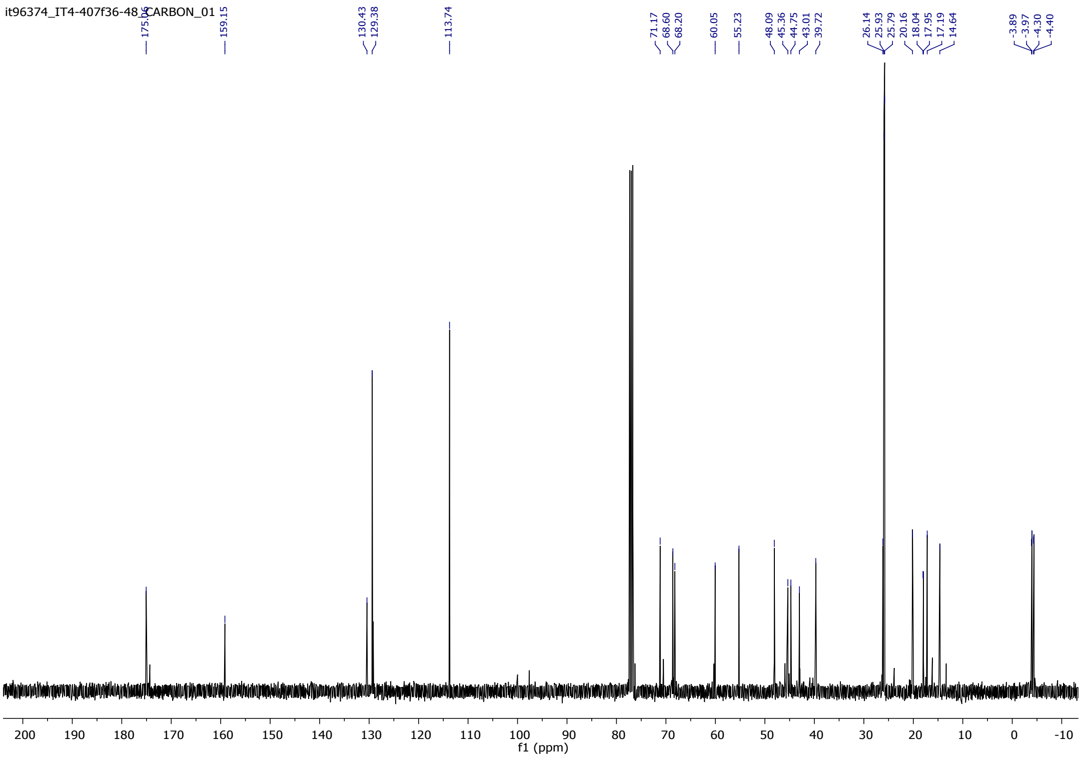

(3*S*, 5*S*, 7*R*)-5,7-Di*(tert*-butyldimethylsilyloxy)-8-(10*R*, 11*R*)-11-(*p*-methoxybenzyloxy)-10-methylbutamido-3-methyloctanoic acid 25

^{1}H NMR (400 MHz, $CDCl_3$)

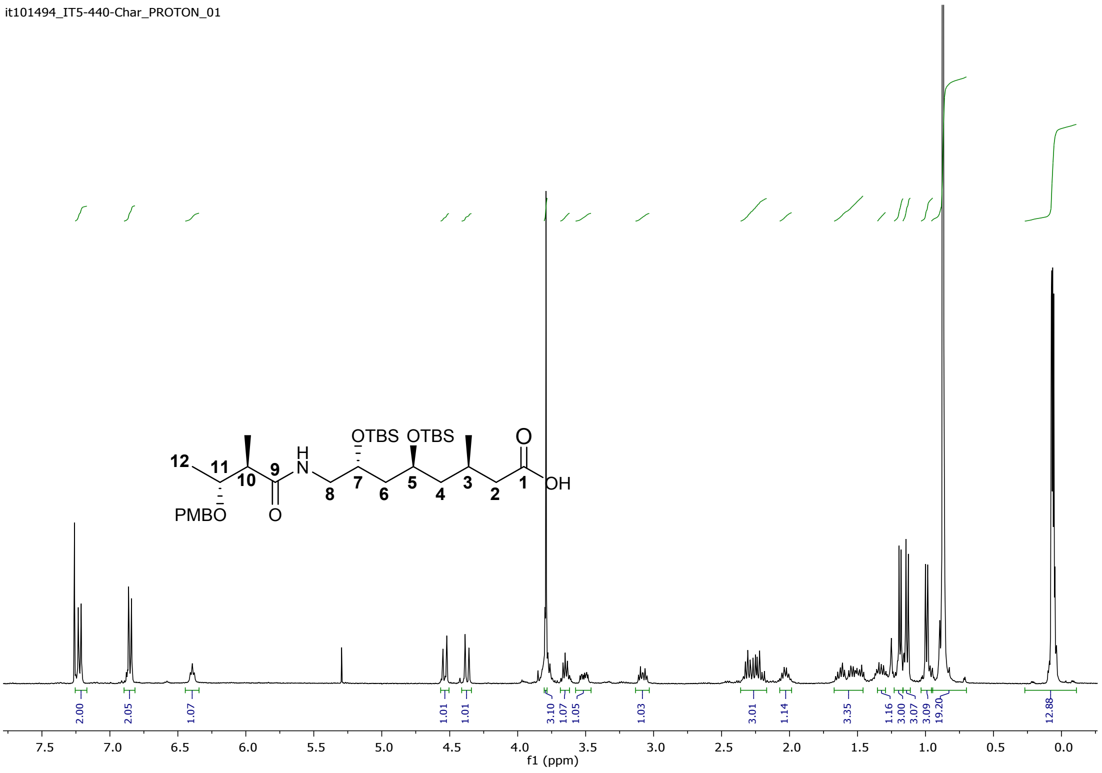

^{13}C NMR (125 MHz, $CDCl_3$)

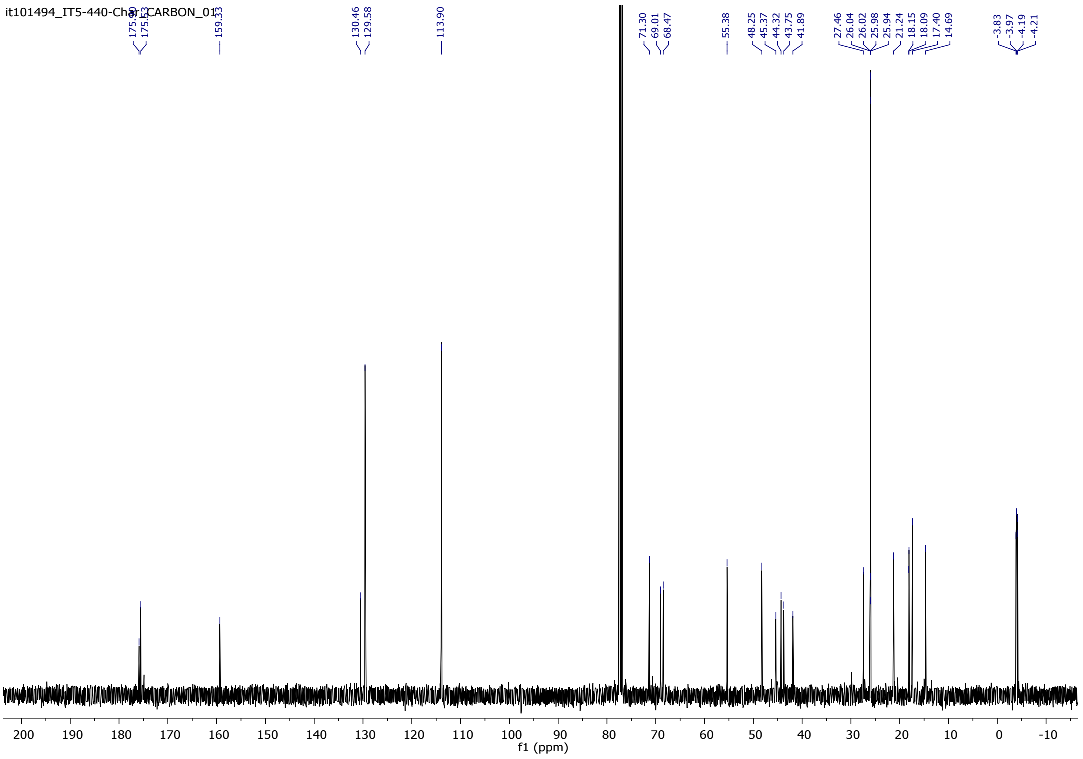

Lactone 26

^{1}H NMR (400 MHz, $CDCl_3$)

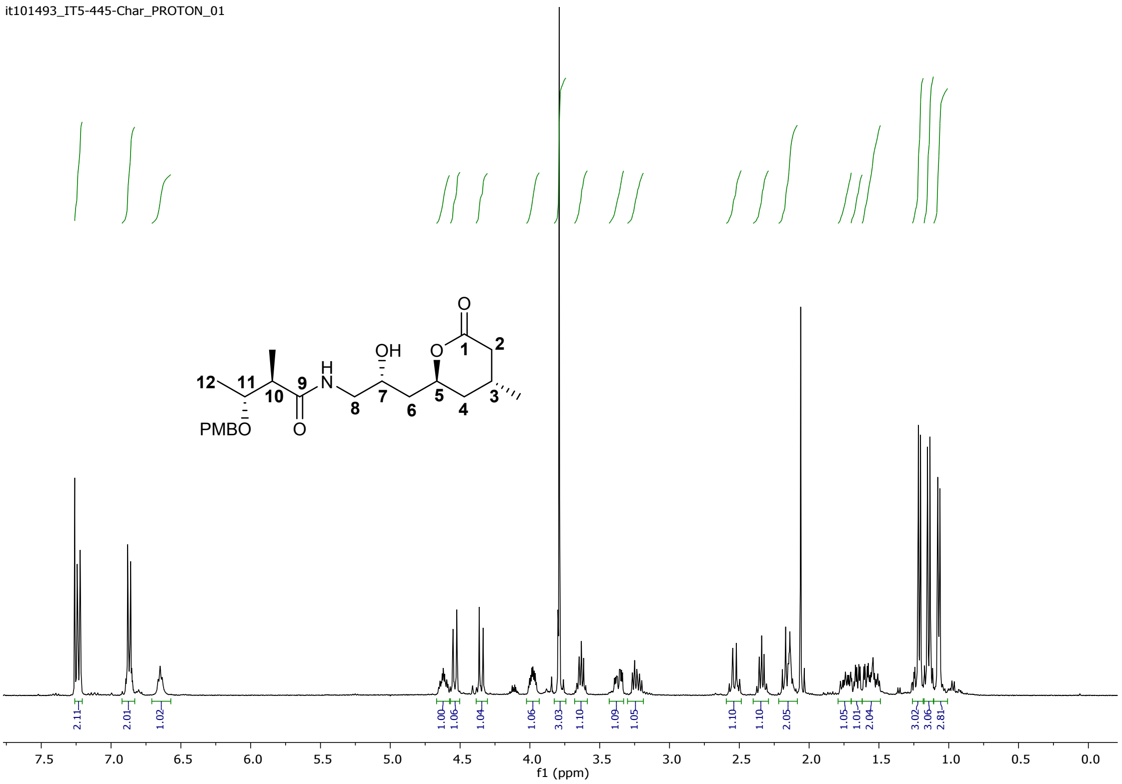

^{13}C NMR (125 MHz, $CDCl_3$)

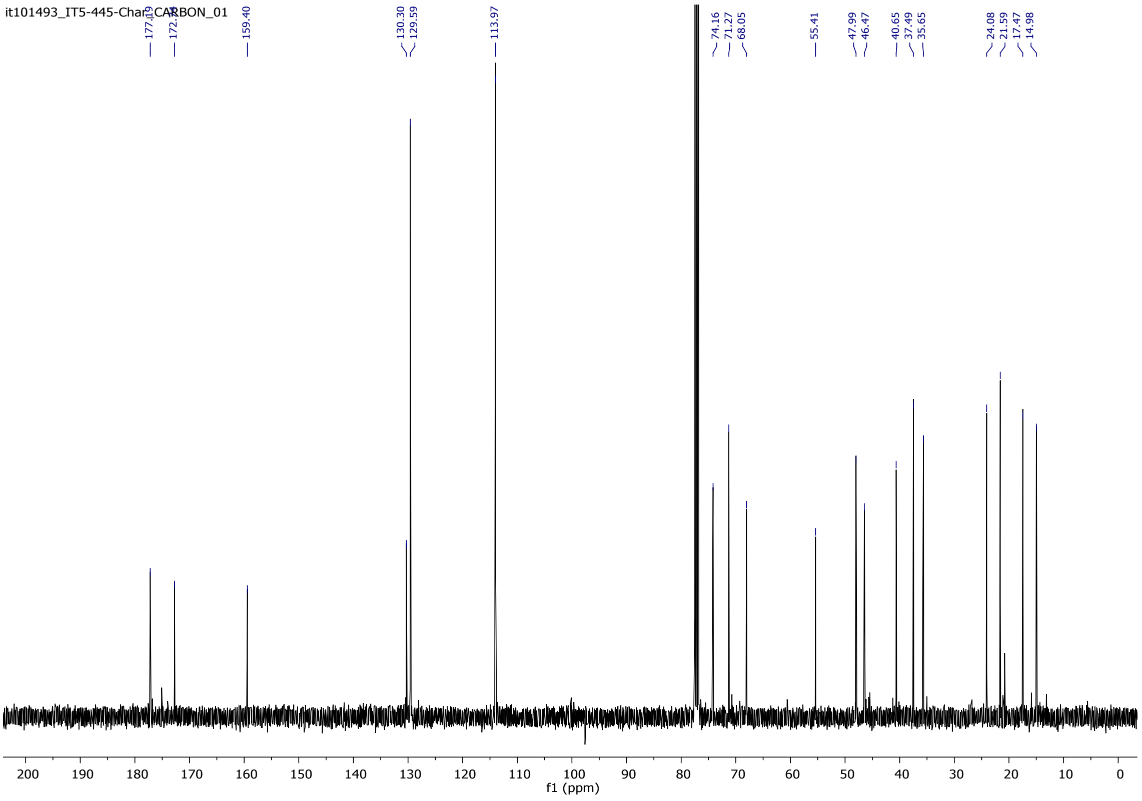

Fully protected lactone S30

^{1}H NMR (400 MHz, $CDCl_3$)

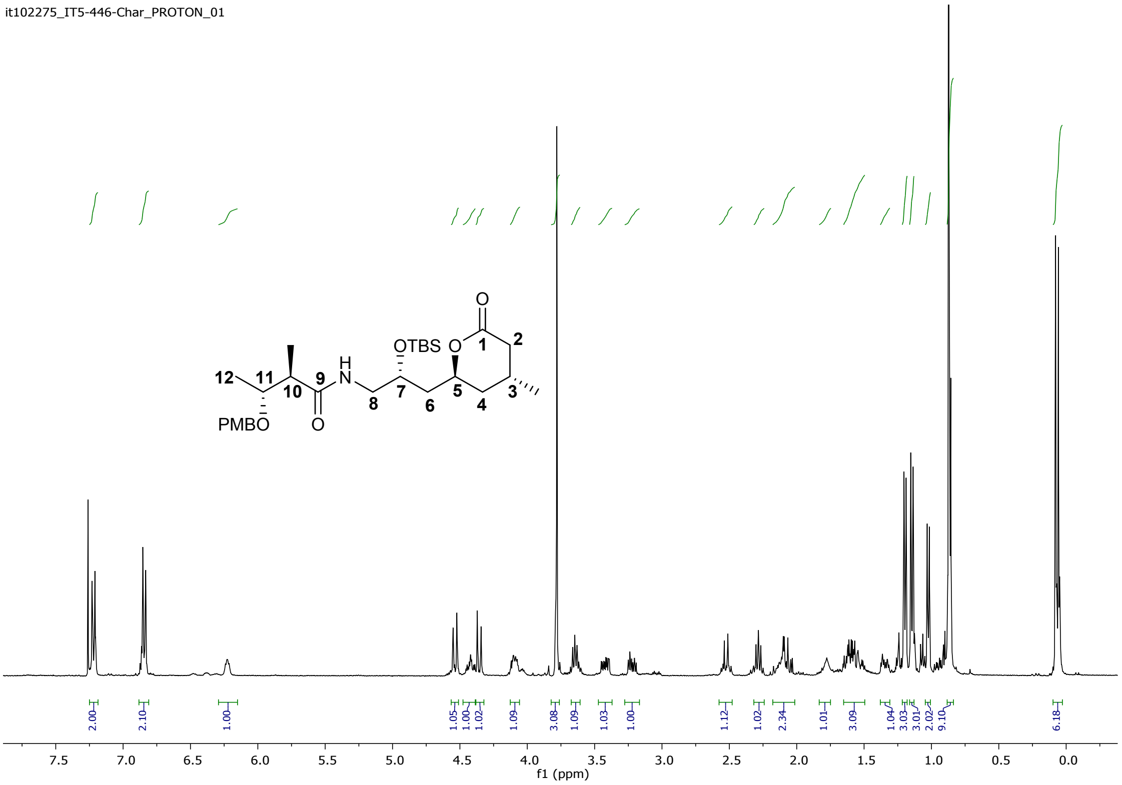

^{13}C NMR (125 MHz, $CDCl_3$)

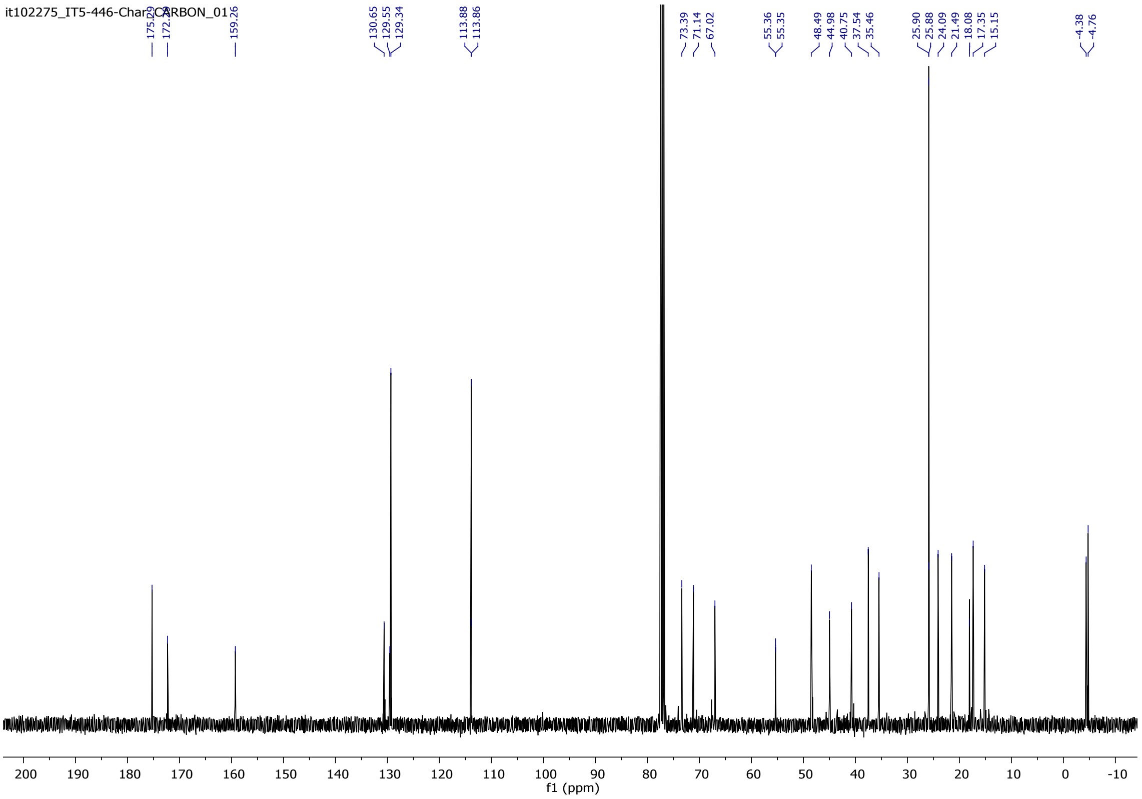

TBS protected lactone 27

^{1}H NMR (400 MHz, $CDCl_3$)

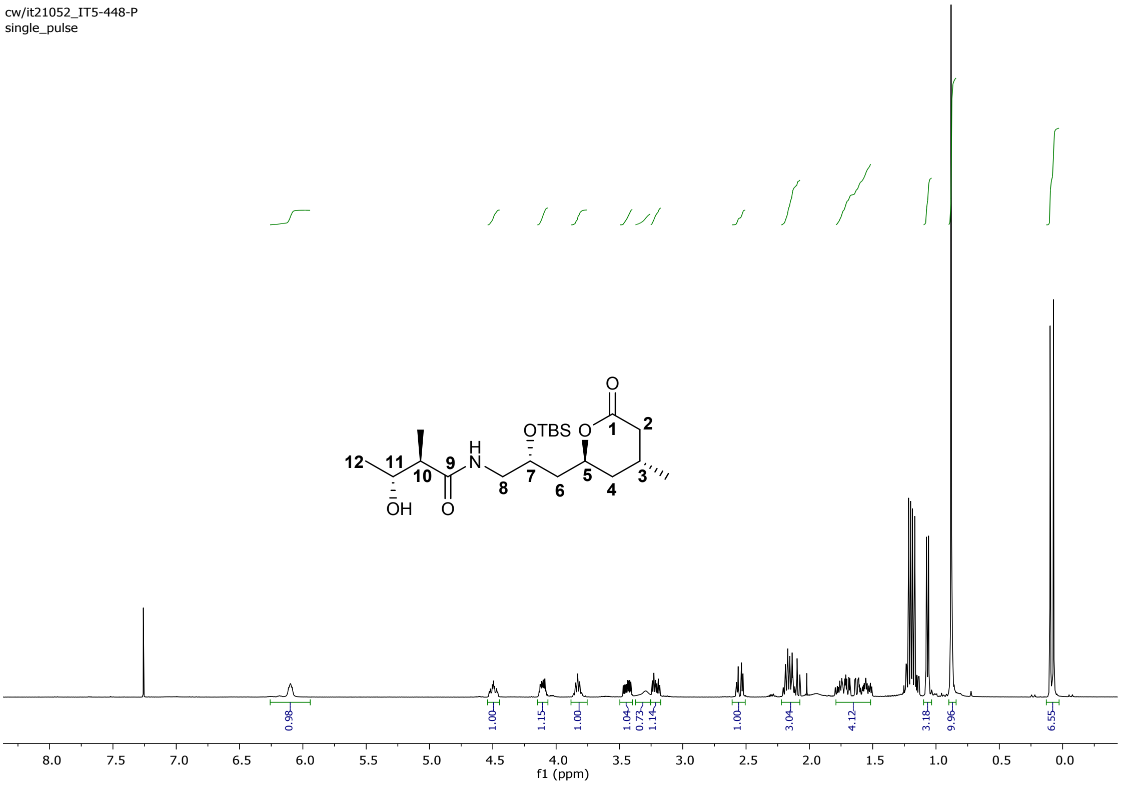

^{13}C NMR (125 MHz, $CDCl_3$)

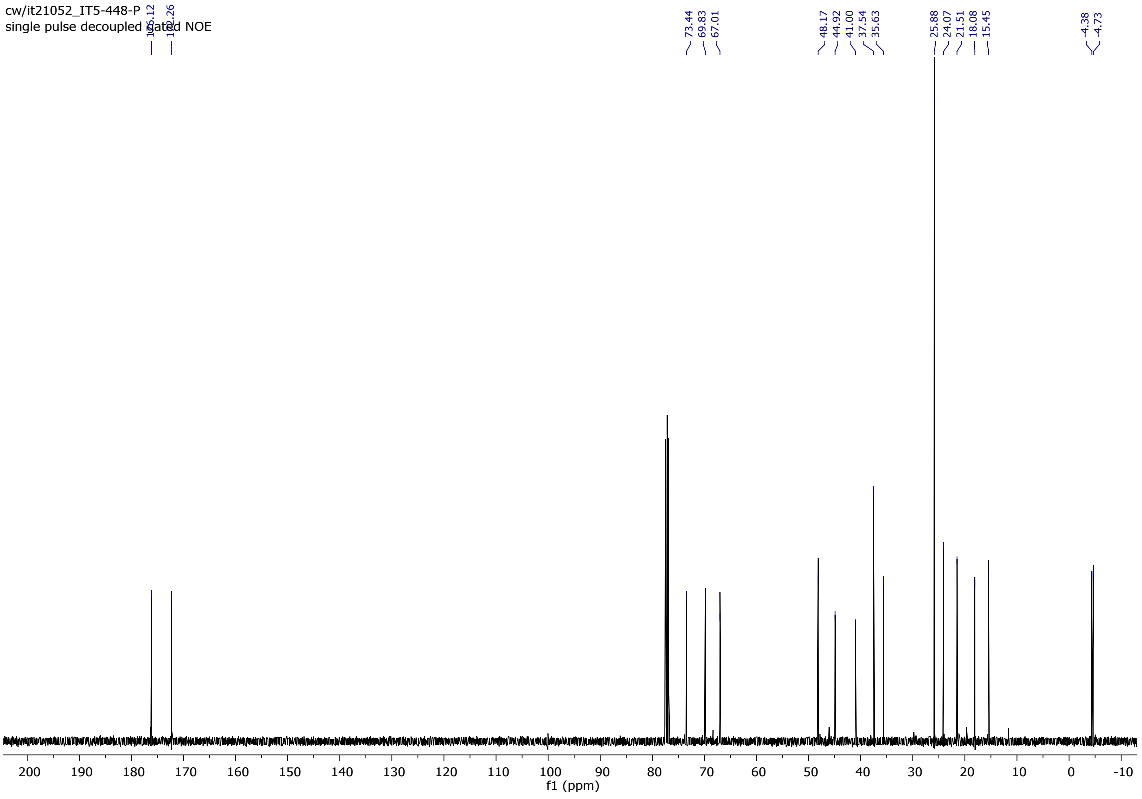

Protected lactone 28

^{1}H NMR (400 MHz, $CDCl_3$)

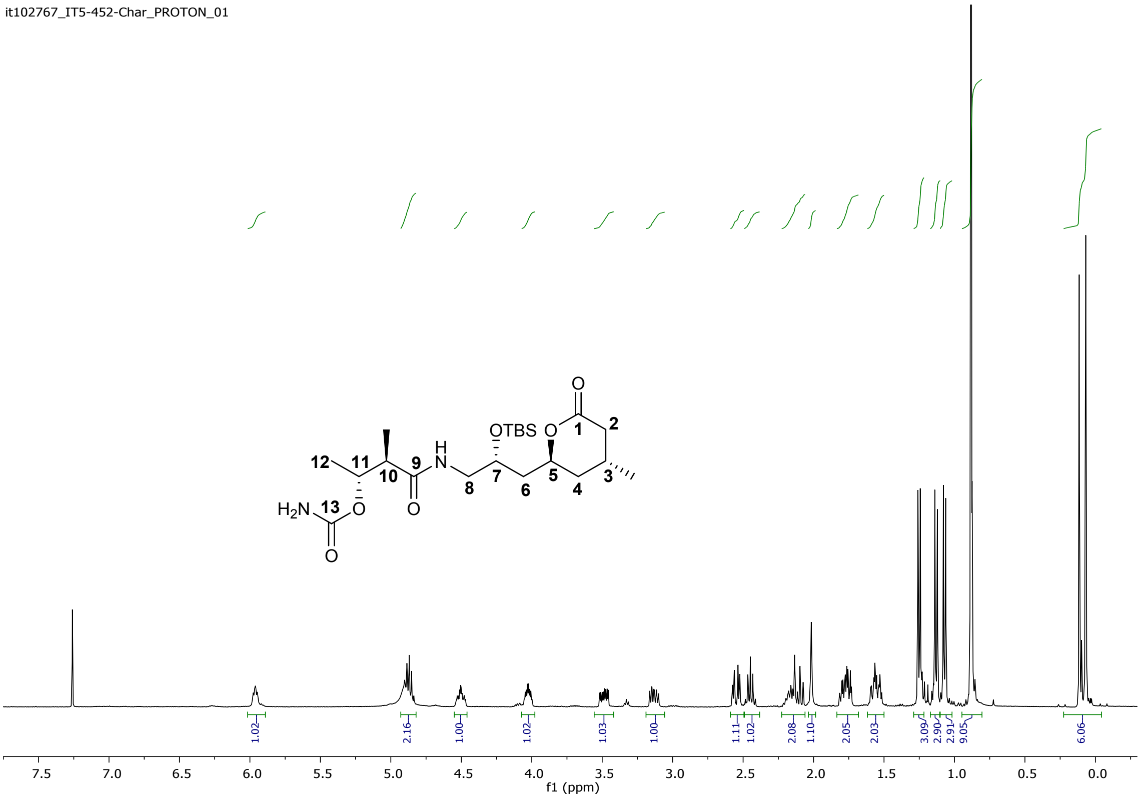

^{13}C NMR (125 MHz, $CDCl_3$)

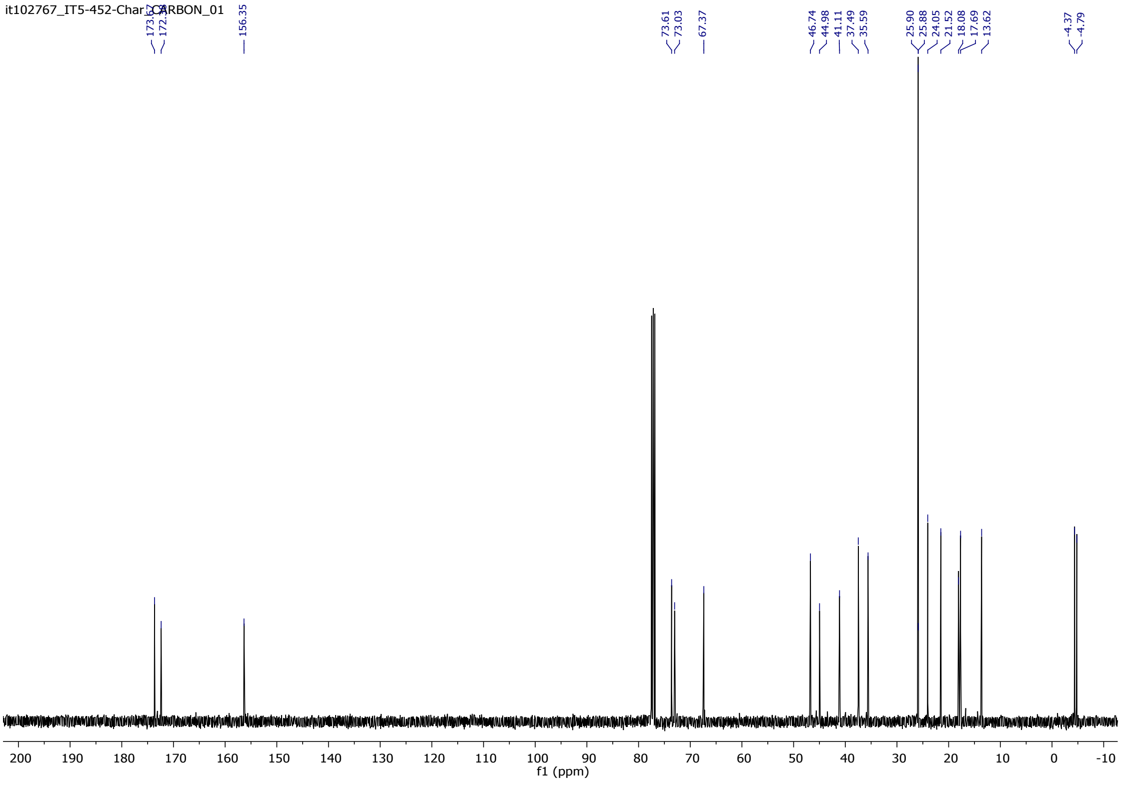

Synthetic lactone 10

^{1}H NMR (500 MHz, Methanol-d$_4$)

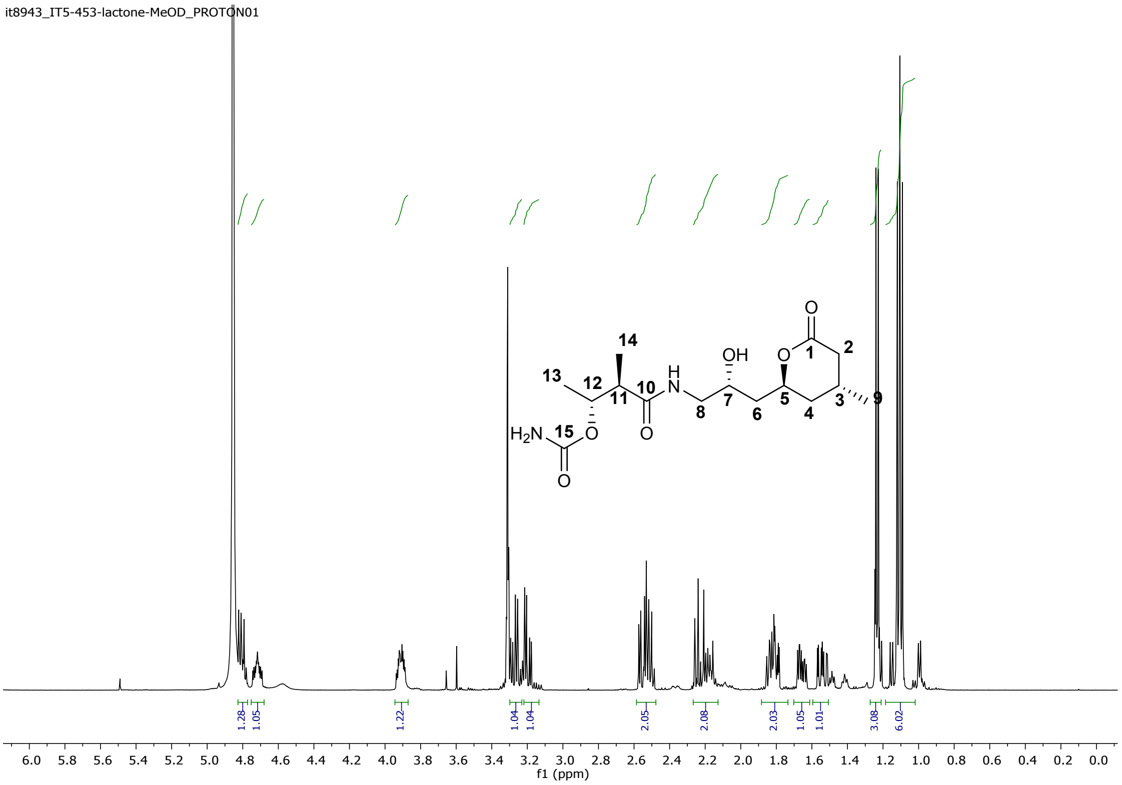

^{13}C NMR (125 MHz, methanol-d$_4$)

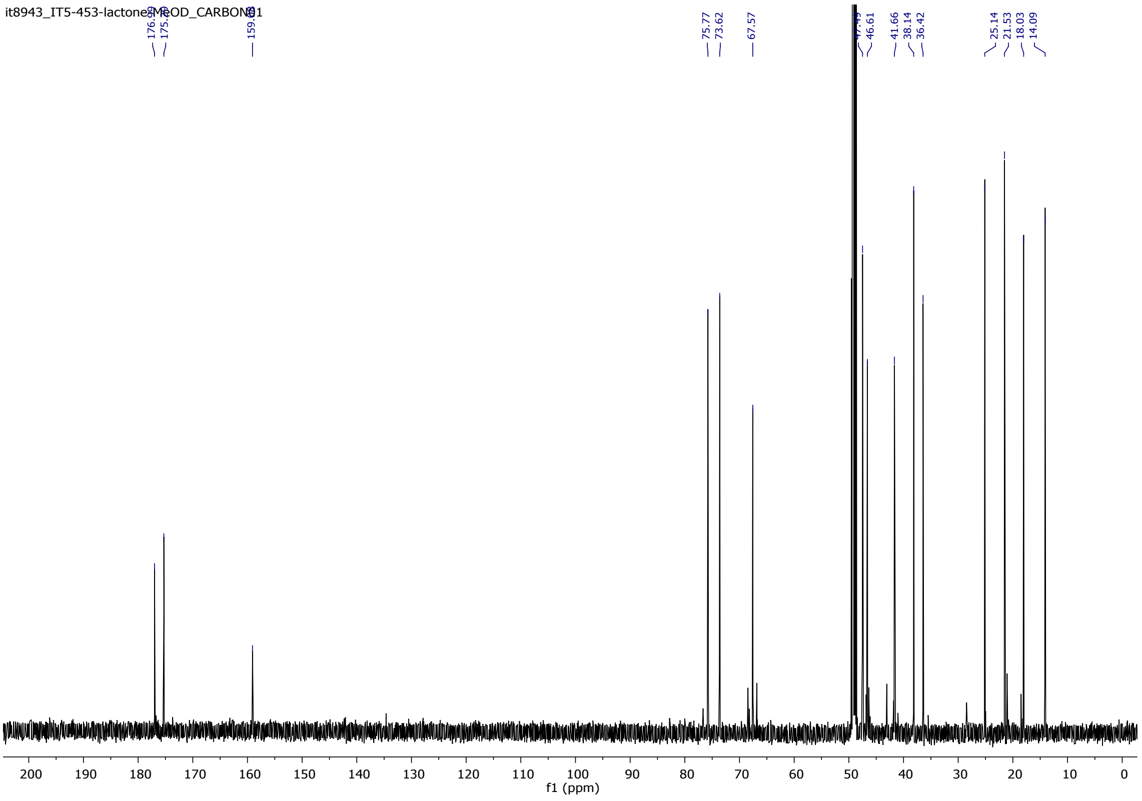

Ester by-product S31

^{1}H NMR (500 MHz, $CDCl_3$)

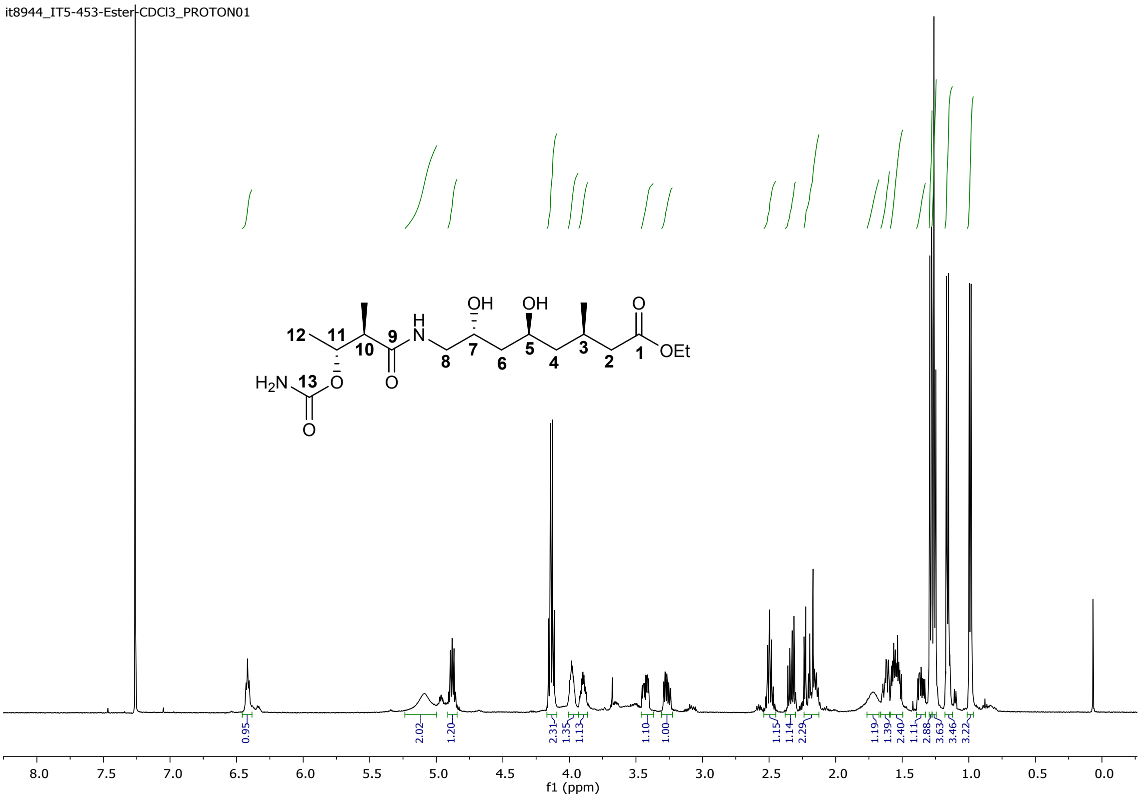

^{13}C NMR (125 MHz, $CDCl_3$)

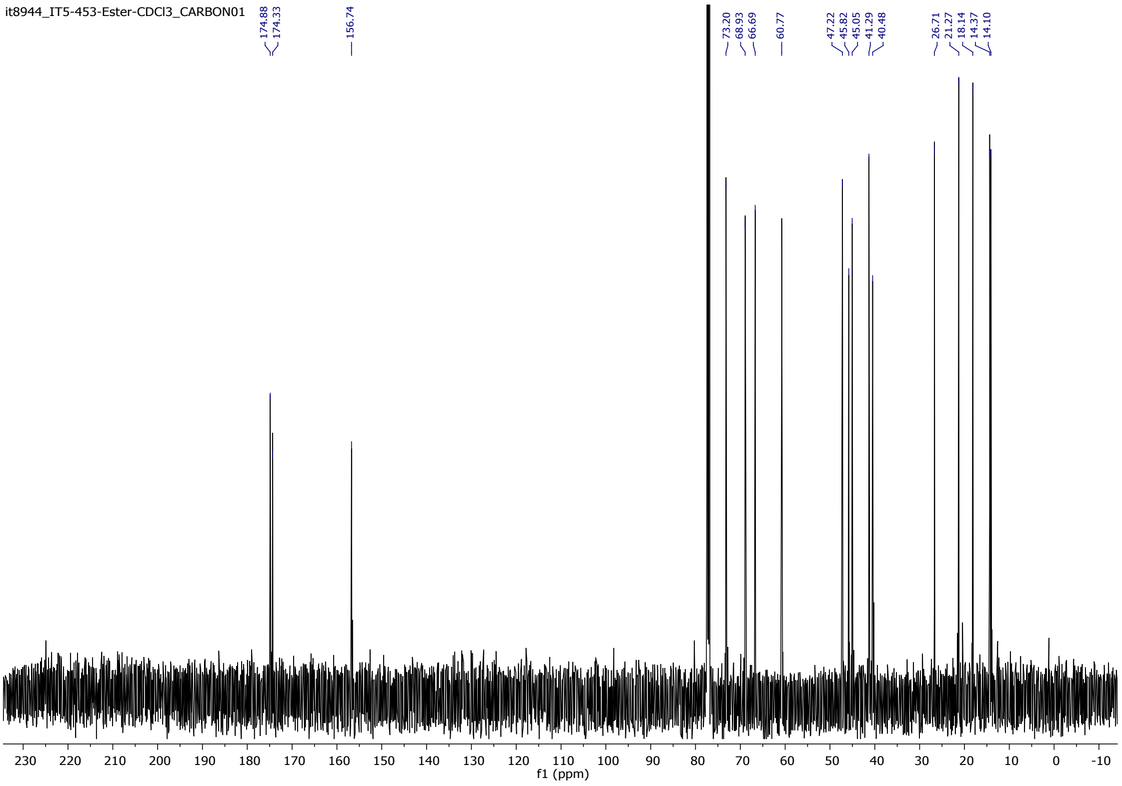

Supplement: Supplementary file 1 [file SC-008-C7SC01670K-s001.pdf]
